# **Major mistakes or errors in the use of trial sequential analysis in systematic reviews or meta-analyses – the METSA systematic review**

**DOI:** 10.1186/s12874-024-02318-y

**Published:** 2024-09-09

**Authors:** Christian Gunge Riberholt, Markus Harboe Olsen, Joachim Birch Milan, Sigurlaug Hanna Hafliðadóttir, Jeppe Houmann Svanholm, Elisabeth Buck Pedersen, Charles Chin Han Lew, Mark Aninakwah Asante, Johanne Pereira Ribeiro, Vibeke Wagner, Buddheera W. M. B. Kumburegama, Zheng-Yii Lee, Julie Perrine Schaug, Christina Madsen, Christian Gluud

**Affiliations:** 1 Copenhagen Trial Unit, Centre for Clinical Intervention Research, The Capital Region, Copenhagen University Hospital – Rigshospitalet, Blegdamsvej 9, Copenhagen, 2100 Denmark; 2 Department of Brain and Spinal Cord Injury, Neuroscience Centre, Copenhagen University Hospital – Rigshospitalet, Valdemar Hansens Vej 23, Glostrup, 2600 Denmark; 3 Department of Neuroanaesthesiology, Neuroscience Centre, Copenhagen University Hospital – Rigshospitalet, Blegdamsvej 9, Copenhagen, 2100 Denmark; 4 Bjarg Rehabilitation Center, Bugðusíðu 1, 603, Akureyri, Iceland; 5 Department of Gastrointestinal Surgery, Aalborg University Hospital South, Hobrovej 18-22, Aalborg, 9000 Denmark; 6 Department of Dietetics and Nutrition, Ng Teng Fong General Hospital, Singapore, Singapore; 7 Faculty of Health and Social Sciences, Singapore Institute of Technology, Singapore, Singapore; 8 Center for Evidence-Based Psychiatry, Psychiatric Research Unit, Psychiatry Region Zealand, Faelledvej 6, Slagelse, 4200 Denmark; 9 Department of Psychology, Faculty of Health Sciences, University of Southern Denmark, Campusvej 55, 5230, Odense, Denmark; 10 Department of Clinical Medicine, Faculty of Health and Medical Sciences, University of Copenhagen, Copenhagen, Denmark; 11 Department of Anaesthesiology, Faculty of Medicine, University of Malaya, Kuala Lumpur, Malaysia; 12 Department of Cardiac Anesthesiology & Intensive Care Medicine, Charité, Berlin, Germany; 13 Psychiatric Research Unit, Psychiatry Region Zealand, Region Zealand, Fælledvej 6, Slagelse, 4200 Denmark; 14 Department of Regional Health Research, The Faculty of Health Sciences, University of Southern Denmark, Odense, Denmark

**Keywords:** Meta-analysis, Methodology, Research-on-research, Systematic review, Trial sequential analysis

## Abstract

**Background:**

Systematic reviews and data synthesis of randomised clinical trials play a crucial role in clinical practice, research, and health policy. Trial sequential analysis can be used in systematic reviews to control type I and type II errors, but methodological errors including lack of protocols and transparency are cause for concern. We assessed the reporting of trial sequential analysis.

**Methods:**

We searched Medline and the Cochrane Database of Systematic Reviews from 1 January 2018 to 31 December 2021 for systematic reviews and meta-analysis reports that include a trial sequential analysis. Only studies with at least two randomised clinical trials analysed in a forest plot and a trial sequential analysis were included. Two independent investigators assessed the studies. We evaluated protocolisation, reporting, and interpretation of the analyses, including their effect on any GRADE evaluation of imprecision.

**Results:**

We included 270 systematic reviews and 274 meta-analysis reports and extracted data from 624 trial sequential analyses. Only 134/270 (50%) systematic reviews planned the trial sequential analysis in the protocol. For analyses on dichotomous outcomes, the proportion of events in the control group was missing in 181/439 (41%), relative risk reduction in 105/439 (24%), alpha in 30/439 (7%), beta in 128/439 (29%), and heterogeneity in 232/439 (53%). For analyses on continuous outcomes, the minimally relevant difference was missing in 125/185 (68%), variance (or standard deviation) in 144/185 (78%), alpha in 23/185 (12%), beta in 63/185 (34%), and heterogeneity in 105/185 (57%). Graphical illustration of the trial sequential analysis was present in 93% of the analyses, however, the Z-curve was wrongly displayed in 135/624 (22%) and 227/624 (36%) did not include futility boundaries. The overall transparency of all 624 analyses was very poor in 236 (38%) and poor in 173 (28%).

**Conclusions:**

The majority of trial sequential analyses are not transparent when preparing or presenting the required parameters, partly due to missing or poorly conducted protocols. This hampers interpretation, reproducibility, and validity.

**Study registration:**

PROSPERO CRD42021273811

**Supplementary Information:**

The online version contains supplementary material available at 10.1186/s12874-024-02318-y.

**Box 1:** Pre-defined parameters for trial sequential analysis

To control for the risk of type I and type II errors, a diversity adjusted required information size, and correspondingly adjusted confidence intervals can be calculated using trial sequential analysis. This is particularly relevant when an adequate sample size for the meta-analysis has not yet been reached. Trial sequential analysis is sensitive to the selected parameter values, which therefore need to be predefined, preferable in a protocol made publicly available before the review is conducted (planned prospectively) to minimize risk of bias. As most meta-analyses are moving towards a required information size, the power can be insufficient, and they should be considered as interim meta-analyses.	
For dichotomous outcomes, researchers need to pre-define the following parameters in a protocol:	
*Proportion of events in the control group* – An estimated number for the control event rate ideally taken from previous systematic reviews, randomised clinical trials at low risk of bias, or from the meta-analysis itself. How this value was chosen should be clearly stated in the protocol for each relevant outcome.	
*Relative risk reduction* – The absolute risk reduction divided by the control event rate should ideally be taken from previous systematic reviews or randomised clinical trials at low risk of bias, or a clinical meaningful and realistic effect, when the former is not possible. This should be clearly stated in the protocol for each relevant outcome, including how this value was reached.	
*Alpha level* – The risk of committing type I errors (concluding effect when there is none). Traditionally set at 5% [1], but considerations on former published research, and the number of outcomes (to account for multiplicity) should be considered. The intended value for each outcome should be clearly stated if possible.	
*Power (1–beta)* Beta is the risk of committing type II errors (concluding absence of effect when there is an effect). Power is 1 – beta. Traditionally set at 80% in trials but at a meta-analytical level, it is recommended to consider at least 90% power. The intended value for each outcome should be clearly stated.	
*Heterogeneity correction* – If heterogeneity exists in the forest plot, trial sequential analysis can adjust for heterogeneity. It is recommended to use *diversity* as this is a more accurate measure of heterogeneity between trials than inconsistency. The required information size will then be termed diversity adjusted required information size (DARIS). Protocols should clearly disclose the method chosen to estimate the statistical heterogeneity.	
For continuous outcomes, researchers need to pre-define the following parameters in a protocol:	
*Minimally relevant difference* – The magnitude of change that is meaningful for the patient, should ideally be derived from relevant benchmark studies, if possible, previous systematic reviews, or randomised clinical trials at low risk of bias as alternatives. It should be clearly stated in the protocol for each relevant outcome, including how this value was reached.	
*Variance* – The variance of the continuous outcome in the control group. It should be clearly stated in the protocol for each outcome, including how this value was or will be calculated.	
*Alpha level* – The risk of committing type I errors (concluding effect when there is none). Traditionally set at 5%, but considerations on former published research, and number of outcomes (to account for multiplicity) should be considered. The intended value for each outcome should be clearly stated.	
*Power (–Beta)* Beta is the risk of committing type II errors (concluding absence of effect when there is an effect). Power is 1 – beta. Traditionally set at 80% but at a meta-analytical level it is recommended to consider at least 90% power. The intended value for each outcome should be clearly stated.	
*Heterogeneity correction* – If statistical heterogeneity exists in the forest plot, Trial Sequential Analysis can adjust for heterogeneity. It is recommended to use *diversity* as this is a more accurate measure of heterogeneity between trials than inconsistency. The required information size will then be termed diversity-adjusted required information size (DARIS). Protocols should clearly disclose the method chosen to estimate the statistical heterogeneity.	
All parameters are used to calculate the *required information size* in the trial sequential analysis. The arial sequential analysis can conclude whether there is benefit or harm from results before the required information size is reached (Z-curve breaching the monitoring boundaries). Futility should be concluded if no difference is present with sufficient accrued number of participants (i.e. the Z-curve penetrates the futility boundaries). A trial sequential analysis-adjusted confidence interval can be calculated and used for imprecision rating in GRADE instead of the naïve 95% confidence interval.	
For examples of adequately reported trial sequential analyses we can refer to review by Goh et al. (2019) [64] and protocol by Stokes et al. (2016) [65].	
Besides the parameters defined above, standard considerations from conducting systematic reviews should be defined (e.g. specifying the research hypothesis, handling of zero events, doing one- or two-sided tests). For more information on this we refer to the Cochrane Handbook [1].	

## Introduction

Systematic reviews with meta-analysis play a major role when producing guidelines for clinical practice, research, and health policy, and the publication rate is growing [1–7]. Systematic reviews of randomised clinical trials should be conducted in a structured way using a publicly available protocol published before the review is conducted to minimise bias and include all relevant literature [1, 7]. When sufficient data are available, it is possible to meta-analyse data to determine the cumulative estimate of all randomised clinical trials, under the assumption that the systematic review is adequately conducted [8].

Underpowered studies are at high risk of type I and type II errors [9–12]. In a systematic assessment of Cochrane reviews, Turner and colleagues showed that 70% of the meta-analyses had less than 50% power to detect a 30% relative risk reduction [10]. Similar results have been found in non-Cochrane systematic reviews [13]. Updating systematic reviews is often a necessity as data from new randomised clinical trials appear, but the resulting repeated significance testing increases the risk of random errors. This is like interim analysis in a single randomised clinical trial [14].

A team at the Copenhagen Trial Unit developed the Trial Sequential Analysis programme to calculate a required information size in the meta-analyses of the systematic review to control for type I and type II errors with Lan-DeMets-O’Brian-Fleming monitoring boundaries for benefit, harm, and futility [15–18]. In random-effects meta-analyses, one can adjust the required information size with the heterogeneity, termed the diversity-adjusted required information size (DARIS) [15, 19]. As with all statistical methods, transparency is essential for replicability and reliable interpretation. Since the first version of the Trial Sequential Analysis software in 2008 [16], several systematic reviews and meta-analysis reports have included Trial Sequential Analysis in their data synthesis. Cochrane recognises and endorses the role of trial sequential analysis as a secondary analysis to provide additional interpretation, but only if planned prospectively with a complete analysis plan in the protocol [1, 20, 21].

Systematic reviews are commonly retrospective in nature, that is, all or some of the results are known before completing the analysis. This can potentially cause sequential decision bias, as the known results can affect decisions in the planning of the sequential analysis [22].

To gain sufficient power in randomised clinical trials, institution review boards mandate reporting all parameters required for the sample size estimation of randomised clinical trials [23]. Such requirements should also be upheld in systematic reviews using Trial Sequential Analysis. Accordingly, parameters for sequential testing such as minimal important effect size, relative risk reduction, alpha, beta, and heterogeneity should be reported before data synthesis [11, 24, 25]. The completeness and transparency of reporting the above-mentioned parameters in published systematic reviews have not been empirically evaluated.

In preparation for developing updated guidelines for using Trial Sequential Analysis [11], we systematically assessed the use of the trial sequential analysis across all medical fields, groups of patients, interventions, comparators, and outcomes in systematic reviews and meta-analyses [26]. We aimed to evaluate how the authors protocolised, reported, and interpreted the obtained results to improve transparency of trial sequential analyses in future systematic reviews. This effort would benefit clinical practitioners, decision-makers, and patients as transparency is a key for more unbiased decision making.

## Methods

We submitted the protocol to Systematic Reviews [23] and made it publicly available on the 13th of September 2021 through ResearchSquare (10.21203/rs.3.rs-900530/v1) before completing the initial screening of studies and before starting data extraction. The protocol was also prospectively registered on PROSPERO (CRD42021273811) on the 18th of September 2021 and Research-on-Research on the 7th of March 2023 (https://ror-hub.org/study/3032/). The reporting of this research-on-research study adheres to the reporting guidelines of the Preferred Reporting Items for Systematic Reviews and Meta-Analyses (PRISMA) (Supplemental Table 1) [27] and the PRISMA-S checklist for reporting literature searches [28].

### Types of studies

We included all peer-reviewed publications of systematic reviews and meta-analysis reports. Here, a systematic review is defined as a detailed, organised, and transparent method of gathering, appraising and synthesising data to answer a well-defined question, including public registration and/or a pre-published protocol before starting data extraction [1]. We defined a meta-analysis report as a non-systematic approach which lacks registration and/or a publicly available protocol before data extraction. We included all systematic reviews or meta-analysis reports of randomised clinical trials that included at least one trial sequential analysis. We included studies published between 1st of January 2018 and 31st of December 2021 with at least two randomised clinical trials in at least one conventional forest plot and one trial sequential analysis. The time frame was chosen to retrieve the most recent studies and retrieving 400 to 600 studies. For practical reasons, only studies in English were included in the study.

### Types of participants, interventions, comparators, and outcomes

Our focus was on methodological considerations, thus participants of any race, sex, age, or with any disease or condition were considered. All types of interventions and comparators were accepted for this review, and we did not have any restrictions on the types of outcomes.

### Search strategy

We searched the following databases: Medical Literature Analysis and Retrieval System Online (MEDLINE) and The Cochrane Database of Systematic Reviews (CDSR). We used the following keywords ((trial sequential and (analys* or monitoring boundar*)) or cumulative meta-analys*). The full search strategies are presented in Supplemental Table 2. A preliminary search was conducted on the 9th of July 2021 and the final search was conducted on the 28th of March 2022. We chose not to perform citation search as a sufficient number of studies was already included.

### Selection of studies

**Fig. 1:**
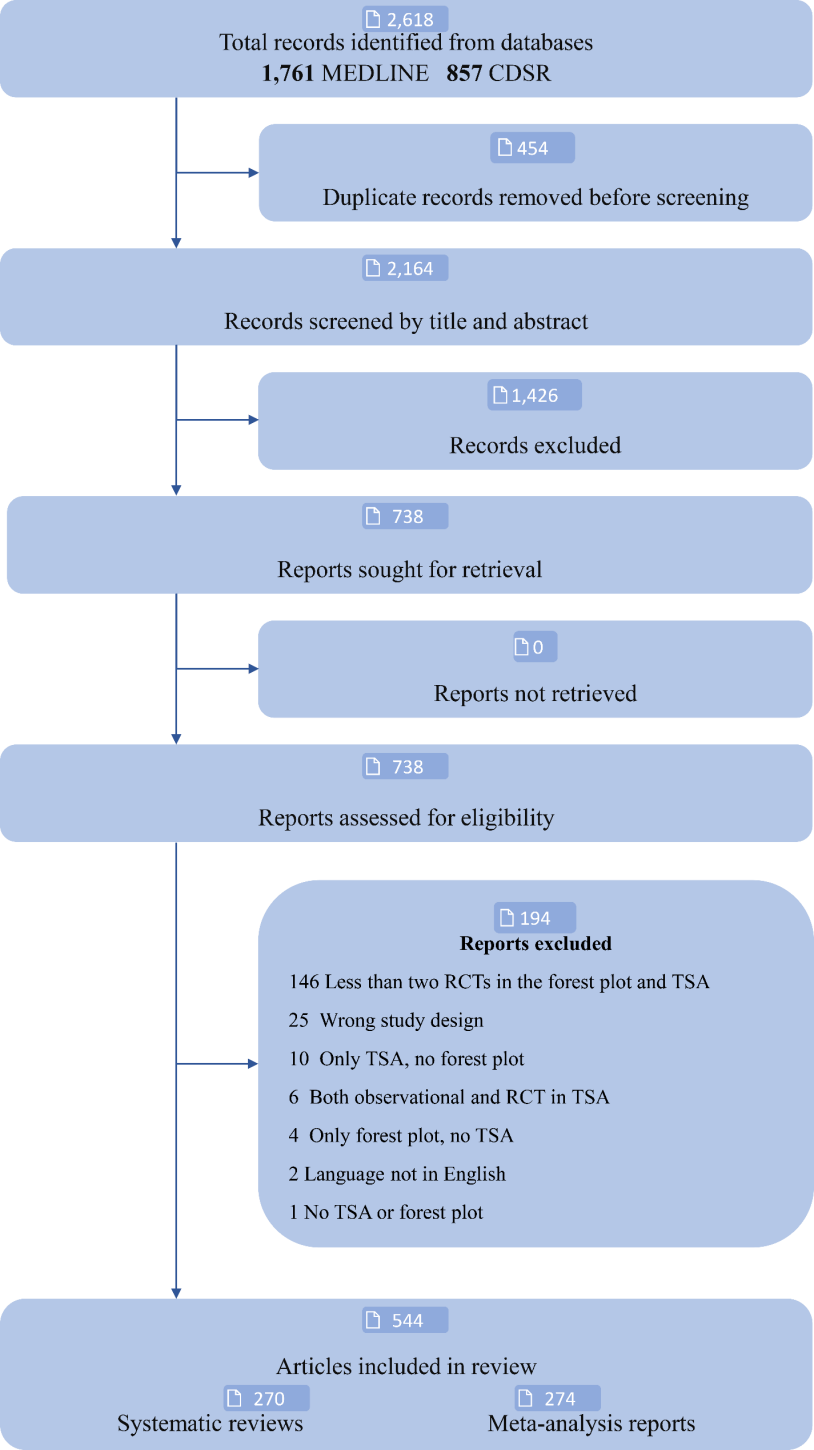
Flowchart. MEDLINE: Medical Literature Analysis and Retrieval System online; CDSR: Cochrane Database of Systematic Reviews; RCT: randomised clinical trial; TSA: Trial Sequential Analysis

Two authors (CGR and MHO) screened the titles and abstracts of all identified studies using Covidence (www.covidence.org, Melbourne, Australia) [29]. All relevant full-text studies were screened for eligibility, and reasons for exclusion were recorded (Fig. 1). Any discrepancy was resolved through discussion between the two authors. If an agreement was not reached, a third author (CG) would act as arbitrator. References for studies excluded during the full-text screen can be found in Supplemental Table 3.

### Extraction of data and quality assessment

Thirteen researchers participated in the extractions and extracted data independently and in pairs of two (CGR, JBM, SHH, JHS, EBP, CCHL, MAA, JPR, VW, BK, ZYL, JPS, CM). The authors assigned themselves for study assessment on an ad-hoc basis. After extracting data, consensus was sought between the two extractors of each study. Weekly online meetings were held during the data extraction to develop and maintain a high internal validity. At these meetings, examples and interpretations of data were discussed, and consensus was reached in the project group.

Data extractions were carried out using a standardised data extraction form in REDCap (Research Electronic Data Capture, University of Kansas, United States) hosted at Rigshospitalet, Denmark [30, 31]. The data extraction forms were divided into five major categories: (1) study details (authors, year, country, documents used for evaluation, study type, medical fields, intervention and overall goal of intervention, specific diagnosis or health issue); (2) Assessing the methodological quality of systematic reviews 2 (AMSTAR 2) [32]; (3) study description (date of search, number of outcomes, subgroup analyses performed, outcomes to which trial sequential analysis were applied, number of trials included in the review and in the largest meta-analysis, details on Grading of Recommendations Assessment, Development and Evaluation (GRADE) [33]); (4) Trial sequential analysis description (meta-analytic model, chosen alpha level, power, relative risk reduction, the proportion of events in the control group, minimally relevant difference, variance, heterogeneity correction, effect measure, correction for zero events, graphical presentation, specific results, and how the trial sequential analysis affected GRADE); and (5) protocol (details for planning the trial sequential analysis). Lastly, the researchers were asked to subjectively evaluate the transparency of the trial sequential analysis on a scale of ‘very poor’ (crucial parameters missing, typically four or more), ‘poor’ (several important parameters missing, typically two or three), ‘good’ (few parameters missing, typically only one), and ‘excellent’ (all parameters transparently presented). Details are available in Supplemental Material C. When multiple outcomes had been analysed with trial sequential analysis, we extracted data for one dichotomous and one continuous outcome. For studies including more than one outcome, an algorithm was developed for choosing between the outcomes ensuring the highest level outcome, e.g. primary, or the outcome with the highest acquired information size was chosen (Supplemental Material A). If only dichotomous or continuous outcomes were presented, only data on one trial sequential analysis was extracted.

Two authors independently assessed the methodological quality of all included systematic reviews using the AMSTAR 2 [32] and reached consensus. Each of the 16 items were rated, and the overall confidence in the results of each systematic review was rated as ‘high’, ‘moderate’, ‘low’, or ‘critically low’ [34]. Any discrepancy was resolved through discussion. If an agreement was not reached, the issue was discussed at the weekly meeting and finally a third author (CG) would resolve the disagreement. As lack of a protocol can always be regarded as a critical flaw, studies that did not have a publicly available registration or protocol before starting data extraction (i.e. meta-analysis reports) were automatically evaluated as ‘critically low’ and were not further assessed with the AMSTAR 2 tool.

### Data analysis

Data were exported from REDCap and processed and analysed in R v. 4.2.1 (R Core Team, Vienna, Austria) by MHO in collaboration with CGR, JBM, and CG. Mistakes or errors are presented as n and percentage in tables. Continuous values are presented as median and interquartile range (IQR) or mean and standard deviation (SD). Interpretations were based on a qualitative evaluation of differences.

As a post hoc subgroup analysis, we used data from the included Cochrane systematic reviews to assess if a structured, organisational approach, like Cochrane’s, would enhance the conduct and transparency of trial sequential analysis.

## Results

### Description of studies

After removing duplicates, the initial literature search revealed 2,169 studies published from 1st of January 2018 to 31st of December 2021. After the title and abstract screening, 738 studies were read in full text. The agreement between the two reviewers (MHO and CGR) in this screening showed a kappa of 0.76. From these, 194/738 studies were excluded mainly due to not including at least two randomised clinical trials in the forest plot and/or trial sequential analysis (Supplemental Table 3) leaving 544 included studies (Supplemental Table 4). Of these 270/544 (50%) were systematic reviews (with registration and/or pre-published protocol) and 274/544 were meta-analysis reports (without registration or pre-published protocol) (Fig. 1). From the 544 included studies, we extracted 624 trial sequential analyses, of which 439/624 (70%) analysed dichotomous outcomes and 185/624 (30%) analysed continuous outcomes. These were equally distributed between systematic reviews and meta-analysis reports (Table 1). In 612/624 (98%) trial sequential analyses the Copenhagen Trial Unit’s Trial Sequential Analysis programme in Java was used [15–18].

The corresponding author had a Chinese affiliation in 199/544 (37%) of the studies, but all continents were represented (Supplemental Fig. 1A). The top five medical fields applying Trial Sequential Analysis were ‘internal medicine’, ‘anaesthesiology’, ‘surgery’, ‘eastern medicine’, and ‘dentistry’ (Supplemental Fig. 1B), and the most investigated intervention was ‘pharmacological’ (Supplemental Fig. 1C). The rate of published studies increased from 2018 to 2021 (Table 1). Most systematic reviews had a publicly available registration in PROSPERO (85%) while 44/544 (16%) had a published protocol in a scientific peer-reviewed journal, including 27 (5%) Cochrane reviews (Table 1).

In our AMSTAR 2 assessment, all 274 meta-analysis reports (studies without a protocol) were considered of critically low confidence. Twenty-seven (10%) of systematic reviews were evaluated at high, 18 (7%) at moderate, 35 (13%) at low, and 190 (70%) at critically low confidence (Table 1).

The overall agreement between reviewers was calculated on selected items in the data-extraction form and showed from moderate to almost perfect agreement (Supplemental Table 9).

### Assessments of trial sequential analysis of dichotomous outcomes

From the 439 dichotomous outcomes analysed with Trial Sequential Analysis, we extracted 218 from systematic reviews and 221 from meta-analysis reports (Table 2). The median number of randomised clinical trials included in the forest plots and the acquired information size did not differ for systematic reviews and meta-analysis reports.

Forty out of 439 (9%) Trial Sequential Analysis results could not be interpreted due to missing information. The DARIS was reported in 154 (35%) of the analyses, unadjusted required information size in 67 (15%), 198 (45%) had unclear reporting, and 19 (4%) did not report a required information size (Table 2).

#### Proportion in the control group

Proportion of events in the control group was not reported in 76/218 (35%) of systematic reviews and 105/221 (48%) of meta-analysis reports, whereas 59/439 (13%) described the method of determining the proportion of events in the control group without providing the actual rate. For systematic reviews 112/218 (52%) used the observed proportion from the forest plot and 90/221 (41%) from their meta-analysis (Table 2).

#### Relative risk reduction

The relative risk reduction was presented in 333/438 (76%) of the studies, of which 73/218 (34%) of the systematic reviews and 90/221 (41%) of the meta-analysis reports did not report the rationale for the chosen value. Approximately one in five studies used a relative risk reduction above 25%, every third used a relative risk reduction from 20 to 24.9%, and a fourth of the studies used a relative risk reduction of 20% or below (Table 2).

#### Alpha, power, and heterogeneity

**Fig. 2:**
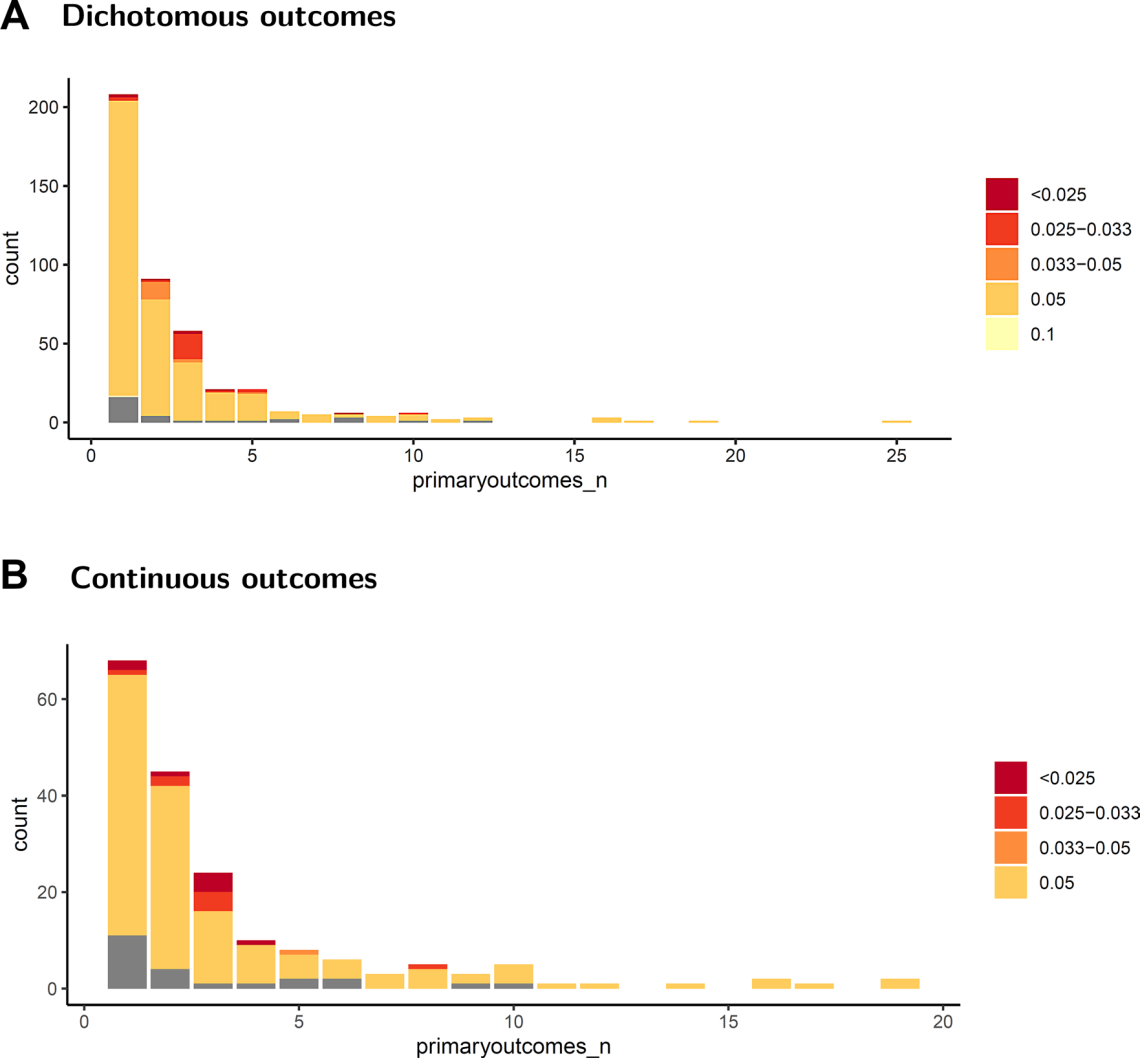
Comparison between the number of outcomes and alpha level. Figures are showing the number of outcomes in studies with dichotomous outcomes (**A**) and continuous outcomes (**B**). Studies not reporting the alpha level are depicted in grey. **A**: 176 of 409 (43%) dichotomous outcomes had a reported alpha level of 5% or higher and more than one primary outcome. **B**: 91 of 162 (56%) continuous outcomes had a reported alpha level of 5% or higher and more than one primary outcome

For the analyses, 164/218 (75%) systematic reviews and 200/221 (91%) meta-analysis reports used an alpha level of 0.05. For systematic reviews or meta-analyses, 31/439 (7%) did not report the alpha level (Table 2). A total of 177/409 (43%) reporting the alpha level were analysed alongside other primary outcomes without adjusting the alpha level (Fig. 2a). The power was set at 90% in 306/439 (70%) studies; however, 82/218 (38%) systematic reviews and 46/221 (21%) meta-analysis reports did not state the level of power used in the analyses. Authors used diversity (D^2^) for heterogeneity adjustment in 157/439 (36%) and inconsistency (I^2^) in 32/439 (7%). The method for heterogeneity correction used or intended to be used in the Trial Sequential Analysis was not described in 107/218 (49%) of systematic reviews and 125/221 (57%) of meta-analysis reports (Table 2).

#### Trial sequential analysis adjusted confidence intervals

We found that 86/439 (20%) of the analyses of dichotomous outcomes were presented with a Trial Sequential Analysis-adjusted confidence interval. The systematic reviews presented this in 60/218 (28%) and the meta-analysis reports in 26/221 (12%) (Table 2).

#### Information size

The median percentage acquired information size over the D^2^-, I^2^-, or non-adjusted required information size for the dichotomous outcomes was 60% (IQR 25–122%) for systematic reviews and 75% (IQR 38–134%) for meta-analysis reports. Fifty out of 439 studies (11%), 30/218 (14%) systematic reviews and 20/221 (9%) meta-analysis reports, failed to report the required or acquired information size or both (Table 2).

#### Graphical presentation

Dichotomous outcomes were presented graphically in 409/439 (93%) analyses (Table 2). The conventional 5% significance limit was not outlined in 48/439 (12%) analyses, and the Z-curve was incorrectly outlined in 97/439 (24%) analysis mainly due to selection of equal trial spacing. Overall, systematic reviews performed better than meta-analysis reports (Table 2).

#### Transparency of Trial Sequential Analysis

Forty-five of 218 (21%) systematic reviews had excellent transparency in reporting Trial Sequential Analysis parameters compared to 12/221 (5%) meta-analysis reports. Overall, 259/439 (59%) of the Trial Sequential Analyses of dichotomous outcomes were categorised as poor or very poor transparency due to the absence of parameters for interpretation (Table 2).

### Assessments of trial sequential analysis of continuous outcomes

A total of 185/624 (30%) Trial Sequential Analyses evaluated continuous outcomes with 94 (51%) from systematic reviews and 91 (49%) from meta-analysis reports. Nineteen out of 185 (10%) of the analyses used the standardised mean difference despite being incompatible with the Trial Sequential Analysis software. The median number of randomised clinical trials included in the forest plots and the acquired information size did not differ for systematic reviews and meta-analysis reports (Table 3).

Sixteen out of 185 (9%) Trial Sequential Analysis results could not be interpreted due to missing information. The DARIS was reported in 46 (25%) of the outcomes, unadjusted required information size in 27 (15%), 104 (56%) had unclear reporting, and 8 (4%) did not report a required information size (Table 3).

#### Minimally relevant difference and variance

The minimally relevant difference was not reported in 125/185 (68%) of analyses, with 54/94 (57%) in systematic reviews and 71/91 (78%) in meta-analysis reports. The variance (or standard deviation) was not reported in 144/185 (86%) Trial Sequential Analyses, with 65/94 (69%) in systematic reviews and 79/91 (87%) in meta-analysis reports (Table 3).

#### Alpha, power, and heterogeneity

A 0.05 alpha level was reported in 145/185 (78%) of the analyses, 23/185 (12%) did not report a specific alpha level, and 17/185 (9%) reported an alpha level lower than 0.05 (Table 3). Ninety-one (56%) of the continuous outcome measures were analysed alongside other primary outcomes without adjusting the alpha level (Fig. 2b). Power was set at 0.9 for all continuous outcomes that provided a definition, however, 63/185 (34%) of the analyses did not report power at all. D^2^ was used in 53/185 (29%) of the analyses and heterogeneity correction was not reported in 105/185 (57%), of which 14 (8%) did not find heterogeneity in their forest plot analysis (Table 3).

#### Trial sequential analysis-adjusted confidence intervals

We found that 30/185 (16%) of the analyses presented the Trial Sequential Analysis-adjusted confidence intervals, and this was more common in 19/94 (20%) systematic reviews than in the 11/91 (12%) meta-analysis reports (Table 3).

#### Information size

The median percentage acquired information size over the D^2^-, I^2^-, or non-adjusted required information size for the continuous outcomes was 100% (IQR 60–178%) for systematic reviews and 71% (IQR 36–137%) for the meta-analysis reports. Twenty-seven of 185 (15%) of studies failed to report the required or acquired information size or both (Table 3).

#### Graphical presentation

Continuous outcomes were presented graphically in 171/185 (92%) analyses (Table 3). The futility boundaries in 75/185 (41%) analyses and the conventional 5% significance limit in 15/185 (8%) analyses were not outlined. The Z-curve was incorrectly outlined in 38/185 (21%) analyses, mainly due to selection of equal trial spacing. Overall, systematic reviews performed better than meta-analysis reports (Table 3).

#### Transparency of trial sequential analysis

We rated transparency as excellent for the Trial Sequential Analysis in 10/94 (11%) systematic reviews and 3/91 (3%) meta-analysis reports of continuous outcomes. Transparency was poor or very poor in 150/185 (81%) analyses (Table 3).

### Impact of trial sequential analysis on assessment of imprecision and rating the certainty of evidence

Certainty of evidence was assessed using GRADE in 301/544 (55%) studies. This was done in 194/218 (72%) systematic reviews and 107/221 (39%) meta-analysis reports. Downgrading of imprecision was explicitly affected by the Trial Sequential Analyses in 88/301 (29%) of outcomes assessed with GRADE. This approach was more used in the systematic reviews (Table 1).

### Subgroup comparing Cochrane reviews to non-Cochrane reviews

The comparison of Cochrane systematic reviews to non-Cochrane systematic reviews can be found in the supplemental material (Supplemental material B, Supplemental Tables 6, 7 and 8). In general, the Cochrane systematic reviews performed better in protocolising and reporting parameters for Trial Sequential Analysis and, hence, were assessed with higher transparency.

## Discussion

In this study, we investigated the most common mistakes or errors when using trial sequential analysis to control type I and type II errors in systematic reviews and in meta-analysis reports. For trial sequential analysis, the most prevalent choice was the Trial Sequential Analysis programme from the Copenhagen Trial Unit. The most common and serious mistake or error was the lack of a protocol publicly available before starting data extraction and outlining the methods for conducting the Trial Sequential Analysis. Few dichotomous outcomes (13%) had excellent transparency in reporting parameters, such as the proportion of events in the control group, relative risk reduction, value for alpha level, power (or beta), heterogeneity, or adjustment for possible multiplicity issues. Only 7% of the continuous outcomes transparently reported the minimally relevant difference, variance or standard deviation, value for alpha level, power (or beta), heterogeneity, and adjustment for possible multiplicity issues. Furthermore, half of all analyses did not include the required information size. Comparing systematic reviews to meta-analysis reports emphasises the superior quality of the former in some cases, but also underscores the weaknesses and waste of both, with the wide possibilities for amendments [21, 35–38]. In general, the Cochrane systematic reviews performed better in reporting parameters relevant to Trial Sequential Analysis for both dichotomous and continuous outcomes. This is in line with previous studies [39–42].

There are limitations to our research-on-research study. Firstly, including only studies in English is a potential limitation, however, most systematic reviews with high clinical impact tend to be published in internationally recognised journals in English. As stated in our protocol, we expected approximately 500 studies to be included during the chosen period which showed to be sufficient when only including articles in English. We have little reason to believe that studies published in other languages would improve the conclusion of our study.

Secondly, we only investigated one method for controlling type I and type II errors and the results should be seen in the light of this frequentist approach. Other methods exist and could potentially have better reporting. However, the frequentist approach is by far the most common in medical science. Thirdly, we chose to only extract data on only one dichotomous and one continuous outcome, if possible. The rationale was that mistakes would be generic within one article, and we therefore decided to prioritise the primary outcomes as described in our protocol [26]. Potential additional errors or mistakes could have been made on secondary outcomes, yet this would be unlikely to change the conclusion of our study.

Fourth, as we could not anticipate the exact mistakes or errors that we would find, we could not define the complete data extraction form in advance. Nevertheless, the proportion of errors or mistakes we found were generic in both systematic reviews and meta-analysis reports indicating a substantial and relevant problem. Additionally, it is challenging for us to distinguish between errors and mistakes in our findings since we lack the ability to assess the authors’ expertise in conducting systematic reviews as errors in this context would refer to methodological flaws or negligence and mistakes would refer to a misguided action that was unintended. Hence, we have generally referred to these discrepancies as mistakes or errors without specific categorisation.

Lastly, the AMSTAR 2 assessments revealed low or critically low confidence in most of our included studies, which should raise concerns. Nevertheless, others have found similar results when assessing systematic review methodology [43]. Here, the methodological quality has been reported within several medical fields with critically low or low confidence in 85% of systematic reviews [43]. Our findings appear to reflect the overall quality of systematic reviews in general. However, it is important to note that our results specifically pertain to studies utilising Trial Sequential Analysis and do not encompass the reporting and protocolisation of other types of systematic reviews which have been previously examined [44, 45]. Also, studies without a protocol were defined as critically low based on the AMSTAR guideline.

There was some variance in the agreement between different items in our data-extraction form. This may reflect the complexity of the questions asked by the group but could also reflect the lack of transparency in the published articles.

We found that more than half of the studies did not have a registration or a protocol before conducting the review, and more than half of the protocols did not plan to conduct a Trial Sequential Analysis. Even though the PRISMA-P group published a guideline in 2015 on how to report items in protocols for systematic reviews [46], our cohort of studies implies a lack of quality in reporting and protocolising systematic reviews, as previously reported [44, 47]. The PRISMA-P guidelines emphasise the need for transparency, accuracy, and completeness of reporting in protocols. These requirements also apply to the published protocol before conduct of the review and the Trial Sequential Analysis [11]. As retrospectively performed sequential analyses are prone to sequential decision bias it underlines the importance of defining these variables in a pre-registered protocol.

All the parameters required for Trial Sequential Analysis have important functions in estimating the required information size and should be reported to facilitate critical appraisal, replication, and accurate interpretation [16, 17]. We showed that only 11% of systematic reviews or meta-analysis reports describe the Trial Sequential Analysis parameters with high transparency (excellent), while 66% were categorised as poor or very poor. This lack of transparency diminishes the replicability, trustworthiness, and interpretability of the results.

The alpha level of published Trial Sequential Analyses was often either not appropriately adjusted for multiplicity or not reported. In frequentist randomised clinical trials it is required to decide on a type I or type II error proportion before conducting the trial, to ensure reasonable accuracy of the trial result [48]. Traditionally a 5% alpha level has been used in systematic reviews [1]. Lowering the alpha level should be considered to avoid multiplicity issues [49]. A large proportion of the studies had only one primary outcome, and presented a valid 0.05 alpha level, but almost half of the studies had more than one primary outcome and did not adjust the alpha level (Fig. 2). Furthermore, 9% of reviews did not report an alpha level for the Trial Sequential Analysis at all, without which the analysis is impossible to interpret. Although this may not seem like a concerningly large number, it is a fundamental part of frequentist research and should be reported [9, 50].

Likewise, the power is equally important to avoid false conclusions that an intervention has no effect. We found that 31% of the studies did not report on the level of power used in the Trial Sequential Analysis, and one study deliberately chose a power lower than 0.8. The study by Turner and colleagues investigated the power in meta-analyses from Cochrane reviews and found that in 1,107 meta-analyses, the majority did not have > 50% power to detect a relative risk reduction of 30% [10]. These data, alongside the data from the current study, indicate a need for greater awareness and concern for reporting power in systematic reviews. We suggest this to be an important focus during the review process, driven by the reviewers.

Statistical heterogeneity is an inherent property of a meta-analysis due to the pooling of data from different trials and is traditionally quantified as I^2^ in the pooled meta-analytic data [1]. For Trial Sequential Analysis of random-effects analyses, it is recommended to express heterogeneity as D^2^ when calculating the required information size (meta-analytic sample size) [19]. We found that over half of the studies failed to report if they adjusted for heterogeneity or failed to report the method for heterogeneity adjustment in the Trial Sequential Analysis. This considerably impacts the estimated required information and, consequently, future clinical trials [51].

When analysing dichotomous outcomes using Trial Sequential Analysis, it is essential to define the anticipated relative risk reduction (or increase) and the proportion of events in the control group. Our findings revealed that 24% of the studies did not report the assumed relative risk reduction for the intervention and 41% did not provide the value for the proportion of events in the control group. These values should be carefully selected during protocol development and supported with strong justifications [52]. Ideally, they should be based on low-risk-of-bias systematic reviews or randomised clinical trials. However, our data showed that only 3% and 6% of the proportion of events in the control group and the relative risk reduction, respectively, were derived from previously published studies. It is problematic to rely solely on the relative risk reduction from the conducted meta-analysis, as this leads to reinforcement. Previous randomised clinical trials indicate that intervention effects rarely exceed a relative risk reduction of 20% or more [53], except for vaccine, antibiotic, and surgical trials [54]. Consequently, it appears that many researchers tend to overestimate the intervention’s effect [55].

We found even larger issues with the Trial Sequential Analysis on continuous outcomes. As highlighted in several publications, the minimally relevant difference can be a challenge to estimate [56, 57] but still important to consider [58–60]. Only 32% of the reviews explicitly defined the minimally relevant difference in their reports, and only 22% defined the variance (or standard deviation). This makes the Trial Sequential Analysis completely un-interpretable as the ratio between the minimally relevant difference and variance has a large influence on the DARIS.

The graphical presentations of the Trial Sequential Analyses were frequently represented for all types of outcomes. When performed correctly, they provide a useful illustration of the relationship between the acquired and required information size. However, common graphical presentation mistakes could misguide readers in their conclusion about the meta-analytic results. First, a common mistake occurs when using equal trial spacing to present the Z-curve. This means the trials are evenly spaced irrespective of their sample sizes. As a result, the visually stretched Z-curve creates an illusion that the required information size has almost been achieved. Secondly, the use of equal trial spacing has an impact on the area of futility. When trials are equally spaced, the region of futility is compressed and pushed closer to the line representing the required information size. In certain instances, it may even disappear altogether. Although omitting the boundary of futility may not be considered a mistake or error and may have been done intentionally, reviewers should be aware of the loss of information when doing so, leading to further possible research waste. This represented the second most common graphical issue. Future software or software updates should have incorporated warning for researchers prone to use such graphs. Graphical presentations can guide or mislead readers and reviewers need to focus on this presentation to create a clear message [61].

Trial Sequential Analysis is a tool for controlling type I and type II errors in trials and meta-analyses of such trials by estimating the DARIS and using monitoring boundaries. Hence, it is important to highlight that a significant proportion (48%) of the studies failed to mention how the required information size was determined, including whether it was calculated using D^2^ or I^2^ adjustment methods. Additionally, 4% of the studies did not provide any information regarding the required information size. The importance of calculating an optimal information size for meta-analytic data has been highlighted by the GRADE recommendations [58, 60]. If used properly, Trial Sequential Analysis can be used to evaluate imprecision in GRADE without use of naïve 95% CI, and if calculated the Trial Sequential Analysis-adjusted confidence interval can be used to support this evaluation [26, 62]. Alternatively, one can follow the latest GRADE recommendation of a minimally contextualised approach where downgrading for imprecision is primarily based on CIs and minimally important differences. Here a Trial Sequential Analysis-adjusted confidence interval can aid the imprecision assessment [58].

The above discussion stresses the importance of a thorough, transparent, accurate, and complete protocol for the systematic review and reporting of the results. Failure to address this issue leaves room for researchers to manipulate their findings intentionally or unintentionally by altering crucial parameters to achieve desired results. For example, one may amplify the relative risk reduction and/or alter the alpha level and power of the Trial Sequential Analysis to reduce the DARIS and enable the Z-curve to cross the Trial Sequential Analysis adjusted boundaries. It is paramount that such practice should be abstained as systematic reviews are frequently used to inform policies and clinical guidelines. In fact, we support a stop to the publication of meta-analysis reports without a proper pre-published or registered protocol [2, 21, 35]. In accordance, we want to highlight that the quality of most PROSPERO registrations is far from having a high enough quality to resemble a full systematic review protocol and we did not come across systematic reviews uploading or referring full protocols through PROSPERO. One may therefore question the validity of our decision to call meta-analyses with a PROSPERO registration for systematic reviews. We considered the PROSPERO registration as at least attempts to formalise the review process before embarking on data extraction and analyses. Future studies ought to assess the quality of PROSPERO registrations and the extent they can function as stand in for a full systematic review protocol.

Our study has shown that researchers using the Trial Sequential Analysis have challenges when preparing and reporting their work. It is, therefore, important that future guidelines and software are created to assist researchers. Thus, a new beta version of the Trial Sequential Analysis software is currently being developed in R – *RTSA* [62]. In the future, we intend to make RTSA with an interface that guides researchers in their decision making. Furthermore, the new version will be able to estimate the required number of trials in addition to calculating the required information size as this is important to achieve the wanted level of power in a random-effects meta-analysis. In RTSA, it is also possible to conduct the Trial Sequential Analysis as retrospective (comparable to the present Java version) or prospective. As stated, “If meta-analysis is the gold standard of evidence, then the prospective meta-analysis must be the diamond standard of evidence. One should aim for being as close to a prospective meta-analysis as possible” [63].

In the present article, we can only provide a broad overview of the major errors or mistakes. In future publications, we will dive into several of the identified major problems and suggest amendments. To prevent research waste future studies of research methods should be prioritised.

## Conclusions

Studies defined as systematic reviews and meta-analysis reports increasingly use Trial Sequential Analyses to control type I and type II errors. Systematic reviews and meta-analysis reports lack transparency when reporting Trial Sequential Analysis specific parameters, partly due to missing or poorly conducted protocols. This calls for more precise guidelines and readers of such reviews are encouraged to critically appraise these studies.

**Table 1:** Study characteristics

	Systematic review	Meta-analysis reports	Overall
**Publications**	270	274	544
**Publication year**			
2018	52 (19.3%)	65 (23.7%)	117 (21.5%)
2019	55 (20.4%)	60 (21.9%)	115 (21.1%)
2020	67 (24.8%)	79 (28.8%)	146 (26.8%)
2021	96 (35.6%)	70 (25.5%)	166 (30.5%)
**AMSTAR 2 evaluation**			
High	27 (10.0%)	0	27 (5.0%)
Moderate	18 (6.7%)	0	18 (3.3%)
Low	35 (13.0%)	0	35 (6.4%)
Critically low	190 (70.4%)	274 (100%)	464 (85.3%)
**Protocolised before data extraction**	270 (100%)	0	270 (49.6%)
**Protocol published in** ^a^			
Scientific journal	44 (16.3%)	0	44 (8.1%)
PROSPERO	230 (85.2%)	0	230 (42.3%)
Webpage	8 (3.0%)	0	8 (1.5%)
**Cochrane reviews**	27 (10.0%)	0	27 (5.0%)
**Planned details on TSA in protocol**	134 (49.6%)	0	134 (24.6%)
Planned dichotomous outcomes	102 (37.8%)	0	102 (18.8%)
Planned continuous outcomes	67 (24.8%)	0	67 (12.3%)
**Number of TSA extracted**	310 (49.8%)	313 (50.2%)	623 (100%)
Dichotomous outcomes	218 (69.9%)	221 (70.8%)	439 (70.0%)
Continuous outcomes	94 (30.1%)	91 (29.2%)	185 (30.0%)
**Used GRADE**	194 (71.9%)	107 (39.1%)	301 (55.3%)
**TSA affected GRADE evaluation**	66 (24.4%)	22 (8.0%)	88 (16.2%)

AMSTAR: Assessing the Methodological quality of Systematic Reviews; GRADE: Grading of Recommendations Assessment, Development and Evaluation; Meta-analysis reports: a non-systematic approach which lacks a pre-published protocol at the time of data extraction; Systematic review: a detailed, organised, and transparent method of gathering, appraising and synthesising data to answer a well-defined question, including a pre-published protocol before starting data extraction; TSA: Trial Sequential Analysis

a. Protocols could be published in both PROSPERO and scientific journals

**Table 2:** Details on Trial Sequential Analyses performed on dichotomous outcomes

	Systematic review	Meta-analysis reports	Overall
**Number of TSA extracted**	218	221	439
**Extracted TSA was done on**			
Primary outcome	200 (91.7%)	290 (94.6%)	409 (93.2%)
Secondary outcome	18 (8.3%)	12 (5.4%)	30 (6.8%)
Exploratory outcome	0 (0%)	0 (0%)	0 (0%)
**Model used**			
Random-effects model	141 (64.7%)	130 (58.8%)	271 (61.7%)
Fixed-effect model	47 (21.6%)	63 (28.5%)	110 (25.1%)
Fixed- and random-effects models	29 (13.3%)	25 (11.3%)	54 (12.3%)
Other^a^	0	1 (0.5%)	1 (0.2%)
Not mentioned	1 (0.5%)	2 (0.9%)	3 (0.7%)
**Dichotomous effect estimates used**			
RR	174 (79.8%)	164 (74.2%)	338 (77.0%)
OR	39 (17.9%)	45 (20.4%)	84 (19.1%)
Peto OR	2 (0.9%)	1 (0.5%)	3 (0.7%)
RD	1 (0.5%)	1 (0.5%)	2 (0.5%)
Other^b^	2 (0.9%)	10 (4.5%)	12 (2.7%)
**Number of trials included in TSA** ^**c**^			
Mean (SD)	11.0 (9.1)	10.8 (8.7)	10.8 (8.9)
Median (IQR)	8.0 (5.0 to 14.0)	9.0 (5.0 to 13.0)	9.0 (5.0 to 13.0)
**Acquired information size**			
Mean (SD)	13,100 (87,400)	5,540 (12,100)	9,220 (61,900)
Median (IQR)	1,490 [680 to 4,590]	1,700 [868 to 4,280]	1,650 [771 to 4,350]
No extractable information	16 (7.3%)	11 (5.0%)	27 (6.2%)
**TSA results**			
Beneficial	67 (30.7%)	81 (36.7%)	148 (33.7%)
Harmful	12 (5.5%)	7 (3.2%)	19 (4.3%)
Futile	34 (15.6%)	40 (18.1%)	74 (16.9%)
Insignificant	76 (34.7%)	82 (37.1%)	158 (36.0%)
Uninterpretable	29 (13.3%)	11 (5.0%)	40 (9.1%)
**Was the Pc presented?**			
Yes, the value was reported	110 (50.5%)	89 (40.3%)	199 (45.3%)
Yes, but no value was reported	32 (14.7%)	27 (12.2%)	59 (13.4%)
No	76 (34.9%)	105 (47.5%)	181 (41.2%)
**How was Pc selected?**			
Previously published data^d^	7 (3.2%)	7 (3.2%)	14 (3.2%)
From current forest plot	112 (51.4%)	90 (40.7%)	202 (46.1%)
Not mentioned	14 (6.4%)	8 (3.6%)	22 (5.0%)
Other^e^	0	1 (0.9%)	1 (0.2%)
Unclear	9 (4.1%)	10 (4.5%)	19 (4.3%)
**Was the RRR presented?**			
Yes	169 (77.5%)	164 (74.2%)	333 (75.9%)
No	49 (22.5%)	57 (25.8%)	106 (24.1%)
**How was RRR selected?**			
Previously published data^d^	14 (6.4%)	17 (7.7%)	31 (7.1%)
From the current forest plot	36 (16.5%)	28 (12.7%)	64 (14.6%)
From author’s clinical experience	28 (12.8%)	19 (8.6%)	47 (10.7%)
From other sources	35 (16.1%)	21 (9.5%)	56 (12.8%)
Not mentioned	73 (33.5%)	90 (40.7%)	163 (37.1%)
**Level of RRR used in outcomes**			
0 to 4.9%	1 (0.5%)	3 (1.4%)	4 (0.9%)
5 to 9.9%	6 (2.8%)	4 (1.8%)	10 (2.3%)
10 to 14.9%	30 (13.8%)	21 (9.5%)	51 (11.6%)
15 to 19.9%	22 (10.1%)	22 (10.0%)	44 (10.0%)
20 to 24.9%	62 (28.4%)	63 (28.5%)	125 (28.5%)
25 to 29.9%	11 (5.0%)	17 (7.7%)	28 (6.4%)
30 to 39.9%	14 (6.4%)	13 (5.9%)	27 (6.2%)
40 to 49.9%	5 (2.3%)	1 (0.5%)	6 (1.4%)
> 50%	12 (5.5%)	19 (8.6%)	31 (7.1%)
**Alpha level chosen**			
< 0.025	4 (1.8%)	3 (1.4%)	7 (1.6%)
0.025 to 0.033	21 (9.6%)	2 (0.9%)	23 (5.2%)
0.033 to 0.05	13 (6.0%)	1 (0.5%)	14 (3.2%)
0.05	164 (75.2%)	200 (90.5%)	364 (82.9%)
0.1	0 (0%)	1 (0.5%)	1 (0.2%)
Not reported	16 (7.3%)	14 (6.3%)	30 (6.8%)
**Power chosen (** ***1-beta*** **)**			
< 0.8	1 (0.5%)	0%	1 (0.2%)
0.85	0	1 (0.4%)	1 (0.2%)
0.9	134 (61.5%)	172 (77.8%)	306 (69.7%)
> 0.9	1 (0.5%)	2 (0.9%)	3 (0.7%)
Not reported	82 (37.6%)	46 (20.8%)	128 (29.2%)
**Heterogeneity used in TSA**			
D^2^	92 (42.2%)	65 (29.4%)	157 (35.8%)
I^2^	11 (5.0%)	21 (9.5%)	32 (7.3%)
No heterogeneity corrected in TSA	8 (3.7%)	10 (4.5%)	18 (4.1%)
Not described / not clear	71 (32.6%)	81 (36.7%)	152 (34.6%)
Not described, but I^2^ = 0%	36 (16.5%)	44 (19.9%)	80 (18.2%)
**How was the RIS presented?**			
DARIS	82 (37.6%)	73 (33.0%)	155 (35.3%)
RIS (unadjusted)	32 (14.7%)	35 (15.8%)	67 (15.3%)
RIS not presented	14 (6.4%)	5 (2.3%)	19 (4.3%)
Unclear^f^	90 (41.3%)	108 (48.9%)	198 (45.1%)
**TSA-adjusted CI included**			
Yes	46 (21.1%)	15 (6.8%)	61 (13.9%)
Yes, but mislabelled	14 (6.4%)	11 (5.0%)	25 (5.7%)
No	151 (69.3%)	194 (87.8%)	345 (78.6%)
Not reported	7 (3.2%)	1 (0.5%)	8 (1.8%)
**Percentage AIS over RIS**			
Mean (SD)	122 (311)	135 (212)	129 (264)
Median (IQR)	60.4 (24.9 to 122.0)	75.4 (37.7 to 134.0)	68.7 (30.0 to 129.0)
Not reported^g^	30 (13.8%)	20 (9.0%)	50 (11.4%)
**Graphical presentation**			
TSA presented as graph	195 (89.4%)	214 (96.8%)	409 (93.2%)
Conventional 5% limit not outlined	22 (11.3%)	26 (12.1%)	48 (11.7%)
Boundary of benefit not outlined	10 (4.6%)	8 (3.6%)	18 (4.1%)
Boundary of harm not outlined	11 (5.1%)	13 (5.9%)	24 (5.5%)
Boundary of futility not outlined	62 (31.8%)	89 (41.6%)	151 (36.9%)
Required information size not outlined	13 (6.0%)	8 (3.6%)	21 (4.8%)
Z-curve not correctly outlined	38 (19.5%)	59 (27.6%)	97 (23.7%)
**Transparency**			
Excellent	45 (20.6%)	12 (5.4%)	57 (13.0%)
Good	58 (26.6%)	65 (29.4%)	123 (28.0%)
Poor	48 (22.0%)	76 (34.4%)	124 (28.2%)
Very poor	67 (30.7%)	68 (30.8%)	135 (30.8%)

AIS: acquired information size; D^2^: diversity; DARIS: diversity-adjusted required information size; I^2^: inconsistency; Meta-analysis reports: a non-systematic approach which lacks a pre-published protocol at the time of data extraction; OR: odds ratio; Pc: proportion of events in the control group; Peto OR: Peto odds ratio; RD: risk difference; RIS: required information size; RR: risk ratio; RRR: relative risk reduction; Systematic review: a detailed, organised, and transparent method of gathering, appraising and synthesising data to answer a well-defined question, including a pre-published protocol before starting data extraction; TSA: Trial Sequential Analysis; TSA adjusted CI: Trial Sequential Analysis-adjusted confidence interval

a. One study used empirical Bayes binary random effect

b. Preferred alternative method was hazard ratio

c. Missing value in 4 systematic reviews

d. Previous systematic reviews, randomised clinical trials or observational studies

e. One study hypothesised the value for proportion of events in the control group

f. Unclear if RIS was adjusted or unadjusted

g. Missing either AIS, RIS, or both

**Table 3:** Details on Trial Sequential Analyses performed on continuous outcomes

	Systematic review	Meta-analysis reports	Overall
**Number of TSA extracted**	94	91	185
**Extracted TSA was done on**			
Primary outcome	81 (86.2%)	78 (85.7%)	159 (85.9%)
Secondary outcome	12 (12.8%)	13 (14.3%)	25 (13.5%)
Exploratory outcome	1 (1.1%)	0 (0%)	1 (0.5%)
**Model used**			
Random-effects model	83 (88.3%)	73 (80.2%)	156 (84.3%)
Fixed-effect model	6 (6.4%)	13 (14.3%)	19 (10.3%)
Fixed- and random-effects models	5 (5.3%)	5 (5.5%)	10 (5.4%)
**Continuous effect estimates used**			
Mean difference	84 (89.4%)	80 (87.9%)	164 (88.6%)
Standardised mean difference	9 (9.6%)	10 (11.0%)	19 (10.3%)
Other^a^	1 (1.1%)	1 (1.1%)	2 (1.1%)
**Number of trials included in TSA**			
Mean (SD)	10.8 (9.8)	9.7 (8.4)	10.3 (9.1)
Median (IQR)	8.0 (5.0 to 13.0)	7.0 (5.0 to 11.5)	8.0 (5.0 to 12.3)
**Acquired information size**			
Mean (SD)	1,090 (1,270)	1,090 (1,410)	1,090 (1,330)
Median (IQR)	690 (398 to 1,260)	607 (354 to 1,100)	667 (356 to 1,170)
No extractable information	6 (6.4%)	11 (12.1%)	17 (9.2%)
**TSA results**			
Beneficial	50 (53.2%)	47 (51.6%)	97 (52.4%)
Harmful	7 (7.4%)	5 (5.5%)	12 (6.5%)
Futile	11 (11.7%)	4 (4.4%)	15 (8.1%)
Insignificant	16 (17.0%)	29 (31.9%)	45 (24.3%)
Uninterpretable	10 (10.6%)	6 (6.6%)	16 (8.6%)
**Was the minimally relevant difference presented**			
Yes	40 (42.6%)	20 (22.0%)	60 (32.4%)
No	54 (57.4%)	71 (78.0%)	125 (67.6%)
**Was variance presented**			
Yes	29 (30.9%)	12 (13.2%)	41 (22.2%)
No	65 (69.1%)	79 (86.8%)	144 (77.8%)
**Alpha level chosen**			
< 0.025	7 (7.4%)	1 (1.1%)	8 (4.3%)
0.025 to 0.033	5 (5.3%)	3 (3.3%)	8 (4.3%)
0.033 to 0.05	1 (1.1%)	0 (0%)	1 (0.5%)
0.05	72 (76.6%)	73 (80.2%)	145 (78.4%)
Not reported	9 (9.6%)	14 (15.4%)	23 (12.4%)
**Power chosen (** ***1-beta*** **)**			
0.9^b^	60 (63.8%)	62 (68.1%)	122 (65.9%)
Not reported	34 (36.2%)	29 (31.9%)	63 (34.1%)
**Heterogeneity**			
D^2^	33 (35.1%)	20 (22.0%)	53 (28.6%)
I^2^	5 (5.3%)	6 (6.6%)	11 (5.9%)
No heterogeneity corrected in TSA	7 (7.4%)	9 (9.9%)	16 (8.6%)
Not described / not clear	44 (46.8%)	47 (51.6%)	91 (49.2%)
Not described, but I^2^ = 0%	5 (5.3%)	9 (9.9%)	14 (7.6%)
**How was the RIS presented?**			
DARIS	26 (27.7%)	20 (22.0%)	46 (24.9%)
RIS (unadjusted)	16 (17.0%)	11 (12.1%)	27 (14.6%)
RIS not presented	4 (4.3%)	4 (4.4%)	8 (4.3%)
Unclear^c^	48 (51.1%)	56 (61.5%)	104 (56.2%)
**TSA-adjusted CI included**			
Yes	15 (16.0%)	9 (9.9%)	24 (13.0%)
Yes, but mislabelled	4 (4.3%)	2 (2.2%)	6 (3.2%)
No	75 (79.8%)	80 (87.9%)	155 (83.8%)
**Percentage AIS over RIS**			
Mean (SD)	168 (216)	138 (267)	154 (241)
Median (IQR)	100 (60.2 to 178.0)	71.3 (36.1 to 137.0)	90.7 (46.8 to 162.0)
Missing	10 (10.6%)	17 (18.7%)	27 (14.6%)
**Graphical presentation**			
TSA presented as graph	85 (90.4%)	86 (94.5%)	171 (92.4%)
Conventional 5% limit not outlined	7 (7.5%)	8 (8.8%)	15 (8.1%)
Boundary of benefit not outlined	1 (1.1%)	5 (5.5%)	6 (3.2%)
Boundary of harm not outlined	2 (2.1%)	6 (6.6%)	8 (4.3%)
Boundary of futility not outlined	26 (27.7%)	49 (53.9%)	75 (40.5%)
Required information size not outlined	1 (1.1%)	5 (5.5%)	6 (3.2%)
Z-curve not correctly outlined	13 (13.9%)	25 (27.5%)	38 (20.6%)
**Transparency**			
Excellent	10 (10.6%)	3 (3.3%)	13 (7.0%)
Good	16 (17.0%)	6 (6.6%)	22 (11.9%)
Poor	24 (25.5%)	26 (28.6%)	50 (27.0%)
Very poor	44 (46.8%)	56 (61.5%)	100 (54.1%)

AIS: acquired information size; D2: diversity; DARIS: diversity-adjusted required information size; I2: inconsistency; Meta-analysis reports: a non-systematic approach which lacks a pre-published protocol at the time of data extraction; RIS: required information size; Systematic review: a detailed, organised, and transparent method of gathering, appraising and synthesising data to answer a well-defined question, including a registration and/or a pre-published protocol before starting data extraction; TSA: Trial Sequential Analysis; TSA adjusted CI: Trial Sequential Analysis adjusted confidence interval

a. Hedges’ g

b. Studies only reported power of 0.9

c. Unclear if RIS was adjusted or unadjusted

## Electronic supplementary material

Below is the link to the electronic supplementary material.

Supplementary Material 1

## Data Availability

All data will be made available on zenodo.org after publication of our results.

## Abbreviations

AMSTAR 2: Assessing the methodological quality of systematic reviews 2
D^2^: Diversity
DARIS: Diversity-adjusted required information size
GRADE: Grading of Recommendations Assessment, Development and Evaluation
Pc: Proportion of participants with the outcome in the control group
RRR: Relative risk reduction
I^2^: Inconsistency
SD: Standard deviation
IQR: Interquartile range
