# Supplementary material for: Major mistakes or errors in the use of trial sequential analysis in systematic reviews or meta-analyses – the METSA systematic review
Source: BMC Med Res Methodol. 2024 Sep 9;24:196. doi: 10.1186/s12874-024-02318-y (PMC11382479; doi:10.1186/s12874-024-02318-y)
Supplement: Supplementary file 1 — Supplementary Material 1 [file 12874_2024_2318_MOESM1_ESM.pdf]

Major Mistakes or Errors in the use of Trial Sequential Analysis in Systematic Reviews or Meta-analyses – the METSA Systematic Review

Supplemental material

Christian Gunge Riberholt (0000-0002-6170-186)[1,2,3], Markus Harboe Olsen (0000-0003-0981-0723)[1,3], Joachim Birch Milan (0000-0001-7093-5432)[1], Sigurlaug Hanna Hafliðadóttir (0000-0002-7570-565X)[4], Jeppe Houmann Svanholm (0000-0002-1131-2216)[5], Elisabeth Buck Pedersen (0009-0000-2333-7405)[2], Charles Cin Han Lew (0000-0001-6410-3859)[6,7], Mark Aninakwah Asante (0009-0002-8034-4139)[1], Johanne Marie Pereira Ribeiro[8], Vibeke Wagner (0000-0002-1712-1648)[2,9,], Buddheera W.M.B Kumburegama[1], Zheng-Yii Lee (0000-0003-4505-7476)[10,11], Julie Perrine Schaug (0000-0002-1283-4024)[8], Christina Madsen (0009-0002-9764-7144)[8], Christian Gluud (0000-0002-8861-0799)[1,12]

1. Copenhagen Trial Unit, Centre for Clinical Intervention Research, The Capital Region, Copenhagen University Hospital – Rigshospitalet, Blegdamsvej 9, 2100 Copenhagen, Denmark. christian.riberholt@regionh.dk, markus.harboe.olsen@regionh.dk, joachimbirchmilan@hotmail.com, mark.asante@ctu.dk and christian.gluud@ctu.dk
2. Department of Brain and Spinal Cord Injury, Neuroscience Centre, Copenhagen University Hospital – Rigshospitalet, Valdemarsvej 23, 2600 Glostrup, Denmark. vibeke.wagner@regionh.dk, elisabeth2830@gmail.com
3. Department of Neuroanaesthesiology, Neuroscience Centre, Copenhagen University Hospital – Rigshospitalet, Blegdamsvej 9, 2100, Copenhagen, Denmark.
4. Bjarg Rehabilitation Center, Bugðusíðu 1, 603 Akureyri, Iceland. sigurlaughanna@gmail.com
5. Department of Gastrointestinal Surgery, Aalborg University Hospital South, Hobrovej 18-22, 9000 Aalborg, Denmark. jepardy.svanholm@gmail.com.
6. Department of Dietetics and Nutrition, Ng Teng Fong General Hospital, Singapore
7. Faculty of Health and Social Sciences, Singapore Institute of Technology, Singapore
8. Psychiatric Research Unit, Psychiatry Region Zealand, Region Zealand, Fælledvej 6, 4200 Slagelse, Denmark jscha@regionsjaelland.dk, chmads@regionsjaelland.dk
9. Department of Clinical Medicine, Faculty of Health and Medical Sciences, University of Copenhagen, Denmark
10. Department of Anaesthesiology, Faculty of Medicine, University of Malaya, Malaysia
11. Department of Cardiac Anesthesiology & Intensive Care Medicine, Charité Berlin, Germany
12. Department of Regional Health Research, The Faculty of Health Sciences, University of Southern Denmark, Odense, Denmark

Contents

Supplemental Material A. Amendments to the protocol 3
Supplemental Figure 1. Distribution of studies across the world, on medical fields and intervention types .. 5
Supplemental Table 1. PRISMA and PRISMA-S Checklist 8
Supplemental Table 2. Search strategy 12
Supplemental Table 3. Studies excluded in full text with reason 13
Supplemental Table 4. Included studies 27
Supplemental Table 5. Data on study type and overall proportions 80
Supplemental Material B. Subgroup comparing Cochrane reviews to non-Cochrane reviews 82
Supplemental Table 6. Study characteristics for Cochrane reviews and non-Cochrane reviews 84
Supplemental Table 7. Dichotomous outcomes for Cochrane reviews and non-Cochrane reviews 85
Supplemental Table 8. Continuous outcomes for Cochrane reviews and non-Cochrane reviews 88
Supplemental Table 9. Agreement between two reviewers on selected extraction variables 90
Supplemental Material C. REDCap instruments used for extraction 91

Supplemental Material A. Amendments to the protocol

We have added a decision algorithm to select the trial sequential analyses of interest when more than two are performed in a systematic review. For each type of outcome (dichotomous and continuous), primary outcomes were prioritised over secondary and others. If more than one primary outcome were defined, the outcome with the largest acquired information size (largest number of participants and outcomes) was chosen. If this was equal, the outcome with the highest percentage of acquired information size over the required information size was chosen. If this was equal, we chose the outcome that was first presented in the study. This was decided in the METSA group on the 5th of January 2022 after data was extracted from the first 12 studies.

Figure. Algorithm from the data extraction form in REDCap

REMINDER! When multiple outcomes have been analyzed with TSA, repeat the TSA instrument and extract data from one dichotomous and one continuous outcome measure if applicable.

1. **Dichotomous outcomes**
   a - Identify TSAs performed on dichotomous outcome measures (if NA, skip and go to 2a)
   b - if a>1, identify TSAs from highest rank (primary>secondary>explorative>subgroup)
   c - if b>1, identify TSA with highest acquired information size (AIS)
   d - if c>1, identify TSA with highest AIS/RIS
   e - if d>1, pick the one that appears in the article first
2. **Continuous outcomes**
   a - Identify TSAs performed on continuous outcome measures (if NA, skip)
   b - if a>1, identify TSAs from highest rank (primary>secondary>explorative>subgroup)
   c - if b>1, identify TSA with highest acquired information size (AIS)
   d - if c>1, identify TSA with highest AIS/RIS
   e - if d>1, pick the one that appears in the article first

Throughout the extraction process, minor changes were made to the REDCap database to comply with the choices made by the study authors. The largest update was made on the 22nd of June 2022 after extracting data on 260 (48%) of 543 studies. We updated the extraction checklist to contain more generic choices (checkboxes). The extractions and experiences from the choices made in the first 48% of extractions were used as prerequisites for these changes (e.g. choices of outcomes). The updates did not directly affect the ability to answer a certain question but were made to optimise the data extraction and analysis. After all studies were extracted, we adapted the first 48% of the studies to resemble the newly formed extraction checklist.

In the protocol, we initially intended to find all mistakes and errors. As we cannot distinguish between the two, we have decided to change this to “mistakes or errors”. This has been changed in the title and throughout the text.

We have abandoned statistical analysis in the paper. We did mention in the protocol that we would compare systematic reviews with no major mistakes to those with any major mistake. We believe that the current presentation is more in line with the results and still illustrates the challenges with transparency.

Supplemental Figure 1. Distribution of studies across the world, on medical fields and intervention types

Figure 1A. World map

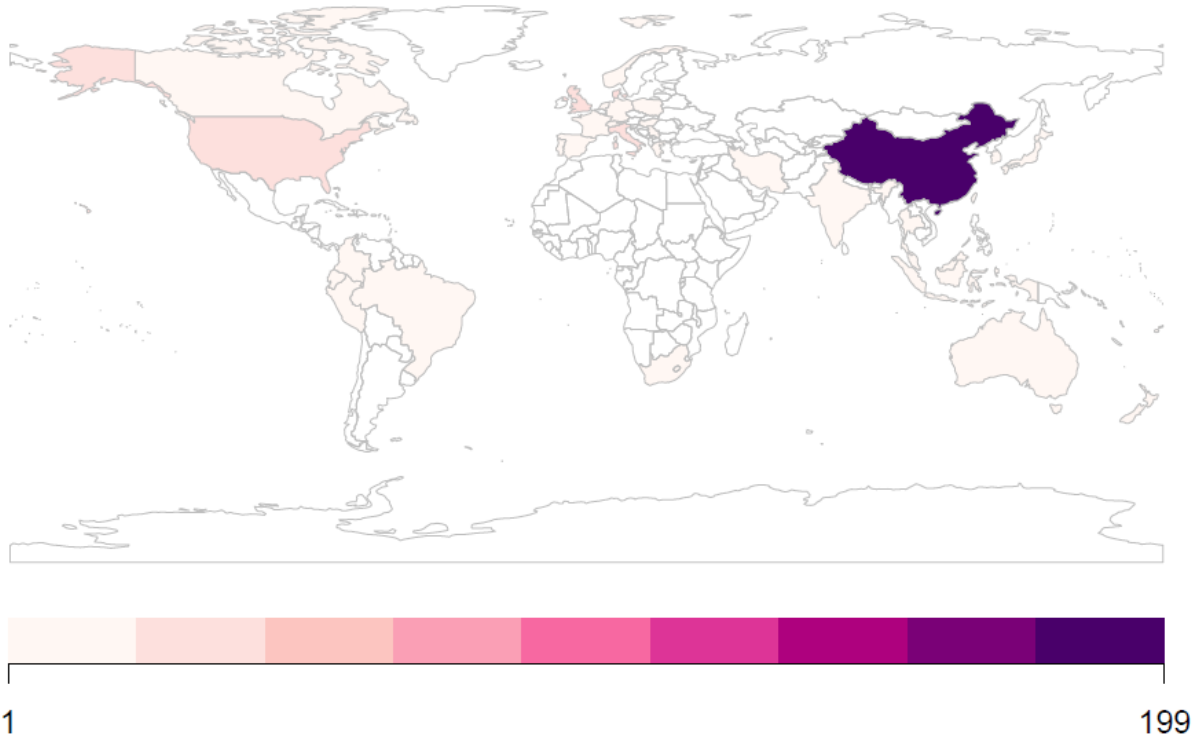

36 out of 195 countries are represented.

	Systematic review	Meta-analysis	Overall
	(N=270)	(N=274)	(N=544)
**country**			
Australia	5 (1.9%)	1 (0.4%)	6 (1.1%)
Bahrain	6 (2.2%)	2 (0.7%)	8 (1.5%)
Brazil	7 (2.6%)	6 (2.2%)	13 (2.4%)
Canada	6 (2.2%)	6 (2.2%)	12 (2.2%)
China	64 (23.7%)	135 (49.3%)	199 (36.6%)
Colombia	1 (0.4%)	0 (0%)	1 (0.2%)
Denmark	33 (12.2%)	2 (0.7%)	35 (6.4%)
Fiji	1 (0.4%)	0 (0%)	1 (0.2%)
France	1 (0.4%)	3 (1.1%)	4 (0.7%)
Germany	3 (1.1%)	5 (1.8%)	8 (1.5%)
Greece	1 (0.4%)	5 (1.8%)	6 (1.1%)
Hungary	2 (0.7%)	3 (1.1%)	5 (0.9%)
India	11 (4.1%)	7 (2.6%)	18 (3.3%)
Italy	17 (6.3%)	16 (5.8%)	33 (6.1%)
Japan	13 (4.8%)	8 (2.9%)	21 (3.9%)
Malaysia	12 (4.4%)	6 (2.2%)	18 (3.3%)
Netherlands	8 (3.0%)	5 (1.8%)	13 (2.4%)
Norway	1 (0.4%)	0 (0%)	1 (0.2%)
Peru	1 (0.4%)	0 (0%)	1 (0.2%)
Poland	1 (0.4%)	0 (0%)	1 (0.2%)
Portugal	1 (0.4%)	2 (0.7%)	3 (0.6%)
Serbia	1 (0.4%)	0 (0%)	1 (0.2%)
Singapore	1 (0.4%)	0 (0%)	1 (0.2%)
South Africa	2 (0.7%)	0 (0%)	2 (0.4%)
South Korea	1 (0.4%)	3 (1.1%)	4 (0.7%)
Spain	1 (0.4%)	5 (1.8%)	6 (1.1%)
Switzerland	14 (5.2%)	5 (1.8%)	19 (3.5%)
Taiwan	11 (4.1%)	8 (2.9%)	19 (3.5%)
Thailand	1 (0.4%)	0 (0%)	1 (0.2%)
United Kingdom	29 (10.7%)	16 (5.8%)	45 (8.3%)
United States	14 (5.2%)	20 (7.3%)	34 (6.3%)
Ecuador	0 (0%)	1 (0.4%)	1 (0.2%)
Indonesia	0 (0%)	1 (0.4%)	1 (0.2%)
Iran	0 (0%)	1 (0.4%)	1 (0.2%)
New Zealand	0 (0%)	1 (0.4%)	1 (0.2%)
Qatar	0 (0%)	1 (0.4%)	1 (0.2%)

36 out of 195 countries are represented. The map shows through colour coding the number of studies included. The table shows the exact numbers from each country based on systematics reviews, meta-analysis and overall.

Figure 1B. Number of studies with Trial Sequential Analysis included within different medical specialities.

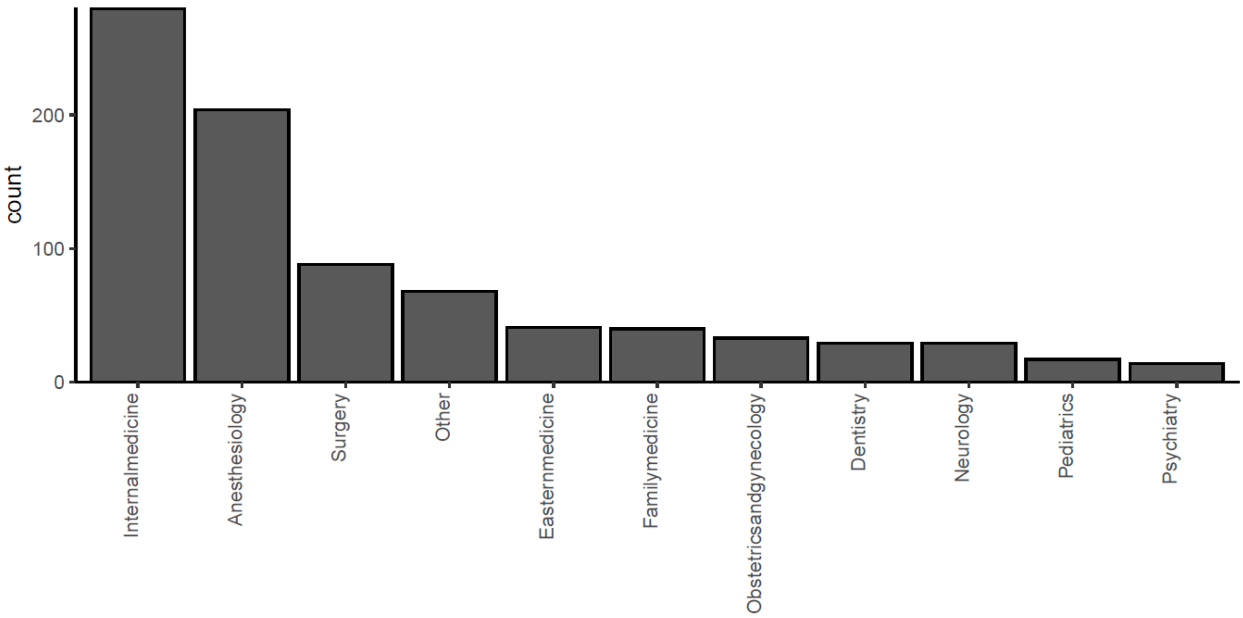

Figure 1C. Number of studies with Trial Sequential Analysis included within different types of interventions.

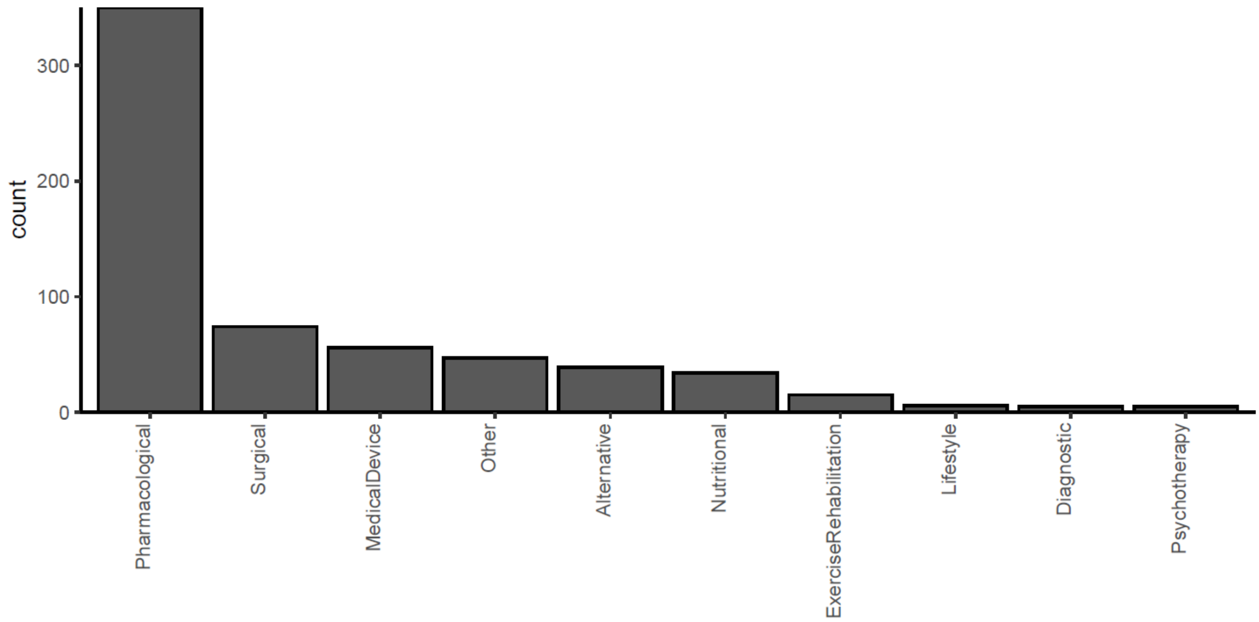

Supplemental Table 1. PRISMA and PRISMA-S Checklist

Section and Topic	Item #	Checklist item	Location where item is reported
**TITLE**			
Title	1	Identify the report as a systematic review.	Page 1
**ABSTRACT**			
Abstract	2	See the PRISMA 2020 for Abstracts checklist.	Page 3
**INTRODUCTION**			
Rationale	3	Describe the rationale for the review in the context of existing knowledge.	Page 7 to8
Objectives	4	Provide an explicit statement of the objective(s) or question(s) the review addresses.	Page 8
**METHODS**			
Eligibility criteria	5	Specify the inclusion and exclusion criteria for the review and how studies were grouped for the syntheses.	Page 9
Information sources	6	Specify all databases, registers, websites, organisations, reference lists and other sources searched or consulted to identify studies. Specify the date when each source was last searched or consulted.	Page 10
Search strategy	7	Present the full search strategies for all databases, registers and websites, including any filters and limits used.	Supplemental Table 2 and page 10
Selection process	8	Specify the methods used to decide whether a study met the inclusion criteria of the review, including how many reviewers screened each record and each report retrieved, whether they worked independently, and if applicable, details of automation tools used in the process.	Page 10
Data collection process	9	Specify the methods used to collect data from reports, including how many reviewers collected data from each report, whether they worked independently, any processes for obtaining or confirming data from study investigators, and if applicable, details of automation tools used in the process.	Page 10 to 11
Data items	10a	List and define all outcomes for which data were sought. Specify whether all results that were compatible with each outcome domain in each study were sought (e.g. for all measures, time points, analyses), and if not, the methods used to decide which results to collect.	Page 10 to 11
	10b	List and define all other variables for which data were sought (e.g. participant and intervention characteristics, funding sources). Describe any assumptions made about any missing or unclear information.	Page 10 to 11
Study risk of bias assessment	11	Specify the methods used to assess risk of bias in the included studies, including details of the tool(s) used, how many reviewers assessed each study and whether they worked independently, and if applicable, details of automation tools used in the process.	Page 11
Effect measures	12	Specify for each outcome the effect measure(s) (e.g. risk ratio, mean difference) used in the synthesis or presentation of results.	Page 11
Synthesis methods	13a	Describe the processes used to decide which studies were eligible for each synthesis (e.g. tabulating the study intervention characteristics and comparing against the planned groups for each synthesis (item #5)).	NA
	13b	Describe any methods required to prepare the data for presentation or synthesis, such as handling of missing summary statistics, or data conversions.	Page 11

Section and Topic	Item #	Checklist item	Location where item is reported
	13c	Describe any methods used to tabulate or visually display results of individual studies and syntheses.	NA
	13d	Describe any methods used to synthesize results and provide a rationale for the choice(s). If meta-analysis was performed, describe the model(s), method(s) to identify the presence and extent of statistical heterogeneity, and software package(s) used.	NA
	13e	Describe any methods used to explore possible causes of heterogeneity among study results (e.g. subgroup analysis, meta-regression).	NA
	13f	Describe any sensitivity analyses conducted to assess robustness of the synthesized results.	Page 12
Reporting bias assessment	14	Describe any methods used to assess risk of bias due to missing results in a synthesis (arising from reporting biases).	NA
Certainty assessment	15	Describe any methods used to assess certainty (or confidence) in the body of evidence for an outcome.	NA
**RESULTS**			
Study selection	16a	Describe the results of the search and selection process, from the number of records identified in the search to the number of studies included in the review, ideally using a flow diagram.	Figure 1<br>Page 13ff
	16b	Cite studies that might appear to meet the inclusion criteria, but which were excluded, and explain why they were excluded.	Supplemental Table 3
Study characteristics	17	Cite each included study and present its characteristics.	NA
Risk of bias in studies	18	Present assessments of risk of bias for each included study.	NA
Results of individual studies	19	For all outcomes, present, for each study: (a) summary statistics for each group (where appropriate) and (b) an effect estimate and its precision (e.g. confidence/credible interval), ideally using structured tables or plots.	Page 13ff
Results of syntheses	20a	For each synthesis, briefly summarise the characteristics and risk of bias among contributing studies.	NA
	20b	Present results of all statistical syntheses conducted. If meta-analysis was done, present for each the summary estimate and its precision (e.g. confidence/credible interval) and measures of statistical heterogeneity. If comparing groups, describe the direction of the effect.	Page 13ff
	20c	Present results of all investigations of possible causes of heterogeneity among study results.	NA
	20d	Present results of all sensitivity analyses conducted to assess the robustness of the synthesized results.	NA
Reporting biases	21	Present assessments of risk of bias due to missing results (arising from reporting biases) for each synthesis assessed.	NA
Certainty of evidence	22	Present assessments of certainty (or confidence) in the body of evidence for each outcome assessed.	NA
**DISCUSSION**			
Discussion	23a	Provide a general interpretation of the results in the context of other evidence.	Page 19
	23b	Discuss any limitations of the evidence included in the review.	Page 23f

Section and Topic	Item #	Checklist item	Location where item is reported
	23c	Discuss any limitations of the review processes used.	Page 23f
	23d	Discuss implications of the results for practice, policy, and future research.	Page 23
**OTHER INFORMATION**			
Registration and protocol	24a	Provide registration information for the review, including register name and registration number, or state that the review was not registered.	Page 9
	24b	Indicate where the review protocol can be accessed, or state that a protocol was not prepared.	Page 9
	24c	Describe and explain any amendments to information provided at registration or in the protocol.	Supplemental Material A
Support	25	Describe sources of financial or non-financial support for the review, and the role of the funders or sponsors in the review.	Page 26
Competing interests	26	Declare any competing interests of review authors.	Page 25
Availability of data, code and other materials	27	Report which of the following are publicly available and where they can be found: template data collection forms; data extracted from included studies; data used for all analyses; analytic code; any other materials used in the review.	Page 25 Supplemental

From: Page MJ, McKenzie JE, Bossuyt PM, Boutron I, Hoffmann TC, Mulrow CD, et al. The PRISMA 2020 statement: an updated guideline for reporting systematic reviews. BMJ 2021;372:n71. doi: 10.1136/bmj.n71

For more information, visit: http://www.prisma-statement.org/

PRISMA-S Checklist

Section/topic	#	Checklist item	Location(s) Reported
**INFORMATION SOURCES AND METHODS**			
Database name	1	Name each individual database searched, stating the platform for each.	P9
Multi-database searching	2	If databases were searched simultaneously on a single platform, state the name of the platform, listing all of the databases searched.	P9
Study registries	3	List any study registries searched.	NA
Online resources and browsing	4	Describe any online or print source purposefully searched or browsed (e.g., tables of contents, print conference proceedings, web sites), and how this was done.	NA
Citation searching	5	Indicate whether cited references or citing references were examined, and describe any methods used for locating cited/citing references (e.g., browsing reference lists, using a citation index, setting up email alerts for references citing included studies).	P10
Contacts	6	Indicate whether additional studies or data were sought by contacting authors, experts, manufacturers, or others.	NA
Other methods	7	Describe any additional information sources or search methods used.	NA
**SEARCH STRATEGIES**			
Full search strategies	8	Include the search strategies for each database and information source, copied and pasted exactly as run.	Supplement
Limits and restrictions	9	Specify that no limits were used, or describe any limits or restrictions applied to a search (e.g., date or time period, language, study design) and provide justification for their use.	P9
Search filters	10	Indicate whether published search filters were used (as originally designed or modified), and if so, cite the filter(s) used.	NA
Prior work	11	Indicate when search strategies from other literature reviews were adapted or reused for a substantive part or all of the search, citing the previous review(s).	NA
Updates	12	Report the methods used to update the search(es) (e.g., rerunning searches, email alerts).	P10
Dates of searches	13	For each search strategy, provide the date when the last search occurred.	P10

**PEER REVIEW**			
Peer review	14	Describe any search peer review process.	P27
**MANAGING RECORDS**			
Total Records	15	Document the total number of records identified from each database and other information sources.	Figure 1<br>Supplement
Deduplication	16	Describe the processes and any software used to deduplicate records from multiple database searches and other information sources.	P10

Supplemental Table 2. Search strategy

Search strategies for
'Trial Sequential Analysis'
(C Gunge Riberholt)

Searches performed 9 July 2021

**Total number of records identified:**	**2109 records**
**Number of duplicates excluded:**	**12 records**
**Number of records in final list:**	**2097 records**
**Number of new records sent to authors:**	**825 records**

Cochrane Database of Systematic Reviews (2021; Issue 7) in the Cochrane Library (253 Cochrane Reviews, 95 Cochrane Protocols)

#1 ("trial sequential" and (analys* or (monitoring next boundar*))) or (cumulative next meta-analys*)

MEDLINE Ovid (2004 to July 2021) (1761 hits)

1. ((trial sequential and (analys* or monitoring boundar*)) or cumulative meta-analys*).mp. [mp=title, abstract, original title, name of substance word, subject heading word, floating sub-heading word, keyword heading word, organism supplementary concept word, protocol supplementary concept word, rare disease supplementary concept word, unique identifier, synonyms]

2. limit 1 to yr="2004 -Current"

Supplemental Table 3. Studies excluded in full text with reason

Study		Reason for exclusion
1	Osterlind J, Gerhardsson J, Myrberg T. Critical care transition programs on readmission or death: a systematic review and meta-analysis. *Acta Anaesthesiol Scand* 2020; **64**:870–83. doi:10.1111/aas.13591	Less than two randomised clinical trials in the forest plot and TSA
2	Song G, Zhang J, Wang X, *et al.* Usefulness of speckle-tracking echocardiography for early detection in children with Duchenne muscular dystrophy: a meta-analysis and trial sequential analysis. *Cardiovasc Ultrasound* 2020;**18**:26. doi:10.1186/s12947-020-00209-y	Less than two randomised clinical trials in the forest plot and TSA
3	XX Y, MQ L, YF Z, *et al.* Associations of KCNQ1 Polymorphisms with the Risk of Type 2 Diabetes Mellitus: an Updated Meta-Analysis with Trial Sequential Analysis. *J Diabetes Res* 2020;**2020**:7145139. doi:10.1155/2020/7145139	Less than two randomised clinical trials in the forest plot and TSA
4	Chen W, Li H, Chen Y, *et al.* Bone-Patellar Tendon-Bone Autografts Versus Hamstring Autografts Using the Same Suspensory Fixations in ACL Reconstruction: a Systematic Review and Meta-analysis. *Orthop J Sport Med* 2019;**7**:2325967119885314. doi:10.1177/2325967119885314	Both observational and RCT in TSA
5	Huang G, Jiang H, Lin Y, *et al.* Prognostic value of plasma fibrinogen in hepatocellular carcinoma: a meta-analysis. *Cancer Manag Res* 2018;**10**:5027–41. doi:10.2147/CMAR.S175780	Less than two randomised clinical trials in the forest plot and TSA
6	Li Y, Du M, Wang S, *et al.* Clinicopathological Implication of Long Non-Coding RNAs SOX2 Overlapping Transcript and Its Potential Target Gene Network in Various Cancers. *Front Genet* 2019;**10**:1375. doi:10.3389/fgene.2019.01375	Less than two randomised clinical trials in the forest plot and TSA
7	Zhu M, Qiu S, Zhang X, *et al.* The associations between CYP24A1 polymorphisms and cancer susceptibility: a meta-analysis and trial sequential analysis. *Pathol Res Pract* 2018;**214**:53–63. doi:10.1016/j.prp.2017.11.014	Less than two randomised clinical trials in the forest plot and TSA
8	Wang X, Huang Y, Li L, *et al.* Assessment of performance of the Gail model for predicting breast cancer risk: a systematic review and meta-analysis with trial sequential analysis. *Breast cancer Res* 2018;**20**:18. doi:10.1186/s13058-018-0947-5	Less than two randomised clinical trials in the forest plot and TSA
9	Jiang W, Shi L, Liu H, *et al.* Systematic review and meta-analysis of the genetic association between protamine polymorphism and male infertility. *Andrologia* 2018;**50**:e12990. doi:10.1111/and.12990	Less than two randomised clinical trials in the forest plot and TSA
10	Cao Z, Wei L, Zhu W, *et al.* Meta-analysis of CDKN2A methylation to find its role in prostate cancer development and progression, and also to find the effect of CDKN2A expression on disease-free survival (PRISMA). *Medicine (Baltimore)* 2018;**97**:e0182. doi:10.1097/MD.0000000000010182	Less than two randomised clinical trials in the forest plot and TSA
11	AK K, SL N, Nizamuddin J, *et al.* Clinical Study Designs and Sources of Error in Medical Research. *J Cardiothorac Vasc Anesth* 2018;**32**:2789–801. doi:10.1053/j.jvca.2018.02.009	Wrong study design
12	Chen L, Wang Y, Zhang T, *et al.* Utility of posaconazole therapeutic drug monitoring and assessment of plasma concentration threshold for effective prophylaxis of invasive fungal infections: a meta-analysis with trial sequential analysis. *BMC Infect Dis* 2018;**18**:155. doi:10.1186/s12879-018-3055-3	Less than two randomised clinical trials in the forest plot and TSA
13	Qiao W, Liu H, Liu R, *et al.* Prognostic and clinical significance of histone deacetylase 1 expression in breast cancer: a meta-analysis. *Clin Chim acta* 2018;**483**:209–15. doi:10.1016/j.cca.2018.05.005	Less than two randomised clinical trials in the forest plot and TSA

14	JS K, Qiu S, Cheng Y, *et al.* Associations between three common single nucleotide polymorphisms (rs266729, rs2241766, and rs1501299) of ADIPOQ and cardiovascular disease: a meta-analysis. *Lipids Health Dis* 2018;**17**:126. doi:10.1186/s12944-018-0767-8	Less than two randomised clinical trials in the forest plot and TSA
15	He H, WT C, YH Z, *et al.* Lack of associations between the FTO polymorphisms and gestational diabetes: a meta-analysis and trial sequential analysis. *Gene* 2018;**677**:169–75. doi:10.1016/j.gene.2018.07.064	Less than two randomised clinical trials in the forest plot and TSA
16	Zhang J, Zhang H, Xiu Y, *et al.* Prognostic Significance of P16INK4a Expression in Penile Squamous Cell Carcinoma: a Meta-Analysis with Trial Sequential Analysis. *Biomed Res Int* 2018;**2018**:8345893. doi:10.1155/2018/8345893	Less than two randomised clinical trials in the forest plot and TSA
17	Naing C, NH H, W ST, *et al.* Association of tumour necrosis factor-alpha (TNF-alpha) gene polymorphisms (-308 G>A and -238 G>A) and the risk of severe dengue: a meta-analysis and trial sequential analysis. *Plos ONE [electronic Resour* 2018;**13**:e0205413. doi:10.1371/journal.pone.0205413	Less than two randomised clinical trials in the forest plot and TSA
18	Meng J, Wang S, Zhang M, *et al.* TP73 G4C14-A4T14 polymorphism and cancer susceptibility: evidence from 36 case-control studies. *Biosci Rep* 2018;**38**. doi:10.1042/BSR20181452	Less than two randomised clinical trials in the forest plot and TSA
19	Qi M, Xiong X. Promoter hypermethylation of RARbeta2, DAPK, hMLH1, p14, and p15 is associated with progression of breast cancer: a PRISMA-compliant meta-analysis. *Medicine (Baltimore)* 2018;**97**:e13666. doi:10.1097/MD.0000000000013666	Less than two randomised clinical trials in the forest plot and TSA
20	Jiang Y, Li W, Lu J, *et al.* Association between PRKAA1 rs13361707 T>C polymorphism and gastric cancer risk: evidence based on a meta-analysis. *Medicine (Baltimore)* 2018;**97**:e0302. doi:10.1097/MD.0000000000010302	Less than two randomised clinical trials in the forest plot and TSA
21	Yan L, Gu Y, Luan T, *et al.* Associations between serum vitamin D and the risk of female reproductive tumors: a meta-analysis with trial sequential analysis. *Medicine (Baltimore)* 2018;**97**:e0360. doi:10.1097/MD.0000000000010360	Less than two randomised clinical trials in the forest plot and TSA
22	Yuan Y, Wang X, Ren L, *et al.* Associations between interleukin-10 gene polymorphisms and systemic lupus erythematosus risk: a meta-analysis with trial sequential analysis. *Clin Exp Rheumatol* 2019;**37**:242–53.NS -	Less than two randomised clinical trials in the forest plot and TSA
23	Singh V, SK B, DVS S, *et al.* SNPs in ERCC1, ERCC2, and XRCC1 genes of the DNA repair pathway and risk of male infertility in the Asian populations: association study, meta-analysis, and trial sequential analysis. *J Assist Reprod Genet* 2019;**36**:79–90. doi:10.1007/s10815-018-1339-6	Less than two randomised clinical trials in the forest plot and TSA
24	MX L, BB L, Li F, *et al.* Roles of Cyclooxygenase-2 gene -765G>C (rs20417) and -1195G>A (rs689466) polymorphisms in gastric cancer: a systematic review and meta-analysis. *Gene* 2019;**685**:125–35. doi:10.1016/j.gene.2018.10.077	Less than two randomised clinical trials in the forest plot and TSA
25	RK M, MA K, Hussain A, *et al.* A trial sequential meta-analysis of TNF-alpha -308G>A (rs800629) gene polymorphism and susceptibility to colorectal cancer. *Biosci Rep* 2019;**39**. doi:10.1042/BSR20181052	Less than two randomised clinical trials in the forest plot and TSA
26	Hoshijima H, Mihara T, Denawa Y, *et al.* Airtraq R is superior to the Macintosh laryngoscope for tracheal intubation: systematic review with trial sequential analysis. *Am J Emerg Med* 2019;**37**:1367–8. doi:10.1016/j.ajem.2018.12.018	Less than two randomised clinical trials in the forest plot and TSA
27	Li Y, Liu B, Jing X, *et al.* Epicardial fat tissue in patients with diabetes mellitus: a systematic review and meta-analysis. *Cardiovasc Diabetol* 2019;**18**:3. doi:10.1186/s12933-019-0807-3	Less than two randomised clinical trials in the forest plot and TSA
28	ML G, HS M, AD M, *et al.* Restrictive Versus Liberal Fluid Regimens in Patients Undergoing Pancreaticoduodenectomy: a Systematic Review and	Both observational and RCT in TSA

	Meta-Analysis. *J Gastrointest Surg* 2019;**23**:1250–65. doi:10.1007/s11605-018-04089-6	
29	Bobo Z, Xin W, Jiang L, *et al.* Robotic gastrectomy versus laparoscopic gastrectomy for gastric cancer: meta-analysis and trial sequential analysis of prospective observational studies. *Surg Endosc* 2019;**33**:1033–48. doi:10.1007/s00464-018-06648-z	Less than two randomised clinical trials in the forest plot and TSA
30	Wang S, Gao H, Zuo J, *et al.* Cyclooxygenase-2 expression correlates with development, progression, metastasis, and prognosis of osteosarcoma: a meta-analysis and trial sequential analysis. *FEBS Open Bio* 2019;**9**:226–40. doi:10.1002/2211-5463.12560	Less than two randomised clinical trials in the forest plot and TSA
31	Chen Y, Hu Y, Song Z. The association between interleukin-6 gene -174G/C single nucleotide polymorphism and sepsis: an updated meta-analysis with trial sequential analysis. *BMC Med Genet* 2019;**20**:35. doi:10.1186/s12881-019-0766-2	Less than two randomised clinical trials in the forest plot and TSA
32	Ming X, Qiu S, Liu X, *et al.* Prognostic Role of Tenascin-C for Cancer Outcome: a Meta-Analysis. *Technol Cancer Res Treat* 2019;**18**:1533033818821106. doi:10.1177/1533033818821106	Less than two randomised clinical trials in the forest plot and TSA
33	Chen J, Chen X, Huang C, *et al.* Predictors of seizure recurrence in patients with surgery for focal cortical dysplasia: pairwise and network meta-analysis and trial sequential analysis. *Childs Nerv Syst* 2019;**35**:753–67. doi:10.1007/s00381-019-04124-0	Less than two randomised clinical trials in the forest plot and TSA
34	Osman M, YA G, Foster T, *et al.* Meta-Analysis of Outcomes of Transcatheter Aortic Valve Implantation Among Patients With Low Gradient Severe Aortic Stenosis. *Am J Cardiol* 2019;**124**:423–9. doi:10.1016/j.amjcard.2019.05.006	Less than two randomised clinical trials in the forest plot and TSA
35	TM K, Wetterslev J, Gluud C, *et al.* Apparently conclusive forest plots on interventions in critical care may be inconclusive-a meta-epidemiological study. *J Clin Epidemiol* 2019;**114**:1–10. doi:10.1016/j.jclinepi.2019.05.011	Wrong study design
36	Chen Z, Liu C, Huang J, *et al.* Clinical Efficacy of Extracorporeal Cardiopulmonary Resuscitation for Adults with Cardiac Arrest: meta-Analysis with Trial Sequential Analysis. *Biomed Res Int* 2019;**2019**:6414673. doi:10.1155/2019/6414673	Less than two randomised clinical trials in the forest plot and TSA
37	Zhu M, Tan Z, Luo Z, *et al.* Association of the vitamin D metabolism gene GC and CYP27B1 polymorphisms with cancer susceptibility: a meta-analysis and trial sequential analysis. *Biosci Rep* 2019;**39**. doi:10.1042/BSR20190368	Less than two randomised clinical trials in the forest plot and TSA
38	Zhao Y, Zhu R, Wang D, *et al.* Genetics of diabetic neuropathy: systematic review, meta-analysis and trial sequential analysis. *Ann Clin Transl Neurol* 2019;**6**:1996–2013. doi:10.1002/acn3.50892	Less than two randomised clinical trials in the forest plot and TSA
39	Terrazzino S, Cargnin S, Deantonio L, *et al.* Impact of ATM rs1801516 on late skin reactions of radiotherapy for breast cancer: evidences from a cohort study and a trial sequential meta-analysis. *Plos ONE [electronic Resour* 2019;**14**:e0225685. doi:10.1371/journal.pone.0225685	Less than two randomised clinical trials in the forest plot and TSA
40	Huang B, YK W, LY Q, *et al.* A functional polymorphism rs10830963 in melatonin receptor 1B associated with the risk of gestational diabetes mellitus. *Biosci Rep* 2019;**39**. doi:10.1042/BSR20190744	Less than two randomised clinical trials in the forest plot and TSA
41	Jiang Y, Li W, Lu J, *et al.* HLA-G +3142 C>G polymorphism and cancer risk: evidence from a meta-analysis and trial sequential analysis. *Medicine (Baltimore)* 2019;**98**:e16067. doi:10.1097/MD.0000000000016067	Less than two randomised clinical trials in the forest plot and TSA
42	Liu Q, Ding L, Qiu X, *et al.* Updated evaluation of endoscopic submucosal dissection versus surgery for early gastric cancer: a systematic review and meta-analysis. *Int J Surg* 2020;**73**:28–41. doi:10.1016/j.ijsu.2019.11.027	Less than two randomised clinical trials in the forest plot and TSA

43	Liu X, Zhao Y, Li Y, *et al.* Quantitative assessment of lncRNA H19 polymorphisms and cancer risk: a meta-analysis based on 48,166 subjects. *Artif cells, nanomedicine, Biotechnol* 2020;**48**:15–27. doi:10.1080/21691401.2019.1699804	Less than two randomised clinical trials in the forest plot and TSA
44	Baloyiannis I, Perivoliotis K, Ntellas P, *et al.* Comparing the safety, efficacy, and oncological outcomes of laparoscopic and open colectomy in transverse colon cancer: a meta-analysis. *Int J Colorectal Dis* 2020;**35**:373–86. doi:10.1007/s00384-020-03516-y	Less than two randomised clinical trials in the forest plot and TSA
45	Xu T, Zhang S, Qiu D, *et al.* Association between matrix metalloproteinase 9 polymorphisms and breast cancer risk: an updated meta-analysis and trial sequential analysis. *Gene* 2020;**759**:144972. doi:10.1016/j.gene.2020.144972	Less than two randomised clinical trials in the forest plot and TSA
46	Miraglia D, LA M, Alonso W. Esmolol in the management of pre-hospital refractory ventricular fibrillation: a systematic review and meta-analysis. *Am J Emerg Med* 2020;**38**:1921–34. doi:10.1016/j.ajem.2020.05.083	Less than two randomised clinical trials in the forest plot and TSA
47	HL C, GR C, PJ H, *et al.* Decisive evidence corroborates a null relationship between MTHFR C677T and chronic kidney disease: a case-control study and a meta-analysis. *Medicine (Baltimore)* 2020;**99**:e21045. doi:10.1097/MD.0000000000021045	Less than two randomised clinical trials in the forest plot and TSA
48	Zhao J, Li D, Tang H, *et al.* Association of vascular endothelial growth factor polymorphisms with polycystic ovarian syndrome risk: a meta-analysis. *Reprod Biol Endocrinol* 2020;**18**:18. doi:10.1186/s12958-020-00577-0	Less than two randomised clinical trials in the forest plot and TSA
49	GCM S, Nikolakopoulou A, Efthimiou O, *et al.* Evaluation of Cumulative Meta-analysis of Rare Events as a Tool for Clinical Trials Safety Monitoring. *JAMA Netw open* 2020;**3**:e2015031. doi:10.1001/jamanetworkopen.2020.15031	Wrong study design
50	Zhuo C, Yi T, Wei C, *et al.* Association of cytotoxic T lymphocyte-associated protein 4 gene -1772T/C polymorphism with gastric cancer risk: a prisma-compliant meta-analysis. *Medicine (Baltimore)* 2020;**99**:e23542. doi:10.1097/MD.0000000000023542	Less than two randomised clinical trials in the forest plot and TSA
51	Cargnin S, Galli U, JI S, *et al.* CTLA-4 rs231775 and risk of acute renal graft rejection: an updated meta-analysis with trial sequential analysis. *Sci Rep* 2020;**10**:12850. doi:10.1038/s41598-020-69849-4	Less than two randomised clinical trials in the forest plot and TSA
52	Zhang J, Cai Z, Ma C, *et al.* Impacts of Outdoor Air Pollution on Human Semen Quality: a Meta-Analysis and Systematic Review. *Biomed Res Int* 2020;**2020**:7528901. doi:10.1155/2020/7528901	Less than two randomised clinical trials in the forest plot and TSA
53	HC C, WT C, TL S, *et al.* PPARG Pro12Ala Polymorphism with CKD in Asians: a Meta-Analysis Combined with a Case-Control Study-A Key for Reaching Null Association. *Genes (Basel)* 2020;**11**. doi:10.3390/genes11060705	Less than two randomised clinical trials in the forest plot and TSA
54	XQ X, DG C, Yang Q. Lack of association between BDNF rs6265 polymorphism and risk of type 2 diabetes: a protocol for meta-analysis and trial sequential analysis. *Medicine (Baltimore)* 2021;**100**:e23305. doi:10.1097/MD.0000000000023305	Less than two randomised clinical trials in the forest plot and TSA
55	YE W, Ma L, Zhang H, *et al.* Significant association between the endothelial lipase gene 584C/T polymorphism and coronary artery disease risk. *Biosci Rep* 2020;**40**. doi:10.1042/BSR20200027	Less than two randomised clinical trials in the forest plot and TSA
56	CY C, MY W, YJ C, *et al.* Anesthesia and Long-term Oncological Outcomes: a Systematic Review and Meta-analysis. *Anesth Analg* 2021;**132**:623–34. doi:10.1213/ANE.0000000000005237	Less than two randomised clinical trials in the forest plot and TSA
57	Doleman B, Mathiesen O, JC J, *et al.* Methodologies for systematic reviews with meta-analysis of randomised clinical trials in pain, anaesthesia, and	Wrong study design

	perioperative medicine. *Br J Anaesth* 2021;**126**:903–11. doi:10.1016/j.bja.2021.01.004	
58	GH Q, CH W, HG Z, *et al.* Comprehensive analysis of the effect of rs2295080 and rs2536 polymorphisms within the mTOR gene on cancer risk. *Biosci Rep* 2020;**40**. doi:10.1042/BSR20191825	Less than two randomised clinical trials in the forest plot and TSA
59	Liu J, Song G, Zhao G, *et al.* Lack of association between IGF2BP2 rs4402960 polymorphism and gestational diabetes mellitus: a case-control study, meta-analysis and trial sequential analysis. *Biosci Rep* 2020;**40**. doi:10.1042/BSR20200990	Less than two randomised clinical trials in the forest plot and TSA
60	Perivoliotis K, Baloyiannis I, Sarakatsianou C, *et al.* Comparing the efficacy and safety of laparoscopic and robotic adrenalectomy: a meta-analysis and trial sequential analysis. *Langenbecks Arch Surg* 2020;**405**:125–35. doi:10.1007/s00423-020-01860-9	Less than two randomised clinical trials in the forest plot and TSA
61	Cai W, Liu X, Li Y, *et al.* New sights on the associations between the XRCC1 gene polymorphisms and hepatocellular carcinoma susceptibility. *J Cell Biochem* 2020;**121**:1005–22. doi:10.1002/jcb.29335	Less than two randomised clinical trials in the forest plot and TSA
62	Haque F, MBI R, SHM A, *et al.* Performance analysis of noninvasive electrophysiological methods for the assessment of diabetic sensorimotor polyneuropathy in clinical research: a systematic review and meta-analysis with trial sequential analysis. *Sci Rep* 2020;**10**:21770. doi:10.1038/s41598-020-78787-0	Less than two randomised clinical trials in the forest plot and TSA
63	Perivoliotis K, Baloyiannis I, Symeonidis D, *et al.* The role of bursectomy in the surgical management of gastric cancer: a meta-analysis and systematic review. *Updates Surg* 2020;**72**:939–50. doi:10.1007/s13304-020-00801-x	Both observational and RCT in TSA
64	Rezaei F, Mohammadi H, Heydari M, *et al.* Association between IL-8 (-251T/A) and IL-6 (-174G/C) Polymorphisms and Oral Cancer Susceptibility: a Systematic Review and Meta-Analysis. *Medicina (Kaunas)* 2021;**57**. doi:10.3390/medicina57050405	Less than two randomised clinical trials in the forest plot and TSA
65	Chen G, Hu C, Song Y, *et al.* Relationship between Aurora-A V57I Polymorphism and the Risk of Cancer: a Meta-Analysis and Trial Sequential Analysis. *J Cancer* 2020;**11**:3225–34. doi:10.7150/jca.40567	Less than two randomised clinical trials in the forest plot and TSA
66	Chen G, Hu C, Song Y, *et al.* Relationship Between the ApaI (rs7975232), BsmI (rs1544410), FokI (rs2228570), and TaqI (rs731236) Variants in the Vitamin D Receptor Gene and Urolithiasis Susceptibility: an Updated Meta-Analysis and Trial Sequential Analysis. *Front Genet* 2020;**11**:234. doi:10.3389/fgene.2020.00234	Less than two randomised clinical trials in the forest plot and TSA
67	Gu Y, Cheng H, Zong L, *et al.* Operative and Oncological Outcomes Comparing Sentinel Node Mapping and Systematic Lymphadenectomy in Endometrial Cancer Staging: meta-Analysis With Trial Sequential Analysis. *Front Oncol* 2020;**10**:580128. doi:10.3389/fonc.2020.580128	Less than two randomised clinical trials in the forest plot and TSA
68	Song G, Qiao W, Sun L, *et al.* A Meta-Analysis of Different Types of Cardiac Adipose Tissue in HIV Patients. *Biomed Res Int* 2020;**2020**:8234618. doi:10.1155/2020/8234618	Less than two randomised clinical trials in the forest plot and TSA
69	Bassi F, Cicciu M, R DL, *et al.* Piezoelectric bone surgery compared with conventional rotary instruments in oral surgery and implantology: summary and consensus statements of the International Piezoelectric Surgery Academy Consensus Conference 2019. *Int J oral Implantol* 2020;**13**:235–9.NS -	Wrong study design
70	Cui J, Wang L, Piao J, *et al.* Initial surgical versus non-surgical treatments for advanced hypopharyngeal cancer: a meta-analysis with trial sequential analysis. *Int J Surg* 2020;**82**:249–59. doi:10.1016/j.ijsu.2020.04.059	Both observational and RCT in TSA

71	CS P, DL V, Casazza G, *et al.* Glucocorticosteroids for people with alcoholic hepatitis (Cochrane review). *Ter Arkh* 2019;**91**:52–66. doi:10.26442/00403660.2019.08.000354	Language not in English
72	Liu T, Zhan Y, Wang Y, *et al.* Obstructive sleep apnea syndrome and risk of renal impairment: a systematic review and meta-analysis with trial sequential analysis. *Schlaf & Atmung [Sleep & breathing]* 2020;**25**:17–27. doi:10.1007/s11325-020-02090-5	Less than two randomised clinical trials in the forest plot and TSA
73	A FD, F DL, D van W. Arterial spin labeling MR imaging for differentiation between high- and low-grade glioma-a meta-analysis. *Neuro Oncol* 2018;**20**:1450–61. doi:10.1093/neuonc/noy095	Less than two randomised clinical trials in the forest plot and TSA
74	Wang B, Huang Y. Effect of aspirin use on neoadjuvant chemoradiotherapy for rectal cancer: a meta-analysis with trial sequential analysis. *J Cancer Res Clin Oncol* 2020;**146**:2161–71. doi:10.1007/s00432-020-03222-w	Less than two randomised clinical trials in the forest plot and TSA
75	Pergialiotis V, Fanaki M, Bellos I, *et al.* Evaluation of umbilical cord entanglement as a predictive factor of adverse pregnancy outcomes: a meta-analysis. *Eur J Obstet Gynecol Reprod Biol* 2019;**243**:150–7. doi:10.1016/j.ejogrb.2019.10.038	Less than two randomised clinical trials in the forest plot and TSA
76	Qian C, He Y, Li Y, *et al.* Association Between Aspirin Use and Risk of Aneurysmal Subarachnoid Hemorrhage: a Meta-analysis. *World Neurosurg* 2020;**138**:299–308. doi:10.1016/j.wneu.2020.01.120	Less than two randomised clinical trials in the forest plot and TSA
77	Zhong D, Wu C, Bai J, *et al.* Comparative diagnostic efficacy of serum Krebs von den Lungen-6 and surfactant D for connective tissue disease-associated interstitial lung diseases: a meta-analysis. *Medicine (Baltimore)* 2020;**99**:e19695. doi:10.1097/MD.0000000000019695	Less than two randomised clinical trials in the forest plot and TSA
78	Feng Y, Zhang Z, Lou T, *et al.* The security of radical trachelectomy in the treatment of IA-IIA cervical carcinoma requires further evaluation: updated meta-analysis and trial sequential analysis. *Arch Gynecol Obstet* 2019;**299**:1525–36. doi:10.1007/s00404-019-05141-9	Less than two randomised clinical trials in the forest plot and TSA
79	Wu Z, Zhang S, Xu J, *et al.* Norepinephrine vs Vasopressin: which Vasopressor Should Be Discontinued First in Septic Shock? A Meta-Analysis. *Shock* 2020;**53**:50–7. doi:10.1097/SHK.0000000000001345	Less than two randomised clinical trials in the forest plot and TSA
80	Liu B, Li Y, Liu Y, *et al.* Association of epicardial adipose tissue with non-alcoholic fatty liver disease: a meta-analysis. *Hepatol Int* 2019;**13**:757–65. doi:10.1007/s12072-019-09972-1	Less than two randomised clinical trials in the forest plot and TSA
81	Pergialiotis V, Bellos I, Hatziagelaki E, *et al.* Progestogens for the prevention of preterm birth and risk of developing gestational diabetes mellitus: a meta-analysis. *Am J Obstet Gynecol* 2019;**221**:429-436.e5. doi:10.1016/j.ajog.2019.05.033	Less than two randomised clinical trials in the forest plot and TSA
82	HC W, Ooi Y, SJ P, *et al.* The role of three interleukin 10 gene polymorphisms (- 1082 A > G, - 819 C > T, - 592 A > C) in the risk of chronic and aggressive periodontitis: a meta-analysis and trial sequential analysis. *BMC Oral Health* 2018;**18**:171. doi:10.1186/s12903-018-0637-9	Less than two randomised clinical trials in the forest plot and TSA
83	Shi H, He H, SC O, *et al.* Association of STAT3 and STAT4 polymorphisms with susceptibility to chronic hepatitis B virus infection and risk of hepatocellular carcinoma: a meta-analysis. *Biosci Rep* 2019;**39**. doi:10.1042/BSR20190783	Less than two randomised clinical trials in the forest plot and TSA
84	Jiang M, C-l L, C-q P, *et al.* The risk of bloodstream infection associated with totally implantable venous access ports in cancer patient: a systematic review and meta-analysis. *Support care cancer* 2020;**28**:361–72. doi:10.1007/s00520-019-04809-x	Less than two randomised clinical trials in the forest plot and TSA

85	Heesen M, Hilber N, Rijs K, *et al.* Intrathecal catheterisation after observed accidental dural puncture in labouring women: update of a meta-analysis and a trial-sequential analysis. *Int J Obstet Anesth* 2020;**41**:71–82. doi:10.1016/j.ijoa.2019.08.001	Less than two randomised clinical trials in the forest plot and TSA
86	Zhao B, Hu L, Dong Y, *et al.* The Effect of Magnesium Intake on Stroke Incidence: a Systematic Review and Meta-Analysis With Trial Sequential Analysis. *Front Neurol [electronic Resour* 2019;. **10**:852. doi:10.3389/fneur.2019.00852	Less than two randomised clinical trials in the forest plot and TSA
87	Wang Y, Huang S, Wu X, *et al.* Correlation between MCP-1-2518A/G polymorphism and the risk of Alzheimer's disease. *J Neural Transm* 2018;**125**:1781–6. doi:10.1007/s00702-018-1936-7	Less than two randomised clinical trials in the forest plot and TSA
88	Al-Razhi B, Fadag A, AY A. Limited Evidence Suggests that Single-visit RCT may Have a Similar Risk of Long-term Complications but a Higher Risk of Flare-Ups Compared with Multiple-Visit Endodontic Treatments. *J Evid Based Dent Pract* 2018;**18**:243–5. doi:10.1016/j.jebdp.2018.06.001	Wrong study design
89	Feng Y, Zhang Z, Lou T, *et al.* The safety of fertility preservation for microinvasive cervical adenocarcinoma: a meta-analysis and trial sequential analysis. *Arch Gynecol Obstet* 2018;**298**:465–75. doi:10.1007/s00404-018-4799-0	Less than two randomised clinical trials in the forest plot and TSA
90	Thakur N, Kumari S, Mehrotra R. Association between Cyclin D1 G870A (rs9344) polymorphism and cancer risk in Indian population: meta-analysis and trial sequential analysis. *Biosci Rep* 2018;**38**. doi:10.1042/BSR20180694	Less than two randomised clinical trials in the forest plot and TSA
91	Marti-Carvajal A, MA AEA, Marti-Amarista C, *et al.* Antiplatelet agents for preventing vaso-occlusive events in people with sickle cell disease: a systematic review. *Clin Adv Hematol Oncol* 2019;**17**:234–43.NS -	Less than two randomised clinical trials in the forest plot and TSA
92	Scurt FG, Ewert L, Mertens PR, *et al.* Clinical outcomes after ABO-incompatible renal transplantation: a systematic review and meta-analysis. *Lancet* 2019;**393**:2059–72. doi:10.1016/S0140-6736(18)32091-9	Less than two randomised clinical trials in the forest plot and TSA
93	Han S, Huang T, Li W, *et al.* Association Between Hypoxia-Inducible Factor-2alpha (HIF-2alpha) Expression and Colorectal Cancer and Its Prognostic Role: a Systematic Analysis. *Cell Physiol Biochem* 2018;**48**:516–27. doi:10.1159/000491806	Less than two randomised clinical trials in the forest plot and TSA
94	Zheng F, Yu H. RASSF1A promoter methylation was associated with the development, progression and metastasis of cervical carcinoma: a meta-analysis with trial sequential analysis. *Arch Gynecol Obstet* 2018;**297**:467–77. doi:10.1007/s00404-017-4639-7	Less than two randomised clinical trials in the forest plot and TSA
95	Li Q, Ma C, Zhang Z, *et al.* Association between cyclooxygenase-2 (COX-2) 8473 T > C polymorphism and cancer risk: a meta-analysis and trial sequential analysis. *BMC Cancer* 2018;**18**:847. doi:10.1186/s12885-018-4753-3	Less than two randomised clinical trials in the forest plot and TSA
96	Harrison W, Angoulvant F, House S, *et al.* Hypertonic saline in bronchiolitis and type i error: a trial sequential analysis. *Pediatrics* 2018;**142**. doi:10.1542/peds.2018-1144	Less than two randomised clinical trials in the forest plot and TSA
97	Wang Y, XL W, Deng X, *et al.* Association of CD14-260 (-159) C/T and Alzheimer's disease: systematic review and trial sequential analyses. *J Neural Transm* 2018;:1313–8. doi:10.1007/s00702-018-1896-y	Less than two randomised clinical trials in the forest plot and TSA
98	SA A-M, Halboub E, HM A-S, *et al.* Association between serum zinc levels and recurrent aphthous stomatitis: a meta-analysis with trial sequential analysis. *Clin Oral Investig* 2021;**25**:407–15. doi:10.1007/s00784-020-03704-8	Less than two randomised clinical trials in the forest plot and TSA

99	Pasin L, Cavalli G, Navalesi P, *et al.* Anakinra for patients with COVID-19: a meta-analysis of non-randomized cohort studies. *Eur J Intern Med* 2021;**86**:34–40. doi:10.1016/j.ejim.2021.01.016	Less than two randomised clinical trials in the forest plot and TSA
100	YS R, LF L, Peng T, *et al.* The effect of milrinone on mortality in adult patients who underwent CABG surgery: a systematic review of randomized clinical trials with a meta-analysis and trial sequential analysis. *BMC Cardiovasc Disord* 2020;**20**:328. doi:10.1186/s12872-020-01598-8	Only forest plot, not Trial Sequential Analysis
101	Capion T, Lilja-Cyron A, Juhler M, *et al.* Prompt closure versus gradual weaning of external ventricular drainage for hydrocephalus in adult patients with aneurysmal subarachnoid haemorrhage: a systematic review. *BMJ Open* 2020;**10**:e040722. doi:10.1136/bmjopen-2020-040722	Less than two randomised clinical trials in the forest plot and TSA
102	Tian G, Li G, Guan L, *et al.* Pretreatment albumin-to-alkaline phosphatase ratio as a prognostic indicator in solid cancers: a meta-analysis with trial sequential analysis. *Int J Surg* 2020;**81**:66–73. doi:10.1016/j.ijsu.2020.07.024	Less than two randomised clinical trials in the forest plot and TSA
103	Ma M, Tao L, Liu A, *et al.* Macrophage migration inhibitory factor-794 catt microsatellite polymorphism and risk of tuberculosis: a meta-analysis. *Biosci Rep* 2018;**38**. doi:10.1042/BSR20171626	Less than two randomised clinical trials in the forest plot and TSA
104	Hu Y, Xu K, Jiang L, *et al.* Associations between Three CTLA-4 Polymorphisms and Hashimoto's Thyroiditis Risk: an Updated Meta-Analysis with Trial Sequential Analysis. *Genet Test Mol Biomarkers* 2018;**22**:224–36. doi:10.1089/gtmb.2017.0243	Less than two randomised clinical trials in the forest plot and TSA
105	Zhang R, Huo C, Wang X, *et al.* Two Common MTHFR Gene Polymorphisms (C677T and A1298C) and Fetal Congenital Heart Disease Risk: an Updated Meta-Analysis with Trial Sequential Analysis. *Cell Physiol Biochem* 2018;**45**:2483–96. doi:10.1159/000488267	Less than two randomised clinical trials in the forest plot and TSA
106	SH Q, Boulemden A, Szafranek A, *et al.* Meta-analysis of sutureless technology versus standard aortic valve replacement and transcatheter aortic valve replacement. *Eur J cardio-thoracic Surg* 2018;**53**:463–71. doi:10.1093/ejcts/ezx307	Both observational and RCT in TSA
107	Liu G. CDH1 promoter methylation in patients with cervical carcinoma: a systematic meta-analysis with trial sequential analysis. *Future Oncol* 2018;**14**:51–63. doi:10.2217/fon-2017-0267	Less than two randomised clinical trials in the forest plot and TSA
108	KB B. Pulmonary artery perfusion versus no pulmonary per-fusion during cardiopulmonary bypass. *Dan Med J* 2018;**65**.NS -	Wrong study design
109	H-L C, M-L Z, Qin G. Prevention strategy for father-to-child transmission of hepatitis B virus: a systematic review and meta-analysis. *J Matern neonatal Med* 2018;**31**:3275–82. doi:10.1080/14767058.2017.1368482	Less than two randomised clinical trials in the forest plot and TSA
110	Yang F, Wei K, Qin Z, *et al.* Association between TNF-a-308G/A polymorphism and esophageal cancer risk: an updated meta-analysis and trial sequential analysis. *J Cancer* 2019;**10**:1086–96. doi:10.7150/jca.29390	Less than two randomised clinical trials in the forest plot and TSA
111	Han S, Huang T, Hou F, *et al.* The prognostic value of hypoxia-inducible factor-1alpha in advanced cancer survivors: a meta-analysis with trial sequential analysis. *Ther Adv Med Oncol* 2019;**11**:1758835919875851. doi:10.1177/1758835919875851	Less than two randomised clinical trials in the forest plot and TSA
112	Xiong Y, Zhang Q, Ye J, *et al.* Associations between three XRCC1 polymorphisms and hepatocellular carcinoma risk: a meta-analysis of case-control studies. *Plos ONE [electronic Resour* 2018;**13**:e0206853. doi:10.1371/journal.pone.0206853	Less than two randomised clinical trials in the forest plot and TSA

113	Casans-Frances R, AT R-A, MA G-R, *et al.* The importance of trial sequential analysis in the evaluation of the results of a meta-analysis. *Minerva Anestesiol* 2019;**85**:342–3. doi:10.23736/S0375-9393.19.13599-7	Wrong study design
114	Kheiri B, Bachuwa G, DL B. Drug-eluting stents versus bare-metal stents with a single month of dual antiplatelet therapy: a trial sequential analysis. *J Thromb Thrombolysis* 2019;**48**:11–3. doi:10.1007/s11239-019-01861-6	Only Trial Sequential Analysis, no forest plot
115	Kumar A, Shariff M. Rate of reoperation at 1 year for aortic repair vs replacement in aortic regurgitation. A trial sequence analysis of published meta-analysis results. *J Card Surg* 2019;**34**:714–6. doi:10.1111/jocs.14164	Only Trial Sequential Analysis, no forest plot
116	Kumar A, Shariff M. Trial sequential analysis of studies comparing the frequency of target-vessel revascularization with drug-coated balloons as compared with second-generation drug-eluting stents in coronary in-stent restenosis: have we generated enough evidence in the fiel. *Indian Heart J* 2019;**71**:288–90. doi:10.1016/j.ihj.2019.06.001	Only Trial Sequential Analysis, no forest plot
117	Zhu Y, Chen Z, Jiang H, *et al.* The genetic association between EGF A61G polymorphism (rs4444903) and risk of colorectal cancer: an update meta-analysis and trial sequential analysis. *Medicine (Baltimore)* 2019;**98**:e14007. doi:10.1097/MD.0000000000014007	Less than two randomised clinical trials in the forest plot and TSA
118	Manogaran M, SS Y. Data for beta-blockade in ACLS - A trial sequential analysis. *Resuscitation* 2020;**150**:191–2. doi:10.1016/j.resuscitation.2020.02.019	Only Trial Sequential Analysis, no forest plot
119	Alfirevic Z, Gates S. Trial sequential analysis: useful or useless? *BJOG* 2020;**127**:1227–8. doi:10.1111/1471-0528.16282	Wrong study design
120	Shah A, AF S. Trial sequential analysis: adding a new dimension to meta-analysis. *Anaesthesia* 2020;**75**:15–20. doi:10.1111/anae.14705	Wrong study design
121	HH K, HP G. Optimal Interpretation of Trial Sequential Analysis in Understanding the Effect of Dexmedetomidine on Acute Kidney Injury After Cardiac Surgery. *J Cardiothorac Vasc Anesth* 2020;**34**:2548. doi:10.1053/j.jvca.2020.04.016	Wrong study design
122	KR K, Taljaard M. Enough is enough: does trial sequential analysis tell us when we don't need to do any more studies? *Anaesthesia* 2021;**76**:594–7. doi:10.1111/anae.15242	Wrong study design
123	Lu S, Wang Y, Hu J, *et al.* The IL-6 rs1800795 and rs1800796 polymorphisms are associated with coronary artery disease risk. *J Cell Mol Med* 2020;**24**:6191–207. doi:10.1111/jcmm.15246	Less than two randomised clinical trials in the forest plot and TSA
124	Amar A, Afzal A, SA H, *et al.* Association of vitamin D receptor gene polymorphisms and risk of urolithiasis: results of a genetic epidemiology study and comprehensive meta-analysis. *Urolithiasis* 2020;**48**:385–401. doi:10.1007/s00240-019-01157-7	Less than two randomised clinical trials in the forest plot and TSA
125	Mahto H, Pati A, SK S, *et al.* Association of MBL-2 gene polymorphisms with systemic lupus erythematosus: an updated meta-analysis and trial sequential analysis. *Lupus* 2020;**29**:1227–37. doi:10.1177/0961203320939156	Less than two randomised clinical trials in the forest plot and TSA
126	Song G, Qiao W, Liu K, *et al.* Epicardial adipose tissue in patients with chronic kidney disease: a meta-analysis study and trial sequential analysis. *Int Urol Nephrol* 2020;**52**:2345–55. doi:10.1007/s11255-020-02575-y	Less than two randomised clinical trials in the forest plot and TSA
127	Ang E, KT N, ZX L, *et al.* Effect of regional anaesthesia only versus general anaesthesia on cancer recurrence rate: a systematic review and meta-analysis with trial sequential analysis. *J Clin Anesth* 2020;**67**:110023. doi:10.1016/j.jclinane.2020.110023	Less than two randomised clinical trials in the forest plot and TSA

128	Liu J, Song G, Zhao G, *et al.* Gene polymorphism associated with TGF-beta1 and susceptibility to preeclampsia: a meta-analysis and trial sequential analysis. *J Obstet Gynaecol Res* 2021;**47**:2031–41. doi:10.1111/jog.14751	Less than two randomised clinical trials in the forest plot and TSA
129	Jiang C, Li L, Wu M, *et al.* Association of KCNJ10 variants and the susceptibility to clinical epilepsy. *Clin Neurol Neurosurg* 2021;**200**:106340. doi:10.1016/j.clineuro.2020.106340	Less than two randomised clinical trials in the forest plot and TSA
130	Wang C, Zhou X, Liu H, *et al.* Three polymorphisms of renin-angiotensin system and preeclampsia risk. *J Assist Reprod Genet* 2020;**37**:3121–42. doi:10.1007/s10815-020-01971-8	Less than two randomised clinical trials in the forest plot and TSA
131	Kumar A, Doshi R, Shariff M. Role of antibiotic envelopes in preventing cardiac implantable electronic device infection: a meta-analysis of 14 859 procedures. *J arrhythmia* 2020;**36**:176–9. doi:10.1002/joa3.12262	Less than two randomised clinical trials in the forest plot and TSA
132	Yuan M, Yu C, Yu K. Association of human XPA rs1800975 polymorphism and cancer susceptibility: an integrative analysis of 71 case-control studies. *Cancer Cell Int* 2020;**20**:164. doi:10.1186/s12935-020-01244-5	Less than two randomised clinical trials in the forest plot and TSA
133	Huang J, Gao H, HZ T. SOX1 Promoter Hypermethylation as a Potential Biomarker for High-Grade Squamous Intraepithelial Neoplasia Lesion and Cervical Carcinoma: a Meta-Analysis With Trial Sequential Analysis. *Front Genet* 2020;**11**:633. doi:10.3389/fgene.2020.00633	Less than two randomised clinical trials in the forest plot and TSA
134	Caputo R, Asprea M, Giovannetti L, *et al.* Nephrotoxicity of three formulations of amphotericin B: trial sequential analysis. *Arch Med Sci* 2020;**16**:1493–5. doi:10.5114/aoms.2020.93338	Only Trial Sequential Analysis, no forest plot
135	LS S, LM L, LH Z, *et al.* Ablation strategies for arrhythmogenic right ventricular cardiomyopathy: a systematic review and meta-analysis. *J Geriatr Cardiol* 2020;**17**:694–703. doi:10.11909/j.issn.1671-5411.2020.11.001	Less than two randomised clinical trials in the forest plot and TSA
136	JM A, Bakare L, AM C, *et al.* Cefazolin Versus Anti-Staphylococcal Penicillins for the Treatment of Patients with Methicillin-Susceptible Staphylococcus aureus Infection: a Meta-Analysis with Trial Sequential Analysis. *Infect Dis Ther* 2019;**8**:671–86. doi:10.1007/s40121-019-00259-4	Less than two randomised clinical trials in the forest plot and TSA
137	Zhang Y, Chen L, Chen H. A meta-analysis of the correlation between non-steroidal anti-inflammatory drugs and recurrent colorectal adenomatous polyps. *Am J Transl Res* 2021;**13**:2432–8.NS -	Only forest plot, no Trial Sequential Analysis
138	Liu X, Zhao Y, Li Y, *et al.* Association between HOTAIR genetic polymorphisms and cancer susceptibility: a meta-analysis involving 122,832 subjects. *Genomics* 2020;**112**:3036–55. doi:10.1016/j.ygeno.2020.05.018	Less than two randomised clinical trials in the forest plot and TSA
139	Liu J, MQ L, DF C, *et al.* The Association between Interleukin-6 Gene Polymorphisms and Risk of Systemic Lupus Erythematosus: a Meta-analysis with Trial Sequential Analysis. *Immunol Invest* 2021;**50**:259–72. doi:10.1080/08820139.2020.1769646	Less than two randomised clinical trials in the forest plot and TSA
140	Corrao S, Natoli G, Argano C. Nonalcoholic fatty liver disease is associated with intrahepatic cholangiocarcinoma and not with extrahepatic form: definitive evidence from meta-analysis and trial sequential analysis. *Eur J Gastroenterol Hepatol* 2021;**33**:62–8. doi:10.1097/MEG.0000000000001684	Less than two randomised clinical trials in the forest plot and TSA
141	YH H, WH F, DJ T, *et al.* The Decisive Case-Control Study Elaborates the Null Association between ESR1 XbaI and Osteoarthritis in Asians: a Case-Control Study and Meta-Analysis. *Genes (Basel)* 2021;**12**. doi:10.3390/genes12030404	Less than two randomised clinical trials in the forest plot and TSA
142	Zhang J, Li X, Cai Z, *et al.* Association between testosterone with type 2 diabetes in adult males, a meta-analysis and trial sequential analysis. *Aging male* 2020;**23**:607–18. doi:10.1080/13685538.2018.1557139	Less than two randomised clinical trials in the forest plot and TSA

143	Osman M, MB M, Regner S, *et al.* Induced Hypothermia in Patients with Cardiac Arrest and a Non-shockable Rhythm: meta-analysis and Trial Sequential Analysis. *Neurocrit Care* 2021;**34**:279–86. doi:10.1007/s12028-020-01034-x	Less than two randomised clinical trials in the forest plot and TSA
144	PB W, XM W, Qian R, *et al.* Association between IL12B polymorphisms and inflammatory bowel disease in Caucasian population: a meta-analysis. *Cytokine* 2020;**136**:155296. doi:10.1016/j.cyto.2020.155296	Less than two randomised clinical trials in the forest plot and TSA
145	JSK C. Aspirin-omitted dual antithrombotics vs. triple antithrombotics: a trial sequential analysis. *Eur Hear J Cardiovasc Pharmacother* 2021;**7**:e32–4. doi:10.1093/ehjcvp/pvaa061	Wrong study design
146	Yan F, Jiang Q, He M, *et al.* PARP inhibitor treatment of advanced breast cancer beyond the BRCA-mutated type: a meta-analysis. *Future Oncol* 2021;**17**:2381–93. doi:10.2217/fon-2020-1175	Only forest plot, no Trial Sequential Analysis
147	WC H, YY C, YH L, *et al.* Composite Cardiovascular Outcomes in Patients With Primary Aldosteronism Undergoing Medical Versus Surgical Treatment: a Meta-Analysis. *Front Endocrinol (Lausanne)* 2021;**12**:644260. doi:10.3389/fendo.2021.644260	Less than two randomised clinical trials in the forest plot and TSA
148	Liu J, Song G, Zhao G, *et al.* Association between TNF-alpha polymorphisms and gestational diabetes mellitus: a meta-analysis and trial sequential analysis. *Gynecol Endocrinol* 2021;**37**:506–10. doi:10.1080/09513590.2020.1804549	Less than two randomised clinical trials in the forest plot and TSA
149	Yu L, Shao M, Zhou T, *et al.* Association of CTLA-4 (+49 A/G) polymorphism with susceptibility to autoimmune diseases: a meta-analysis with trial sequential analysis. *Int Immunopharmacol* 2021;**96**:107617. doi:10.1016/j.intimp.2021.107617	Less than two randomised clinical trials in the forest plot and TSA
150	JSK C, Harky A. Trial sequential analysis in forest plots: a clinically oriented approach with real-world example. *J Thorac Cardiovasc Surg* 2021;**162**:167–73. doi:10.1016/j.jtcvs.2020.06.063	Only Trial Sequential Analysis, no forest plot
151	Pergialiotis V, Kotrogianni P, Koutaki D, *et al.* Umbilical cord coiling index for the prediction of adverse pregnancy outcomes: a meta-analysis and sequential analysis. *J Matern neonatal Med* 2019;:1–8. doi:10.1080/14767058.2019.1594187	Less than two randomised clinical trials in the forest plot and TSA
152	Shariff M, Kumar A, Adalja D, *et al.* Inferior vena cava filters reduce symptomatic but not fatal pulmonary emboli after major trauma: a meta-analysis with trial sequential analysis. *Eur J trauma Emerg surgery 2020 MAR 27* Published Online First: 2020. doi:10.1007/s00068-020-01350-z	Both observational and RCT in TSA
153	JSK C. The results are in: cardiovascular benefits of glucagon-like peptide-1 receptor agonists - a trial sequential analysis. *Eur J Prev Cardiol 2020 apr 17* Published Online First: 2020. doi:10.1177/2047487320918722	Only Trial Sequential Analysis, no forest plot
154	YP L, Fan S, Liang Z, *et al.* Phosphodiesterase Type 5 Inhibitors and Risk of Skin Cancers in Men: a Meta-Analysis and Trial Sequential Analysis Involving 7,479,852 Subjects. *world J mens Heal 2020 aug 28* Published Online First: 2020. doi:10.5534/wjmh.200082	Less than two randomised clinical trials in the forest plot and TSA
155	A DC, Pasin L, Boscolo A, *et al.* Trial Sequential Analysis: plain and Simple. *Korean J Anesthesiol 2020 dec 18* Published Online First: 2020. doi:10.4097/kja.20637	Wrong study design
156	YY H, GB J, YF S, *et al.* Association between the pri-miR-26a-1 rs7372209 C>T polymorphism and cancer susceptibility: multivariate analysis and trial sequential analysis. *Aging (Albany NY)* 2020;**12**:19060–72. doi:10.18632/aging.103696	Less than two randomised clinical trials in the forest plot and TSA

157	JSK C, Singh S, Eriksen P, *et al.* Transcatheter Aortic Valve Implantation in Bicuspid Aortic Valve with Aortic Stenosis: a Meta-Analysis and Trial Sequential Analysis. *Brazilian J Cardiovasc surgery 2020 dec 23* Published Online First: 2020. doi:10.21470/1678-9741-2020-0146	Less than two randomised clinical trials in the forest plot and TSA
158	MJ O, Seron P, Buitrago-Garcia D, *et al.* Cardiac rehabilitation effectiveness for coronary artery disease by clinical era: trial sequential analysis. *Eur J Prev Cardiol 2020 dec 01* Published Online First: 2020. doi:10.1093/eurjpc/zwaa110	Only Trial Sequential Analysis, no forest plot
159	Wang J, Liu H, Wang Y, *et al.* The Polymorphisms of Interleukin-12B Gene and Susceptibility to Inflammatory Bowel Diseases: a Meta-analysis and Trial Sequential Analysis. *Immunol Invest* 2021;:1–20. doi:10.1080/08820139.2020.1863981	Less than two randomised clinical trials in the forest plot and TSA
160	Gao S, Xu T, Mao C, *et al.* Lack of Associations between Endoplasmic Reticulum Aminopeptidase 2 Gene Polymorphisms and Ankylosing Spondylitis: a Meta-analysis with Trial Sequential Analysis. *Immunol Invest* 2021;:1–12. doi:10.1080/08820139.2020.1869253	Less than two randomised clinical trials in the forest plot and TSA
161	PB W, Zhang Y, Nie G, *et al.* Association between genetic variants in ZNF365 and inflammatory bowel disease risk in Caucasians: a meta-analysis and trial sequential analysis. *Expert Rev Clin Immunol* 2021;:1–7. doi:10.1080/1744666X.2021.1939012	Less than two randomised clinical trials in the forest plot and TSA
162	Iaculli F, Rengo C, Lodato V, *et al.* Fracture resistance of endodontically-treated maxillary premolars restored with different type of posts and direct composite reconstructions: a systematic review and meta-analysis of in vitro studies. *Dent Mater 2021 jun 17* Published Online First: 2021. doi:10.1016/j.dental.2021.06.007	Less than two randomised clinical trials in the forest plot and TSA
163	Iddawela S, SL M, SA Z, *et al.* Pedicled or skeletonized bilateral internal mammary artery harvesting - a meta- analysis and trial sequential analysis. *Expert Rev Cardiovasc Ther* 2021;:1–8. doi:10.1080/14779072.2021.1939684	Less than two randomised clinical trials in the forest plot and TSA
164	CC Y, Wang J. E-cadherin (CDH1) gene -160C/A polymorphism and the risk of colorectal cancer: a meta-analysis involving 17,291 subjects. *J Gene Med* 2021;:e3370. doi:10.1002/jgm.3370	Less than two randomised clinical trials in the forest plot and TSA
165	Song G, Qiao W, Wang X, *et al.* Association of lung ultrasound score with mortality and severity of COVID-19: a Meta-analysis and Trial Sequential Analysis. *Int J Infect Dis* Published Online First: 2021. doi:10.1016/j.ijid.2021.06.026	Less than two randomised clinical trials in the forest plot and TSA
166	HT W, TB H, ML L, *et al.* Magnesium sulphate treatment decreases the risk of cerebral palsy after preterm birth. *Ugeskr Laeger* 2020;**182**.NS -	Language not in English
167	JSK C, DHH L. Does lysing make life better? A trial sequential analysis. *J Neurol* 2020;**267**:1842–5. doi:10.1007/s00415-020-09801-8	Wrong study design
168	LP F, HL C. Early amniotomy for induction of labor: a trial sequential analysis. *Am J Obstet Gynecol* 2020;**222**:99–100. doi:10.1016/j.ajog.2019.09.028	Wrong study design
169	Trial Sequential Analysis of Drug-Eluting Stents Versus Bare-Metal Stents in Saphenous Vein Graft Intervention. *Am J Cardiol* 2019;**124**:823–4. doi:10.1016/j.amjcard.2019.05.043	Only Trial Sequential Analysis, no forest plot
170	HT W, Brok J, TB H, *et al.* Antenatal magnesium sulphate for the prevention of cerebral palsy in infants born preterm: a double-blind, randomised, placebo-controlled, multi-centre trial. *BJOG* 2020;**127**:1217–25. doi:10.1111/1471-0528.16239	Wrong study design
171	Heneghan C, JK A. Sodium valproate: who knew what and when? Cumulative meta-analysis gives extra insights. *BMJ evidence-based Med* 2019;**24**:127–9. doi:10.1136/bmjebm-2018-111068	Wrong study design

172	KT N, ZX L, Ang E, *et al.* Association of obstructive sleep apnea and postoperative cardiac complications: a systematic review and meta-analysis with trial sequential analysis. *J Clin Anesth* 2020;**62**:109731. doi:10.1016/j.jclinane.2020.109731	Less than two randomised clinical trials in the forest plot and TSA
173	MS L, HZ L, GJ H, *et al.* Trial sequential analysis suggested the potential overestimated effect of carbonic anhydrase inhibitor for respiratory failure and metabolic alkalosis. *Crit Care* 2019;**23**:138. doi:10.1186/s13054-019-2384-y	Wrong study design
174	YJ W. Does oral Nonsteroidal Anti-inflammatory Drugs (NSAIDs) premedication in patients with irreversible pulpitis increase the success rate of inferior alveolar nerve block? *Evid Based Dent* 2019;**20**:20–1. doi:10.1038/s41432-019-0014-9	Wrong study design
175	Han S, Huang T, Wu X, *et al.* Prognostic value of ALDH1 and Nestin in advanced cancer: a systematic meta-analysis with trial sequential analysis. *Ther Adv Med Oncol* 2019;**11**:1758835919830831. doi:10.1177/1758835919830831	Less than two randomised clinical trials in the forest plot and TSA
176	Gartlehner G, Nussbaumer-Streit B, Wagner G, *et al.* Increased risks for random errors are common in outcomes graded as high certainty of evidence. *J Clin Epidemiol* 2019;**106**:50–9. doi:10.1016/j.jclinepi.2018.10.009	Wrong study design
177	HA V, Pedersen J, Faltinsen E, *et al.* Training, executive, attention and motor skills (TEAMS) training versus standard treatment for preschool children with attention deficit hyperactivity disorder: a randomised clinical trial. *BMC Res Notes* 2018;**11**:366. doi:10.1186/s13104-018-3478-3	Wrong study design
178	Cortegiani A, Giarratano A. Untargeted antifungal treatment in nonneutropenic critically Ill patients: should further studies be performed based on trial sequential analysis results? *Antimicrob Agents Chemother* 2018;**62**. doi:10.1128/AAC.00810-18	Wrong study design
179	PM S, Borle A, JK M, *et al.* Evaluation of transversus abdominis plane block for renal transplant recipients - A meta-analysis and trial sequential analysis of published studies. *Saudi J Anaesth* 2018;**12**:261–71. doi:10.4103/sja.SJA_598_17	Less than two randomised clinical trials in the forest plot and TSA
180	Yan Y, AP D, Chen W, *et al.* Cumulative meta-analysis and trial sequential analysis of correlation between hOGG1 Ser326Cys polymorphism and the risk of head and neck squamous cell carcinoma. *Oncotarget* 2018;**9**:13077–87. doi:10.18632/oncotarget.24055	Less than two randomised clinical trials in the forest plot and TSA
181	RK M, SA D, Jawed A, *et al.* Impact of LMP7 (rs2071543) gene polymorphism in increasing cancer risk: evidence from a meta-analysis and trial sequential analysis. *Oncotarget* 2018;**9**:6572–85. doi:10.18632/oncotarget.23547	Less than two randomised clinical trials in the forest plot and TSA
182	Sridharan K, Sivaramakrishnan G. Botulinum Toxin for Refractory Trigeminal Neuralgia: a Trial Sequential Analysis of Randomized Clinical Trials. *J Neurosci Rural Pract* 2018;**9**:3–4. doi:10.4103/jnrp.jnrp_447_17	Only Trial Sequential Analysis, no forest plot
183	Dong F, BH Z, SL Z, *et al.* Association Between SLC30A8 rs13266634 Polymorphism and Risk of T2DM and IGR in Chinese Population: a Systematic Review and Meta-Analysis. *Front Endocrinol (Lausanne)* 2018;**9**:564. doi:10.3389/fendo.2018.00564	Less than two randomised clinical trials in the forest plot and TSA
184	Khan S, SA D, RK M, *et al.* Angiotensin-Converting Enzyme Gene I/D Polymorphism Is Associated With Systemic Lupus Erythematosus Susceptibility: an Updated Meta-Analysis and Trial Sequential Analysis. *Front Physiol* 2018;**9**:1793. doi:10.3389/fphys.2018.01793	Less than two randomised clinical trials in the forest plot and TSA
185	Zhuang Q, Chen Z, Shen J, *et al.* RASSF1A promoter methylation correlates development, progression, and poor cancer-specific survival of renal cell	Less than two randomised clinical trials in the forest plot and TSA

	carcinoma: trial sequential analysis. *Onco Targets Ther* 2019;**12**:119–34. doi:10.2147/OTT.S183142	
186	Han S, Huang T, Wu X, *et al.* Prognostic Value of CD133 and SOX2 in Advanced Cancer. *J Oncol print* 2019;**2019**:3905817. doi:10.1155/2019/3905817	Less than two randomised clinical trials in the forest plot and TSA
187	Han S, Huang T, Li W, *et al.* Prognostic Value of CD44 and Its Isoforms in Advanced Cancer: a Systematic Meta-Analysis With Trial Sequential Analysis. *Front Oncol* 2019;**9**:39. doi:10.3389/fonc.2019.00039	Less than two randomised clinical trials in the forest plot and TSA
188	PH C, HJ J, LJ O-Y, *et al.* Does hydroxychloroquine reduce mortality in patients with COVID-19? A meta-analysis with trial sequential analysis. *Int J Clin Pract* 2021;**75**:e14448. doi:10.1111/ijcp.14448	Wrong study design
189	Safi S, NJ S, SK K, *et al.* Beta-blockers in patients without heart failure after myocardial infarction. *Cochrane Database Syst Rev* Published Online First: 2021. doi:10.1002/14651858.CD012565.pub2	Only forest plot, no Trial Sequential Analysis
190	Wang Y, Tian G, Chen S, *et al.* Myosteatosis reduces overall survival in patients with digestive system malignancies: a meta-analysis with trial sequential analysis. *Nutr Res* 2021;**94**:25–33. doi:10.1016/j.nutres.2021.08.003	Less than two randomised clinical trials in the forest plot and TSA
191	YW H, JW L, TL C. Volumetric Bone Mineral Density Measured by HR-pQCT in Patients with Psoriasis or Psoriatic Arthritis: a Systematic Review and Meta-Analysis with Trial Sequential Analysis. *Healthcare* 2021;**9**. doi:10.3390/healthcare9081056	Less than two randomised clinical trials in the forest plot and TSA
192	Yi H, Wang Y. A meta-analysis of exosome in the treatment of spinal cord injury. *Open Med* 2021;**16**:1043–60. doi:10.1515/med-2021-0304	Wrong study design
193	Guo J, Wang Y, Chen J, *et al.* Systematic review and trial sequential analysis of high-intensity focused ultrasound combined with chemotherapy versus chemotherapy in the treatment of unresectable pancreatic ductal adenocarcinoma. *Int J Hyperth* 2021;**38**:1375–83. doi:10.1080/02656736.2021.1962550	Less than two randomised clinical trials in the forest plot and TSA
194	A DC, Tassone M, Geraldini F, *et al.* Explanation of trial sequential analysis: using a post-hoc analysis of forest plots published in Korean Journal of Anesthesiology. *Korean J Anesthesiol* 2021;**74**:383–93. doi:10.4097/kja.21218	No TSA or forest plot

TSA: Trial Sequential Analysis

Supplemental Table 4. Included studies

Abushanab, D., & Al-Badriyeh, D. (2021). Efficacy and Safety of Ibuprofen Plus Paracetamol in a Fixed-Dose Combination for Acute Postoperative Pain in Adults: Meta-Analysis and a Trial Sequential Analysis. *CNS Drugs*, *35*(1), 105–120. https://doi.org/10.1007/s40263-020-00777-7
Aiolfi, A., Cavalli, M., Micheletto, G., Bruni, P. G., Lombardo, F., Morlacchi, A., Bonitta, G., Campanelli, G., & Bona, D. (2020). Open mesh vs. suture umbilical hernia repair: systematic review and updated trial sequential meta-analysis of randomized controlled trials. *Hernia*, *24*(4), 707–715. https://doi.org/10.1007/s10029-020-02146-1
Aiolfi, A., Cavalli, M., Del Ferraro, S., Manfredini, L., Lombardo, F., Bonitta, G., Bruni, P. G., Panizzo, V., Campanelli, G., & Bona, D. (2021). Total extraperitoneal (TEP) versus laparoscopic transabdominal preperitoneal (TAPP) hernioplasty: systematic review and trial sequential analysis of randomized controlled trials. *Hernia*, *25*(5), 1147–1157. https://doi.org/10.1007/s10029-021-02407-7
Albrecht, E., Grape, S., Frauenknecht, J., Kilchoer, L., & Kirkham, K. R. (2020). Low- versus high-dose intraoperative opioids: A systematic review with meta-analyses and trial sequential analyses. *Acta Anaesthesiologica Scandinavica*, *64*(1), 6–22. https://doi.org/10.1111/aas.13470
Albrecht, E., Wegrzyn, J., Dabetic, A., & El-Boghdadly, K. (2021). The analgesic efficacy of iPACK after knee surgery: A systematic review and meta-analysis with trial sequential analysis. *Journal of Clinical Anesthesia*, *72*(PG-110305), 110305. https://doi.org/10.1016/j.jclinane.2021.110305
Alhajj, M. N., Qi, C. H., Sayed, M. E., Johari, Y., & Ariffin, Z. (2022). Fracture Resistance of Titanium and Fiber Dental Posts: A Systematic Review and Meta-Analysis. *Journal of Prosthodontics*, *31*(5), 374–384. https://doi.org/10.1111/jopr.13428
Al-Rudayni, A. H. M., Gopinath, D., Maharajan, M. K., Veettil, S. K., & Menon, R. K. (2021). Efficacy of photobiomodulation in the treatment of cancer chemotherapy-induced oral mucositis: A meta-analysis with trial sequential analysis. *International Journal of Environmental Research and Public Health*, *18*(14). https://doi.org/10.3390/ijerph18147418
Al-Rudayni, A. H. M., Gopinath, D., Maharajan, M. K., Veettil, S. K., & Menon, R. K. (2021). Efficacy of oral cryotherapy in the prevention of oral mucositis associated with cancer chemotherapy: Systematic review with meta-analysis and trial sequential analysis. *Current Oncology*, *28*(4), 2852–2867. https://doi.org/10.3390/curroncol28040250
Andersen, I. B., Andreassen, M., & Krogh, J. (2021). The effect of dopamine agonists on metabolic variables in adults with type 2 diabetes: A systematic review with meta analysis and trial sequential analysis of randomized clinical trials. *Diabetes, Obesity and Metabolism*, *23*(1), 58–67. https://doi.org/10.1111/dom.14183
Ando, T., Holmes, A. A., Pahuja, M., Javed, A., Briasoulis, A., Telila, T., Takagi, H., Schreiber, T., Afonso, L., Grines, C. L., & Bangalore, S. (2018). Meta-Analysis Comparing Patent Foramen Ovale Closure Versus Medical Therapy to Prevent Recurrent Cryptogenic Stroke. *American Journal of Cardiology*, *121*(5), 649–655. https://doi.org/10.1016/j.amjcard.2017.11.037

Antoniou, G. A., & Antoniou, S. A. (2021). Editor's Choice – Percutaneous Access Does Not Confer Superior Clinical Outcomes Over Cutdown Access for Endovascular Aneurysm Repair: Meta-Analysis and Trial Sequential Analysis of Randomised Controlled Trials. *European Journal of Vascular and Endovascular Surgery*, *61*(3), 383–394. https://doi.org/10.1016/j.ejvs.2020.11.008

Antoniou, G. A., Onwuka, C. C., Antoniou, S. A., & Russell, D. (2019). Meta-analysis and trial sequential analysis of prophylactic negative pressure therapy for groin wounds in vascular surgery. *Journal of Vascular Surgery*, *70*(5), 1700-1710.e6. https://doi.org/10.1016/j.jvs.2019.01.083

Antoniou, S. A., García-Alamino, J. M., Hajibandeh, S., Hajibandeh, S., Weitzendorfer, M., Muysoms, F. E., Granderath, F. A., Chalkiadakis, G. E., Emmanuel, K., Antoniou, G. A., Gioumidou, M., Iliopoulou-Kosmadaki, S., Mathioudaki, M., & Souliotis, K. (2018). Single-incision surgery trocar-site hernia: an updated systematic review meta-analysis with trial sequential analysis by the Minimally Invasive Surgery Synthesis of Interventions Outcomes Network (MISSION). *Surgical Endoscopy*, *32*(1), 14–23. https://doi.org/10.1007/s00464-017-5717-4

Arulkumaran, N., Khpal, M., Tam, K., Baheerathan, A., Corredor, C., & Singer, M. (2020). Effect of Antibiotic Discontinuation Strategies on Mortality and Infectious Complications in Critically Ill Septic Patients: A Meta-Analysis and Trial Sequential Analysis. *Critical Care Medicine*, *48*(5), 757–764. https://doi.org/10.1097/CCM.0000000000004267

Asaad, P., O'Connor, A., Hajibandeh, S., & Hajibandeh, S. (2021). Meta-analysis and trial sequential analysis of randomized evidence comparing general anesthesia vs regional anesthesia for laparoscopic cholecystectomy . *World Journal of Gastrointestinal Endoscopy*, *13*(5), 137–154. https://doi.org/10.4253/wjge.v13.i5.137

Assouline, B., Cools, E., Schorer, R., Kayser, B., Elia, N., & Licker, M. (2021). Preoperative exercise training to prevent postoperative pulmonary complications in adults undergoing major surgery: A systematic review and meta-analysis with trial sequential analysis. *Annals of the American Thoracic Society*, *18*(4), 678–688. https://doi.org/10.1513/AnnalsATS.202002-183OC

Assouline, B., Faivre, A., Verissimo, T., Sangla, F., Berchtold, L., Giraud, R., Bendjelid, K., Sgardello, S., Elia, N., Pugin, J., De Seigneux, S., & Legouis, D. (2021). Thiamine, Ascorbic Acid, and Hydrocortisone As a Metabolic Resuscitation Cocktail in Sepsis: A Meta-Analysis of Randomized Controlled Trials With Trial Sequential Analysis. *Critical Care Medicine*, *49*(12), 2112–2120. https://doi.org/10.1097/CCM.0000000000005262

Baandrup, L., Ebdrup, B. H., Rasmussen, J. O., Lindschou, J., Gluud, C., & Glenthøj, B. Y. (2018). Pharmacological interventions for benzodiazepine discontinuation in chronic benzodiazepine users. *Cochrane Database of Systematic Reviews*, *2018*(3), CD011481. https://doi.org/10.1002/14651858.CD011481.pub2

Bajaj, N. S., Gupta, K., Gharpure, N., Pate, M., Chopra, L., Kalra, R., & Prabhu, S. D. (2020). Effect of immunomodulation on cardiac remodelling and outcomes in heart failure: a quantitative synthesis of the literature. *ESC Heart Failure*, *7*(3), 1319–1330. https://doi.org/10.1002/ehf2.12681

Bajaj, N. S., Vaduganathan, M., Qamar, A., Gupta, K., Gupta, A., Golwala, H., Butler, J., Goldhaber, S. Z., & Mehra, M. R. (2019). Extended prophylaxis for venous thromboembolism after hospitalization for medical

illness: A trial sequential and cumulative meta-analysis. *PLoS Medicine*, *16*(4), e1002797. https://doi.org/10.1371/journal.pmed.1002797

Bakker, W. J., Aufenacker, T. J., Boschman, J. S., & Burgmans, J. P. J. (2021). Heavyweight Mesh Is Superior to Lightweight Mesh in Laparo-endoscopic Inguinal Hernia Repair: A Meta-analysis and Trial Sequential Analysis of Randomized Controlled Trials. *Annals of Surgery*, *273*(5), 890–899. https://doi.org/10.1097/SLA.0000000000003831

Ball, L., Serpa Neto, A., Trifiletti, V., Mandelli, M., Firpo, I., Robba, C., Gama de Abreu, M., Schultz, M. J., Patroniti, N., Rocco, P. R. M., & Pelosi, P. (2020). Effects of higher PEEP and recruitment manoeuvres on mortality in patients with ARDS: a systematic review, meta-analysis, meta-regression and trial sequential analysis of randomized controlled trials. *Intensive Care Medicine Experimental* , *8*(Suppl 1 PG-39), 39. https://doi.org/10.1186/s40635-020-00322-2

Bangalore, S., Maron, D. J., Stone, G. W., & Hochman, J. S. (2020). Routine Revascularization Versus Initial Medical Therapy for Stable Ischemic Heart Disease: A Systematic Review and Meta-Analysis of Randomized Trials. *Circulation*, *142*(9), 841–857. https://doi.org/10.1161/CIRCULATIONAHA.120.048194

Barbarawi, M., Al-abdouh, A., Barbarawi, O., Lakshman, H., Al kasasbeh, M., & Chen, K. (2022). SGLT2 inhibitors and cardiovascular and renal outcomes: a meta-analysis and trial sequential analysis. *Heart Failure Reviews*, *27*(3), 951–960. https://doi.org/10.1007/s10741-021-10083-z

Barbarawi, M., Alabdouh, A., Barbarawi, O., Lakshman, H., Alkasasbeh, M., Rizk, F., Bachuwa, G., & Alkotob, M. L. (2020). Targeted Temperature Management in Cardiac Arrest Patients With an Initial Non-Shockable Rhythm: A Systematic Review and Meta-Analysis. *Shock (Augusta, Ga.)*, *54*(5), 623–630. https://doi.org/10.1097/SHK.0000000000001550

Barbateskovic, M., Krauss, S. R., Collet, M. O., Andersen-Ranberg, N. C., Mathiesen, O., Jakobsen, J. C., Perner, A., & Wetterslev, J. (2020). Haloperidol for the treatment of delirium in critically ill patients: A systematic review with meta-analysis and Trial Sequential Analysis. *Acta Anaesthesiologica Scandinavica*, *64*(2), 254–266. https://doi.org/10.1111/aas.13501

Barbateskovic, M., Marker, S., Granholm, A., Anthon, C. T., Krag, M., Jakobsen, J. C., Perner, A., Wetterslev, J., & Møller, M. H. (2019). Stress ulcer prophylaxis with proton pump inhibitors or histamin-2 receptor antagonists in adult intensive care patients: a systematic review with meta-analysis and trial sequential analysis. *Intensive Care Medicine*, *45*(2), 143–158. https://doi.org/10.1007/s00134-019-05526-z

Barbateskovic, M., Schjørring, O. L., Krauss, S. R., Meyhoff, C. S., Jakobsen, J. C., Rasmussen, B. S., Perner, A., & Wetterslev, J. (2021). Higher vs Lower Oxygenation Strategies in Acutely Ill Adults: A Systematic Review With Meta-Analysis and Trial Sequential Analysis. *Chest*, *159*(1), 154–173. https://doi.org/10.1016/j.chest.2020.07.015

Bellos, I., Iliopoulos, D. C., & Perrea, D. N. (2019). Allopurinol Administration for the Prevention of Contrast-Induced Nephropathy: A Network Meta-analysis with Trial Sequential Analysis. *Journal of Cardiovascular Pharmacology*, *73*(5), 307–315. https://doi.org/10.1097/FJC.0000000000000663

Besen, B. A. M. P., Romano, T. G., Mendes, P. V., Gallo, C. A., Zampieri, F. G., Nassar, A. P., & Park, M. (2019). Early Versus Late Initiation of Renal Replacement Therapy in Critically Ill Patients: Systematic Review and

Meta-Analysis. *Journal of Intensive Care Medicine*, *34*(9), 714–722. https://doi.org/10.1177/0885066617710914

Bhattacharjee, D., Doleman, B., Lund, J., & Williams, J. (2019). Mirtazapine for Postoperative Nausea and Vomiting: Systematic Review, Meta-analysis, and Trial Sequential Analysis. *Journal of Perianesthesia Nursing*, *34*(4), 680–690. https://doi.org/10.1016/j.jopan.2018.11.006

Bjelakovic, M., Nikolova, D., Bjelakovic, G., & Gluud, C. (2021). Vitamin D supplementation for chronic liver diseases in adults. *Cochrane Database of Systematic Reviews*, *2021*(8). https://doi.org/10.1002/14651858.CD011564.pub3

Blanco-Silvente, L., Castells, X., Garre-Olmo, J., Vilalta-Franch, J., Saez, M., Barceló, M. A., & Capellà, D. (2019). Study of the strength of the evidence and the redundancy of the research on pharmacological treatment for Alzheimer's disease: a cumulative meta-analysis and trial sequential analysis. *European Journal of Clinical Pharmacology*, *75*(12), 1659–1667. https://doi.org/10.1007/s00228-019-02742-w

Bocchile, R. L. R., Cazati, D. C., Timenetsky, K. T., & Neto, A. S. (2018). The effects of high-flow nasal cannula on intubation and re-intubation in critically ill patients: A systematic review, meta-analysis and trial sequential analysis. *Revista Brasileira de Terapia Intensiva*, *30*(4), 487–495. https://doi.org/10.5935/0103-507X.20180070

Bocskai, T., Kovács, M., Szakács, Z., Gede, N., Hegyi, P., Varga, G., Pap, I., Tóth, I., Révész, P., Szanyi, I., Németh, A., Gerlinger, I., Karádi, K., & Lujber, L. (2020). Is the bispectral index monitoring protective against postoperative cognitive decline? A systematic review with meta-analysis. *PLoS ONE*, *15*(2), e0229018. https://doi.org/10.1371/journal.pone.0229018

Bohara, S., Gaonkar, V. B., Garg, K., Rajpal, P. M. S., Singh, P. K., Singh, M., Suri, A., Chandra, P. S., & Kale, S. S. (2021). Effect of statins on functional outcome and mortality following aneurysmal subarachnoid hemorrhage – Results of a meta-analysis, metaregression and trial sequential analysis. *Clinical Neurology and Neurosurgery*, *207*(PG-106787), 106787. https://doi.org/10.1016/j.clineuro.2021.106787

Bohara, S., Garg, K., Singh Rajpal, P. M., & Kasliwal, M. (2021). Role of Cilostazol in Prevention of Vasospasm After Aneurysmal Subarachnoid Hemorrhage–A Systematic Review, Meta-Analysis, and Trial Sequential Analysis. *World Neurosurgery*, *150*(PG-161-170), 161–170. https://doi.org/10.1016/j.wneu.2021.02.069

Bolland, M. J., Grey, A., & Avenell, A. (2018). Effects of vitamin D supplementation on musculoskeletal health: a systematic review, meta-analysis, and trial sequential analysis. *The Lancet Diabetes and Endocrinology*, *6*(11), 847–858. https://doi.org/10.1016/S2213-8587(18)30265-1

Brand, M., Prodehl, L., & Ede, C. J. (2018). Surgical portosystemic shunts versus transjugular intrahepatic portosystemic shunt for variceal haemorrhage in people with cirrhosis. *Cochrane Database of Systematic Reviews*, *2018*(10), CD001023. https://doi.org/10.1002/14651858.CD001023.pub3

Bryant, A., Lawrie, T. A., Dowswell, T., Fordham, E. J., Mitchell, S., Hill, S. R., & Tham, T. C. (2021). Ivermectin for Prevention and Treatment of COVID-19 Infection: A Systematic Review, Meta-analysis, and Trial Sequential Analysis to Inform Clinical Guidelines. *American Journal of Therapeutics*, *28*(4), E434–E460. https://doi.org/10.1097/MJT.0000000000001402

Buggeskov, K. B., Grønlykke, L., Risom, E. C., Wei, M. L., & Wetterslev, J. (2018). Pulmonary artery perfusion versus no perfusion during cardiopulmonary bypass for open heart surgery in adults. *Cochrane Database of Systematic Reviews*, *2018*(2), CD011098. https://doi.org/10.1002/14651858.CD011098.pub2

Bullen, N. L., Hajibandeh, S., Hajibandeh, S., Smart, N. J., & Antoniou, S. A. (2021). Suture fixation versus self-gripping mesh for open inguinal hernia repair: a systematic review with meta-analysis and trial sequential analysis. *Surgical Endoscopy*, *35*(6), 2480–2492. https://doi.org/10.1007/s00464-020-07658-6

Bullen, N. L., Massey, L. H., Antoniou, S. A., Smart, N. J., & Fortelny, R. H. (2019). Open versus laparoscopic mesh repair of primary unilateral uncomplicated inguinal hernia: a systematic review with meta-analysis and trial sequential analysis. *Hernia*, *23*(3), 461–472. https://doi.org/10.1007/s10029-019-01989-7

Burcharth, J., Falkenberg, A., Schack, A., Ekeloef, S., & Gögenur, I. (2021). The effects of early enteral nutrition on mortality after major emergency abdominal surgery: A systematic review and meta-analysis with Trial Sequential Analysis. *Clinical Nutrition*, *40*(4), 1604–1612. https://doi.org/10.1016/j.clnu.2021.02.050

Butler, E., Møller, M. H., Cook, O., Granholm, A., Penketh, J., Rygård, S. L., Aneman, A., & Perner, A. (2019). The effect of systemic corticosteroids on the incidence of gastrointestinal bleeding in critically ill adults: a systematic review with meta-analysis. *Intensive Care Medicine*, *45*(11), 1540–1549. https://doi.org/10.1007/s00134-019-05754-3

Buzquurz, F., Bojesen, R. D., Grube, C., Madsen, M. T., & Gögenur, I. (2020). Impact of oral preoperative and perioperative immunonutrition on postoperative infection and mortality in patients undergoing cancer surgery: systematic review and meta-analysis with trial sequential analysis. *BJS Open*, *4*(5), 764–775. https://doi.org/10.1002/bjs5.50314

Cabiddu, M. F., Russi, A., Appolloni, L., Mengato, D., & Chiumente, M. (2022). Omega-3 for the prevention of cardiovascular diseases: Meta-analysis and trial-sequential analysis. *European Journal of Hospital Pharmacy*, *29*(3), 134–138. https://doi.org/10.1136/ejhpharm-2020-002207

Cabrini, L., Baiardo Redaelli, M., Filippini, M., Fominskiy, E., Pasin, L., Pintaudi, M., Plumari, V. P., Putzu, A., Votta, C. D., Pallanch, O., Ball, L., Landoni, G., Pelosi, P., & Zangrillo, A. (2020). Tracheal intubation in patients at risk for cervical spinal cord injury: A systematic review. *Acta Anaesthesiologica Scandinavica*, *64*(4), 443–454. https://doi.org/10.1111/aas.13532

Caldeira, D., David, C., Costa, J., Ferreira, J. J., & Pinto, F. J. (2018). Non-Vitamin K antagonist oral anticoagulants in patients with atrial fibrillation and valvular heart disease: Systematic review and meta-analysis. *European Heart Journal - Cardiovascular Pharmacotherapy*, *4*(2), 111–118. https://doi.org/10.1093/ehjcvp/pvx028

Caldeira, D., Nunes-Ferreira, A., Rodrigues, R., Vicente, E., Pinto, F. J., & Ferreira, J. J. (2019). Non-vitamin K antagonist oral anticoagulants in elderly patients with atrial fibrillation: A systematic review with meta-analysis and trial sequential analysis. *Archives of Gerontology and Geriatrics*, *81*(PG-209-214), 209–214. https://doi.org/10.1016/j.archger.2018.12.013

Canullo, L., Laino, L., Longo, F., Filetici, P., D'Onofrio, I., & Troiano, G. (2020). Does Chlorhexidine Prevent Complications in Extractive, Periodontal, and Implant Surgery? A Systematic Review and Meta-analysis with

Trial Sequential Analysis. *The International Journal of Oral & Maxillofacial Implants*, *35*(6), 1149–1158. https://doi.org/10.11607/jomi.8216

Canullo, L., Pesce, P., Patini, R., Antonacci, D., & Tommasato, G. (2020). What Are the Effects of Different Abutment Morphologies on Peri-implant Hard and Soft Tissue Behavior? A Systematic Review and Meta-Analysis. *The International Journal of Prosthodontics*, *33*(3), 297–306. https://doi.org/10.11607/ijp.6577

Canullo, L., Troiano, G., Sbricoli, L., Guazzo, R., Laino, L., Caiazzo, A., & Pesce, P. (2020). The Use of Antibiotics in Implant Therapy: A Systematic Review and Meta-Analysis with Trial Sequential Analysis on Early Implant Failure. *The International Journal of Oral & Maxillofacial Implants*, *35*(3), 485–494. https://doi.org/10.11607/jomi.7995

Cao, H. J., Liang, S. B., Zhou, W., Wu, J. R., & Zhang, C. L. (2019). Evaluation of the adjunctive effect of Xing Nao Jing Injection for viral encephalitis: A systematic review and meta-analysis of randomized controlled trials. *Medicine (United States)*, *98*(15), e15181. https://doi.org/10.1097/MD.0000000000015181

Cao, H. J., Yu, M. L., Wang, L. Q., Fei, Y. T., Xu, H., & Liu, J. P. (2019). Acupuncture for primary insomnia: An updated systematic review of randomized controlled trials. *Journal of Alternative and Complementary Medicine*, *25*(5), 451–474. https://doi.org/10.1089/acm.2018.0046

Capodanno, D., Maio, M. Di, Greco, A., Bhatt, D. L., Gibson, C. M., Goette, A., Lopes, R. D., Mehran, R., Vranckx, P., & Angiolillo, D. J. (2020). Safety and efficacy of double antithrombotic therapy with non–vitamin k antagonist oral anticoagulants in patients with atrial fibrillation undergoing percutaneous coronary intervention: A systematic review and meta-analysis. *Journal of the American Heart Association*, *9*(16), e017212. https://doi.org/10.1161/JAHA.120.017212

Cecoro, G., Piccirillo, A., Martuscelli, G., Del Fabbro, M., Annunziata, M., & Guida, L. (2021). Efficacy of locally delivered statins as an adjunct to scaling and root planing in the treatment of periodontitis: A systematic review and meta-analysis. *European Review for Medical and Pharmacological Sciences*, *25*(18), 5737–5754. https://doi.org/10.26355/eurrev_202109_26792

Cecoro, G., Piccirillo, A., Martuscelli, G., Del Fabbro, M., Annunziata, M., & Guida, L. (2021). Efficacy of locally delivered statins as an adjunct to scaling and root planing in the treatment of periodontitis: A systematic review and meta-analysis. *European Review for Medical and Pharmacological Sciences*, *25*(18), 5737–5754. https://doi.org/10.26355/eurrev_202109_26792

Chan, J. S. K., Kot, T. K. M., NG, M., & Harky, A. (2020). Continuous Infusion Versus Intermittent Boluses of Furosemide in Acute Heart Failure: A Systematic Review and Meta-Analysis. *Journal of Cardiac Failure*, *26*(9), 786–793. https://doi.org/10.1016/j.cardfail.2019.11.013

Chang, C. Y., Chen, H. A., Chien, Y. J., & Wu, M. Y. (2021). Attenuation of the increase in intraocular pressure with dexmedetomidine: Systematic review with meta-analysis and trial sequential analysis. *Journal of Clinical Anesthesia*, *68*(PG-110065), 110065. https://doi.org/10.1016/j.jclinane.2020.110065

Chang, C. Y., Chien, Y. J., & Wu, M. Y. (2020). Attenuation of increased intraocular pressure with propofol anesthesia: A systematic review with meta-analysis and trial sequential analysis. *Journal of Advanced Research*, *24*(PG-223-238), 223–238. https://doi.org/10.1016/j.jare.2020.02.008

Chang, Y. J., Hung, K. C., Chen, I. W., Kuo, C. L., Teng, I. C., Lin, M. C., Yew, M., Liao, S. W., Wu, C. Y., Yu, C. H., Lan, K. M., & Sun, C. K. (2021). Efficacy of greater occipital nerve block for pain relief in patients with postdural puncture headache: A meta-analysis. *Medicine (United States)*, *100*(51), E28438. https://doi.org/10.1097/MD.0000000000028438

Chang, Y. M., Liang, C. M., Weng, T. H., Chien, K. H., & Lee, C. H. (2021). Mitomycin C for the prevention of corneal haze in photorefractive keratectomy: a meta-analysis and trial sequential analysis. *Acta Ophthalmologica*, *99*(6), 652–662. https://doi.org/10.1111/aos.14704

Chen, A., Elia, N., Dunaiceva, J., Rudiger, A., Walder, B., & Bollen Pinto, B. (2020). Effect of ivabradine on major adverse cardiovascular events and mortality in critically ill patients: a systematic review and meta-analyses of randomised controlled trials with trial sequential analyses. *British Journal of Anaesthesia*, *124*(6), 726–738. https://doi.org/10.1016/j.bja.2020.01.027

Chen, C. H., Huang, C. Y., Chang, C. Y., & Cheng, Y. F. (2020). Efficacy of low-level laser therapy for tinnitus: A systematic review with meta-analysis and trial sequential analysis. *Brain Sciences*, *10*(12), 1–16. https://doi.org/10.3390/brainsci10120931

Chen, C. H., Huang, C. Y., Lin, H. Y. H., Wang, M. C., Chang, C. Y., & Cheng, Y. F. (2021). Association of Sodium Thiosulfate with Risk of Ototoxic Effects from Platinum-Based Chemotherapy: A Systematic Review and Meta-analysis. *JAMA Network Open*, *4*(8 PG-e2118895), e2118895. https://doi.org/10.1001/jamanetworkopen.2021.18895

Chen, H., Yao, X., Li, T., Lam, C. W. K., Zhang, R., Zhang, H., Wang, J., Zhang, W., Leung, E. L. H., & Wu, Q. (2020). Compound Kushen injection combined with platinumbased chemotherapy for stage III/IV non-small cell lung cancer: A meta-analysis of 37 RCTs following the PRISMA guidelines. *Journal of Cancer*, *11*(7), 1883–1898. https://doi.org/10.7150/jca.40267

Chen, P. C., Lai, C. H., Fang, C. J., Lai, P. C., & Huang, Y. T. (2022). Intravenous Infusion of Lidocaine for Bowel Function Recovery After Major Colorectal Surgery: A Critical Appraisal Through Updated Meta-Analysis, Trial Sequential Analysis, Certainty of Evidence, and Meta-Regression. *Frontiers in Medicine*, *8*(PG-759215), 759215. https://doi.org/10.3389/fmed.2021.759215

Chen, Q. H., Wang, H. L., Liu, L., Shao, J., Yu, J., & Zheng, R. Q. (2018). Effects of restrictive red blood cell transfusion on the prognoses of adult patients undergoing cardiac surgery: A meta-analysis of randomized controlled trials. *Critical Care*, *22*(1), 142. https://doi.org/10.1186/s13054-018-2062-5

Chen, Q., An, R., Zhou, J., & Yang, B. (2018). Clinical analgesic efficacy of dexamethasone as a local anesthetic adjuvant for transversus abdominis plane (TAP) block: A meta-analysis. *PLoS ONE*, *13*(6), e0198923. https://doi.org/10.1371/journal.pone.0198923

Chen, Z., Chen, R., Zheng, D., Su, Y., Wen, S., Guo, H., Ye, Z., Deng, Y., Liu, G., Zuo, L., Wei, X., & Hou, Y. (2020). Efficacy and safety of haloperidol for delirium prevention in adult patients: An updated meta-analysis with trial sequential analysis of randomized controlled trials. *Journal of Clinical Anesthesia*, *61*(PG-109623), 109623. https://doi.org/10.1016/j.jclinane.2019.09.017

Cheng, M., Hu, J., Zhao, Y., Jiang, J., Qi, R., Chen, S., Li, Y., Zheng, H., Liu, R., Guo, Q., Zhang, X., Qin, Y., & Hua, B. (2021). Efficacy and Safety of Astragalus-Containing Traditional Chinese Medicine Combined With

Platinum-Based Chemotherapy in Advanced Gastric Cancer: A Systematic Review and Meta-Analysis. *Frontiers in Oncology*, *11*(PG-632168), 632168. https://doi.org/10.3389/fonc.2021.632168

Cheng, Z., Dong, S., Bi, D., Wang, Y., Dai, Y., & Zhang, X. (2021). Early Versus Late Preventive Ileostomy Closure Following Colorectal Surgery: Systematic Review and Meta-analysis With Trial Sequential Analysis of Randomized Controlled Trials. *Diseases of the Colon and Rectum*, *64*(1), 128–137. https://doi.org/10.1097/DCR.0000000000001839

Chien, Y. J., Chang, C. Y., Wu, M. Y., Chen, C. H., Horng, Y. S., & Wu, H. C. (2021). Effects of curcumin on glycemic control and lipid profile in polycystic ovary syndrome: Systematic review with meta-analysis and trial sequential analysis. *Nutrients*, *13*(2), 1–14. https://doi.org/10.3390/nu13020684

Chiew, A. L., Gluud, C., Brok, J., & Buckley, N. A. (2018). Interventions for paracetamol (acetaminophen) overdose. *Cochrane Database of Systematic Reviews*, *2018*(2), CD003328. https://doi.org/10.1002/14651858.CD003328.pub3

Ching, S. M., Mokshashri, N. R., Kannan, M. M., Lee, K. W., Sallahuddin, N. A., Ng, J. X., Wong, J. L., Devaraj, N. K., Hoo, F. K., Loo, Y. S., & Veettil, S. K. (2021). Effects of qigong on systolic and diastolic blood pressure lowering: a systematic review with meta-analysis and trial sequential analysis. *BMC Complementary Medicine and Therapies*, *21*(1), 8. https://doi.org/10.1186/s12906-020-03172-3

Chiu, H. T., Jhou, H. J., Chen, P. H., Lee, C. H., & Lin, C. Y. (2022). Comparing Single- and Dual-Antiplatelet Therapies After Transcatheter Aortic Valve Implantation. *Annals of Thoracic Surgery*, *114*(5), 1951–1964. https://doi.org/10.1016/j.athoracsur.2021.09.048

Cho, N., Kang, R. S., McCartney, C. J. L., Pawa, A., Costache, I., Rose, P., & Abdallah, F. W. (2020). Analgesic benefits and clinical role of the posterior suprascapular nerve block in shoulder surgery: a systematic review, meta-analysis and trial sequential analysis. *Anaesthesia*, *75*(3), 386–394. https://doi.org/10.1111/anae.14858

Cho, S. S., Kim, S. E., Kim, H. C., Kim, W. J., & Jeon, J. P. (2019). Clazosentan for Aneurysmal Subarachnoid Hemorrhage: An Updated Meta-Analysis with Trial Sequential Analysis. *World Neurosurgery*, *123*(PG-418-424.e3), 418-424.e3. https://doi.org/10.1016/j.wneu.2018.10.213

Choi, G. J., Kim, Y. Il, Koo, Y. H., Oh, H. C., & Kang, H. (2021). Perioperative magnesium for postoperative analgesia: An umbrella review of systematic reviews and updated meta-analysis of randomized controlled trials. *Journal of Personalized Medicine*, *11*(12). https://doi.org/10.3390/jpm11121273

Chu, D. K., Kim, L. H. Y., Young, P. J., Zamiri, N., Almenawer, S. A., Jaeschke, R., Szczeklik, W., Schünemann, H. J., Neary, J. D., & Alhazzani, W. (2018). Mortality and morbidity in acutely ill adults treated with liberal versus conservative oxygen therapy (IOTA): a systematic review and meta-analysis. *The Lancet*, *391*(10131), 1693–1705. https://doi.org/10.1016/S0140-6736(18)30479-3

Chu, D. K., Wood, R. A., French, S., Fiocchi, A., Jordana, M., Waserman, S., Brożek, J. L., & Schünemann, H. J. (2019). Oral immunotherapy for peanut allergy (PACE): a systematic review and meta-analysis of efficacy and safety. *The Lancet*, *393*(10187), 2222–2232. https://doi.org/10.1016/S0140-6736(19)30420-9

Cicciù, M., Stacchi, C., Fiorillo, L., Cervino, G., Troiano, G., Vercellotti, T., Herford, A. S., Galindo-Moreno, P., & Di Lenarda, R. (2021). Piezoelectric bone surgery for impacted lower third molar extraction compared with

conventional rotary instruments: a systematic review, meta-analysis, and trial sequential analysis. *International Journal of Oral and Maxillofacial Surgery*, *50*(1), 121–131. https://doi.org/10.1016/j.ijom.2020.03.008

Colombo, C., Salvioli, S., Gianola, S., Castellini, G., & Testa, M. (2020). Traction therapy for cervical radicular syndrome is statistically significant but not clinically relevant for pain relief. A systematic literature review with meta-analysis and trial sequential analysis. *Journal of Clinical Medicine*, *9*(11), 1–15. https://doi.org/10.3390/jcm9113389

Comerlato, P. H., Stefani, J., & Viana, L. V. (2021). Mortality and overall and specific infection complication rates in patients who receive parenteral nutrition: Systematic review and meta-analysis with trial sequential analysis. *American Journal of Clinical Nutrition*, *114*(4), 1535–1545. https://doi.org/10.1093/ajcn/nqab218

Cui, J., Tang, D., Chen, Z., & Liu, G. (2018). Impact of Early versus Late Initiation of Renal Replacement Therapy in Patients with Cardiac Surgery-Associated Acute Kidney Injury: Meta-Analysis with Trial Sequential Analysis of Randomized Controlled Trials. *BioMed Research International*, *2018*(PG-6942829), 6942829. https://doi.org/10.1155/2018/6942829

Cui, J., Wei, X., Lv, H., Li, Y., Li, P., Chen, Z., & Liu, G. (2019). The clinical efficacy of intravenous IgM-enriched immunoglobulin (pentaglobin) in sepsis or septic shock: a meta-analysis with trial sequential analysis. *Annals of Intensive Care*, *9*(1), 27. https://doi.org/10.1186/s13613-019-0501-3

De Cassai, A., Boscolo, A., Geraldini, F., Zarantonello, F., Pettenuzzo, T., Pasin, L., Iuzzolino, M., Rossini, N., Pesenti, E., Zecchino, G., Sella, N., Munari, M., & Navalesi, P. (2021). Effect of dexmedetomidine on hemodynamic responses to tracheal intubation: A meta-analysis with meta-regression and trial sequential analysis. *Journal of Clinical Anesthesia*, *72*(PG-110287), 110287. https://doi.org/10.1016/j.jclinane.2021.110287

Deliwala, S. S., Hamid, K., Barbarawi, M., Lakshman, H., Zayed, Y., Kandel, P., Malladi, S., Singh, A., Bachuwa, G., Gurvits, G. E., & Chawla, S. (2021). Artificial intelligence (AI) real-time detection vs. routine colonoscopy for colorectal neoplasia: a meta-analysis and trial sequential analysis. *International Journal of Colorectal Disease*, *36*(11), 2291–2303. https://doi.org/10.1007/s00384-021-03929-3

Deliwala, S. S., Hamid, K., Goyal, H., Ponnapalli, A., Zayed, Y., Bala, A., Lakshman, H., Malladi, S., Jones, S., Santana, M., Leon, B., An, M. T., & Chawla, S. (2022). Proton Pump Inhibitors Versus Histamine-2-Receptor Antagonists for Stress Ulcer Prophylaxis in Critically Ill Patients: A Meta-analysis and Trial Sequential Analysis. *Journal of Clinical Gastroenterology*, *56*(3), 204–217. https://doi.org/10.1097/MCG.0000000000001562

DE-MIGUEL-BALSA, E., ESTEVAN-ORTEGA, R., SEMPERE-SELVA, M. T., LATOUR-PÉREZ, J., BAEZA-ROMÁN, A., MOYA-MARTINEZ, A., & RAMOS-RINCON, J. M. (2021). Can we still consider treatment with colchicine effective in SARS-COV-2 infection? Systematic review, meta-analysis, and trial sequential analysis. *European Review for Medical and Pharmacological Sciences*, *25*(22), 7151–7161. https://doi.org/10.26355/eurrev_202111_27269

Deng, J., Wang, G., Li, J., Wang, S., Li, M., Yin, X., Zhang, L., & Tang, P. (2021). A systematic review and meta-analysis comparing arthroplasty and internal fixation in the treatment of elderly displaced femoral neck

fractures. *OTA International: The Open Access Journal of Orthopaedic Trauma*, *4*(1), e087. https://doi.org/10.1097/oi9.0000000000000087

Desai, N., El-Boghdadly, K., & Albrecht, E. (2021). Epidural vs. transversus abdominis plane block for abdominal surgery – a systematic review, meta-analysis and trial sequential analysis. *Anaesthesia*, *76*(1), 101–117. https://doi.org/10.1111/anae.15068

Devaraj, N. K., Suppiah, S., Veettil, S. K., Ching, S. M., Lee, K. W., Menon, R. K., Soo, M. J., Deuraseh, I., Hoo, F. K., & Sivaratnam, D. (2019). The effects of probiotic supplementation on the incidence of diarrhea in cancer patients receiving radiation therapy: A systematic review with meta-analysis and trial sequential analysis of randomized controlled trials. *Nutrients*, *11*(12). https://doi.org/10.3390/nu11122886

DInges, H. C., Wiesmann, T., Otremba, B., Wulf, H., Eberhart, L. H., & Schubert, A. K. (2021). The analgesic efficacy of liposomal bupivacaine compared with bupivacaine hydrochloride for the prevention of postoperative pain: A systematic review and meta-analysis with trial sequential analysis. *Regional Anesthesia and Pain Medicine*, *46*(6), 490–498. https://doi.org/10.1136/rapm-2020-102427

Dohos, D., Hanák, L., Szakács, Z., Kiss, S., Párniczky, A., Erőss, B., Pázmány, P., Hegyi, P., & Sarlós, P. (2021). Systematic review with meta-analysis: the effects of immunomodulator or biological withdrawal from mono- or combination therapy in inflammatory bowel disease. *Alimentary Pharmacology and Therapeutics*, *53*(2), 220–233. https://doi.org/10.1111/apt.16182

Doshi, R., Kumar, A., Thakkar, S., Shariff, M., Adalja, D., Doshi, A., Taha, M., Gupta, R., Desai, R., Shah, J., & Gullapalli, N. (2020). Meta-analysis Comparing Combined Use of Eicosapentaenoic Acid and Statin to Statin Alone. *American Journal of Cardiology*, *125*(2), 198–204. https://doi.org/10.1016/j.amjcard.2019.10.009

Duan, X., Coburn, M., Rossaint, R., Sanders, R. D., Waesberghe, J. V., & Kowark, A. (2018). Efficacy of perioperative dexmedetomidine on postoperative delirium: systematic review and meta-analysis with trial sequential analysis of randomised controlled trials. *British Journal of Anaesthesia*, *121*(2), 384–397. https://doi.org/10.1016/j.bja.2018.04.046

Duan, Z. X., Chen, D. X., Yang, B. Z., & Zhang, X. Q. (2021). Transfusion Strategies for Pediatric Cardiac Surgery: A Meta-Analysis and Trial Sequential Analysis. *Pediatric Cardiology*, *42*(6), 1241–1251. https://doi.org/10.1007/s00246-021-02644-8

Eck, R. J., Bult, W., Wetterslev, J., Gans, R. O. B., Meijer, K., Keus, F., & Van Der Horst, I. C. C. (2019). Intermediate Dose Low-Molecular-Weight Heparin for Thrombosis Prophylaxis: Systematic Review with Meta-Analysis and Trial Sequential Analysis. *Seminars in Thrombosis and Hemostasis*, *45*(8), 810–824. https://doi.org/10.1055/s-0039-1696965

Eck, R. J., Bult, W., Wetterslev, J., Gans, R. O. B., Meijer, K., van der Horst, I. C. C., & Keus, F. (2019). Low dose low-molecular-weight heparin for thrombosis prophylaxis: Systematic review with meta-analysis and trial sequential analysis. *Journal of Clinical Medicine*, *8*(12). https://doi.org/10.3390/jcm8122039

Ede, C. J., Nikolova, D., & Brand, M. (2018). Surgical portosystemic shunts versus devascularisation procedures for prevention of variceal rebleeding in people with hepatosplenic schistosomiasis. *Cochrane Database of Systematic Reviews*, *2018*(8), CD011717. https://doi.org/10.1002/14651858.CD011717.pub2

Elgendy, I. Y., Gad, M., Elbadawi, A., Elgendy, A. Y., & Mahmoud, A. N. (2020). Is complete revascularization for multivessel disease during primary percutaneous coronary intervention associated with lower cardiovascular mortality? An updated meta-analysis and trial sequential of randomized trials. *European Heart Journal - Quality of Care and Clinical Outcomes*, *6*(4), 341–342. https://doi.org/10.1093/ehjqcco/qcz067

Elgendy, I. Y., Mahmoud, A. N., Gad, M., Elgendy, A. Y., & Bhatt, D. L. (2020). Long-Term Outcomes With Drug-Eluting Stents or Coronary Artery Bypass Surgery for Unprotected Left Main Coronary Disease: A Meta-Analysis and Trial Sequential Analysis of Randomized Trials. *American Journal of Cardiology*, *126*(PG-111-112), 111–112. https://doi.org/10.1016/j.amjcard.2020.04.005

Ellis, L. R., Zulfiqar, S., Holmes, M., Marshall, L., Dye, L., & Boesch, C. (2022). A systematic review and meta-analysis of the effects of Hibiscus sabdariffa on blood pressure and cardiometabolic markers. *Nutrition Reviews*, *80*(6), 1723–1737. https://doi.org/10.1093/nutrit/nuab104

Eltair, M., Hajibandeh, S., Nuno, A., Abdullah, K. H., Alkaili-Alyamani, A., Aslam, M. I., Sinha, A., & Agarwal, T. (2020). Meta-analysis and trial sequential analysis of robotic versus laparoscopic total mesorectal excision in management of rectal cancer. *International Journal of Colorectal Disease*, *35*(8), 1423–1438. https://doi.org/10.1007/s00384-020-03655-2

Erba, L., Furlan, L., Monti, A., Marsala, E., Cernuschi, G., Solbiati, M., Bracco, C., Bandini, G., Pecorino Meli, M., Casazza, G., Montano, N., Sbrojavacca, R., & Costantino, G. (2021). Short vs long-course antibiotic therapy in pyelonephritis: a comparison of systematic reviews and guidelines for the SIMI choosing wisely campaign. *Internal and Emergency Medicine*, *16*(2), 313–323. https://doi.org/10.1007/s11739-020-02401-4

Fairfield, C., Penninga, L., Powell, J., Harrison, E. M., & Wigmore, S. J. (2018). Glucocorticosteroid-free versus glucocorticosteroid-containing immunosuppression for liver transplanted patients. *Cochrane Database of Systematic Reviews*, *2018*(4), CD007606. https://doi.org/10.1002/14651858.CD007606.pub4

Fan, S. Q., Jin, S., Tang, T. C., Chen, M., & Zheng, H. (2021). Efficacy of acupuncture for migraine prophylaxis: a trial sequential meta-analysis. *Journal of Neurology*, *268*(11), 4128–4137. https://doi.org/10.1007/s00415-020-10178-x

Fan, Z., Di, A., Huang, F., Zhao, S., Qiu, M., Wu, C., Huang, C., Guo, R., Tian, Q., & Wu, S. (2021). The effectiveness and safety of Tuina for tension-type headache: A systematic review and meta-analysis. *Complementary Therapies in Clinical Practice*, *43*(PG-101293), 101293. https://doi.org/10.1016/j.ctcp.2020.101293

Ferella, L., Limoncin, E., Vittorini, F., Chalaszczyk, A., Sorce, C., Grimaldi, G., Franzese, P., Ruggieri, V., Varrassi, E., Di Staso, M., Gimenez De Lorenzo, R., Marampon, F., Tombolini, V., Masciocchi, C., & Gravina, G. L. (2019). Are we ready for a paradigm shift from high-dose conventional to moderate hypofractionated radiotherapy in intermediate-high risk prostate cancer? A systematic review of randomized controlled trials with trial sequential analysis. *Critical Reviews in Oncology/Hematology*, *139*(PG-75-82), 75–82. https://doi.org/10.1016/j.critrevonc.2019.04.012

Fiorelli, E. M., Carandini, T., Gagliardi, D., Bozzano, V., Bonzi, M., Tobaldini, E., Comi, G. Pietro, Scarpini, E. A., Montano, N., & Solbiati, M. (2018). Secondary prevention of cryptogenic stroke in patients with patent

foramen ovale: A systematic review and meta-analysis. *Internal and Emergency Medicine*, *13*(8), 1287–1303. https://doi.org/10.1007/s11739-018-1909-8

Frost, S. A., Hou, Y. C., Lombardo, L., Metcalfe, L., Lynch, J. M., Hunt, L., Alexandrou, E., Brennan, K., Sanchez, D., Aneman, A., & Christensen, M. (2018). Evidence for the effectiveness of chlorhexidine bathing and health care-associated infections among adult intensive care patients: A trial sequential meta-analysis. *BMC Infectious Diseases*, *18*(1), 679. https://doi.org/10.1186/s12879-018-3521-y

Fu, Z., Geng, X., Chi, K., Song, C., Wu, D., Liu, C., & Hong, Q. (2021). Efficacy and safety of finerenone in patients with chronic kidney disease: A systematic review with meta-analysis and trial sequential analysis. *Annals of Palliative Medicine*, *10*(7), 7428–7439. https://doi.org/10.21037/apm-21-763

Fujii, T., Ganeko, R., Kataoka, Y., Furukawa, T. A., Featherstone, R., Doi, K., Vincent, J. L., Pasero, D., Robert, R., Ronco, C., & Bagshaw, S. M. (2018). Polymyxin B-immobilized hemoperfusion and mortality in critically ill adult patients with sepsis/septic shock: a systematic review with meta-analysis and trial sequential analysis. *Intensive Care Medicine*, *44*(2), 167–178. https://doi.org/10.1007/s00134-017-5004-9

Furlan, L., Erba, L., Trombetta, L., Sacco, R., Colombo, G., Casazza, G., Solbiati, M., Montano, N., Marta, C., Sbrojavacca, R., Perticone, F., Corazza, G. R., & Costantino, G. (2019). Short- vs long-course antibiotic therapy for pneumonia: a comparison of systematic reviews and guidelines for the SIMI Choosing Wisely Campaign. *Internal and Emergency Medicine*, *14*(3), 377–394. https://doi.org/10.1007/s11739-018-1955-2

Gamp, M., Becker, C., Tondorf, T., Hochstrasser, S., Metzger, K., Meinlschmidt, G., Langewitz, W., Schäfert, R., Bassetti, S., & Hunziker, S. (2019). Effect of Bedside vs. Non-bedside Patient Case Presentation During Ward Rounds: a Systematic Review and Meta-analysis. *Journal of General Internal Medicine*, *34*(3), 447–457. https://doi.org/10.1007/s11606-018-4714-1

Gao, D., Wang, Y., Zhang, R., & Zhang, Y. (2021). Efficacy of Acetazolamide for the Prophylaxis of Acute Mountain Sickness: A Systematic Review, Meta-Analysis and Trial Sequential Analysis of Randomized Clinical Trials. *American Journal of the Medical Sciences*, *361*(5), 635–645. https://doi.org/10.1016/j.amjms.2020.12.022

Gao, H. F., Lin, Y. Y., Zhu, T., Ji, F., Zhang, L. L., Yang, C. Q., Yang, M., Li, J. Q., Cheng, M. Y., & Wang, K. (2021). Adjuvant CDK4/6 inhibitors combined with endocrine therapy in HR-positive, HER2-negative early breast cancer: A meta-analysis of randomized clinical trials. *Breast*, *59*(PG-165-175), 165–175. https://doi.org/10.1016/j.breast.2021.07.002

Gao, Y., Hou, L., Lu, C., Wang, Q., Pan, B., Wang, Q., Tian, J., & Ge, L. (2020). Enteral Lactoferrin Supplementation for Preventing Sepsis and Necrotizing Enterocolitis in Preterm Infants: A Meta-Analysis With Trial Sequential Analysis of Randomized Controlled Trials. *Frontiers in Pharmacology*, *11*(no pagination PG-1186), 1186. https://doi.org/10.3389/fphar.2020.01186

Gareb, B., van Bakelen, N. B., Dijkstra, P. U., Vissink, A., Bos, R. R. M., & van Minnen, B. (2020). Biodegradable versus titanium osteosynthesis in maxillofacial traumatology: a systematic review with meta-analysis and trial sequential analysis. *International Journal of Oral and Maxillofacial Surgery*, *49*(7), 914–931. https://doi.org/10.1016/j.ijom.2019.11.009

Gareb, B., van Bakelen, N. B., Dijkstra, P. U., Vissink, A., Bos, R. R. M., & van Minnen, B. (2021). Efficacy and morbidity of biodegradable versus titanium osteosyntheses in orthognathic surgery: A systematic review

with meta-analysis and trial sequential analysis. *European Journal of Oral Sciences*, *129*(5), e12800. https://doi.org/10.1111/eos.12800

Garofalo, C., Capuano, I., Pennino, L., De Gregorio, I., Riccio, E., Provenzano, M., Crocetto, F., Buonanno, P., Pandolfo, S. D., Andreucci, M., & Pisani, A. (2021). The effects of somatostatin analogues on liver volume and quality of life in polycystic liver disease: a meta-analysis of randomized controlled trials. *Scientific Reports*, *11*(1), 23500. https://doi.org/10.1038/s41598-021-02812-z

Ge, X., Wang, W., Hou, L., Yang, K., & Fa, X. (2018). Inspiratory muscle training is associated with decreased postoperative pulmonary complications: Evidence from randomized trials. *Journal of Thoracic and Cardiovascular Surgery*, *156*(3), 1290-1300.e5. https://doi.org/10.1016/j.jtcvs.2018.02.105

Giacoppo, D., Caronna, N., Frangieh, A. H., Michel, J., Andò, G., Tarantini, G., Kasel, A. M., Capodanno, D., & Byrne, R. A. (2018). Long-term effectiveness and safety of transcatheter closure of patent foramen ovale compared with antithrombotic therapy alone: A meta-analysis of six randomised clinical trials and 3,560 patients with reconstructed time-to-event data. *EuroIntervention*, *14*(8), 857–867. https://doi.org/10.4244/EIJ-D-18-00341

Gianola, S., Castellini, G., Pecoraro, V., Monticone, M., Banfi, G., & Moja, L. (2020). Effect of Muscular Exercise on Patients With Muscular Dystrophy: A Systematic Review and Meta-Analysis of the Literature. *Frontiers in Neurology*, *11*(PG-958), 958. https://doi.org/10.3389/fneur.2020.00958

Giglio, M., Dalfino, L., Puntillo, F., & Brienza, N. (2019). Hemodynamic goal-directed therapy and postoperative kidney injury: An updated meta-analysis with trial sequential analysis. *Critical Care*, *23*(1), 232. https://doi.org/10.1186/s13054-019-2516-4

Goda, R., Ganeshkumar, A., Katiyar, V., Sharma, R., Gurjar, H. K., Chaturvedi, A., Sahu, R., Rai, H. I. S., & Vora, Z. (2022). Efficacy of antimicrobial medicated ventricular catheters: a network meta-analysis with trial sequential analysis. *Neurosurgical Review*, *45*(1), 91–102. https://doi.org/10.1007/s10143-021-01532-2

Goh, E. T., Stokes, C. S., Sidhu, S. S., Vilstrup, H., Gluud, L. L., & Morgan, M. Y. (2018). L-ornithine L-aspartate for prevention and treatment of hepatic encephalopathy in people with cirrhosis. *Cochrane Database of Systematic Reviews*, *2018*(5), CD012410. https://doi.org/10.1002/14651858.CD012410.pub2

Gonvers, E., El-Boghdadly, K., Grape, S., & Albrecht, E. (2021). Efficacy and safety of intrathecal morphine for analgesia after lower joint arthroplasty: a systematic review and meta-analysis with meta-regression and trial sequential analysis. *Anaesthesia*, *76*(12), 1648–1658. https://doi.org/10.1111/anae.15569

Göstemeyer, G., da Mata, C., McKenna, G., & Schwendicke, F. (2019). Atraumatic vs conventional restorative treatment for root caries lesions in older patients: Meta- and trial sequential analysis. *Gerodontology*, *36*(3), 285–293. https://doi.org/10.1111/ger.12409

Grape, S., Kirkham, K. R., Frauenknecht, J., & Albrecht, E. (2019). Intra-operative analgesia with remifentanil vs. dexmedetomidine: a systematic review and meta-analysis with trial sequential analysis. *Anaesthesia*, *74*(6), 793–800. https://doi.org/10.1111/anae.14657

Grape, S., El-Boghdadly, K., & Albrecht, E. (2020). Analgesic efficacy of PECS vs paravertebral blocks after radical mastectomy: A systematic review, meta-analysis and trial sequential analysis. *Journal of Clinical Anesthesia*, *63*(PG-109745), 109745. https://doi.org/10.1016/j.jclinane.2020.109745

Grape, S., Jaunin, E., El-Boghdadly, K., Chan, V., & Albrecht, E. (2020). Analgesic efficacy of PECS and serratus plane blocks after breast surgery: A systematic review, meta-analysis and trial sequential analysis. *Journal of Clinical Anesthesia*, *63*(PG-109744), 109744. https://doi.org/10.1016/j.jclinane.2020.109744

Grape, S., Kirkham, K. R., & Albrecht, E. (2020). The Analgesic Efficacy of Transversus Abdominis Plane Block After Bariatric Surgery: a Systematic Review and Meta-analysis with Trial Sequential Analysis. *Obesity Surgery*, *30*(10), 4061–4070. https://doi.org/10.1007/s11695-020-04768-x

Grape, S., Kirkham, K. R., Akiki, L., & Albrecht, E. (2021). Transversus abdominis plane block versus local anesthetic wound infiltration for optimal analgesia after laparoscopic cholecystectomy: A systematic review and meta-analysis with trial sequential analysis. *Journal of Clinical Anesthesia*, *75*(PG-110450), 110450. https://doi.org/10.1016/j.jclinane.2021.110450

Grape, S., Kirkham, K. R., & Albrecht, E. (2022). Transversus abdominis plane block versus local anaesthetic wound infiltration for analgesia after caesarean section: A systematic review and meta-analysis with trial sequential analysis. *European Journal of Anaesthesiology*, *39*(3), 244–251. https://doi.org/10.1097/EJA.0000000000001552

Gu, S. C., & Wang, C. De. (2018). Early Selective Serotonin Reuptake Inhibitors for Recovery after Stroke: A Meta-Analysis and Trial Sequential Analysis. *Journal of Stroke and Cerebrovascular Diseases*, *27*(5), 1178–1189. https://doi.org/10.1016/j.jstrokecerebrovasdis.2017.11.031

Gudivada, K. K., Kumar, A., Shariff, M., Sampath, S., Varma, M. M., Sivakoti, S., & Krishna, B. (2021). Antioxidant micronutrient supplementation in critically ill adults: A systematic review with meta-analysis and trial sequential analysis. *Clinical Nutrition*, *40*(3), 740–750. https://doi.org/10.1016/j.clnu.2020.06.033

Guo, S., Guo, X., Zhang, H., Zhang, X., & Li, Z. (2020). The Effect of Diacerein on Type 2 Diabetic Mellitus: A Systematic Review and Meta-Analysis of Randomized Controlled Trials with Trial Sequential Analysis. *Journal of Diabetes Research*, *2020*(PG-2593792), 2593792. https://doi.org/10.1155/2020/2593792

Guo, W. Q., Li, L., Su, Q., Sun, Y. H., Wang, X. T., Dai, W. R., & Li, H. Q. (2018). Optimal timing of complete revascularization in patients with st-segment elevation myocardial infarction and multivessel disease: A pairwise and network meta-analysis. *Clinical Epidemiology*, *10*(PG-1037-1051), 1037–1051. https://doi.org/10.2147/CLEP.S167138

Guo, W., Yi, L., Zhou, B., & Li, M. (2020). Chitosan modifies glycemic levels in people with metabolic syndrome and related disorders: meta-analysis with trial sequential analysis. *Nutrition Journal*, *19*(1), 130. https://doi.org/10.1186/s12937-020-00647-4

Guo, X., Guo, S., Miao, Z., Li, Z., & Zhang, H. (2018). Myo-inositol lowers the risk of developing gestational diabetic mellitus in pregnancies: A systematic review and meta-analysis of randomized controlled trials with trial sequential analysis. *Journal of Diabetes and Its Complications*, *32*(3), 342–348. https://doi.org/10.1016/j.jdiacomp.2017.07.007

Guo, Z., Liu, J., Lei, L., Xue, Y., Liu, L., Huang, H., Chen, S., Liu, Y., Lin, Y., Tao, J., Xu, Q., Wu, K., Zhang, L., & Chen, J. Y. (2020). Effect of N-acetylcysteine on prevention of contrast-associated acute kidney injury in patients with STEMI undergoing primary percutaneous coronary intervention: A systematic review and

meta-analysis of randomised controlled trials. *BMJ Open*, *10*(10), e039009. https://doi.org/10.1136/bmjopen-2020-039009

Gupta, A., Sahai, A., Aggarwal, V., Mehta, N., Abraham, D., Jala, S., & Singh, A. (2021). Anesthetic efficacy of primary and supplemental buccal/lingual infiltration in patients with irreversible pulpitis in human mandibular molars: a systematic review and meta-analysis. *Journal of Dental Anesthesia and Pain Medicine*, *21*(4), 283. https://doi.org/10.17245/jdapm.2021.21.4.283

Gupta, R., Malik, A. H., Gupta, R., Ranchal, P., Yandrapalli, S., Patel, B., Frishman, W. H., Aronow, W. S., & Garg, J. (2021). Dual Versus Triple Therapy in Patients with Acute Coronary Syndrome and an Anticoagulation Indication: A Systematic Review with Meta-Analysis and Trial-Sequential Analysis. *Cardiology in Review*, *29*(5), 245–252. https://doi.org/10.1097/CRD.0000000000000320

Habib Bedwani, N. A. R., Kelada, M., Smart, N., Szydlo, R., Patten, D. K., & Bhargava, A. (2021). Glue versus mechanical mesh fixation in laparoscopic inguinal hernia repair: meta-analysis and trial sequential analysis of randomized clinical trials. *British Journal of Surgery*, *108*(1), 14–23. https://doi.org/10.1093/bjs/znaa002

Hajibandeh, S., Hajibandeh, S., Antoniou, S. A., Torella, F., & Antoniou, G. A. (2018). Meta-analysis and trial sequential analysis of local vs. general anaesthesia for carotid endarterectomy. *Anaesthesia*, *73*(10), 1280–1289. https://doi.org/10.1111/anae.14320

Hajibandeh, S., Finch, D. A., Mohamedahmed, A. Y. Y., Iskandar, A., Venkatesan, G., Hajibandeh, S., & Satyadas, T. (2021). Meta-analysis and trial sequential analysis of three-port vs four-port laparoscopic cholecystectomy (level 1 evidence). *Updates in Surgery*, *73*(2), 451–471. https://doi.org/10.1007/s13304-021-00982-z

Hajibandeh, S., Hajibandeh, S., Kennedy-Dalby, A., Rehman, S., & Zadeh, R. A. (2018). Purse-string skin closure versus linear skin closure techniques in stoma closure: a comprehensive meta-analysis with trial sequential analysis of randomised trials. *International Journal of Colorectal Disease*, *33*(10), 1319–1332. https://doi.org/10.1007/s00384-018-3139-y

Hajibandeh, S., Hajibandeh, S., & Maw, A. (2020). Meta-analysis and trial sequential analysis of randomized controlled trials comparing high and low ligation of the inferior mesenteric artery in rectal cancer surgery. *Diseases of the Colon and Rectum*, *63*(7), 988–999. https://doi.org/10.1097/DCR.0000000000001693

Hajibandeh, S., Hajibandeh, S., Saeed, S., Bird, J., Kannappa, L. K., & Ratnayake, I. (2022). Effect of hyaluronate-based bioresorbable membrane (Seprafilm) on outcomes of abdominal surgery: a meta-analysis and trial sequential analysis of randomised controlled trials. *Updates in Surgery*, *74*(3), 865–881. https://doi.org/10.1007/s13304-021-01117-0

He, J., Hou, J. huan, Qi, J., Zhang, T., Wang, Y. ling, & Qian, M. (2020). Mindfulness Ased Stress Reduction Interventions for Cancer Related Fatigue: A Meta-Analysis and Systematic Review. *Journal of the National Medical Association*, *112*(4), 387–394. https://doi.org/10.1016/j.jnma.2020.04.006

Heesen, M., Klimek, M., Imberger, G., Hoeks, S. E., Rossaint, R., & Straube, S. (2018). Co-administration of dexamethasone with peripheral nerve block: intravenous vs perineural application: systematic review, meta-analysis, meta-regression and trial-sequential analysis. *British Journal of Anaesthesia*, *120*(2), 212–227. https://doi.org/10.1016/j.bja.2017.11.062

Heesen, M., Rijs, K., Hilber, N., Eid, K., Al-Oweidi, A., Rossaint, R., & Klimek, M. (2019). Effect of intravenous dexamethasone on postoperative pain after spinal anaesthesia – a systematic review with meta-analysis and trial sequential analysis. *Anaesthesia*, *74*(8), 1047–1056. https://doi.org/10.1111/anae.14666
Heesen, M., Rijs, K., Hilber, N., Ngan Kee, W. D., Rossaint, R., van der Marel, C., & Klimek, M. (2019). Ephedrine versus phenylephrine as a vasopressor for spinal anaesthesia-induced hypotension in parturients undergoing high-risk caesarean section: meta-analysis, meta-regression and trial sequential analysis. *International Journal of Obstetric Anesthesia*, *37*(PG-16-28), 16–28. https://doi.org/10.1016/j.ijoa.2018.10.006
Herbert, G., Perry, R., Andersen, H. K., Atkinson, C., Penfold, C., Lewis, S. J., Ness, A. R., & Thomas, S. (2019). Early enteral nutrition within 24 hours of lower gastrointestinal surgery versus later commencement for length of hospital stay and postoperative complications. *Cochrane Database of Systematic Reviews*, *2019*(7), CD004080. https://doi.org/10.1002/14651858.CD004080.pub4
Herrod, P. J., Boyd-Carson, H., Doleman, B., Blackwell, J., Williams, J. P., Bhalla, A., Nelson, R. L., Tou, S., & Lund, J. N. (2019). Prophylactic antibiotics for penetrating abdominal trauma: duration of use and antibiotic choice. *Cochrane Database of Systematic Reviews*, *2019*(12), CD010808. https://doi.org/10.1002/14651858.CD010808.pub2
Hiemstra, B., Koster, G., Wetterslev, J., Gluud, C., Jakobsen, J. C., Scheeren, T. W. L., Keus, F., & van der Horst, I. C. C. (2019). Dopamine in critically ill patients with cardiac dysfunction: A systematic review with meta-analysis and trial sequential analysis. *Acta Anaesthesiologica Scandinavica*, *63*(4), 424–437. https://doi.org/10.1111/aas.13294
Hoshijima, H., Maruyama, K., Mihara, T., Boku, A. S., Shiga, T., & Nagasaka, H. (2020). Use of the GlideScope does not lower the hemodynamic response to tracheal intubation more than the Macintosh laryngoscope: A systematic review and meta-analysis. *Medicine (United States)*, *99*(48), E23345. https://doi.org/10.1097/MD.0000000000023345
Hoshijima, H., Maruyama, K., Mihara, T., Mieda, T., Shiga, T., & Nagasaka, H. (2018). Airtraq® reduces the hemodynamic response to tracheal intubation using single-lumen tubes in adults compared with the Macintosh laryngoscope: A systematic review and meta-analysis of randomized control trials. *Journal of Clinical Anesthesia*, *47*(PG-86-94), 86–94. https://doi.org/10.1016/j.jclinane.2018.03.022
Hoshijima, H., Mihara, T., Denawa, Y., Shiga, T., & Mizuta, K. (2021). Comparison of Hemodynamic Responses to Administration of Vasopressin and Norepinephrine Under General Anesthesia: A Systematic Review and Meta-analysis of Randomized Controlled Trials with Trial Sequential Analysis. *Journal of Cardiothoracic and Vascular Anesthesia*, *35*(1), 61–69. https://doi.org/10.1053/j.jvca.2020.08.011
Hoshijima, H., Mihara, T., Maruyama, K., Denawa, Y., Mizuta, K., Shiga, T., & Nagasaka, H. (2018). C-MAC videolaryngoscope versus Macintosh laryngoscope for tracheal intubation: A systematic review and meta-analysis with trial sequential analysis. *Journal of Clinical Anesthesia*, *49*(PG-53-62), 53–62. https://doi.org/10.1016/j.jclinane.2018.06.007
Hoshijima, H., Mihara, T., Maruyama, K., Denawa, Y., Takahashi, M., Shiga, T., & Nagasaka, H. (2018). McGrath videolaryngoscope versus Macintosh laryngoscope for tracheal intubation: A systematic review

and meta-analysis with trial sequential analysis. *Journal of Clinical Anesthesia*, *46*(PG-25-32), 25–32. https://doi.org/10.1016/j.jclinane.2017.12.030

Hu, B., Zhou, Q., Hu, Y. yang, Zhuang, L., Yi, L. ping, Cao, J. xia, Li, T. qi, & Wang, J. (2019). Efficacy and Safety of Once-Weekly versus Twice-Weekly Bortezomib in Patients with Hematologic Malignancies: A Meta-analysis with Trial Sequential Analysis. *Pharmacotherapy*, *39*(6), 697–708. https://doi.org/10.1002/phar.2267

Hu, J., Spina, S., Zadek, F., Kamenshchikov, N. O., Bittner, E. A., Pedemonte, J., & Berra, L. (2019). Effect of nitric oxide on postoperative acute kidney injury in patients who underwent cardiopulmonary bypass: a systematic review and meta-analysis with trial sequential analysis. *Annals of Intensive Care*, *9*(1), 129. https://doi.org/10.1186/s13613-019-0605-9

Hu, Z., Qu, S., Zhang, J., Cao, X., Wang, P., Huang, S., Shi, F., Dong, Y., Wu, J., Tang, B., & Zhu, J. (2019). Efficacy and Safety of Platelet-Rich Plasma for Patients with Diabetic Ulcers: A Systematic Review and Meta-analysis. *Advances in Wound Care*, *8*(7), 298–308. https://doi.org/10.1089/wound.2018.0842

Huang, F., Xie, Y., Zhao, S., Feng, Z., Chen, G., & Xu, Y. (2019). The Effectiveness and Safety of Acupoint Catgut Embedding for the Treatment of Postmenopausal Osteoporosis: A Systematic Review and Meta-Analysis. *Evidence-Based Complementary and Alternative Medicine*, *2019*(PG-2673763), 2673763. https://doi.org/10.1155/2019/2673763

Huang, F., Zhao, S., Dai, L., Feng, Z., Wu, Z., Chen, J., Guo, R., Tian, Q., Fan, Z., & Wu, S. (2020). Tuina for cervical vertigo: A systematic review and meta-analysis of randomized controlled trials. *Complementary Therapies in Clinical Practice*, *39*(PG-101115), 101115. https://doi.org/10.1016/j.ctcp.2020.101115

Huang, H., Liao, D., Zou, Y., & Chi, H. (2020). The effects of chitosan supplementation on body weight and body composition: a systematic review and meta-analysis of randomized controlled trials. *Critical Reviews in Food Science and Nutrition*, *60*(11), 1815–1825. https://doi.org/10.1080/10408398.2019.1602822

Huang, H., Zou, Y., Chi, H., & Liao, D. (2018). Lipid-Modifying Effects of Chitosan Supplementation in Humans: A Pooled Analysis with Trial Sequential Analysis. *Molecular Nutrition and Food Research*, *62*(8), e1700842. https://doi.org/10.1002/mnfr.201700842

Huang, H. ping, Zhao, W. jun, & Pu, J. (2020). Effect of mild hypothermia on prognosis of patients with severe traumatic brain injury: A meta-analysis with trial sequential analysis. *Australian Critical Care*, *33*(4), 375–381. https://doi.org/10.1016/j.aucc.2019.08.005

Huang, H. P., Zhao, W. J., Pu, J., & He, F. (2021). Prophylactic negative pressure wound therapy for surgical site infection in obese women undergoing cesarean section: an evidence synthesis with trial sequential analysis. *Journal of Maternal-Fetal and Neonatal Medicine*, *34*(15), 2498–2505. https://doi.org/10.1080/14767058.2019.1668924

Huang, H. ping, Zhao, W. jun, Wen, F., & Li, X. yu. (2021). Application of ultrasound-guided radial artery cannulation in paediatric patients: A systematic review and meta-analysis. *Australian Critical Care*, *34*(4), 388–394. https://doi.org/10.1016/j.aucc.2020.09.001

Huang, H. W., Sun, X. M., Shi, Z. H., Chen, G. Q., Chen, L., Friedrich, J. O., & Zhou, J. X. (2018). Effect of High-Flow Nasal Cannula Oxygen Therapy Versus Conventional Oxygen Therapy and Noninvasive Ventilation on

Reintubation Rate in Adult Patients After Extubation: A Systematic Review and Meta-Analysis of Randomized Controlled Trials. *Journal of Intensive Care Medicine*, *33*(11), 609–623. https://doi.org/10.1177/0885066617705118

Huang, H., Li, G., Wang, H., & He, M. (2018). Optimal skin antiseptic agents for prevention of surgical site infection in cesarean section: a meta-analysis with trial sequential analysis. *Journal of Maternal-Fetal and Neonatal Medicine*, *31*(24), 3267–3274. https://doi.org/10.1080/14767058.2017.1368481

Huang, K. L., Wang, S. Y., Lu, W. C., Chang, Y. H., Su, J., & Lu, Y. T. (2019). Effects of low-dose computed tomography on lung cancer screening: A systematic review, meta-analysis, and trial sequential analysis. *BMC Pulmonary Medicine*, *19*(1), 126. https://doi.org/10.1186/s12890-019-0883-x

Huang, X., Wang, J., Lin, W., Zhang, N., Du, J., Long, Z., Yang, Y., Zheng, B., Zhong, F., Wu, Q., & Ma, W. (2020). Kanglaite injection plus platinum-based chemotherapy for stage III/IV non-small cell lung cancer: A meta-analysis of 27 RCTs. *Phytomedicine*, *67*(PG-153154), 153154. https://doi.org/10.1016/j.phymed.2019.153154

Hung, K. C., Chen, J. Y., Feng, I. J., Chiang, M. H., Wu, S. C., Chen, I. W., Lin, Y. T., Chang, Y. J., Wu, Z. F., Lu, H. F., & Sun, C. K. (2021). Efficacy and airway complications of Parker Flex-Tip tubes and standard endotracheal tubes during airway manipulation: A meta-analysis and trial sequential analysis. *European Journal of Anaesthesiology*, *38*(8), 813–824. https://doi.org/10.1097/EJA.0000000000001539

Hussain, N., Lagnese, C. M., Hayes, B., Kumar, N., Weaver, T. E., Essandoh, M. K., Reno, J., Small, R. H., & Abdallah, F. W. (2020). Comparative analgesic efficacy and safety of intermittent local anaesthetic epidural bolus for labour: a systematic review and meta-analysis. *British Journal of Anaesthesia*, *125*(4), 560–579. https://doi.org/10.1016/j.bja.2020.05.060

Hussain, N., Shastri, U., McCartney, C. J. L., Gilron, I., Fillingim, R. B., Clarke, H., Katz, J., Juni, P., Laupacis, A., Wijeysundera, D., & Abdallah, F. W. (2018). Should thoracic paravertebral blocks be used to prevent chronic postsurgical pain after breast cancer surgery? a systematic analysis of evidence in light of IMMPACT recommendations. *Pain*, *159*(10), 1955–1971. https://doi.org/10.1097/j.pain.0000000000001292

Ivan, I., Budiman, F., Ruby, R., Wendi, I. P., & Ridjab, D. A. (2021). Current evidence of survival benefit between chest-compression only versus standard cardiopulmonary resuscitation in out-of-hospital cardiac arrest: Updated systematic review and meta-analysis of randomized controlled trials with trial sequential analysis. *Herz*, *46*(PG-), 198–208. https://doi.org/10.1007/s00059-020-04982-4

Jackson, J. L., Kuriyama, A., Kuwatsuka, Y., Nickoloff, S., Storch, D., Jackson, W., Zhang, Z. J., & Hayashino, Y. (2019). Beta-blockers for the prevention of headache in adults, a systematic review and meta-analysis. *PLoS ONE*, *14*(3), e0212785. https://doi.org/10.1371/journal.pone.0212785

Jairam, A. P., López-Cano, M., Garcia-Alamino, J. M., Pereira, J. A., Timmermans, L., Jeekel, J., Lange, J., & Muysoms, F. (2020). Prevention of incisional hernia after midline laparotomy with prophylactic mesh reinforcement: a meta-analysis and trial sequential analysis. *BJS Open*, *4*(3), 357–368. https://doi.org/10.1002/bjs5.50261

Jayaraman, J., Nagendrababu, V., Pulikkotil, S. J., Veettil, S. K., & Dhar, V. (2020). Effectiveness of formocresol and ferric sulfate as pulpotomy material in primary molars: A systematic review and meta-

analysis with trial sequential analysis of randomized clinical trials. *Quintessence International*, *51*(1), 38–48. https://doi.org/10.3290/j.qi.a43617

Jensen, M. M., Marker, S., Do, H. Q., Barbateskovic, M., Perner, A., & Møller, M. H. (2021). Prophylactic acid suppressants in children in the intensive care unit: a systematic review with meta-analysis and trial sequential analysis. *Acta Anaesthesiologica Scandinavica*, *65*(3), 292–301. https://doi.org/10.1111/aas.13731

Jhuang, B. J., Yeh, B. H., Huang, Y. T., & Lai, P. C. (2021). Efficacy and Safety of Remimazolam for Procedural Sedation: A Meta-Analysis of Randomized Controlled Trials With Trial Sequential Analysis. *Frontiers in Medicine*, *8*(PG-641866), 641866. https://doi.org/10.3389/fmed.2021.641866

Ji, H., Zhou, X., Wei, W., Wu, W., & Yao, S. (2020). Ginkgol Biloba extract as an adjunctive treatment for ischemic stroke: A systematic review and meta-analysis of randomized clinical trials. *Medicine (United States)*, *99*(2), e18568. https://doi.org/10.1097/MD.0000000000018568

Jiang, L., Sheng, Y., Feng, X., & Wu, J. (2019). The effects and safety of vasopressin receptor agonists in patients with septic shock: A meta-analysis and trial sequential analysis. *Critical Care*, *23*(1), 91. https://doi.org/10.1186/s13054-019-2362-4

Jiang, Q., Li, J., Mei, L., Du, J., Levrini, L., Abbate, G. M., & Li, H. (2018). Periodontal health during orthodontic treatment with clear aligners and fixed appliances: A meta-analysis. *Journal of the American Dental Association*, *149*(8), 712-720.e12. https://doi.org/10.1016/j.adaj.2018.04.010

Jiang, X., Jiang, C., Huang, C., Chen, G., Jiang, K., Huang, B., & Liu, F. (2018). Berberine Combined with Triple Therapy versus Triple Therapy for Helicobacter pylori Eradication: A Meta-Analysis of Randomized Controlled Trials. *Evidence-Based Complementary and Alternative Medicine*, *2018*(PG-8716910), 8716910. https://doi.org/10.1155/2018/8716910

Jiao, X. F., Lin, X. M., Ni, X. F., Li, H. L., Zhang, C., Yang, C. S., Song, H. X., Yi, Q. S., & Zhang, L. L. (2019). Volatile anesthetics versus total intravenous anesthesia in patients undergoing coronary artery bypass grafting: An updated metaanalysis and trial sequential analysis of randomized controlled trials. *PLoS ONE*, *14*(10), e0224562. https://doi.org/10.1371/journal.pone.0224562

Jin, Z., Durrands, T., Li, R., Gan, T. J., & Lin, J. (2020). Pectoral block versus paravertebral block: A systematic review, meta-analysis and trial sequential analysis. *Regional Anesthesia and Pain Medicine*, *45*(9), 727–732. https://doi.org/10.1136/rapm-2020-101512

Jin, Z., Kowa, C. Y., Gan, S., Lin, J., & Gan, T. J. (2021). Efficacy of palonosetron-dexamethasone combination compared to palonosetron alone for prophylaxis against postoperative nausea and vomiting. *Current Medical Research and Opinion*, *37*(5), 711–718. https://doi.org/10.1080/03007995.2021.1893677

Jin, Z., Li, R., Gan, T. J., He, Y., & Lin, J. (2020). Pectoral Nerve (PECs) block for postoperative analgesia-a systematic review and meta-analysis with trial sequential analysis. *International Journal of Physiology, Pathophysiology and Pharmacology*, *12*(1), 40–50. http://www.ncbi.nlm.nih.gov/pubmed/32211121%0Ahttp://www.pubmedcentral.nih.gov/articlerender.fcgi?artid=PMC7076325

Jing, R., Dai, H. J., Lin, F., Ge, W. Y., & Pan, L. H. (2018). Conscious Sedation versus General Anesthesia for Patients with Acute Ischemic Stroke Undergoing Endovascular Therapy: A Systematic Review and Meta-

Analysis. *BioMed Research International*, *2018*(PG-2318489), 2318489. https://doi.org/10.1155/2018/2318489

Jørgensen, M. S., Storebø, O. J., Stoffers-Winterling, J. M., Faltinsen, E., Todorovac, A., & Simonsen, E. (2021). Psychological therapies for adolescents with borderline personality disorder (BPD) or BPD features—A systematic review of randomized clinical trials with meta-analysis and Trial Sequential Analysis. *PLoS ONE*, *16*(1 January), e0245331. https://doi.org/10.1371/journal.pone.0245331

Junhai, Z., Bangchuan, H., Shijin, G., Jing, Y., & Li, L. (2021). Glucocorticoids for acute respiratory distress syndrome: A systematic review with meta-analysis and trial sequential analysis. *European Journal of Clinical Investigation*, *51*(6), e13496. https://doi.org/10.1111/eci.13496

Juul, S., Nielsen, E. E., Feinberg, J., Siddiqui, F., Jørgensen, C. K., Barot, E., Holgersson, J., Nielsen, N., Bentzer, P., Veroniki, A. A., Thabane, L., Bu, F., Klingenberg, S., Gluud, C., & Jakobsen, J. C. (2021). Interventions for treatment of COVID-19: Second edition of a living systematic review with meta-analyses and trial sequential analyses (The LIVING Project). *PLoS ONE*, *16*(3 March), e0248132. https://doi.org/10.1371/journal.pone.0248132

Juul, S., Nielsen, E. E., Feinberg, J., Siddiqui, F., Jørgensen, C. K., Barot, E., Nielsen, N., Bentzer, P., Veroniki, A. A., Thabane, L., Bu, F., Klingenberg, S., Gluud, C., & Jakobsen, J. C. (2020). Interventions for treatment of COVID-19: A living systematic review with meta-analyses and trial sequential analyses (The LIVING Project). *PLoS Medicine*, *17*(9), e1003293. https://doi.org/10.1371/journal.pmed.1003293

Kalafateli, M., Buzzetti, E., Thorburn, D., Davidson, B. R., Tsochatzis, E., & Gurusamy, K. S. (2018). Pharmacological interventions for acute hepatitis C infection. *Cochrane Database of Systematic Reviews*, *2018*(12), CD011644. https://doi.org/10.1002/14651858.CD011644.pub3

Kashani, H. H., Lodewyks, C., Kavosh, M. S., Jeyaraman, M. M., Neilson, C., Okoli, G., Rabbani, R., Abou-Setta, A. M., Zarychanski, R., & Grocott, H. P. (2020). The effect of restrictive versus liberal transfusion strategies on longer-term outcomes after cardiac surgery: a systematic review and meta-analysis with trial sequential analysis. *Canadian Journal of Anesthesia*, *67*(5), 577–587. https://doi.org/10.1007/s12630-020-01592-w

Katiyar, V., Chaturvedi, A., Sharma, R., Gurjar, H. K., Goda, R., Singla, R., & Ganeshkumar, A. (2020). Meta-Analysis with Trial Sequential Analysis on the Efficacy and Safety of Erythropoietin in Traumatic Brain Injury: A New Paradigm. *World Neurosurgery*, *142*(PG-465-475), 465–475. https://doi.org/10.1016/j.wneu.2020.05.142

Katsanos, K., Spiliopoulos, S., Kitrou, P., Krokidis, M., & Karnabatidis, D. (2018). Risk of death following application of paclitaxel-coated balloons and stents in the femoropopliteal artery of the leg: A systematic review and meta-analysis of randomized controlled trials. *Journal of the American Heart Association*, *7*(24), e011245. https://doi.org/10.1161/JAHA.118.011245

Kawakami, H., Mihara, T., Nakamura, N., Ka, K., & Goto, T. (2018). Effect of magnesium added to local anesthetics for caudal anesthesia on postoperative pain in pediatric surgical patients: A systematic review and meta-analysis with Trial Sequential Analysis. *PLoS ONE*, *13*(1), e0190354. https://doi.org/10.1371/journal.pone.0190354

Kawakami, H., Nakajima, D., Mihara, T., Sato, H., & Goto, T. (2019). Effectiveness of Magnesium in Preventing Shivering in Surgical Patients: A Systematic Review and Meta-analysis. *Anesthesia and Analgesia*, *129*(3), 689–700. https://doi.org/10.1213/ANE.0000000000004024

Kawano-Dourado, L., Zampieri, F. G., Azevedo, L. C. P., Corrêa, T. D., Figueiró, M., Semler, M. W., Kellum, J. A., & Cavalcanti, A. B. (2018). Low-versus high-chloride content intravenous solutions for critically ill and perioperative adult patients: A systematic review and meta-analysis. *Anesthesia and Analgesia*, *126*(2), 513–521. https://doi.org/10.1213/ANE.0000000000002641

Khan, R. M. A., Ali, B., Hajibandeh, S., & Hajibandeh, S. (2018). Effect of mesalazine on recurrence of diverticulitis in patients with symptomatic uncomplicated diverticular disease: a meta-analysis with trial sequential analysis of randomized controlled trials. *Colorectal Disease*, *20*(6), 469–478. https://doi.org/10.1111/codi.14064

Kheiri, B., Osman, M., Bakhit, A., Radaideh, Q., Abdalla, A., Barbarawi, M., Zayed, Y., Ahmed, S., Bachuwa, G., & Hassan, M. (2019). Dual versus triple therapy for patients with atrial fibrillation and acute coronary syndrome: a meta-analysis and trial sequential analysis of randomized controlled trials. *Journal of Thrombosis and Thrombolysis*, *48*(3), 511–513. https://doi.org/10.1007/s11239-019-01874-1

Kilambi, R., & Singh, A. N. (2018). Duct-to-mucosa versus dunking techniques of pancreaticojejunostomy after pancreaticoduodenectomy: Do we need more trials? A systematic review and meta-analysis with trial sequential analysis. *Journal of Surgical Oncology*, *117*(5), 928–939. https://doi.org/10.1002/jso.24986

Kiss, S., Németh, D., Hegyi, P., Földi, M., Szakács, Z., Erőss, B., Tinusz, B., Hegyi, P. J., Sarlós, P., & Alizadeh, H. (2021). Granulocyte and monocyte apheresis as an adjunctive therapy to induce and maintain clinical remission in ulcerative colitis: a systematic review and meta-analysis. *BMJ Open*, *11*(5), e042374. https://doi.org/10.1136/bmjopen-2020-042374

Klimek, M., Rossaint, R., van de Velde, M., & Heesen, M. (2018). Combined spinal-epidural vs. spinal anaesthesia for caesarean section: meta-analysis and trial-sequential analysis. *Anaesthesia*, *73*(7), 875–888. https://doi.org/10.1111/anae.14210

Kofler, T., Kurmann, R., Lehnick, D., Cioffi, G. M., Chandran, S., Attinger-Toller, A., Toggweiler, S., Kobza, R., Moccetti, F., Cuculi, F., Jolly, S. S., & Bossard, M. (2021). Colchicine in patients with coronary artery disease: A systematic review and meta-analysis of randomized trials. *Journal of the American Heart Association*, *10*(16), e021198. https://doi.org/10.1161/JAHA.121.021198

Kokotovic, D., Berkfors, A., Gögenur, I., Ekeloef, S., & Burcharth, J. (2021). The effect of postoperative respiratory and mobilization interventions on postoperative complications following abdominal surgery: a systematic review and meta-analysis. *European Journal of Trauma and Emergency Surgery*, *47*(4), 975–990. https://doi.org/10.1007/s00068-020-01522-x

Kondo, Y., Ota, K., Imura, H., Hara, N., & Shime, N. (2020). Prolonged versus intermittent β-lactam antibiotics intravenous infusion strategy in sepsis or septic shock patients: A systematic review with meta-Analysis and trial sequential analysis of randomized trials. *Journal of Intensive Care*, *8*(1), 77. https://doi.org/10.1186/s40560-020-00490-z

Kong, M., Chen, H., Xin, Y., Jiang, Y., Han, Y., & Sheng, H. (2021). High ligation of the inferior mesenteric artery and anastomotic leakage in anterior resection for rectal cancer: a systematic review and meta-

analysis of randomized controlled trial studies. *Colorectal Disease*, *23*(3), 614–624. https://doi.org/10.1111/codi.15419

Koning, M. V., Klimek, M., Rijs, K., Stolker, R. J., & Heesen, M. A. (2020). Intrathecal hydrophilic opioids for abdominal surgery: a meta-analysis, meta-regression, and trial sequential analysis. *British Journal of Anaesthesia*, *125*(3), 358–372. https://doi.org/10.1016/j.bja.2020.05.061

Korang, S. K., Maagaard, M., Feinberg, J. B., Perner, A., Gluud, C., & Jakobsen, J. C. (2021). The effects of adding quinolones to beta-lactam antibiotics for sepsis. *Acta Anaesthesiologica Scandinavica*, *65*(8), 1023–1032. https://doi.org/10.1111/aas.13831

Kotb, A., Hajibandeh, S., Hajibandeh, S., & Satyadas, T. (2021). Meta-analysis and trial sequential analysis of randomised controlled trials comparing standard versus extended lymphadenectomy in pancreatoduodenectomy for adenocarcinoma of the head of pancreas. *Langenbeck's Archives of Surgery*, *406*(3), 547–561. https://doi.org/10.1007/s00423-020-01999-5

Kreienbühl, L., Elia, N., Pfeil-Beun, E., Walder, B., & Tramèr, M. R. (2018). Patient-controlled versus clinician-controlled sedation with propofol: Systematic review and meta-analysis with trial sequential analyses. *Anesthesia and Analgesia*, *127*(4), 873–880. https://doi.org/10.1213/ANE.0000000000003361

Krois, J., Göstemeyer, G., Reda, S., & Schwendicke, F. (2018). Sealing or infiltrating proximal carious lesions. *Journal of Dentistry*, *74*(PG-15-22), 15–22. https://doi.org/10.1016/j.jdent.2018.04.026

Kulkarni, A. A., Sharma, G., Deo, K. B., & Jain, T. (2022). Umbilical port versus epigastric port for gallbladder extraction in laparoscopic cholecystectomy: A systematic review and meta-analysis of randomized controlled trials with trial sequential analysis. *Surgeon*, *20*(3), e26–e35. https://doi.org/10.1016/j.surge.2021.02.009

Kumar, A., Shariff, M., Adalja, D., & Doshi, R. (2019). Intravascular ultrasound versus angiogram guided drug eluting stent implantation. A systematic review and updated meta-analysis with trial sequential analysis. *IJC Heart and Vasculature*, *25*(PG-100419), 100419. https://doi.org/10.1016/j.ijcha.2019.100419

Kumar, A., Shariff, M., & Doshi, R. (2020). Impact of rosuvastatin versus atorvastatin on coronary atherosclerotic plaque volume – a systematic review and meta-analysis with trial sequential analysis of randomized control trials. *European Journal of Preventive Cardiology*, *27*(19), 2138–2141. https://doi.org/10.1177/2047487319868035

Kuriyama, A., Maeda, H., Sun, R., & Aga, M. (2018). Topical application of corticosteroids to tracheal tubes to prevent postoperative sore throat in adults undergoing tracheal intubation: a systematic review and meta-analysis. *Anaesthesia*, *73*(12), 1546–1556. https://doi.org/10.1111/anae.14273

Kuriyama, A., & Maeda, H. (2019). Preoperative intravenous dexamethasone prevents tracheal intubation-related sore throat in adult surgical patients: a systematic review and meta-analysis. *Canadian Journal of Anesthesia*, *66*(5), 562–575. https://doi.org/10.1007/s12630-018-01288-2

Kuriyama, A., & Maeda, H. (2019). Topical application of licorice for prevention of postoperative sore throat in adults: A systematic review and meta-analysis. *Journal of Clinical Anesthesia*, *54*(PG-25-32), 25–32. https://doi.org/10.1016/j.jclinane.2018.10.025

Kuriyama, A., Maeda, H., & Sun, R. (2019). Aerosolized corticosteroids to prevent postoperative sore throat in adults: A systematic review and meta-analysis. *Acta Anaesthesiologica Scandinavica*, *63*(3), 282–291. https://doi.org/10.1111/aas.13275

Kuriyama, A., Maeda, H., & Sun, R. (2019). Topical application of magnesium to prevent intubation-related sore throat in adult surgical patients: a systematic review and meta-analysis. *Canadian Journal of Anesthesia*, *66*(9), 1082–1094. https://doi.org/10.1007/s12630-019-01396-7

Kuriyama, A., Nakanishi, M., Kamei, J., Sun, R., Ninomiya, K., & Hino, M. (2020). Topical application of ketamine to prevent postoperative sore throat in adults: A systematic review and meta-analysis. *Acta Anaesthesiologica Scandinavica*, *64*(5), 579–591. https://doi.org/10.1111/aas.13553

Lai, B. yong, Liang, N., Cao, H. juan, Yang, G. yan, Jia, L. yan, Hu, R. xue, Lu, C. li, Zhao, N. qi, Fang, S. nan, Liu, X. han, Zhang, Y. jing, Fei, Y. tong, Wu, D. rong, & Liu, J. ping. (2018). Pediatric Tui Na for acute diarrhea in children under 5 years old: A systematic review and meta-analysis of randomized clinical trials. *Complementary Therapies in Medicine*, *41*(PG-10-22), 10–22. https://doi.org/10.1016/j.ctim.2018.08.011

Lang, B., Zhang, L., Zhang, W., Lin, Y., Fu, Y., & Chen, S. (2020). A comparative evaluation of dexmedetomidine and midazolam in pediatric sedation: A meta-analysis of randomized controlled trials with trial sequential analysis. *CNS Neuroscience and Therapeutics*, *26*(8), 862–875. https://doi.org/10.1111/cns.13377

Lee, H. A., Kawakami, H., Mihara, T., Sato, H., & Goto, T. (2021). Impact of anesthetic agents on the amount of bleeding during dilatation and evacuation: A systematic review and meta-analysis. *PLoS ONE*, *16*(12 December), e0261494. https://doi.org/10.1371/journal.pone.0261494

Lee, K. W., Devaraj, N. K., Ching, S. M., Veettil, S. K., Hoo, F. K., Deuraseh, I., & Soo, M. J. (2021). Effect of sglt-2 inhibitors on non-alcoholic fatty liver disease among patients with type 2 diabetes mellitus: Systematic review with meta-analysis and trial sequential analysis of randomized clinical trials. *Oman Medical Journal*, *36*(3), e273. https://doi.org/10.5001/OMJ.2021.62

Lee, K. W., Loh, H. C., Ching, S. M., Devaraj, N. K., & Hoo, F. K. (2020). Effects of vegetarian diets on blood pressure lowering: A systematic review with meta-analysis and trial sequential analysis. *Nutrients*, *12*(6). https://doi.org/10.3390/nu12061604

Lee, L. L., Huang, S. F., Lai, P. C., & Huang, Y. T. (2020). Effect of exercise on slowing breastfeeding-induced bone loss: A meta-analysis and trial sequential analysis. *Journal of Obstetrics and Gynaecology Research*, *46*(9), 1790–1800. https://doi.org/10.1111/jog.14346

Lee, M., Kim, H., Lee, C., & Kang, H. (2021). Effect of intravenous dexmedetomidine and remifentanil on neonatal outcomes after caesarean section under general anaesthesia: A systematic review and meta-Analysis. *European Journal of Anaesthesiology*, *38*(10), 1085–1095. https://doi.org/10.1097/EJA.0000000000001558

Lepot, A., Elia, N., Tramèr, M. R., & Rehberg, B. (2021). Preventing pain after breast surgery: A systematic review with meta-analyses and trial-sequential analyses. *European Journal of Pain (United Kingdom)*, *25*(1), 5–22. https://doi.org/10.1002/ejp.1648

Li, C., Zhao, Q., Yang, K., Jiang, L., & Yu, J. (2019). Thromboelastography or rotational thromboelastometry for bleeding management in adults undergoing cardiac surgery: A systematic review with meta-analysis and trial sequential analysis. *Journal of Thoracic Disease*, *11*(4), 1170–1181. https://doi.org/10.21037/jtd.2019.04.39
Li, J. L., Fan, G. Y., Liu, Y. J., Zeng, Z. H., Huang, J. J., Yang, Z. M., & Meng, X. Y. (2018). Long-term efficacy of maintenance therapy for multiple myeloma: A quantitative synthesis of 22 randomized controlled trials. *Frontiers in Pharmacology*, *9*(APR), 430. https://doi.org/10.3389/fphar.2018.00430
Li, J., Liu, D., Wu, J., Fan, X., & Dong, Q. (2018). Dapoxetine for the treatment of premature ejaculation: A meta-analysis of randomized controlled trials with trial sequential analysis. *Annals of Saudi Medicine*, *38*(5), 366–375. https://doi.org/10.5144/0256-4947.2018.366
Li, L., Zhang, Y., Jia, L., Jia, D., Faramand, A., Chong, W., Fang, Y., Ma, L., & Fang, F. (2020). Levetiracetam versus phenytoin for the treatment of established status epilepticus: A systematic review and meta-analysis of randomized controlled trials. *Seizure*, *78*(PG-43-48), 43–48. https://doi.org/10.1016/j.seizure.2020.03.002
Li, L., Zhang, Y., Wang, P., Chong, W., Hai, Y., Xu, P., & Fang, F. (2021). Conservative versus liberal oxygen therapy for acutely ill medical patients: A systematic review and meta-analysis. *International Journal of Nursing Studies*, *118*(PG-103924), 103924. https://doi.org/10.1016/j.ijnurstu.2021.103924
Li, S., Huang, M., Wu, G., Huang, W., Huang, Z., Yang, X., Ou, J., Wei, Q., Liu, C., & Yu, S. (2020). Efficacy of Chinese Herbal Formula Sini Zuojin Decoction in Treating Gastroesophageal Reflux Disease: Clinical Evidence and Potential Mechanisms. *Frontiers in Pharmacology*, *11*(PG-76), 76. https://doi.org/10.3389/fphar.2020.00076
Li, S., Liu, D., Chen, Z., Wei, S., Xu, W., Li, X., & Wei, Q. (2021). Comparative efficacy and safety of four classical prescriptions for clearing damp-heat recommended by clinical guidelines in treating rheumatoid arthritis: A network meta-analysis. *Annals of Palliative Medicine*, *10*(7), 7298–7328. https://doi.org/10.21037/apm-21-445
Li, T., Li, X., Huang, F., Tian, Q., Fan, Z. Y., & Wu, S. (2021). Clinical Efficacy and Safety of Acupressure on Low Back Pain: A Systematic Review and Meta-Analysis. *Evidence-Based Complementary and Alternative Medicine*, *2021*(PG-8862399), 8862399. https://doi.org/10.1155/2021/8862399
Li, W., Xu, F., Huang, R., Fan, W., Fu, C., Xu, L., Wang, X., Lu, H., & Li, Y. (2021). Xueshuantong Injection in Treating Deep Venous Thrombosis: A Systematic Review and Trial Sequential Analysis. *Evidence-Based Complementary and Alternative Medicine*, *2021*(PG-6622925), 6622925. https://doi.org/10.1155/2021/6622925
Li, X., Liu, C., Mao, Z., Li, Q., & Zhou, F. (2021). Timing of renal replacement therapy initiation for acute kidney injury in critically ill patients: a systematic review of randomized clinical trials with meta-analysis and trial sequential analysis. *Critical Care*, *25*(1), 15. https://doi.org/10.1186/s13054-020-03451-y
Li, X., Liu, C., Mao, Z., Qi, S., Song, R., & Zhou, F. (2021). Effectiveness of polymyxin B-immobilized hemoperfusion against sepsis and septic shock: A systematic review and meta-analysis. *Journal of Critical Care*, *63*(PG-187-195), 187–195. https://doi.org/10.1016/j.jcrc.2020.09.007

Li, Y., Liu, H., Peng, W., & Song, Z. (2018). Nicorandil improves clinical outcomes in patients with stable angina pectoris requiring PCI: a systematic review and meta-analysis of 14 randomized trials. *Expert Review of Clinical Pharmacology*, *11*(9), 855–865. https://doi.org/10.1080/17512433.2018.1508342

Li, Y., & Zhang, B. (2020). Effects of anesthesia depth on postoperative cognitive function and inflammation: A systematic review and meta-analysis. *Minerva Anestesiologica*, *86*(9), 965–973. https://doi.org/10.23736/S0375-9393.20.14251-2

Li, Y., Shen, Y., Tang, T., Tang, Z., Song, W., Yang, Z., Zhang, X., Wang, M., Bai, X., & Liang, T. (2020). Oncolytic virus combined with traditional treatment versus traditional treatment alone in patients with cancer: a meta-analysis. *International Journal of Clinical Oncology*, *25*(11), 1901–1913. https://doi.org/10.1007/s10147-020-01760-4

Lian, X. J., Huang, D. Z., Cao, Y. S., Wei, Y. X., Lian, Z. Z., Qin, T. H., He, P. C., Liu, Y. H., & Wang, S. H. (2019). Reevaluating the Role of Corticosteroids in Septic Shock: An Updated Meta-Analysis of Randomized Controlled Trials. *BioMed Research International*, *2019*(PG-3175047), 3175047. https://doi.org/10.1155/2019/3175047

Liang, N., Kong, D. Z., Lu, C. L., Ma, S. S., Li, Y. Q., Nikolova, D., Jakobsen, J. C., Gluud, C., & Liu, J. P. (2019). Radix sophorae flavescentis versus other drugs or herbs for chronic hepatitis B. *Cochrane Database of Systematic Reviews*, *2019*(6), CD013106. https://doi.org/10.1002/14651858.CD013106.pub2

Liang, N., Kong, D. Z., Ma, S. S., Lu, C. L., Yang, M., Feng, L. Da, Shen, C., Diao, R. H., Cui, L. J., Lu, X. Y., Nikolova, D., Jakobsen, J. C., Gluud, C., & Liu, J. P. (2019). Radix Sophorae flavescentis versus no intervention or placebo for chronic hepatitis B. *Cochrane Database of Systematic Reviews*, *2019*(4), CD013089. https://doi.org/10.1002/14651858.CD013089.pub2

Liang, S. B., Lai, B. Y., Cao, H. J., Cai, Q. H., Bai, X., Li, J., Zhang, Y. P., Chi, Y., Robinson, N., & Liu, J. P. (2020). Pediatric tuina for the treatment of anorexia in children under 14 years: a systematic review and meta-analysis of randomized controlled trials. *Complementary Therapies in Medicine*, *51*(PG-102411), 102411. https://doi.org/10.1016/j.ctim.2020.102411

Liao, A. H. W., Yeoh, S. R., Lin, Y. C., Lam, F., Chen, T. L., & Chen, C. Y. (2019). Lidocaine lubricants for intubation-related complications: a systematic review and meta-analysis. *Canadian Journal of Anesthesia*, *66*(10), 1221–1239. https://doi.org/10.1007/s12630-019-01408-6

Lim, B. L., Lee, W. F., Ng, W. M., Situ, W., Loo, K. V., Man Goh, C. J., & Chan, W. L. (2022). Benefits and safety of transdermal glyceryl trinitrate in acute stroke: A systematic review and meta-analysis of randomized trials. *Academic Emergency Medicine*, *29*(6), 772–788. https://doi.org/10.1111/acem.14408

Lin, C., Tu, H., Jie, Z., Zhou, X., & Li, C. (2021). Effect of Dexmedetomidine on Delirium in Elderly Surgical Patients: A Meta-analysis of Randomized Controlled Trials. *Annals of Pharmacotherapy*, *55*(5), 624–636. https://doi.org/10.1177/1060028020951954

Lin, M. H., Lee, C. H., Lin, C., Zou, Y. F., Lu, C. H., Hsieh, C. H., & Lee, C. H. (2019). Low-dose aspirin for the primary prevention of cardiovascular disease in diabetic individuals: A meta-analysis of randomized control trials and trial sequential analysis. *Journal of Clinical Medicine*, *8*(5). https://doi.org/10.3390/jcm8050609

Lin, P., Zhao, Y., Li, X., Jiang, F., & Liang, Z. (2021). Decreased mortality in acute respiratory distress syndrome patients treated with corticosteroids: an updated meta-analysis of randomized clinical trials with trial sequential analysis. *Critical Care*, *25*(1), 122. https://doi.org/10.1186/s13054-021-03546-0
Ling, X., Sun, X., Kong, H., Peng, S., Yu, Z., Wen, J., & Yuan, B. (2021). Chinese Herbal Medicine for the Treatment of Children and Adolescents With Refractory Mycoplasma Pneumoniae Pneumonia: A Systematic Review and a Meta-Analysis. *Frontiers in Pharmacology*, *12*(PG-678631), 678631. https://doi.org/10.3389/fphar.2021.678631
Liu, C., Fu, Z., Jiang, J., Chi, K., Geng, X., Mao, Z., Song, C., Sun, G., Hong, Q., Cai, G., Chen, X., & Sun, X. (2021). Safety and Efficacy of Roxadustat for Anemia in Patients With Chronic Kidney Disease: A Meta-Analysis and Trial Sequential Analysis. *Frontiers in Medicine*, *8*(PG-724456), 724456. https://doi.org/10.3389/fmed.2021.724456
Liu, C., Lu, G., Wang, D., Lei, Y., Mao, Z., Hu, P., Hu, J., Liu, R., Han, D., & Zhou, F. (2019). Balanced crystalloids versus normal saline for fluid resuscitation in critically ill patients: A systematic review and meta-analysis with trial sequential analysis. *American Journal of Emergency Medicine*, *37*(11), 2072–2078. https://doi.org/10.1016/j.ajem.2019.02.045
Liu, C., Mao, Z., Kang, H., Hu, X., Jiang, S., Hu, P., Hu, J., & Zhou, F. (2018). Comparison between the long-axis/in-plane and short-axis/out-of-plane approaches for ultrasound-guided vascular catheterization: An updated meta-analysis and trial sequential analysis. *Therapeutics and Clinical Risk Management*, *14*(PG-331-340), 331–340. https://doi.org/10.2147/TCRM.S152908
Liu, L., Yang, Z., Lu, K., Yi, B., & Yang, Y. (2021). Liposomal Bupivacaine Single-Injection Compared With Continuous Local Anesthetic Nerve Blocks for Painful Surgeries: A Meta-Analysis of Randomized Controlled Trials and Trial Sequential Analysis. *Pain Practice*, *21*(1), 88–99. https://doi.org/10.1111/papr.12927
Liu, Q., Shi, Z., Zhang, T., Jiang, T., Luo, X., Su, X., Yang, Y., & Wei, W. (2022). Efficacy and Safety of Chinese Herbal Medicine Xiao Yao San in Functional Gastrointestinal Disorders: A meta-Analysis and Trial Sequential Analysis of Randomized Controlled Trials. *Frontiers in Pharmacology*, *12*(PG-821802), 821802. https://doi.org/10.3389/fphar.2021.821802
Liu, W., Zhang, M., Chen, G., Li, Z., & Wei, F. (2020). Drug-Coated Balloon for de Novo Coronary Artery Lesions: A Systematic Review and Trial Sequential Meta-analysis of Randomized Controlled Trials. *Cardiovascular Therapeutics*, *2020*(PG-4158363), 4158363. https://doi.org/10.1155/2020/4158363
Liu, X., Wang, Y., & Wang, S. (2022). The efficacy of psychological interventions for depressed primary caregivers of patients with Alzheimer's disease: A systematic review and meta-analysis. *Journal of Nursing Scholarship*, *54*(3), 355–366. https://doi.org/10.1111/jnu.12742
Liu, X., Shi, W., Liu, Z., Shi, S., Ke, C., Zhang, P., Tan, Z., & Zhang, W. (2020). Effects of acupuncture on Luteinized Unruptured Follicle Syndrome: A meta-analysis of randomized controlled trials. *Complementary Therapies in Medicine*, *49*(PG-102319), 102319. https://doi.org/10.1016/j.ctim.2020.102319

Liu, Y., Liang, F., Liu, X., Shao, X., Jiang, N., & Gan, X. (2018). Dexmedetomidine Reduces Perioperative Opioid Consumption and Postoperative Pain Intensity in Neurosurgery: A Meta-analysis. *Journal of Neurosurgical Anesthesiology*, *30*(2), 146–155. https://doi.org/10.1097/ANA.0000000000000403
Loh, H. C., Lim, R., Lee, K. W., Ooi, C. Y., Chuan, D. R., Looi, I., Kah Hay, Y., & Abdul Karim Khan, N. (2021). Effects of vitamin e on stroke: A systematic review with meta-analysis and trial sequential analysis. *Stroke and Vascular Neurology*, *6*(1), 109–120. https://doi.org/10.1136/svn-2020-000519
López-Cano, M., Kraft, M., Curell, A., Puig-Asensio, M., Balibrea, J., Armengol-Carrasco, M., & García-Alamino, J. M. (2019). Use of Topical Antibiotics before Primary Incision Closure to Prevent Surgical Site Infection: A Meta-Analysis. *Surgical Infections*, *20*(4), 261–270. https://doi.org/10.1089/sur.2018.279
López-Cano, M., Kraft, M., Curell, A., Puig-Asensio, M., Balibrea, J., Armengol-Carrasco, M., & García-Alamino, J. M. (2019). A Meta-analysis of Prophylaxis of Surgical Site Infections with Topical Application of Povidone Iodine Before Primary Closure. *World Journal of Surgery*, *43*(2), 374–384. https://doi.org/10.1007/s00268-018-4798-0
López-Pacheco, A., Soto-Peñaloza, D., Gómez, M., Peñarrocha-Oltra, D., & Alarcón, M. A. (2021). Socket seal surgery techniques in the esthetic zone: a systematic review with meta-analysis and trial sequential analysis of randomized clinical trials. *International Journal of Implant Dentistry*, *7*(1), 13. https://doi.org/10.1186/s40729-021-00294-2
Lu, J. W., Huang, Y. W., & Chen, T. L. (2021). Efficacy and safety of adalimumab in hidradenitis suppurativa: A systematic review and meta-analysis of randomized controlled trials. *Medicine (United States)*, *100*(22), E26190. https://doi.org/10.1097/MD.0000000000026190
Lu, X., Han, W., Gao, Y. X., Guo, S. G., Yu, S. Y., Yu, X. Z., Zhu, H. D., & Li, Y. (2021). Effi cacy and safety of corticosteroids in immunocompetent patients with septic shock. *World Journal of Emergency Medicine*, *12*(2), 124–130. https://doi.org/10.5847/WJEM.J.1920-8642.2021.02.007
Lu, Y., Li, Y. W., Wang, L., Lydic, R., Baghdoyan, H. A., Shi, X. Y., & Zhang, H. (2019). Promoting sleep and circadian health may prevent postoperative delirium: A systematic review and meta-analysis of randomized clinical trials. *Sleep Medicine Reviews*, *48*(PG-101207), 101207. https://doi.org/10.1016/j.smrv.2019.08.001
Lu, Y., Zhang, H., Teng, F., Xia, W. J., Sun, G. X., & Wen, A. Q. (2018). Early Goal-Directed Therapy in Severe Sepsis and Septic Shock: A Meta-Analysis and Trial Sequential Analysis of Randomized Controlled Trials. *Journal of Intensive Care Medicine*, *33*(5), 296–309. https://doi.org/10.1177/0885066616671710
Lu, Z., Chang, W., Meng, S., Xue, M., Xie, J., Xu, J., Qiu, H., Yang, Y., & Guo, F. (2020). The Effect of High-Flow Nasal Oxygen Therapy on Postoperative Pulmonary Complications and Hospital Length of Stay in Postoperative Patients: A Systematic Review and Meta-Analysis. *Journal of Intensive Care Medicine*, *35*(10), 1129–1140. https://doi.org/10.1177/0885066618817718
Luís, Â., Domingues, F., & Pereira, L. (2018). Metabolic changes after licorice consumption: A systematic review with meta-analysis and trial sequential analysis of clinical trials. *Phytomedicine*, *39*(PG-17-24), 17–24. https://doi.org/10.1016/j.phymed.2017.12.010
Lundstrøm, L. H., Duez, C. H. V., Nørskov, A. K., Rosenstock, C. V., Thomsen, J. L., Møller, A. M., Strande, S., & Wetterslev, J. (2018). Effects of avoidance or use of neuromuscular blocking agents on outcomes in

tracheal intubation: a Cochrane systematic review. *British Journal of Anaesthesia*, *120*(6), 1381–1393. https://doi.org/10.1016/j.bja.2017.11.106

Luvira, V., Satitkarnmanee, E., Pugkhem, A., Kietpeerakool, C., Lumbiganon, P., & Pattanittum, P. (2021). Postoperative adjuvant chemotherapy for resectable cholangiocarcinoma. *Cochrane Database of Systematic Reviews*, *2021*(9). https://doi.org/10.1002/14651858.CD012814.pub2

Lyu, Q. Q., Chen, Q. H., Zheng, R. Q., Yu, J. Q., & Gu, X. H. (2020). Effect of Low-Dose Hydrocortisone Therapy in Adult Patients With Septic Shock: A Meta-Analysis With Trial Sequential Analysis of Randomized Controlled Trials. *Journal of Intensive Care Medicine*, *35*(10), 971–983. https://doi.org/10.1177/0885066618803062

Ma, S., Xu, C., Liu, S., Sun, X., Li, R., Mao, M., Feng, S., & Wang, X. (2021). Efficacy and safety of systematic corticosteroids among severe COVID-19 patients: a systematic review and meta-analysis of randomized controlled trials. *Signal Transduction and Targeted Therapy*, *6*(1), 83. https://doi.org/10.1038/s41392-021-00521-7

Maagaard, M., Karlsson, W. K., Ovesen, C., Gluud, C., & Jakobsen, J. C. (2021). Interventions for altering blood pressure in people with acute subarachnoid haemorrhage. *Cochrane Database of Systematic Reviews*, *2021*(11), CD013096. https://doi.org/10.1002/14651858.CD013096.pub2

Maagaard, M., Nielsen, E. E., Sethi, N. J., Liang, N., Yang, S. H., Gluud, C., & Jakobsen, J. C. (2022). Ivabradine added to usual care in patients with heart failure: a systematic review with meta-analysis and trial sequential analysis. *BMJ Evidence-Based Medicine*, *27*(4), 224–234. https://doi.org/10.1136/bmjebm-2021-111724

Maagaard, M., Nielsen, E. E., Sethi, N. J., Ning, L., Yang, S. H., Gluud, C., & Jakobsen, J. C. (2020). Effects of adding ivabradine to usual care in patients with angina pectoris: a systematic review of randomised clinical trials with meta-analysis and Trial Sequential Analysis. *Open Heart*, *7*(2). https://doi.org/10.1136/openhrt-2020-001288

Machado, F. C., Vieira, J. E., De Orange, F. A., & Ashmawi, H. A. (2019). Intraoperative Methadone Reduces Pain and Opioid Consumption in Acute Postoperative Pain: A Systematic Review and Meta-Analysis. *Anesthesia and Analgesia*, *129*(6), 1723–1732. https://doi.org/10.1213/ANE.0000000000004404

Mahmoud, A. N., Gad, M. M., Elgendy, A. Y., Elgendy, I. Y., & Bavry, A. A. (2019). Efficacy and safety of aspirin for primary prevention of cardiovascular events: A meta-analysis and trial sequential analysis of randomized controlled trials. *European Heart Journal*, *40*(7), 607–617. https://doi.org/10.1093/eurheartj/ehy813

Makkar, J. K., Singh, N. P., Bhatia, N., Samra, T., & Singh, P. M. (2021). Fascia iliaca block for hip fractures in the emergency department: meta-analysis with trial sequential analysis. *American Journal of Emergency Medicine*, *50*(PG-654-660), 654–660. https://doi.org/10.1016/j.ajem.2021.09.038

Makrgeorgou, A., Leonardi-Bee, J., Bath-Hextall, F. J., Murrell, D. F., Tang, M. L. K., Roberts, A., & Boyle, R. J. (2018). Probiotics for treating eczema. *Cochrane Database of Systematic Reviews*, *2018*(11), CD006135. https://doi.org/10.1002/14651858.CD006135.pub3

Mallama, M., Valencia, A., Rijs, K., Rietdijk, W. J. R., Klimek, M., & Calvache, J. A. (2021). A systematic review and trial sequential analysis of intravenous vs. oral peri-operative paracetamol. *Anaesthesia*, *76*(2), 270–276. https://doi.org/10.1111/anae.15163

Manuel, L. C., Lidia, A. M. D., Pereira, J. A., Manuel, A. C., & Josep, M. G. A. (2018). Balancing mesh-related complications and benefits in primary ventral and incisional hernia surgery. A meta-analysis and trial sequential analysis. *PLoS ONE*, *13*(6), e0197813. https://doi.org/10.1371/journal.pone.0197813

Maraolo, A. E., Crispo, A., Piezzo, M., Di Gennaro, P., Vitale, M. G., Mallardo, D., Ametrano, L., Celentano, E., Cuomo, A., Ascierto, P. A., & Cascella, M. (2021). The use of tocilizumab in patients with covid-19: A systematic review, meta-analysis and trial sequential analysis of randomized controlled studies. *Journal of Clinical Medicine*, *10*(21). https://doi.org/10.3390/jcm10214935

Marker, S., Barbateskovic, M., Perner, A., Wetterslev, J., Jakobsen, J. C., Krag, M., Granholm, A., Anthon, C. T., & Møller, M. H. (2020). Prophylactic use of acid suppressants in adult acutely ill hospitalised patients: A systematic review with meta-analysis and trial sequential analysis. *Acta Anaesthesiologica Scandinavica*, *64*(6), 714–728. https://doi.org/10.1111/aas.13568

Marsman, M. S., Wetterslev, J., Jahrome, A. K., Gluud, C., Moll, F. L., Keus, F., & Koning, G. G. (2021). Carotid endarterectomy with patch angioplasty versus primary closure in patients with symptomatic and significant stenosis: a systematic review with meta-analyses and trial sequential analysis of randomized clinical trials. *Systematic Reviews*, *10*(1), 139. https://doi.org/10.1186/s13643-021-01692-8

Martí-Carvajal, A. J., Valli, C., Martí-Amarista, C. E., Solà, I., Martí-Fàbregas, J., & Bonfill Cosp, X. (2020). Citicoline for treating people with acute ischemic stroke. *Cochrane Database of Systematic Reviews*, *2020*(8), CD013066. https://doi.org/10.1002/14651858.CD013066.pub2

McQuilten, Z. K., French, C. J., Nichol, A., Higgins, A., & Cooper, D. J. (2018). Effect of age of red cells for transfusion on patient outcomes: a systematic review and meta-analysis. *Transfusion Medicine Reviews*, *32*(2), 77–88. https://doi.org/10.1016/j.tmrv.2018.02.002

Meco, M., Montisci, A., Giustiniano, E., Greco, M., Pappalardo, F., Mammana, L., Panisi, P., Roscitano, C., Cirri, S., Donatelli, F., & Albano, G. (2020). Viscoelastic Blood Tests Use in Adult Cardiac Surgery: Meta-Analysis, Meta-Regression, and Trial Sequential Analysis. *Journal of Cardiothoracic and Vascular Anesthesia*, *34*(1), 119–127. https://doi.org/10.1053/j.jvca.2019.06.030

Meng, S. S., Chang, W., Lu, Z. H., Xie, J. F., Qiu, H. B., Yang, Y., & Guo, F. M. (2019). Effect of surfactant administration on outcomes of adult patients in acute respiratory distress syndrome: A meta-analysis of randomized controlled trials. *BMC Pulmonary Medicine*, *19*(1), 9. https://doi.org/10.1186/s12890-018-0761-y

Meyhoff, T. S., Møller, M. H., Hjortrup, P. B., Cronhjort, M., Perner, A., & Wetterslev, J. (2020). Lower vs Higher Fluid Volumes During Initial Management of Sepsis: A Systematic Review With Meta-Analysis and Trial Sequential Analysis. *Chest*, *157*(6), 1478–1496. https://doi.org/10.1016/j.chest.2019.11.050

Meza Mauricio, J., Furquim, C. P., Bustillos-Torrez, W., Soto-Peñaloza, D., Peñarrocha-Oltra, D., Retamal-Valdes, B., & Faveri, M. (2021). Does enamel matrix derivative application provide additional clinical

benefits in the treatment of maxillary Miller class I and II gingival recession? A systematic review and meta-analysis. *Clinical Oral Investigations*, *25*(4), 1613–1626. https://doi.org/10.1007/s00784-021-03782-2

Miao, S., Shi, M., Zou, L., & Wang, G. (2018). Effect of intrathecal dexmedetomidine on preventing shivering in cesarean section after spinal anesthesia: A meta-analysis and trial sequential analysis. *Drug Design, Development and Therapy*, *12*(PG-3775-3783), 3775–3783. https://doi.org/10.2147/DDDT.S178665

Michelet, D., Brasher, C., Horlin, A. L., Bellon, M., Julien-Marsollier, F., Vacher, T., Pontone, S., & Dahmani, S. (2018). Ketamine for chronic non-cancer pain: A meta-analysis and trial sequential analysis of randomized controlled trials. *European Journal of Pain (United Kingdom)*, *22*(4), 632–646. https://doi.org/10.1002/ejp.1153

Migliavaca, C. B., Stein, C., Colpani, V., Eibel, B., Bgeginski, R., Simões, M. V., Rohde, L. E., & Falavigna, M. (2020). High-dose versus low-dose angiotensin converting enzyme inhibitors in heart failure: Systematic review and meta-analysis. *Open Heart*, *7*(2). https://doi.org/10.1136/openhrt-2019-001228

Miles, L. F., Litton, E., Imberger, G., & Story, D. (2019). Intravenous iron therapy for non-anaemic, iron-deficient adults. *Cochrane Database of Systematic Reviews*, *2019*(12), CD013084. https://doi.org/10.1002/14651858.CD013084.pub2

Mohamedahmed, A. Y. Y., Zaman, S., Stonelake, S., Ahmad, A. N., Datta, U., Hajibandeh, S., & Hajibandeh, S. (2021). Incision and drainage of cutaneous abscess with or without cavity packing: a systematic review, meta-analysis, and trial sequential analysis of randomised controlled trials. *Langenbeck's Archives of Surgery*, *406*(4), 981–991. https://doi.org/10.1007/s00423-020-01941-9

Moreira, F. T., Palomba, H., De Freitas Chaves, R. C., Bouman, C., Schultz, M. J., & Neto, A. S. (2018). Early versus delayed initiation of renal replacement therapy for acute kidney injury: An updated systematic review, meta-analysis, meta-regression and trial sequential analysis of randomized controlled trials. *Revista Brasileira de Terapia Intensiva*, *30*(3), 376–384. https://doi.org/10.5935/0103-507X.20180054

Munir, M. B., Osman, K., Saleem, M., Patel, K., & Balla, S. (2019). Trial Sequential Analysis Comparing Bleeding and Major Adverse Cardiovascular Events in Patients with Atrial Fibrillation and Acute Coronary Syndrome on Dual versus Triple Therapy. *Cureus*, *11*(6 PG-e4880), e4880. https://doi.org/10.7759/cureus.4880

Na, W., Shen, H., Li, Y., & Qu, D. (2021). Hydrocortisone, ascorbic acid, and thiamine (HAT) for sepsis and septic shock: a meta-analysis with sequential trial analysis. *Journal of Intensive Care*, *9*(1), 75. https://doi.org/10.1186/s40560-021-00589-x

Nagendrababu, V., Abbott, P. V., Pulikkotil, S. J., Veettil, S. K., & Dummer, P. M. H. (2021). Comparing the anaesthetic efficacy of 1.8 mL and 3.6 mL of anaesthetic solution for inferior alveolar nerve blocks for teeth with irreversible pulpitis: a systematic review and meta-analysis with trial sequential analysis. *International Endodontic Journal*, *54*(3), 331–342. https://doi.org/10.1111/iej.13428

Nagendrababu, V., Aly Ahmed, H. M., Pulikkotil, S. J., Veettil, S. K., Dharmarajan, L., & Setzer, F. C. (2019). Anesthetic Efficacy of Gow-Gates, Vazirani-Akinosi, and Mental Incisive Nerve Blocks for Treatment of Symptomatic Irreversible Pulpitis: A Systematic Review and Meta-analysis with Trial Sequential Analysis. *Journal of Endodontics*, *45*(10), 1175-1183.e3. https://doi.org/10.1016/j.joen.2019.06.008

Nagendrababu, V., Pulikkotil, S. J., Veettil, S. K., Jinatongthai, P., & Gutmann, J. L. (2019). Efficacy of Biodentine and Mineral Trioxide Aggregate in Primary Molar Pulpotomies—A Systematic Review and Meta-Analysis With Trial Sequential Analysis of Randomized Clinical Trials. *Journal of Evidence-Based Dental Practice*, *19*(1), 17–27. https://doi.org/10.1016/j.jebdp.2018.05.002

Nagendrababu, V., Pulikkotil, S. J., Veettil, S. K., Teerawattanapong, N., & Setzer, F. C. (2018). Effect of Nonsteroidal Anti-inflammatory Drug as an Oral Premedication on the Anesthetic Success of Inferior Alveolar Nerve Block in Treatment of Irreversible Pulpitis: A Systematic Review with Meta-analysis and Trial Sequential Analysis. *Journal of Endodontics*, *44*(6), 914-922.e2. https://doi.org/10.1016/j.joen.2018.02.017

Nagy, Á., Kim, J. H., Jeong, M. E., Heo, M. H., Putzu, A., Belletti, A., Biondi-Zoccai, G., & Landoni, G. (2019). Non-vitamin K oral anticoagulants for coronary or peripheral artery disease: a systematic review and meta-analysis of mortality and major bleeding. *Minerva Cardioangiologica*, *67*(6), 477–486. https://doi.org/10.23736/S0026-4725.19.05043-6

Nakajima, D., Kawakami, H., Mihara, T., Sato, H., & Goto, T. (2020). Effectiveness of intravenous lidocaine in preventing postoperative nausea and vomiting in pediatric patients: A systematic review and meta-analysis. *PLoS ONE*, *15*(1), e0227904. https://doi.org/10.1371/journal.pone.0227904

Namdari, M., Amdjadi, P., Bayat, A., Seifi, M., & Alzwghaibi, A. (2021). Comparison of the failure rate, bonding time and ARI score of two orthodontic bonding systems: Self-Etch Primer and Conventional Etching Primer: A systematic review and meta-analysis. *International Orthodontics*, *19*(4), 566–579. https://doi.org/10.1016/j.ortho.2021.09.001

Nath, S., Pulikkotil, S., Dharmarajan, L., Arunachalam, M., & Jing, K. (2020). Effect of locally delivered doxycycline as an adjunct to scaling and root planing in the treatment of periodontitis in smokers: A systematic review of randomized controlled trials with meta-analysis and trial sequential analysis. Dental Research Journal, 17(4), 235. https://doi.org/10.4103/1735-3327.292059

Nath, S., Shen, C., Koziarz, A., Banfield, L., Nowrouzi-Kia, B., Fava, M. A., & Hodge, W. G. (2021). Transepithelial versus Epithelium-off Corneal Collagen Cross-linking for Corneal Ectasia: A Systematic Review and Meta-analysis. *Ophthalmology*, *128*(8), 1150–1160. https://doi.org/10.1016/j.ophtha.2020.12.023

Navarese, E. P., Lansky, A. J., Kereiakes, D. J., Kubica, J., Gurbel, P. A., Gorog, D. A., Valgimigli, M., Curzen, N., Kandzari, D. E., Bonaca, M. P., Brouwer, M., Umińska, J., Jaguszewski, M. J., Raggi, P., Waksman, R., Leon, M. B., Wijns, W., & Andreotti, F. (2021). Cardiac mortality in patients randomised to elective coronary revascularisation plus medical therapy or medical therapy alone: a systematic review and meta-analysis. *European Heart Journal*, *42*(45), 4638–4651. https://doi.org/10.1093/eurheartj/ehab246

Ng, K. T., Shubash, C. J., & Chong, J. S. (2019). The effect of dexmedetomidine on delirium and agitation in patients in intensive care: systematic review and meta-analysis with trial sequential analysis. *Anaesthesia*, *74*(3), 380–392. https://doi.org/10.1111/anae.14472

Ng, K. T., Yap, J. L. L., Izham, I. N., Teoh, W. Y., Kwok, P. E., & Koh, W. J. (2020). The effect of intravenous magnesium on postoperative morphine consumption in noncardiac surgery: A systematic review and meta-analysis with trial sequential analysis. *European Journal of Anaesthesiology*, *37*(3), 212–223. https://doi.org/10.1097/EJA.0000000000001164

Ng, K. T., Chan, X. L., Tan, W., & Wang, C. Y. (2019). Levosimendan use in patients with preoperative low ejection fraction undergoing cardiac surgery: A systematic review with meta-analysis and trial sequential analysis. *Journal of Clinical Anesthesia*, *52*(PG-37-47), 37–47. https://doi.org/10.1016/j.jclinane.2018.08.019

Ng, K. T., Teoh, W. Y., & Khor, A. J. (2020). The effect of melatonin on delirium in hospitalised patients: A systematic review and meta-analyses with trial sequential analysis. *Journal of Clinical Anesthesia*, *59*(PG-74-81), 74–81. https://doi.org/10.1016/j.jclinane.2019.06.027

Ng, K. T., Tsia, A. K. V., & Chong, V. Y. L. (2019). Robotic Versus Conventional Laparoscopic Surgery for Colorectal Cancer: A Systematic Review and Meta-Analysis with Trial Sequential Analysis. *World Journal of Surgery*, *43*(4), 1146–1161. https://doi.org/10.1007/s00268-018-04896-7

Ni, Y. N., Wang, T., Liang, B. M., & Liang, Z. A. (2021). The Effect of Conservative Oxygen Therapy in Reducing Mortality in Critical Care Patients: A Meta-Analysis and Trial Sequential Analysis. *Frontiers in Medicine*, *8*(PG-738418), 738418. https://doi.org/10.3389/fmed.2021.738418

Nielsen, E. E., Feinberg, J. B., Bu, F. L., Hecht Olsen, M., Raymond, I., Steensgaard-Hansen, F., & Jakobsen, J. C. (2020). Beneficial and harmful effects of sacubitril/valsartan in patients with heart failure: A systematic review of randomised clinical trials with meta-analysis and trial sequential analysis. *Open Heart*, *7*(2). https://doi.org/10.1136/openhrt-2020-001294

Odor, P. M., Bampoe, S., Gilhooly, D., Creagh-Brown, B., & Ramani Moonesinghe, S. (2020). Perioperative interventions for prevention of postoperative pulmonary complications: Systematic review and meta-analysis. *The BMJ*, *368*(PG-m540), m540. https://doi.org/10.1136/bmj.m540

Okoli, G. N., Rabbani, R., Copstein, L., Al-Juboori, A., Askin, N., & Abou-Setta, A. M. (2021). Remdesivir for coronavirus disease 2019 (COVID-19): a systematic review with meta-analysis and trial sequential analysis of randomized controlled trials. *Infectious Diseases*, *53*(9), 691–699. https://doi.org/10.1080/23744235.2021.1923799

Onwochei, D. N., Van Ross, J., Singh, P. M., Salter, A., & Monks, D. T. (2019). Carbetocin reduces the need for additional uterotonics in elective caesarean delivery: a systematic review, meta-analysis and trial sequential analysis of randomised controlled trials. *International Journal of Obstetric Anesthesia*, *40*(PG-14-23), 14–23. https://doi.org/10.1016/j.ijoa.2019.06.007

Onwochei, D. N., Owolabi, A., Singh, P. M., & Monks, D. T. (2020). Carbetocin compared with oxytocin in non-elective Cesarean delivery: a systematic review, meta-analysis, and trial sequential analysis of randomized-controlled trials. *Canadian Journal of Anesthesia*, *67*(11), 1524–1534. https://doi.org/10.1007/s12630-020-01779-1

Onwochei, D., Nair, G., Young, B., & Desai, N. (2021). Conventional landmark palpation versus preprocedural ultrasound for neuraxial procedures in nonobstetric patients: A systematic review with meta-analysis and trial sequential analysis of randomised controlled trials. *European Journal of Anaesthesiology*, *38*(Suppl 2 PG-S73-S86), S73–S86. https://doi.org/10.1097/EJA.0000000000001525

Osman, M., Kheiri, B., Shigle, A. J., Saleem, M., Osman, K., Sengupta, P. P., & Moreland, J. A. (2019). Ticagrelor after pharmacological thrombolysis in patients with ST-segment elevation myocardial

infarctions: insight from a trial sequential analysis. *Journal of Thrombosis and Thrombolysis*, *48*(4), 661–667. https://doi.org/10.1007/s11239-019-01953-3

Osman, M., Saleem, M., Osman, K., Kheiri, B., Regner, S., Radaideh, Q., Moreland, J. A., Rao, S. V., & Kapadia, S. (2020). Radial versus femoral access for percutaneous coronary intervention in patients with ST-segment elevation myocardial infarction: Trial sequential analysis. *American Heart Journal*, *224*(PG-98-104), 98–104. https://doi.org/10.1016/j.ahj.2020.03.014

Ou, Q., Yu, Y., Li, A., Chen, J., Yu, T., Xu, X., Xie, X., Chen, Y., Lin, D., Zeng, Q., Zhang, Y., Tang, X., Yao, H., & Luo, B. (2020). Association of survival and genomic mutation signature with immunotherapy in patients with hepatocellular carcinoma. *Annals of Translational Medicine*, *8*(5), 230–230. https://doi.org/10.21037/atm.2020.01.32

Ou-Yang, L. J., Chen, P. H., Jhou, H. J., Su, V. Y. F., & Lee, C. H. (2020). Proportional assist ventilation versus pressure support ventilation for weaning from mechanical ventilation in adults: A meta-analysis and trial sequential analysis. *Critical Care*, *24*(1), 556. https://doi.org/10.1186/s13054-020-03251-4

Ouyang, X., Qu, R., Hu, B., Wang, Y., Yao, F., Lv, B., Sun, C., Deng, Y., & Chen, C. (2022). Is metoclopramide beneficial for the postpyloric placement of nasoenteric tubes? A systematic review and meta-analysis of randomized controlled trials. *Nutrition in Clinical Practice*, *37*(2), 316–327. https://doi.org/10.1002/ncp.10725

Pan, L., Zhang, T., Cao, H., Sun, H., & Liu, G. (2020). Ginsenoside Rg3 for chemotherapy-induced myelosuppression: A meta-analysis and systematic review. *Frontiers in Pharmacology*, *11*(PG-649), 649. https://doi.org/10.3389/fphar.2020.00649

Pan, L., Zhang, T., Sun, H., & Liu, G. (2019). Ginsenoside Rg3 (Shenyi Capsule) Combined with Chemotherapy for Digestive System Cancer in China: A Meta-Analysis and Systematic Review. *Evidence-Based Complementary and Alternative Medicine*, *2019*(PG-2417418), 2417418. https://doi.org/10.1155/2019/2417418

Park, S. K., Son, Y. G., Yoo, S., Lim, T., Kim, W. H., & Kim, J. T. (2018). Deep vs. moderate neuromuscular blockade during laparoscopic surgery A systematic review and meta-analysis. *European Journal of Anaesthesiology*, *35*(11), 867–875. https://doi.org/10.1097/EJA.0000000000000884

Patini, R., Spagnuolo, G., Guglielmi, F., Staderini, E., Simeone, M., Camodeca, A., & Gallenzi, P. (2020). Clinical Effects of Mercury in Conservative Dentistry: A Systematic Review, Meta-Analysis, and Trial Sequential Analysis of Randomized Controlled Trials. *International Journal of Dentistry*, *2020*(PG-8857238), 8857238. https://doi.org/10.1155/2020/8857238

Pedersen, S. S., Fabritius, M. L., Kongebro, E. K., & Meyhoff, C. S. (2021). Antioxidant treatment to reduce mortality and serious adverse events in adult surgical patients: A systematic review with meta-analysis and trial sequential analysis. *Acta Anaesthesiologica Scandinavica*, *65*(4), 438–450. https://doi.org/10.1111/aas.13752

Peng, K., Chen, W. R., Meng, X. W., Zhang, J., & Ji, F. H. (2018). Intra-articular dexmedetomidine in knee arthroscopy: A systematic review and meta-analysis. *Scientific Reports*, *8*(1), 4089. https://doi.org/10.1038/s41598-018-22482-8

Peng, K., Li, D., Applegate, R. L., Lubarsky, D. A., Ji, F. hai, & Liu, H. (2020). Effect of Dexmedetomidine on Cardiac Surgery-Associated Acute Kidney Injury: A Meta-Analysis With Trial Sequential Analysis of Randomized Controlled Trials. *Journal of Cardiothoracic and Vascular Anesthesia*, *34*(3), 603–613. https://doi.org/10.1053/j.jvca.2019.09.011

Peng, L., Li, L., Wang, P., Chong, W., Li, Y., Zha, X., Deng, H., Fan, H., & Zhang, Y. (2020). Association between Vitamin D supplementation and mortality in critically ill patients: A systematic review and meta-analysis of randomized clinical trials. *PLoS ONE*, *15*(12 December), e0243768. https://doi.org/10.1371/journal.pone.0243768

Pensier, J., de Jong, A., Hajjej, Z., Molinari, N., Carr, J., Belafia, F., Chanques, G., Futier, E., Azoulay, E., & Jaber, S. (2019). Effect of lung recruitment maneuver on oxygenation, physiological parameters and mortality in acute respiratory distress syndrome patients: a systematic review and meta-analysis. *Intensive Care Medicine*, *45*(12), 1691–1702. https://doi.org/10.1007/s00134-019-05821-9

Pensier, J., Deffontis, L., Rollé, A., Aarab, Y., Capdevila, M., Monet, C., Carr, J., Futier, E., Molinari, N., Jaber, S., & De Jong, A. (2022). Hydroxyethyl Starch for Fluid Management in Patients Undergoing Major Abdominal Surgery: A Systematic Review with Meta-analysis and Trial Sequential Analysis. *Anesthesia and Analgesia*, *134*(4), 686–695. https://doi.org/10.1213/ANE.0000000000005803

Pergialiotis, V., Daskalakis, G., Thomakos, N., Haidopoulos, D., Loutradis, D., & Rodolakis, A. (2019). Impact of vertical versus horizontal vaginal cuff closure on vaginal length following hysterectomy: a meta-analysis of randomized trials. *International Urogynecology Journal*, *30*(8), 1239–1245. https://doi.org/10.1007/s00192-019-03881-5

Pergialiotis, V., Mitsopoulou, D., Biliou, E., Bellos, I., Karagiannis, V., Papapanagiotou, A., Rodolakis, A., & Daskalakis, G. (2021). Cephalad-caudad versus transverse blunt expansion of the low transverse hysterotomy during cesarean delivery decreases maternal morbidity: a meta-analysis. *American Journal of Obstetrics and Gynecology*, *225*(2), 128.e1-128.e13. https://doi.org/10.1016/j.ajog.2021.04.231

Petersen, M. W., Perner, A., Ravn, F., Sjövall, F., & Møller, M. H. (2018). Untargeted antifungal therapy in adult patients with complicated intra-abdominal infection: a systematic review. *Acta Anaesthesiologica Scandinavica*, *62*(1), 6–18. https://doi.org/10.1111/aas.13031

Petersen, M. W., Perner, A., Jonsson, A. B., Bahador, M., Sjövall, F., & Møller, M. H. (2019). Empirical metronidazole for patients with severe bacterial infection: A systematic review with meta-analysis and trial sequential analysis. *Acta Anaesthesiologica Scandinavica*, *63*(6), 802–813. https://doi.org/10.1111/aas.13327

Piccoli, G. F., Mesquita, L. A., Stein, C., Aziz, M., Zoldan, M., Degobi, N. A. H., Spiazzi, B. F., Lopes Junior, G. L., Colpani, V., & Gerchman, F. (2021). Do GLP-1 Receptor Agonists Increase the Risk of Breast Cancer? A Systematic Review and Meta-analysis. *Journal of Clinical Endocrinology and Metabolism*, *106*(3), 912–921. https://doi.org/10.1210/clinem/dgaa891

Pinto, L. C., Falcetta, M. R., Rados, D. V., Leitão, C. B., & Gross, J. L. (2019). Glucagon-like peptide-1 receptor agonists and pancreatic cancer: a meta-analysis with trial sequential analysis. *Scientific Reports*, *9*(1), 2375. https://doi.org/10.1038/s41598-019-38956-2

Pinto, L. C., Rados, D. V., Barkan, S. S., Leitão, C. B., & Gross, J. L. (2018). Dipeptidyl peptidase-4 inhibitors, pancreatic cancer and acute pancreatitis: A meta-analysis with trial sequential analysis. *Scientific Reports*, *8*(1), 782. https://doi.org/10.1038/s41598-017-19055-6

Pinto, L. C., Rados, D. V., Remonti, L. R., Viana, L. V., Pulz, G. T., Carpena, M. P., Borges, R. P., Marobin, R., Beretta, M. V., Pedrollo, E. F., Londero, T. M., Machry, R., Janeczko, L., Moehlecke, M., Falcetta, M. R., Bauer, A. C., Silveiro, S. P., Gerchman, F., Rodrigues, T. C., … Leitão, C. B. (2020). Patient-centered Management of Type 2 Diabetes Mellitus Based on Specific Clinical Scenarios: Systematic Review, Meta-analysis and Trial Sequential Analysis. *Journal of Clinical Endocrinology and Metabolism*, *105*(11), 1–12. https://doi.org/10.1210/clinem/dgaa534

Pradelli, L., Mayer, K., Klek, S., Omar Alsaleh, A. J., Clark, R. A. C., Rosenthal, M. D., Heller, A. R., & Muscaritoli, M. (2020). ω-3 Fatty-Acid Enriched Parenteral Nutrition in Hospitalized Patients: Systematic Review With Meta-Analysis and Trial Sequential Analysis. *Journal of Parenteral and Enteral Nutrition*, *44*(1), 44–57. https://doi.org/10.1002/jpen.1672

Putzu, A., Clivio, S., Belletti, A., & Cassina, T. (2018). Perioperative levosimendan in cardiac surgery: A systematic review with meta-analysis and trial sequential analysis. *International Journal of Cardiology*, *251*(PG-22-31), 22–31. https://doi.org/10.1016/j.ijcard.2017.10.077

Putzu, A., de Carvalho e Silva, C. M. P. D., de Almeida, J. P., Belletti, A., Cassina, T., Landoni, G., & Hajjar, L. A. (2018). Perioperative statin therapy in cardiac and non-cardiac surgery: a systematic review and meta-analysis of randomized controlled trials. *Annals of Intensive Care*, *8*(1), 95. https://doi.org/10.1186/s13613-018-0441-3

Qiu, M., Ding, L., & Zhou, H. (2021). Effects of SGLT2 inhibitors on cardiovascular and renal outcomes in type 2 diabetes: A meta-analysis with trial sequential analysis. *Medicine (United States)*, *100*(10), E25121. https://doi.org/10.1097/MD.0000000000025121

Rados, D. V., Falcetta, M. R. R., Pinto, L. C., Leitão, C. B., & Gross, J. L. (2021). All-cause mortality and cardiovascular safety of basal insulin treatment in patients with type 2 diabetes mellitus: A systematic review with meta-analysis and trial sequential analysis. *Diabetes Research and Clinical Practice*, *173*(PG-108688), 108688. https://doi.org/10.1016/j.diabres.2021.108688

Rai, D., Tahir, M. W., Bandyopadhyay, D., Chowdhury, M., Kharsa, A., Pendala, V. S., Ali, H., Naidu, S. S., & Baibhav, B. (2021). Meta-Analysis and Trial Sequential Analysis of Randomized Controlled Trials for Multivessel PCI Versus Culprit Artery Only PCI in STEMI Without Cardiogenic Shock. *Current Problems in Cardiology*, *46*(3), 100646. https://doi.org/10.1016/j.cpcardiol.2020.100646

Ramstad, E., Storebø, O. J., Gerner, T., Krogh, H. B., Holmskov, M., Magnusson, F. L., Moreira-Maia, C. R., Skoog, M., Groth, C., Gillies, D., Zwi, M., Kirubakaran, R., Gluud, C., & Simonsen, E. (2018). Hallucinations and other psychotic symptoms in response to methylphenidate in children and adolescents with attention-deficit/hyperactivity disorder: a Cochrane systematic review with meta-analysis and trial sequential analysis # . *Scandinavian Journal of Child and Adolescent Psychiatry and Psychology*, *6*(1), 52–71. https://doi.org/10.21307/sjcapp-2018-003

Ravidà, A., Wang, I. C., Sammartino, G., Barootchi, S., Tattan, M., Troiano, G., Laino, L., Marenzi, G., Covani, U., & Wang, H. L. (2019). Prosthetic Rehabilitation of the Posterior Atrophic Maxilla, Short (≤6 mm) or Long (≥10 mm) Dental Implants? A Systematic Review, Meta-analysis, and Trial Sequential Analysis: Naples

Consensus Report Working Group A. *Implant Dentistry*, *28*(6), 590–602. https://doi.org/10.1097/ID.0000000000000919

Ren, Y., Wei, M., Liu, H., Wang, Y., Chen, H., Li, Z., Shi, W., & You, F. (2021). Efficacy and safety of dexmedetomidine as an adjuvant to local wound infiltration anaesthesia: A meta-analysis with trial sequential analysis of 23 randomised controlled trials. *International Wound Journal*, *18*(1), 32–48. https://doi.org/10.1111/iwj.13517

Reynolds, P. M., & MacLaren, R. (2019). Re-evaluating the Utility of Stress Ulcer Prophylaxis in the Critically Ill Patient: A Clinical Scenario-Based Meta-Analysis. *Pharmacotherapy*, *39*(3), 408–420. https://doi.org/10.1002/phar.2172

Riaz, I. Bin, Siddiqi, R., Islam, M., He, H., Riaz, A., Asghar, N., Naqvi, S. A. A., Warner, J. L., Murad, M. H., & Kohli, M. (2021). Adjuvant Tyrosine Kinase Inhibitors in Renal Cell Carcinoma: A Concluded Living Systematic Review and Meta-Analysis. *JCO Clinical Cancer Informatics*, *5*(5), 588–599. https://doi.org/10.1200/cci.21.00035

Riberholt, C. G., Wagner, V., Lindschou, J., Gluud, C., Mehlsen, J., & Møller, K. (2020). Early head-up mobilisation versus standard care for patients with severe acquired brain injury: A systematic review with meta-analysis and Trial Sequential Analysis. *PLoS ONE*, *15*(8 August), e0237136. https://doi.org/10.1371/journal.pone.0237136

Rijs, K., Mercier, F. J., Lucas, D. N., Rossaint, R., Klimek, M., & Heesen, M. (2020). Fluid loading therapy to prevent spinal hypotension in women undergoing elective caesarean section: Network meta-analysis, trial sequential analysis and meta-regression. *European Journal of Anaesthesiology*, *37*(12), 1126–1142. https://doi.org/10.1097/EJA.0000000000001371

Rios, S. A., Bravo, C. A., Weinreich, M., Olmedo, W., Villablanca, P., Villela, M. A., Ramakrishna, H., Hirji, S., Robles, O. A., Mahato, P., Gluud, C., Bhatt, D. L., & Jorde, U. P. (2018). Meta-Analysis and Trial Sequential Analysis Comparing Percutaneous Ventricular Assist Devices Versus Intra-Aortic Balloon Pump During High-Risk Percutaneous Coronary Intervention or Cardiogenic Shock. *American Journal of Cardiology*, *122*(8), 1330–1338. https://doi.org/10.1016/j.amjcard.2018.07.011

Rizos, E. C., Markozannes, G., Tsapas, A., Mantzoros, C. S., & Ntzani, E. E. (2021). Omega-3 supplementation and cardiovascular disease: Formulation-based systematic review and meta-analysis with trial sequential analysis. *Heart*, *107*(2), 150–158. https://doi.org/10.1136/heartjnl-2020-316780

Rojas, J., Srikumaran, U., & McFarland, E. G. (2021). Inconclusive evidence for the efficacy of tranexamic acid in reducing transfusions, postoperative infection or hematoma formation after primary shoulder arthroplasty: A meta-analysis with trial sequential analysis. *Shoulder and Elbow*, *13*(1), 38–50. https://doi.org/10.1177/1758573219896794

Rutherford, D., Massie, E. M., Worsley, C., & Wilson, M. S. J. (2021). Intraperitoneal local anaesthetic instillation versus no intraperitoneal local anaesthetic instillation for laparoscopic cholecystectomy. *Cochrane Database of Systematic Reviews*, *2021*(10). https://doi.org/10.1002/14651858.CD007337.pub4

Rygård, S. L., Jonsson, A. B., Madsen, M. B., Perner, A., Holst, L. B., Johansson, P. I., & Wetterslev, J. (2018). Effects of shorter versus longer storage time of transfused red blood cells in adult ICU patients: a

systematic review with meta-analysis and Trial Sequential Analysis. *Intensive Care Medicine*, *44*(2), 204–217. https://doi.org/10.1007/s00134-018-5069-0

Rygård, S. L., Butler, E., Granholm, A., Møller, M. H., Cohen, J., Finfer, S., Perner, A., Myburgh, J., Venkatesh, B., & Delaney, A. (2018). Low-dose corticosteroids for adult patients with septic shock: a systematic review with meta-analysis and trial sequential analysis. *Intensive Care Medicine*, *44*(7), 1003–1016. https://doi.org/10.1007/s00134-018-5197-6

Saito, M., Maruyama, K., Mihara, T., Hoshijima, H., Hirabayashi, G., Andoh, T., & Mayr, J. (2021). Comparison of polyurethane tracheal tube cuffs and conventional polyvinyl chloride tube cuff for prevention of ventilator-associated pneumonia: A systematic review with meta-analysis. *Medicine (United States)*, *100*(9), E24906. https://doi.org/10.1097/MD.0000000000024906

Sandven, I., Eritsland, J., & Abdelnoor, M. (2020). Remote ischemic conditioning in patients with acute coronary syndromes: A systematic review with meta-analysis and trial sequential analysis. *Clinical Epidemiology*, *12*(PG-595-605), 595–605. https://doi.org/10.2147/CLEP.S249785

Sanfilippo, F., La Via, L., Lanzafame, B., Dezio, V., Busalacchi, D., Messina, A., Ristagno, G., Pelosi, P., & Astuto, M. (2021). Targeted temperature management after cardiac arrest: A systematic review and meta-analysis with trial sequential analysis. *Journal of Clinical Medicine*, *10*(17). https://doi.org/10.3390/jcm10173943

Schnabel, A., Reichl, S. U., Weibel, S., Kranke, P., Zahn, P. K., Pogatzki-Zahn, E. M., & Meyer-Frieem, C. H. (2018). Efficacy and safety of dexmedetomidine in peripheral nerve blocks: A meta-analysis and trial sequential analysis. *European Journal of Anaesthesiology*, *35*(10), 745–758. https://doi.org/10.1097/EJA.0000000000000870

Schwimmbeck, F., Voellger, B., Chappell, D., & Eberhart, L. (2021). Hypertonic saline versus mannitol for traumatic brain injury: A systematic review and meta-analysis with trial sequential analysis. *Journal of Neurosurgical Anesthesiology*, *33*(1), 10–20. https://doi.org/10.1097/ANA.0000000000000644

Sethi, N. J., Nielsen, E. E., Safi, S., Feinberg, J., Gluud, C., & Jakobsen, J. C. (2018). Digoxin for atrial fibrillation and atrial flutter: A systematic review with meta-analysis and trial sequential analysis of randomised clinical trials. *PLoS ONE*, *13*(3), e0193924. https://doi.org/10.1371/journal.pone.0193924

Shah, A., Brunskill, S. J., Desborough, M. J., Doree, C., Trivella, M., & Stanworth, S. J. (2018). Transfusion of red blood cells stored for shorter versus longer duration for all conditions. *Cochrane Database of Systematic Reviews*, *2018*(12), CD010801. https://doi.org/10.1002/14651858.CD010801.pub3

Shah, A., Fisher, S. A., Wong, H., Roy, N. B., McKechnie, S., Doree, C., Litton, E., & Stanworth, S. J. (2019). Safety and efficacy of iron therapy on reducing red blood cell transfusion requirements and treating anaemia in critically ill adults: A systematic review with meta-analysis and trial sequential analysis. *Journal of Critical Care*, *49*(PG-162-171), 162–171. https://doi.org/10.1016/j.jcrc.2018.11.005

Shah, A., Palmer, A. J. R., Fisher, S. A., Rahman, S. M., Brunskill, S., Doree, C., Reid, J., Sugavanam, A., & Stanworth, S. J. (2018). What is the effect of perioperative intravenous iron therapy in patients undergoing non-elective surgery? A systematic review with meta-analysis and trial sequential analysis. *Perioperative Medicine*, *7*(1), 30. https://doi.org/10.1186/s13741-018-0109-4

Shah, U. J., Nguyen, D., Karuppiaah, N., Martin, J., & Sehmbi, H. (2021). Efficacy and safety of caudal dexmedetomidine in pediatric infra-umbilical surgery: A meta-analysis and trial-sequential analysis of randomized controlled trials. *Regional Anesthesia and Pain Medicine*, *46*(5), 422–432. https://doi.org/10.1136/rapm-2020-102024

Shao, S., Kang, H., Qian, Z., Wang, Y., & Tong, Z. (2021). Effect of different levels of PEEP on mortality in ICU patients without acute respiratory distress syndrome: systematic review and meta-analysis with trial sequential analysis. *Journal of Critical Care*, *65*(PG-246-258), 246–258. https://doi.org/10.1016/j.jcrc.2021.06.015

Shao, S., Kang, H., & Tong, Z. (2020). Early neuromuscular blocking agents for adults with acute respiratory distress syndrome: A systematic review, meta-analysis and meta-regression. *BMJ Open*, *10*(11), e037737. https://doi.org/10.1136/bmjopen-2020-037737

Sharma, S., Sharma, G., & Tyagi, S. (2021). Lidocaine lubricant jelly does not reduce pain perception during female urethral catheterization: A systematic review with meta-analysis and trial sequential analysis. *International Journal of Clinical Practice*, *75*(9), e14162. https://doi.org/10.1111/ijcp.14162

Shehata, N., Mistry, N., Da Costa, B. R., Pereira, T. V., Whitlock, R., Curley, G. F., Scott, D. A., Hare, G. M. T., Jüni, P., & Mazer, C. D. (2019). Restrictive compared with liberal red cell transfusion strategies in cardiac surgery: A meta-analysis. *European Heart Journal*, *40*(13), 1081–1088. https://doi.org/10.1093/eurheartj/ehy435

Shen, Q. hong, Li, H. fang, Zhou, X. yan, & Yuan, X. zhong. (2020). Dexmedetomidine in the prevention of postoperative delirium in elderly patients following non-cardiac surgery: A systematic review and meta-analysis. *Clinical and Experimental Pharmacology and Physiology*, *47*(8), 1333–1341. https://doi.org/10.1111/1440-1681.13312

Shen, Q. H., Li, H. F., Zhou, X., Lu, Y., & Yuan, X. Z. (2020). 5-HT 3 receptor antagonists for the prevention of perioperative shivering undergoing spinal anaesthesia: A systematic review and meta-Analysis of randomised controlled trials. *BMJ Open*, *10*(10), e038293. https://doi.org/10.1136/bmjopen-2020-038293

Sheng, Z., Cao, J. Y., Pang, Y. C., Xu, H. C., Chen, J. W., Yuan, J. H., Wang, R., Zhang, C. S., Wang, L. X., & Dong, J. (2019). Effects of lifestyle modification and anti-diabetic medicine on prediabetes progress: A systematic review and meta-analysis. *Frontiers in Endocrinology*, *10*(JULY), 455. https://doi.org/10.3389/fendo.2019.00455

Shi, R., Li, Z. H., Chen, D., Wu, Q. C., Zhou, X. L., & Tie, H. T. (2018). Sole and combined vitamin C supplementation can prevent postoperative atrial fibrillation after cardiac surgery: A systematic review and meta-analysis of randomized controlled trials. *Clinical Cardiology*, *41*(6), 871–878. https://doi.org/10.1002/clc.22951

Shih, Y. W., Su, J. Y., Kung, Y. S., Lin, Y. H., To Anh, D. T., Ridwan, E. S., & Tsai, H. T. (2021). Effectiveness of Acupuncture in Relieving Chemotherapy-induced Leukopenia in Patients With Breast Cancer: A Systematic Review With A Meta-Analysis and Trial Sequential Analysis. *Integrative Cancer Therapies*, *20*(PG-15347354211063884), 15347354211063884. https://doi.org/10.1177/15347354211063884

Shui, M., Xue, Z., Miao, X., Wei, C., & Wu, A. (2021). Intravenous versus inhalational maintenance of anesthesia for quality of recovery in adult patients undergoing non-cardiac surgery: A systematic review

with meta-analysis and trial sequential analysis. *PLoS ONE*, *16*(7 July), e0254271. https://doi.org/10.1371/journal.pone.0254271

Si, X. B., Zhang, X. M., Wang, S., Lan, Y., Zhang, S., & Huo, L. Y. (2019). Allicin as add-on therapy for Helicobacter pylori infection: A systematic review and meta-analysis. *World Journal of Gastroenterology*, *25*(39), 6025–6040. https://doi.org/10.3748/wjg.v25.i39.6025

Simancas-Racines, D., Arevalo-Rodriguez, I., Urrutia, G., Buitrago-Garcia, D., Núñez-González, S., Martínez-Zapata, M. J., Madrid, E., Bonfill, X., & Hidalgo-Ottolenghi, R. (2019). Leukodepleted Packed Red Blood Cells Transfusion in Patients Undergoing Major Cardiovascular Surgical Procedure: Systematic Review and Meta-Analysis. *Cardiology Research and Practice*, *2019*(PG-7543917), 7543917. https://doi.org/10.1155/2019/7543917

Singh, A. N., & Kilambi, R. (2018). Single-stage laparoscopic common bile duct exploration and cholecystectomy versus two-stage endoscopic stone extraction followed by laparoscopic cholecystectomy for patients with gallbladder stones with common bile duct stones: systematic review and meta-analysis of randomized trials with trial sequential analysis. *Surgical Endoscopy*, *32*(9), 3763–3776. https://doi.org/10.1007/s00464-018-6170-8

Singh, N. P., Makkar, J. K., Bhatia, N., & Singh, P. M. (2021). The analgesic effectiveness of ilioinguinal-iliohypogastric block for caesarean delivery: A meta-analysis and trial sequential analysis. *European Journal of Anaesthesiology*, *38*(Suppl 2 PG-S87-S96), S87–S96. https://doi.org/10.1097/EJA.0000000000001379

Singh, N. P., Makkar, J. K., Borle, A., Monks, D., Goudra, B. G., Zorrilla-Vaca, A., & Singh, P. M. (2020). The analgesic efficacy of quadratus lumborum block in caesarean delivery: a meta-analysis and trial sequential analysis. *Journal of Anesthesia*, *34*(6), 814–824. https://doi.org/10.1007/s00540-020-02822-7

Singh, N. P., Makkar, J. K., Borle, A., & Singh, P. M. (2020). Analgesic efficacy of erector spinae plane block for oncologic breast surgery: A meta-analysis and trial sequential analysis. *Breast Journal*, *26*(11), 2295–2298. https://doi.org/10.1111/tbj.14001

Singh, N. P., Makkar, J. K., Yadav, N., Goudra, B. G., & Singh, P. M. (2022). The analgesic efficacy of intravenous dexamethasone for post-caesarean pain: A systematic review with meta-analysis and trial sequential analysis. *European Journal of Anaesthesiology*, *39*(6), 498–510. https://doi.org/10.1097/EJA.0000000000001626

Singh, P. M., Borle, A., Makkar, J. K., Trikha, A., Fish, D., & Sinha, A. (2018). Haloperidol Versus 5-HT3 Receptor Antagonists for Postoperative Vomiting and QTc Prolongation: A Noninferiority Meta-Analysis and Trial Sequential Analysis of Randomized Controlled Trials. *Journal of Clinical Pharmacology*, *58*(2), 131–143. https://doi.org/10.1002/jcph.999

Singh, P. M., Borle, A., Panwar, R., Makkar, J. K., McGrath, I., Trikha, A., & Sinha, A. (2018). Perioperative antiemetic efficacy of dexamethasone versus 5-HT3 receptor antagonists: a meta-analysis and trial sequential analysis of randomized controlled trials. *European Journal of Clinical Pharmacology*, *74*(10), 1201–1214. https://doi.org/10.1007/s00228-018-2495-4

Sivaramakrishnan, G., & Sridharan, K. (2019). Fluoride varnish versus glutaraldehyde for hypersensitive teeth: a randomized controlled trial, meta-analysis and trial sequential analysis. *Clinical Oral Investigations*, *23*(1), 209–220. https://doi.org/10.1007/s00784-018-2428-8
Snow, T. A. C., Littlewood, S., Corredor, C., Singer, M., & Arulkumaran, N. (2021). Effect of Extracorporeal Blood Purification on Mortality in Sepsis: A Meta-Analysis and Trial Sequential Analysis. *Blood Purification*, *50*(4–5), 462–472. https://doi.org/10.1159/000510982
Snow, T. A. C., Saleem, N., Ambler, G., Nastouli, E., McCoy, L. E., Singer, M., & Arulkumaran, N. (2021). Convalescent plasma for COVID-19: a meta-analysis, trial sequential analysis, and meta-regression. *British Journal of Anaesthesia*, *127*(6), 834–844. https://doi.org/10.1016/j.bja.2021.07.033
Snow, T. A. C., Saleem, N., Ambler, G., Nastouli, E., Singer, M., & Arulkumaran, N. (2021). Tocilizumab in COVID-19: a meta-analysis, trial sequential analysis, and meta-regression of randomized-controlled trials. *Intensive Care Medicine*, *47*(6), 641–652. https://doi.org/10.1007/s00134-021-06416-z
Song, Y., Chen, G., Huang, P., Hu, C., & Liu, X. (2020). Effects of tamsulosin combined with solifenacin on lower urinary tract symptoms: Evidence from a systematic review, meta-analysis, and trial sequential analysis of randomized controlled trials. *Frontiers in Pharmacology*, *11*(PG-763), 763. https://doi.org/10.3389/fphar.2020.00763
Song, Z. G., Pang, S. Y., Wang, G. Y., & Zhang, Z. (2021). Comparison of postoperative analgesic effects in response to either dexamethasone or dexmedetomidine as local anesthetic adjuvants: a systematic review and meta-analysis of randomized controlled trials. *Journal of Anesthesia*, *35*(2), 270–287. https://doi.org/10.1007/s00540-021-02895-y
Speyer, H., Jakobsen, A. S., Westergaard, C., Nørgaard, H. C. B., Pisinger, C., Krogh, J., Hjorthøj, C., Nordentoft, M., Gluud, C., Correll, C. U., & Jørgensen, K. B. (2019). Lifestyle Interventions for Weight Management in People with Serious Mental Illness: A Systematic Review with Meta-Analysis, Trial Sequential Analysis, and Meta-Regression Analysis Exploring the Mediators and Moderators of Treatment Effects. *Psychotherapy and Psychosomatics*, *88*(6), 350–362. https://doi.org/10.1159/000502293
Sridharan, K., & Sequeira, R. P. (2018). Drugs for treating severe hypertension in pregnancy: a network meta-analysis and trial sequential analysis of randomized clinical trials. *British Journal of Clinical Pharmacology*, *84*(9), 1906–1916. https://doi.org/10.1111/bcp.13649
Sridharan, K., & Sivaramakrishnan, G. (2019). Drugs for preventing post-operative nausea and vomiting in patients undergoing laparoscopic cholecystectomy: Network meta-analysis of randomized clinical trials and trial sequential analysis. *International Journal of Surgery*, *69*(PG-1-12), 1–12. https://doi.org/10.1016/j.ijsu.2019.07.002
Sridharan, K., & Sivaramakrishnan, G. (2018). Interventions for Improving Bone Mineral Density and Reducing Fracture Risk in Osteogenesis Imperfecta: A Mixed Treatment Comparison Network Meta-analysis of Randomized Controlled Clinical Trials. *Current Clinical Pharmacology*, *13*(3), 190–198. https://doi.org/10.2174/1574884713666180829143927

Sridharan, K., & Sivaramakrishnan, G. (2018). Efficacy and safety of iron chelators in thalassemia and sickle cell disease: a multiple treatment comparison network meta-analysis and trial sequential analysis. *Expert Review of Clinical Pharmacology*, *11*(6), 641–650. https://doi.org/10.1080/17512433.2018.1473760

Sridharan, K., & Sivaramakrishnan, G. (2021). A network meta-analysis of CYP2C9, CYP2C9 with VKORC1 and CYP2C9 with VKORC1 and CYP4F2 genotype-based warfarin dosing strategies compared to traditional. *Journal of Clinical Pharmacy and Therapeutics*, *46*(3), 640–648. https://doi.org/10.1111/jcpt.13334

Sridharan, K., & Sivaramakrishnan, G. (2018). Efficacy and safety of alpha blockers in medical expulsive therapy for ureteral stones: a mixed treatment network meta-analysis and trial sequential analysis of randomized controlled clinical trials. *Expert Review of Clinical Pharmacology*, *11*(3), 291–307. https://doi.org/10.1080/17512433.2018.1424537

Sridharan, K., & Sivaramakrishnan, G. (2018). Pharmacological interventions for preventing acute mountain sickness: a network meta-analysis and trial sequential analysis of randomized clinical trials. *Annals of Medicine*, *50*(2), 147–155. https://doi.org/10.1080/07853890.2017.1407034

Sridharan, K., & Sivaramakrishnan, G. (2019). Vasoactive agents for the management of variceal bleeding: A mixed treatment comparison network meta-analysis and trial sequential analysis of randomized clinical trials. *Drug Research*, *69*(9), 487–495. https://doi.org/10.1055/a-0846-3071

Stacchi, C., Bassi, F., Troiano, G., Rapani, A., Lombardi, T., Jokstad, A., Sennerby, L., & Schierano, G. (2020). Piezoelectric bone surgery for implant site preparation compared with conventional drilling techniques: A systematic review, meta-analysis and trial sequential analysis. *International Journal of Oral Implantology (Berlin, Germany)*, *13*(2), 141–158. http://www.ncbi.nlm.nih.gov/pubmed/32424381

Stacchi, C., Bassi, F., Troiano, G., Rapani, A., Lombardi, T., Jokstad, A., Sennerby, L., & Schierano, G. (2020). Piezoelectric bone surgery for implant site preparation compared with conventional drilling techniques: A systematic review, meta-analysis and trial sequential analysis. *International Journal of Oral Implantology (Berlin, Germany)*, *13*(2), 141–158. http://www.ncbi.nlm.nih.gov/pubmed/32424381

Storebø, O. J., Andersen, M. E., Skoog, M., Hansen, S. J., Simonsen, E., Pedersen, N., Tendal, B., Callesen, H. E., Faltinsen, E., & Gluud, C. (2019). Social skills training for attention deficit hyperactivity disorder (ADHD) in children aged 5 to 18 years. *Cochrane Database of Systematic Reviews*, *2019*(6). https://doi.org/10.1002/14651858.CD008223.pub3

Storebø, O. J., Stoffers-Winterling, J. M., Völlm, B. A., Kongerslev, M. T., Mattivi, J. T., Jørgensen, M. S., Faltinsen, E., Todorovac, A., Sales, C. P., Callesen, H. E., Lieb, K., & Simonsen, E. (2020). Psychological therapies for people with borderline personality disorder. *Cochrane Database of Systematic Reviews*, *2020*(5), CD012955. https://doi.org/10.1002/14651858.CD012955.pub2

Sun, H., Xie, Q., & Peng, Z. (2019). Does Fenoldopam Protect Kidney in Cardiac Surgery? A Systemic Review and Meta-Analysis with Trial Sequential Analysis. *Shock*, *52*(3), 326–333. https://doi.org/10.1097/SHK.0000000000001313

Szapary, L. B., Szakacs, Z., Farkas, N., Schonfeld, K., Babocsay, D., Gajer, M., Kittka, B., Magyari, B., Hegyi, P., Szokodi, I., & Horvath, I. G. (2021). The effect of magnesium on reperfusion arrhythmias in stemi patients,

treated with ppci. A systematic review with a meta-analysis and trial sequential analysis. *Frontiers in Cardiovascular Medicine*, *7*(PG-608193), 608193. https://doi.org/10.3389/fcvm.2020.608193

Szemes, K., Soós, A., Hegyi, P., Farkas, N., Erős, A., Erőss, B., Mezősi, E., Szakács, Z., Márta, K., & Sarlós, P. (2020). Comparable Long-Term Outcomes of Cyclosporine and Infliximab in Patients With Steroid-Refractory Acute Severe Ulcerative Colitis: A Meta-Analysis. *Frontiers in Medicine*, *6*(PG-338), 338. https://doi.org/10.3389/fmed.2019.00338

Tan, H. Sen, Taylor, C., Weikel, D., Barton, K., & Habib, A. S. (2020). Quadratus lumborum block for postoperative analgesia after cesarean delivery: A systematic review with meta-analysis and trial-sequential analysis. *Journal of Clinical Anesthesia*, *67*(PG-110003), 110003. https://doi.org/10.1016/j.jclinane.2020.110003

Tan, Y. Y., Man, X. X., Liu, L. Y., & Xu, H. (2019). Comparison of clinical outcomes between intravascular ultrasound-guided and angiography-guided drug-eluting stent implantation: A meta-analysis of randomised control trials and systematic review. *International Wound Journal*, *16*(3), 649–658. https://doi.org/10.1111/iwj.13073

Tang, Y., Meng, J., Zhang, X., Li, J., & Zhou, Q. (2019). Comparison of dexmedetomidine with propofol as sedatives for pediatric patients undergoing magnetic resonance imaging: A meta-analysis of randomized controlled trials with trial sequential analysis. *Experimental and Therapeutic Medicine*, *18*(3 PG-1775–1785), 1775–1785. https://doi.org/10.3892/etm.2019.7751

Tang, Z., Yang, Y., Yang, Z., Meng, W., & Li, X. (2018). Early precut sphincterotomy does not increase the risk of adverse events for patients with difficult biliary access: A systematic review of randomized clinical trials with meta-analysis and trial sequential analysis. *Medicine (United States)*, *97*(36), e12213. https://doi.org/10.1097/MD.0000000000012213

Taylor, R. S., Long, L., Mordi, I. R., Madsen, M. T., Davies, E. J., Dalal, H., Rees, K., Singh, S. J., Gluud, C., & Zwisler, A. D. (2019). Exercise-Based Rehabilitation for Heart Failure: Cochrane Systematic Review, Meta-Analysis, and Trial Sequential Analysis. *JACC: Heart Failure*, *7*(8), 691–705. https://doi.org/10.1016/j.jchf.2019.04.023

Teoh, R. J. J., Huang, C. J., Chan, C. P., Chien, L. Y., Chung, C. P., Sung, S. H., Chen, C. H., Chiang, C. E., & Cheng, H. M. (2019). Does statin increase the risk of intracerebral hemorrhage in stroke survivors? A meta-analysis and trial sequential analysis. *Therapeutic Advances in Neurological Disorders*, *12*(PG-1756286419864830), 1756286419864830. https://doi.org/10.1177/1756286419864830

Thy, M., Montmayeur, J., Julien-Marsollier, F., Michelet, D., Brasher, C., Dahmani, S., & Orliaguet, G. (2018). Safety and efficacy of peri-operative administration of hydroxyethyl starch in children undergoing surgery. *European Journal of Anaesthesiology*, *35*(7), 484–495. https://doi.org/10.1097/EJA.0000000000000780

Tian, X., Xu, L. L., Liu, X. L., & Chen, W. Q. (2020). Enhanced patient education for colonic polyp and adenoma detection: Meta-analysis of randomized controlled trials. *JMIR MHealth and UHealth*, *8*(6), e17372. https://doi.org/10.2196/17372

Tong, Z., Guo, L., Qi, L., Cui, S., Gao, X., Li, Y., Guo, J., & Gu, Y. (2020). Drug-Coated Balloon Angioplasty and Debulking for the Treatment of Femoropopliteal In-Stent Restenosis: A Systematic Review and Meta-

Analysis. *BioMed Research International*, *2020*(PG-3076346), 3076346. https://doi.org/10.1155/2020/3076346

Tramacere, I., Boncoraglio, G. B., Banzi, R., Del Giovane, C., Kwag, K. H., Squizzato, A., & Moja, L. (2019). Comparison of statins for secondary prevention in patients with ischemic stroke or transient ischemic attack: A systematic review and network meta-analysis. *BMC Medicine*, *17*(1), 67. https://doi.org/10.1186/s12916-019-1298-5

Troiano, G., Lo Russo, L., Canullo, L., Ciavarella, D., Lo Muzio, L., & Laino, L. (2018). Early and late implant failure of submerged versus non-submerged implant healing: A systematic review, meta-analysis and trial sequential analysis. *Journal of Clinical Periodontology*, *45*(5), 613–623. https://doi.org/10.1111/jcpe.12890

Troiano, G., Zhurakivska, K., Lo Muzio, L., Laino, L., Cicciù, M., & Lo Russo, L. (2017). Combination of Bone Graft and Resorbable Membrane for Alveolar Ridge Preservation: a Systematic Review, Meta-analysis and Trial Sequential Analysis. *Journal of Periodontology*, *89*(1 PG-46–57), 1–17. https://doi.org/10.1902/jop.2017.170241

Tsai, H. R., Chen, T. L., Chang, C. Y., Huang, H. K., & Lee, Y. C. (2021). Face-down posture versus non-face-down posture following large idiopathic macular hole surgery: A systemic review and meta-analysis. *Journal of Clinical Medicine*, *10*(21). https://doi.org/10.3390/jcm10214895

Tyraskis, A., Parsons, C., & Davenport, M. (2018). Glucocorticosteroids for infants with biliary atresia following Kasai portoenterostomy. *Cochrane Database of Systematic Reviews*, *2018*(5), CD008735. https://doi.org/10.1002/14651858.CD008735.pub3

Uthman, O. A., Nduka, C. U., Abba, M., Enriquez, R., Nordenstedt, H., Nalugoda, F., Kengne, A. P., & Ekström, A. M. (2019). Comparison of mhealth and face-to-face interventions for smoking cessation among people living with HIV: Meta-analysis. *JMIR MHealth and UHealth*, *7*(1), e203. https://doi.org/10.2196/mhealth.9329

Vadera, S., Yong, C. W. K., Gluud, L. L., & Morgan, M. Y. (2019). Band ligation versus no intervention for primary prevention of upper gastrointestinal bleeding in adults with cirrhosis and oesophageal varices. *Cochrane Database of Systematic Reviews*, *2019*(6), CD012673. https://doi.org/10.1002/14651858.CD012673.pub2

Valkenburg, C., Van der Weijden, F., & Slot, D. E. (2019). Is plaque regrowth inhibited by dentifrice?: A systematic review and meta-analysis with trial sequential analysis. *International Journal of Dental Hygiene*, *17*(1), 27–38. https://doi.org/10.1111/idh.12364

Vargas, M., Marra, A., Buonanno, P., Coviello, A., Iacovazzo, C., & Servillo, G. (2021). Fragility index and fragility quotient in randomized controlled trials on corticosteroids in ards due to covid-19 and non-covid-19 etiology. *Journal of Clinical Medicine*, *10*(22). https://doi.org/10.3390/jcm10225287

Vettoretto, N., Arezzo, A., Famiglietti, F., Cirocchi, R., Moja, L., & Morino, M. (2018). Laparoscopic-endoscopic rendezvous versus preoperative endoscopic sphincterotomy in people undergoing laparoscopic cholecystectomy for stones in the gallbladder and bile duct. *Cochrane Database of Systematic Reviews*, *2018*(4), CD010507. https://doi.org/10.1002/14651858.CD010507.pub2

Wahlstrøm, K. L., Bjerrum, E., Gögenur, I., Burcharth, J., & Ekeloef, S. (2021). Effect of remote ischaemic preconditioning on mortality and morbidity after non-cardiac surgery: Meta-analysis. *BJS Open*, *5*(2). https://doi.org/10.1093/bjsopen/zraa026
Wang, B., He, X., Gong, Y., & Cheng, B. (2018). Levosimendan in Patients with Left Ventricular Dysfunction Undergoing Cardiac Surgery: An Update Meta-Analysis and Trial Sequential Analysis. *BioMed Research International*, *2018*(PG-7563083), 7563083. https://doi.org/10.1155/2018/7563083
Wang, D., Qu, J., Jiang, H., & Jiang, Y. (2019). The safety and efficacy of botulinum toxin for management of scars: A systematic review with meta-analysis and trial sequential analysis. *Toxicon*, *166*(PG-24-33), 24–33. https://doi.org/10.1016/j.toxicon.2019.04.018
Wang, D. D., Ma, T. T., Zhu, H. D., & Peng, C. Bin. (2018). Transdermal fentanyl for cancer pain: Trial sequential analysis of 3406 patients from 35 randomized controlled trials. *Journal of Cancer Research and Therapeutics*, *14*(8), S14–S21. https://doi.org/10.4103/0973-1482.171368
Wang, G., Liu, H., Wang, C., Ji, X., Gu, W., & Mu, Y. (2018). Cinacalcet versus Placebo for secondary hyperparathyroidism in chronic kidney disease patients: A meta-analysis of randomized controlled trials and trial sequential analysis. *Scientific Reports*, *8*(1), 3111. https://doi.org/10.1038/s41598-018-21397-8
Wang, J. H., Van Haselen, R., Wang, M., Yang, G. L., Zhang, Z., Friedrich, M. E., Wang, L. Q., Zhou, Y. Q., Yin, M., Xiao, C. Y., Duan, A. L., Liu, S. C., Chen, B., & Liu, J. P. (2019). Acupuncture for smoking cessation: A systematic review and meta-analysis of 24 randomized controlled trials. *Tobacco Induced Diseases*, *17*(June), 48. https://doi.org/10.18332/tid/109195
Wang, R., Cheng, N., Peng, R., Yu, Z., Nan, M., & Cao, H. (2020). Oral herbal medicine for women with intrahepatic cholestasis in pregnancy: A systematic review of randomized controlled trials. *BMC Complementary Medicine and Therapies*, *20*(1), 303. https://doi.org/10.1186/s12906-020-03097-x
Wang, R., Pan, C., Wang, X., Xu, F., Jiang, S., & Li, M. (2019). The impact of tracheotomy timing in critically ill patients undergoing mechanical ventilation: A meta-analysis of randomized controlled clinical trials with trial sequential analysis. *Heart and Lung*, *48*(1), 46–54. https://doi.org/10.1016/j.hrtlng.2018.09.005
Wang, X., Zheng, B., Lu, X., Bai, R., Feng, L., Wang, Q., Zhao, Y., & He, S. (2018). Preoperative short-course radiotherapy and long-course radiochemotherapy for locally advanced rectal cancer: Meta-analysis with trial sequential analysis of long-term survival data. *PLoS ONE*, *13*(7), e0200142. https://doi.org/10.1371/journal.pone.0200142
Wang, X., Liu, Z., Sui, X., Wu, Q., Wang, J., & Xu, C. (2019). Elemene injection as adjunctive treatment to platinum-based chemotherapy in patients with stage III/IV non-small cell lung cancer: A meta-analysis following the PRISMA guidelines. *Phytomedicine*, *59*(PG-152787), 152787. https://doi.org/10.1016/j.phymed.2018.12.010
Wang, Z., Li, Y., Lin, D., & Ma, J. (2021). Effect of Melatonin on Postoperative Pain and Perioperative Opioid Use: A Meta-analysis and Trial Sequential Analysis. *Pain Practice*, *21*(2), 190–203. https://doi.org/10.1111/papr.12948

Wang, Z., He, Y., & Zheng, Y. (2019). Probiotics for the treatment of bacterial vaginosis: A meta-analysis. *International Journal of Environmental Research and Public Health*, *16*(20). https://doi.org/10.3390/ijerph16203859
Winther-Olesen, M., Møller, M. H., Johansen, K. K., & Aasvang, E. K. (2020). Effects of post-operative furosemide in adult surgical patients: A systematic review and meta-analysis of randomised clinical trials. *Acta Anaesthesiologica Scandinavica*, *64*(3), 282–291. https://doi.org/10.1111/aas.13513
Wolf, H. T., Huusom, L. D., Henriksen, T. B., Hegaard, H. K., Brok, J., & Pinborg, A. (2020). Magnesium sulphate for fetal neuroprotection at imminent risk for preterm delivery: a systematic review with meta-analysis and trial sequential analysis. *BJOG: An International Journal of Obstetrics and Gynaecology*, *127*(10), 1180–1188. https://doi.org/10.1111/1471-0528.16238
Wong, T. Y., Loo, Y. S., Veettil, S. K., Wong, P. S., Divya, G., Ching, S. M., & Menon, R. K. (2020). Efficacy and safety of posaconazole for the prevention of invasive fungal infections in immunocompromised patients: a systematic review with meta-analysis and trial sequential analysis. *Scientific Reports*, *10*(1), 14575. https://doi.org/10.1038/s41598-020-71571-0
Wu, R. han, Feng, S., Han, M., Caldwell, P., Liu, S. gang, Zhang, J., & Liu, J. ping. (2018). Yinzhihuang oral liquid combined with phototherapy for neonatal jaundice: A systematic review and meta-analysis of randomized clinical trials. *BMC Complementary and Alternative Medicine*, *18*(1), 228. https://doi.org/10.1186/s12906-018-2290-x
Wu, S., Bai, X., Guo, C., Huang, Z., Ouyang, H., Huang, J., & Zeng, W. (2021). Ganglioside-monosialic acid (GM1) for prevention of chemotherapy-induced peripheral neuropathy: a meta-analysis with trial sequential analysis. *BMC Cancer*, *21*(1), 1173. https://doi.org/10.1186/s12885-021-08884-4
Wu, T., Hu, C., Huang, W., Xu, Q., Hu, B., & Li, J. (2021). Effect of Combined Hydrocortisone, Ascorbic Acid and Thiamine for Patients with Sepsis and Septic Shock: A Systematic Review and Meta-Analysis. *Shock (Augusta, Ga.)*, *56*(6), 880–889. https://doi.org/10.1097/SHK.0000000000001781
Wu, X. D., Liu, M. M., Liang, X., Hu, N., & Huang, W. (2018). Effects of perioperative supplementation with pro-/synbiotics on clinical outcomes in surgical patients: A meta-analysis with trial sequential analysis of randomized controlled trials. *Clinical Nutrition*, *37*(2), 505–515. https://doi.org/10.1016/j.clnu.2016.10.015
Xia, J. Y., Yang, C., Xu, D. F., Xia, H., Yang, L. G., & Sun, G. J. (2021). Consumption of cranberry as adjuvant therapy for urinary tract infections in susceptible populations: A systematic review and meta-analysis with trial sequential analysis. *PLoS ONE*, *16*(9 September), e0256992. https://doi.org/10.1371/journal.pone.0256992
Xia, L., Zeng, L. H., Pan, J. P., & Ding, Y. M. (2020). Effects of stem cells on non-ischemic cardiomyopathy: a systematic review and meta-analysis of randomized controlled trials. *Cytotherapy*, *22*(12), 699–711. https://doi.org/10.1016/j.jcyt.2020.06.006
Xiang, G. L., Wu, Q. H., Xie, L., Song, J. Q., Wu, X., Hao, S. Y., Zhong, M., & Li, S. Q. (2021). High flow nasal cannula versus conventional oxygen therapy in postoperative patients at high risk for pulmonary complications: A systematic review and meta-analysis. *International Journal of Clinical Practice*, *75*(3), e13828. https://doi.org/10.1111/ijcp.13828

Xie, C., He, C., Gao, J., & Jia, S. (2020). Efficacy and safety of tripterygium glycosides in the treatment of hyperthyroidism: A systemic review and meta-analysis. *Medicine (United States)*, *99*(38), E22282. https://doi.org/10.1097/MD.0000000000022282
Xie, W., Dai, P., Qin, Y., Wu, M., Yang, B., & Yu, X. (2020). Effectiveness of telemedicine for pregnant women with gestational diabetes mellitus: An updated meta-analysis of 32 randomized controlled trials with trial sequential analysis. *BMC Pregnancy and Childbirth*, *20*(1), 198. https://doi.org/10.1186/s12884-020-02892-1
Xie, X., Liu, X., Chen, B., & Wang, Q. (2018). Prophylactic Atrial Fibrillation Ablation in Atrial Flutter Patients without Atrial Fibrillation: A Meta-Analysis with Trial Sequential Analysis. *Medical Science Monitor Basic Research*, *24*(PG-96-102), 96–102. https://doi.org/10.12659/MSMBR.910338
Xing, M., Liang, X., Li, L., Liao, L., Liang, S., Jiang, S., Li, J., Zhang, C., & Zou, W. (2020). Efficacy of caudal vs intravenous administration of α2 adrenoceptor agonists to prolong analgesia in pediatric caudal block: A systematic review and meta-analysis. *Paediatric Anaesthesia*, *30*(12), 1322–1330. https://doi.org/10.1111/pan.14025
Xing, X., Xu, M., Yang, L., Zhang, W., Niu, X., & Gao, D. (2021). The efficacy of intravenous vitamin C in critically ill patients: A meta-analysis of randomized controlled trials. *Clinical Nutrition*, *40*(5), 2630–2639. https://doi.org/10.1016/j.clnu.2021.03.007
Xing, Z., Tang, L., Chen, P., Huang, J., Peng, X., & Hu, X. (2018). Levosimendan in patients with left ventricular dysfunction undergoing cardiac surgery: A meta-analysis and trial sequential analysis of randomized trials. *Scientific Reports*, *8*(1), 7775. https://doi.org/10.1038/s41598-018-26206-w
Xing, Z., Tang, L., Zhu, Z., & Hu, X. (2018). Effects of thrombolysis on outcomes of patients with deep venous thrombosis: An updated meta-analysis. *PLoS ONE*, *13*(9), e0204594. https://doi.org/10.1371/journal.pone.0204594
Xiong, X., Chen, D., & Shi, J. (2021). Is Perioperative Dexmedetomidine Associated With a Reduced Risk of Perioperative Neurocognitive Disorders Following Cardiac Surgery? A Systematic Review and Meta-Analysis With Trial Sequential Analysis of Randomized Controlled Trials. *Frontiers in Medicine*, *8*(PG-645975), 645975. https://doi.org/10.3389/fmed.2021.645975
Xu, C., Liu, S., Huang, Y. Z., Guo, X. W., Xiao, H. B., & Qi, D. Y. (2018). Phenylephrine vs ephedrine in cesarean delivery under spinal anesthesia: A systematic literature review and meta-analysis. *International Journal of Surgery*, *60*(PG-48-59), 48–59. https://doi.org/10.1016/j.ijsu.2018.10.039
Xu, R., Wang, Q., Huang, Y., Wu, L., Liu, Q., Hu, W., Zhou, C., & Du, Q. (2018). Do low-dose corticosteroids improve survival or shock reversal from septic shock in adults? Meta-analysis with trial sequential analysis. *Journal of International Medical Research*, *46*(7), 2513–2524. https://doi.org/10.1177/0300060518774985
Xu, X. L., Liu, X. Di, Liang, M., & Luo, B. M. (2018). Radiofrequency ablation versus hepatic resection for small hepatocellular carcinoma: Systematic review of randomized controlled trials with meta-analysis and trial sequential analysis. *Radiology*, *287*(2), 461–472. https://doi.org/10.1148/radiol.2017162756

Xu, Y., Zheng, X., Liang, B., Gao, J., & Gu, Z. (2018). Vitamins for Prevention of Contrast-induced Acute Kidney Injury: A Systematic Review and Trial Sequential Analysis. *American Journal of Cardiovascular Drugs*, *18*(5), 373–386. https://doi.org/10.1007/s40256-018-0274-3
Xue, M., Zhang, X., Liu, F., Chang, W., Xie, J., Xu, J., Yang, Y., & Qiu, H. (2019). Effects of chloride content of intravenous crystalloid solutions in critically ill adult patients: a meta-analysis with trial sequential analysis of randomized trials. *Annals of Intensive Care*, *9*(1), 30. https://doi.org/10.1186/s13613-019-0506-y
Yamada, H., Doi, K., Tsukamoto, T., Kiyomoto, H., Yamashita, K., Yanagita, M., Terada, Y., & Mori, K. (2019). Low-dose atrial natriuretic peptide for prevention or treatment of acute kidney injury: A systematic review and meta-analysis. *Critical Care*, *23*(1), 41. https://doi.org/10.1186/s13054-019-2330-z
Yamakawa, K., Murao, S., & Aihara, M. (2019). Recombinant Human Soluble Thrombomodulin in Sepsis-Induced Coagulopathy: An Updated Systematic Review and Meta-Analysis. *Thrombosis and Haemostasis*, *119*(1), 56–65. https://doi.org/10.1055/s-0038-1676345
Yan, J., Ma, H., Liu, A., Huang, J., Wu, J., & Yang, J. (2021). Efficacy and Safety of Rotigotine Transdermal Patch on Neuropsychiatric Symptoms of Parkinson's Disease: An Updated Meta-Analysis and Systematic Review. *Frontiers in Neurology*, *12*(PG-722892), 722892. https://doi.org/10.3389/fneur.2021.722892
Yan, W. S., Cao, W. L., Sun, M., Ma, D. Y., & Zhang, P. (2020). Distal locked or unlocked nailing for stable intertrochanteric fractures? A meta-analysis. *ANZ Journal of Surgery*, *90*(1–2), 27–33. https://doi.org/10.1111/ans.15232
Yang, S. S., & Ramdoyal, N. (2020). Do erythropoietin and iron really decrease blood transfusion in surgical patients? A trial sequential analysis. *Canadian Journal of Anesthesia*, *67*(6), 777–778. https://doi.org/10.1007/s12630-019-01564-9
Yao, R., Zhu, Y., Yu, Y., Li, Z., Wang, L., Zheng, L., Li, J., Huang, H., Wu, G., Zhu, F., Xia, Z., Ren, C., & Yao, Y. (2021). Combination therapy of thiamine, vitamin C and hydrocortisone in treating patients with sepsis and septic shock: a meta-analysis and trial sequential analysis. *Burns and Trauma*, *9*(PG-tkab040), tkab040. https://doi.org/10.1093/burnst/tkab040
Ye, G., Wang, S., & Peng, D. (2021). Effects of SGLT2 Inhibitor on Ischemic Events Stemming from Atherosclerotic Coronary Diseases: A Systematic Review and Meta-analysis with Trial Sequential Analysis of Randomized Controlled Trials. *Journal of Cardiovascular Pharmacology*, *77*(6), 787–795. https://doi.org/10.1097/FJC.0000000000001018
Yeh, T. C., Tzeng, N. S., Li, J. C., Huang, Y. C., Hsieh, H. Te, Chu, C. S., & Liang, C. S. (2019). Mortality Risk of Atypical Antipsychotics for Behavioral and Psychological Symptoms of Dementia: A Meta-Analysis, Meta-Regression, and Trial Sequential Analysis of Randomized Controlled Trials. *Journal of Clinical Psychopharmacology*, *39*(5), 472–478. https://doi.org/10.1097/JCP.0000000000001083
Yi, L. J., Tian, X., Shi, B., Chen, H., Liu, X. L., Pi, Y. P., & Chen, W. Q. (2019). Low-Volume Polyethylene Glycol Improved Patient Attendance in Bowel Preparation Before Colonoscopy: A Meta-Analysis With Trial Sequential Analysis. *Frontiers in Medicine*, *6*(PG-92), 92. https://doi.org/10.3389/fmed.2019.00092

Yokoyama, C., Mihara, T., Kashiwagi, S., Koga, M., & Goto, T. (2020). Effects of intravenous dextrose on preventing postoperative nausea and vomiting: A systematic review and meta-analysis with trial sequential analysis. *PLoS ONE*, *15*(4), e0231958. https://doi.org/10.1371/journal.pone.0231958

Young, B., Onwochei, D., & Desai, N. (2021). Conventional landmark palpation vs. preprocedural ultrasound for neuraxial analgesia and anaesthesia in obstetrics – a systematic review and meta-analysis with trial sequential analyses. *Anaesthesia*, *76*(6), 818–831. https://doi.org/10.1111/anae.15255

Yu, Y. F., Wang, Y., Fu, T. P., Chen, K., Liu, J. Q., & Yao, H. R. (2018). Trastuzumab combined with doublet or single-agent chemotherapy as first-line therapy for HER2-positive metastatic breast cancer. *Breast Cancer Research and Treatment*, *168*(2), 337–348. https://doi.org/10.1007/s10549-017-4592-y

Yu, Y., Liu, N., Zeng, Q., Duan, J., Bao, Q., Lei, M., Zhao, J., & Xie, J. (2019). The efficacy of pregabalin for the management of acute and chronic postoperative pain in thoracotomy: A meta-analysis with trial sequential analysis of randomized-controlled trials. *Journal of Pain Research*, *12*(PG-159-170), 159–170. https://doi.org/10.2147/JPR.S183411

Zaman, S., Mohamedahmed, A. Y. Y., Peterknecht, E., Zakaria, R. M., Mohamedahmed, S. Y. Y., Hajibandeh, S., & Hajibandeh, S. (2022). Sutures versus clips for skin closure following caesarean section: a systematic review, meta-analysis and trial sequential analysis of randomised controlled trials. *Langenbeck's Archives of Surgery*, *407*(1), 37–50. https://doi.org/10.1007/s00423-021-02239-0

Zaman, S., Mohamedahmed, A. Y. Y., Srinivasan, A., Stonelake, S., Sillah, A. K., Hajibandeh, S., & Hajibandeh, S. (2021). Single-port laparoscopic appendicectomy versus conventional three-port approach for acute appendicitis: A systematic review, meta-analysis and trial sequential analysis of randomised controlled trials. *Surgeon*, *19*(6), 365–379. https://doi.org/10.1016/j.surge.2021.01.018

Zang, H., Zhang, Q., & Li, X. (2019). Adenosine antagonists for prevention of contrast-induced nephropathy: A meta-analysis of randomized controlled trials with trial sequential analysis. *Experimental and Therapeutic Medicine*, *18*(1 PG-85–98), 85–98. https://doi.org/10.3892/etm.2019.7566

Zayed, Y., Alzghoul, B. N., Banifadel, M., Venigandla, H., Hyde, R., Sutchu, S., Khasawneh, M., Borok, Z., Urbine, D., Jantz, M., & Reddy, R. (2022). Vitamin C, Thiamine, and Hydrocortisone in the Treatment of Sepsis: A Meta-Analysis and Trial Sequential Analysis of Randomized Controlled Trials. *Journal of Intensive Care Medicine*, *37*(3), 327–336. https://doi.org/10.1177/0885066620987809

Zayed, Y., Barbarawi, M., Ismail, E., Samji, V., Kerbage, J., Rizk, F., Salih, M., Bala, A., Obeid, M., Deliwala, S., Demian, S., Al-Sanouri, I., & Reddy, R. (2020). Use of glucocorticoids in patients with acute respiratory distress syndrome: a meta-analysis and trial sequential analysis. *Journal of Intensive Care*, *8*(1), 43. https://doi.org/10.1186/s40560-020-00464-1

Zhang, B., Li, M. M., Chen, W. H., Zhao, J. F., Chen, W. Q., Dong, Y. H., Gong, X., Chen, Q. Y., Zhang, L., Mo, X. K., Luo, X. N., Tian, J., & Zhang, S. X. (2019). Association of Chemoradiotherapy Regimens and Survival among Patients with Nasopharyngeal Carcinoma: A Systematic Review and Meta-analysis. *JAMA Network Open*, *2*(10 PG-e1913619), e1913619. https://doi.org/10.1001/jamanetworkopen.2019.13619

Zhang, C. D., Yamashita, H., Zhang, S., & Seto, Y. (2018). Reevaluation of laparoscopic versus open distal gastrectomy for early gastric cancer in Asia: A meta-analysis of randomized controlled trials. *International Journal of Surgery*, *56*(PG-31-43), 31–43. https://doi.org/10.1016/j.ijsu.2018.05.733

Zhang, H., Lu, Y., Wang, L., Lv, J., Ma, Y., Wang, W., Li, G., & Li, Y. (2019). Bispectral index monitoring of sedation depth during endoscopy: A meta-analysis with trial sequential analysis of randomized controlled trials. *Minerva Anestesiologica*, *85*(4), 412–432. https://doi.org/10.23736/S0375-9393.18.13227-5

Zhang, H., & Chang, R. (2019). Effects of exercise after percutaneous coronary intervention on cardiac function and cardiovascular adverse events in patients with coronary heart disease: Systematic review and meta-analysis. *Journal of Sports Science and Medicine*, *18*(2), 213–222. NS -

Zhang, J., Yu, Y., Miao, S., Liu, L., Gan, S., Kang, X., & Zhu, S. (2019). Effects of peri-operative intravenous administration of dexmedetomidine on emergence agitation after general anesthesia in adults: A meta-analysis of randomized controlled trials. *Drug Design, Development and Therapy*, *13*(PG-2853-2864), 2853–2864. https://doi.org/10.2147/DDDT.S207016

Zhang, J., Li, X., Yang, B., Wu, C., Fan, Y., & Li, H. (2019). Alpha-blockers with or without phosphodiesterase type 5 inhibitor for treatment of lower urinary tract symptoms secondary to benign prostatic hyperplasia: a systematic review and meta-analysis. *World Journal of Urology*, *37*(1), 143–153. https://doi.org/10.1007/s00345-018-2370-z

Zhang, J., Yang, B., Xiao, W., Li, X., & Li, H. (2018). Effects of testosterone supplement treatment in hypogonadal adult males with T2DM: a meta-analysis and systematic review. *World Journal of Urology*, *36*(8), 1315–1326. https://doi.org/10.1007/s00345-018-2256-0

Zhang, L. F., Zhang, C. F., Tang, W. X., He, L., Liu, Y., Tian, D. D., & Ai, Y. Q. (2020). Efficacy of amisulpride on postoperative nausea and vomiting: a systematic review and meta-analysis. *European Journal of Clinical Pharmacology*, *76*(7), 903–912. https://doi.org/10.1007/s00228-020-02869-1

Zhang, P., Wu, L., Shi, X., Zhou, H., Liu, M., Chen, Y., & Lv, X. (2020). Positive end-expiratory pressure during Anesthesia for prevention of postoperative pulmonary complications: A meta-analysis with trial sequential analysis of randomized controlled trials. *Anesthesia and Analgesia*, *130*(4), 879–889. https://doi.org/10.1213/ANE.0000000000004421

Zhang, X., Shao, F., Zhu, L., Ze, Y., Zhu, D., & Bi, Y. (2018). Cardiovascular and microvascular outcomes of glucagon-like peptide-1 receptor agonists in type 2 diabetes: A meta-analysis of randomized controlled cardiovascular outcome trials with trial sequential analysis. *BMC Pharmacology and Toxicology*, *19*(1), 58. https://doi.org/10.1186/s40360-018-0246-x

Zhang, X. L., Zhu, Q. Q., Chen, Y. H., Li, X. L., Chen, F., Huang, J. A., & Xu, B. (2018). Cardiovascular safety, long-term noncardiovascular safety, and efficacy of sodium-glucose cotransporter 2 inhibitors in patients with type 2 diabetes mellitus: A systemic review and meta-analysis with trial sequential analysis. *Journal of the American Heart Association*, *7*(2). https://doi.org/10.1161/JAHA.117.007165

Zhang, X., Wu, Q., Wei, M., Ding, Y., Gu, C., Liu, S., & Wang, Z. (2020). Low-residual diet versus clear-liquid diet for bowel preparation before colonoscopy: meta-analysis and trial sequential analysis of randomized controlled trials. *Gastrointestinal Endoscopy*, *92*(3), 508-518.e3. https://doi.org/10.1016/j.gie.2020.04.069

Zhang, Y., Li, Y., Liu, J., Wei, X., Tan, N., Zhang, J., Wang, W., & Wang, Y. (2021). Association of Vitamin D or Calcium Supplementation with Cardiovascular Outcomes and Mortality: A Meta-Analysis with Trial Sequential Analysis. *Journal of Nutrition, Health and Aging*, *25*(2), 263–270. https://doi.org/10.1007/s12603-020-1551-9

Zhang, Y., Chai, Y., Pan, X., Shen, H., Wei, X., & Xie, Y. (2019). Tai chi for treating osteopenia and primary osteoporosis: A meta-analysis and trial sequential analysis. *Clinical Interventions in Aging*, *14*(PG-91-104), 91–104. https://doi.org/10.2147/CIA.S187588

Zhang, Y., Song, A., Liu, J., Dai, J., & Lin, J. (2021). Therapeutic effect of nebulized hypertonic saline for muco-obstructive lung diseases: A systematic review and meta-analysis with trial sequential analysis. *Journal of Investigative Medicine*, *69*(3), 742–748. https://doi.org/10.1136/jim-2020-001479

Zhang, Z., Yang, Y., Jiang, S. M., & Li, W. G. (2019). Efficacy and safety of immunosuppressive treatment in IgA nephropathy: A meta-analysis of randomized controlled trials. *BMC Nephrology*, *20*(1), 333. https://doi.org/10.1186/s12882-019-1519-3

Zhao, B., Zhao, H., & Zhao, J. (2019). Risk of fatal adverse events in cancer patients treated with sunitinib. *Critical Reviews in Oncology/Hematology*, *137*(PG-115-122), 115–122. https://doi.org/10.1016/j.critrevonc.2019.03.007

Zhao, B., Zhao, H., & Zhao, J. (2019). Incidence and Risk of Fatal Adverse Events in Cancer Patients Treated With Vascular Endothelial Growth Factor Receptor 2-Targeted Agents: A Meta-Analysis With Trial Sequential Analysis of Randomized Controlled Trials. *Frontiers in Medicine*, *6*(PG-176), 176. https://doi.org/10.3389/fmed.2019.00176

Zhao, B., Zhao, H., & Zhao, J. (2020). Fatal adverse events associated with programmed cell death protein 1 or programmed cell death-ligand 1 monotherapy in cancer. *Therapeutic Advances in Medical Oncology*, *12*(PG-1758835919895753), 1758835919895753. https://doi.org/10.1177/1758835919895753

Zhao, B., Deng, H., Li, B., Chen, L., Zou, F., Hu, L., Wei, Y., & Zhang, W. (2020). Association of magnesium consumption with type 2 diabetes and glucose metabolism: A systematic review and pooled study with trial sequential analysis. *Diabetes/Metabolism Research and Reviews*, *36*(3), e3243. https://doi.org/10.1002/dmrr.3243

Zhao, B., Wu, Q., Wang, L., Liao, C., Dong, Y., Xu, J., Wei, Y., & Zhang, W. (2021). Pros and Cons of Aspirin for the Primary Prevention of Cardiovascular Events: A Secondary Study of Trial Sequential Analysis. *Frontiers in Pharmacology*, *11*(PG-592116), 592116. https://doi.org/10.3389/fphar.2020.592116

Zhao, C., Lai, L., Zhang, L., Cai, Z., Ren, Z., Shi, C., Luo, W., & Yan, Y. (2021). The effects of acceptance and commitment therapy on the psychological and physical outcomes among cancer patients: A meta-analysis with trial sequential analysis. *Journal of Psychosomatic Research*, *140*(PG-110304), 110304. https://doi.org/10.1016/j.jpsychores.2020.110304

Zhao, Y., Yang, M., Mao, Z., Yuan, R., Wang, L., Hu, X., Zhou, F., & Kang, H. (2019). The clinical outcomes of selenium supplementation on critically ill patients: A meta-analysis of randomized controlled trials. *Medicine (United States)*, *98*(20), e15473. https://doi.org/10.1097/MD.0000000000015473

Zhao, Z., Xu, K., Zhang, Y., Chen, G., & Zhou, Y. (2021). Quadratus lumborum block for postoperative analgesia after cesarean section: a meta-analysis of randomized controlled trials with trial sequential analysis. *Scientific Reports*, *11*(1), 18104. https://doi.org/10.1038/s41598-021-96546-7

Zheng, B., Zhang, X., Wang, X., Ge, L., Wei, M., Bi, L., Deng, X., Wang, Q., Li, J., & Wang, Z. (2020). A comparison of open, laparoscopic and robotic total mesorectal excision: trial sequential analysis and network meta-analysis. *Colorectal Disease*, *22*(4), 382–391. https://doi.org/10.1111/codi.14872

Zheng, H., Liu, M., Li, S., Shi, Q., Zhang, S., Zhou, Y., & Su, N. (2021). Sodium-Glucose Co-Transporter-2 Inhibitors in Non-Diabetic Adults With Overweight or Obesity: A Systematic Review and Meta-Analysis. *Frontiers in Endocrinology*, *12*(PG-706914), 706914. https://doi.org/10.3389/fendo.2021.706914

Zheng, H., Jin, S., Shen, Y. L., Peng, W. Y., Ye, K., Tang, T. C., Zhao, J., Chen, M., & Li, Z. G. (2021). Chinese Herbal Medicine for Irritable Bowel Syndrome: A Meta-Analysis and Trial Sequential Analysis of Randomized Controlled Trials. *Frontiers in Pharmacology*, *12*(PG-694741), 694741. https://doi.org/10.3389/fphar.2021.694741

Zheng, J., Du, L., Wang, J., Zhang, L., & Chen, G. (2021). Prewarming i-gel laryngeal mask for mechanical ventilation: A meta-analysis of randomised control trials and trial sequential analysis. *BMJ Open*, *11*(8), e045461. https://doi.org/10.1136/bmjopen-2020-045461

Zheng, Z., Luo, Y., Li, J., & Gao, J. (2021). Randomised trials of proton pump inhibitors for gastro-oesophageal reflux disease in patients with asthma: An updated systematic review and meta-analysis. *BMJ Open*, *11*(8), e043860. https://doi.org/10.1136/bmjopen-2020-043860

Zhong, D., Li, J., Yang, H., Li, Y., Huang, Y., Xiao, Q., Liu, T., & Jin, R. (2020). Tai Chi for Essential Hypertension: a Systematic Review of Randomized Controlled Trials. *Current Hypertension Reports*, *22*(3), 25. https://doi.org/10.1007/s11906-020-1031-y

Zhong, L., Ji, X. W., Wang, H. L., Zhao, G. M., Zhou, Q., & Xie, B. (2020). Non-catecholamine vasopressors in the treatment of adult patients with septic shock—evidence from meta-analysis and trial sequential analysis of randomized clinical trials. *Journal of Intensive Care*, *8*(1), 83. https://doi.org/10.1186/s40560-020-00500-0

Zhong, P.-Y., Shang, Y.-S., Bai, N., Ma, Y., Niu, Y., & Wang, Z.-L. (2021). Efficacy and Safety of Very Short-Term Dual Antiplatelet Therapy After Drug-Eluting Stents Implantation for Acute Coronary Syndrome: A Systematic Review and Meta-Analysis of Randomized Clinical Trials. *Frontiers in Cardiovascular Medicine*, *8*(PG-660360), 660360. https://doi.org/10.3389/fcvm.2021.660360

Zhou, J. B., Song, Z. H., Bai, L., Zhu, X. R., Li, H. B., & Yang, J. K. (2018). Could Intensive Blood Pressure Control Really Reduce Diabetic Retinopathy Outcomes? Evidence from Meta-Analysis and Trial Sequential Analysis from Randomized Controlled Trials. *Diabetes Therapy*, *9*(5), 2015–2027. https://doi.org/10.1007/s13300-018-0497-y

Zhou, J., Xu, J., Cheng, A., Li, P., Chen, B., & Sun, S. (2020). Effect of nicorandil treatment adjunctive to percutaneous coronary intervention in patients with acute myocardial infarction: a systematic review and meta-analysis. *Journal of International Medical Research*, *48*(11), 300060520967856. https://doi.org/10.1177/0300060520967856

Zhou, L., Chen, B., Sheng, L., & Turner, A. (2020). The effect of vitamin D supplementation on the risk of breast cancer: a trial sequential meta-analysis. *Breast Cancer Research and Treatment*, *182*(1), 1–8. https://doi.org/10.1007/s10549-020-05669-4

Zhou, P., Liu, J., Deng, X., & Li, Z. (2021). Biceps tenotomy versus tenodesis for lesions of the long head of the biceps tendon: A systematic review and meta-analysis of randomized controlled trials. *Medicine (United States)*, *100*(3), E23993. https://doi.org/10.1097/MD.0000000000023993

Zhou, X., Fang, H., Hu, C., Xu, J., Wang, H., Pan, J., Sha, Y., & Xu, Z. (2021). Effect of hypocaloric versus standard enteral feeding on clinical outcomes in critically ill adults – A meta-analysis of randomized controlled trials with trial sequential analysis. *Medicina Intensiva*, *45*(4), 211–225. https://doi.org/10.1016/j.medin.2019.10.003

Zhou, X., Xie, L., Altinel, Y., & Qiao, N. (2020). Assessment of Evidence Regarding Minimally Invasive Surgery vs. Conservative Treatment on Intracerebral Hemorrhage: A Trial Sequential Analysis of Randomized Controlled Trials. *Frontiers in Neurology*, *11*(PG-426), 426. https://doi.org/10.3389/fneur.2020.00426

Zhou, X., Fang, H., Xu, J., Chen, P., Hu, X., Chen, B., Wang, H., Hu, C., & Xu, Z. (2019). Stress ulcer prophylaxis with proton pump inhibitors or histamine 2 receptor antagonists in critically ill adults-a meta-analysis of randomized controlled trials with trial sequential analysis. *BMC Gastroenterology*, *19*(1), 193. https://doi.org/10.1186/s12876-019-1105-y

Zhou, X., Hu, C., Yao, L., Fan, Z., Sun, L., Wang, Y., & Xu, Z. (2018). Effect of adjunctive corticosteroids on clinical outcomes in adult patients with septic shock - a meta-analysis of randomized controlled trials and trial sequential analysis. *Journal of Critical Care*, *48*(PG-296-306), 296–306. https://doi.org/10.1016/j.jcrc.2018.09.013

Zhu, J., Zhang, W., Ou, N., Song, Y., Kang, J., Liang, Z., Hu, R., Yang, Y., & Liu, X. (2020). Do testosterone supplements enhance response to phosphodiesterase 5 inhibitors in men with erectile dysfunction and hypogonadism: A systematic review and meta-analysis. *Translational Andrology and Urology*, *9*(2), 591–600. https://doi.org/10.21037/tau.2020.01.13

Zhu, R. sen, Kan, S. li, Cao, Z. gang, Jiang, Z. hua, Zhang, X. li, & Hu, W. (2018). Secondary Surgery after Cervical Disc Arthroplasty versus Fusion for Cervical Degenerative Disc Disease: A Meta-analysis with Trial Sequential Analysis. *Orthopaedic Surgery*, *10*(3), 181–191. https://doi.org/10.1111/os.12401

Zhu, Y. J., Zhang, Y., Wang, T. Y., Zhao, J. T., Zhao, Z., Zhu, J. R., & Lan, C. H. (2020). High dose PPI-amoxicillin dual therapy for the treatment of Helicobacter pylori infection: a systematic review with meta-analysis. *Therapeutic Advances in Gastroenterology*, *13*(PG-1756284820937115), 1756284820937115. https://doi.org/10.1177/1756284820937115

Zhu, Y., Zhang, M., Zhang, R., Ye, X., & Wei, J. (2021). Extracorporeal membrane oxygenation versus mechanical ventilation alone in adults with severe acute respiratory distress syndrome: A systematic review and meta-analysis. *International Journal of Clinical Practice*, *75*(9), e14046. https://doi.org/10.1111/ijcp.14046

Zou, Y., Ma, K., Xiong, J. Bin, Xi, C. H., & Deng, X. J. (2018). Comparison of the effects of albumin and crystalloid on mortality among patients with septic shock: Systematic review with meta-analysis and trial

sequential analysis. *Sao Paulo Medical Journal*, *136*(5), 421–432. https://doi.org/10.1590/1516-3180.2017.0285281017

Zwager, C. L., Tuinman, P. R., De Grooth, H. J., Kooter, J., Ket, H., Fleuren, L. M., & Elbers, P. W. G. (2019). Why physiology will continue to guide the choice between balanced crystalloids and normal saline: A systematic review and meta-analysis. *Critical Care*, *23*(1), 366. https://doi.org/10.1186/s13054-019-2658-4

Supplemental Table 5. Data on study type and overall proportions

	Systematic review	Meta-analysis	Overall
**Number of TSA extracted**	312	312	624
Dichotomous outcomes	218 (69.9%)	221 (70.8%)	439 (70.4%)
Continuous outcomes	94 (30.1%)	91 (29.2%)	185 (29.6%)
**Extracted TSA was done on**			
Primary outcome	281 (90.1%)	287 (92.0%)	568 (91.0%)
Secondary outcome	30 (9.6%)	25 (8.0%)	55 (8.8%)
Exploratory outcome	1 (0.3%)	0	1 (0.2%)
**Model used**			
Random-effects model	224 (71.8%)	203 (65.1%)	427 (68.4%)
Fixed-effect model	53 (17.1%)	76 (24.4%)	129 (20.7%)
Fixed- and random-effects models	34 (10.9%)	30 (9.6%)	64 (10.3%)
Other[a]	0	1 (0.3%)	1 (0.2%)
Not mentioned	1 (0.3%)	2 (0.6%)	3 (0.5%)
**Number of trials included in TSA**[b]			
Mean (SD)	11.0 (9.3)	10.4 (8.6)	10.7 (8.9)
Median (IQR)	8 (5 to 14)	8 (5 to 13)	8 (5 to 13)
**Acquired information size**			
Mean (SD)	9,420 (73,100)	4,310 (10,500)	6,870 (52,300)
Median (IQR)	1,160 (551 to 3,300)	1,290 (597 to 3,410)	1,220 (594 to 3,310)
No extractable information	22 (7.1%)	22 (7.1%)	44 (7.1%)
**TSA results**			
Beneficial	117 (37.7%)	128 (41.0%)	245 (39.3%)
Harmful	19 (6.1%)	12 (3.8%)	31 (5.0%)
Futility	45 (14.5%)	44 (14.1%)	89 (14.3%)
Insignificant	92 (29.5%)	111 (35.6%)	203 (32.5%)
Uninterpretable	39 (12.5%)	17 (5.4%)	56 (9.0%)
**Alpha level chosen**			
< 0.025	11 (3.5%)	4 (1.3%)	15 (2.4%)
0.025 to 0.033	26 (8.4%)	5 (1.6%)	31 (5.0%)
0.033 to 0.05	14 (4.5%)	1 (0.3%)	15 (2.4%)
0.05	236 (75.6%)	273 (87.5%)	509 (81.6%)
0.1	0	1 (0.3%)	1 (0.2%)
Not reported	25 (8.0%)	28 (9.0%)	53 (8.5%)
**Power chosen (*1-beta*)**			
< 0.8	1 (0.3%)	0 (0%)	1 (0.2%)
0.85	0 (0%)	1 (0.3%)	1 (0.2%)
0.9	194 (62.2%)	245 (75.0%)	428 (68.6%)
> 0.9	1 (0.3%)	2 (0.6%)	3 (0.5%)
Not reported	116 (37.2%)	75 (24.0%)	191 (30.6%)
**Heterogeneity**			
D^2	125 (40.1%)	85 (27.2%)	209 (33.7%)
I^2	16 (5.1%)	27 (8.7%)	43 (6.9%)
No heterogeneity corrected in TSA	15 (4.8%)	19 (6.1%)	34 (5.4%)
Not described / not clear	115 (36.9%)	128 (41.0%)	243 (38.9%)
Not described, but $I^2=0\%$	41 (13.1%)	53 (17.0%)	94 (15.1%)
**How was the required information size presented?**			
DARIS	108 (34.6%)	93 (29.8%)	201 (32.2%)

RIS (unadjusted)	48 (15.4%)	46 (14.7%)	94 (15.1%)
RIS not presented	18 (5.8%)	9 (2.9%)	27 (4.3%)
Unclear[c]	138 (44.2%)	164 (52.6%)	302 (48.4%)
**TSA-adjusted CI included**			
No	226 (72.4%)	274 (87.8%)	500 (80.1%)
Yes	61 (19.6%)	24 (7.7%)	85 (13.6%)
Yes, but mislabelled	18 (5.8%)	13 (4.2%)	31 (5.0%)
Not reported[d]	7 (2.3%)	1 (0.3%)	8 (1.3%)
**Percentage AIS over RIS**			
Mean (SD)	137 (285)	136 (228)	136 (258)
Median (IQR)	79.5 (32.8 to 137.0)	75.2 (37.5 to 136.0)	76.8 (35.2 to 137.0)
Not reported	40 (12.8%)	37 (11.9%)	77 (12.3%)
**Transparency**			
Excellent	55 (17.6%)	15 (4.8%)	70 (11.2%)
Good	74 (23.7%)	71 (22.8%)	145 (23.2%)
Poor	72 (23.1%)	102 (32.7%)	174 (27.9%)
Very poor	111 (35.6%)	124 (39.7%)	235 (37.7%)

AIS: acquired information size; D2: diversity; DARIS: diversity adjusted required information size; I2: inconsistency; Meta-analysis: a non-systematic approach which lacks a pre-published protocol at the time of data extraction; OR: odds ratio; Pc: proportion of events in the control group; Peto OR: Peto odds ratio; RD: risk difference; RIS: required information size; RR: risk ratio; RRR: relative risk reduction; Systematic review: a detailed, organised, and transparent method of gathering, appraising and synthesising data to answer a well-defined question, including a pre-published protocol before starting data extraction; TSA: Trial Sequential Analysis; TSA adjusted CI: Trial Sequential Analysis adjusted confidence interval;

a. One study used empirical Bayes binary random effect;

b. Missing value in 5 systematic reviews;

c. Unclear if RIS was adjusted or unadjusted;

d. Missing either AIS, RIS, or both.

Supplemental Material B. Subgroup comparing Cochrane reviews to non-Cochrane reviews

The 27 Cochrane reviews performed better on most parameters. We extracted 32 Trial Sequential Analysis of which 23 (62%) were on dichotomous outcomes and nine (28%) on continuous outcomes. The AMSTAR 2 evaluation showed that 20/27 (74%) were evaluated as high confidence, 2/27 (7%) as moderate confidence, 1/27 (4%) as low confidence, and 4/27 (15%) critically low confidence (**Supplemental Table 5**).

Eighteen of 23 (78%) dichotomous outcomes were primary outcomes and included a median of 4 (IQR 2 to 13) trials, with a median acquired information size of 643 (IQR 213 to 1,320) (**Supplemental Table 6**). Six of nine (66%) of the continuous outcomes were categorised as primary outcomes with a median number of trials of 9 (IQR 4 to 16), and a median acquired information size of 627 (IQR 313 to 1,310) (**Supplemental Table 7**). The DARIS was presented in 18 (78.3%) of the dichotomous and 7 (77.8%) of the continuous outcomes.

For dichotomous outcomes the relative risk was used in 20 of 23 (96%) analyses (**Supplemental Table 6**). In analyses of the continuous outcomes, the mean difference was used in 7 (78%) and the standardised mean difference in 2 (22%) (**Supplemental Table 7**). All Cochrane reviews reported the proportion of events in the control group, however, three (13%) of these did not provide the value for the proportion of events in the control group. The proportion from the current forest plot was used in 22 (81%) of analyses, and one from a previously published trial. The relative risk reduction was reported in 96% of analyses with 8 (34%) studies not reporting the rationale, three (13%) studies reporting that the value was predefined from a previous study, six studies (26%) used the current data from the forest plot, and five (22%) used clinical experience (**Supplemental Table 6**).

The alpha level was 5% in 11 (33%) analyses and lower for the remaining 21 (66%) Cochrane reviews. The power was set at 0.9 in 12 (38%) and not reported for 20 (62%) analyses. D^2 was used in 20/23 (91%) of the dichotomous outcomes and 9/9 (100%) of the continuous outcomes. Three did not report the use of heterogeneity adjustment (**Supplemental Table 6 and 7**).

Transparency of the Trial Sequential Analysis was considered excellent in 21/32 (66%), good in three (9%), poor in five (16%), and very poor in three (9%) (**Supplemental Table 6 and 7**).

Sixteen of 32 (50%) analyses had a Trial Sequential Analysis adjusted confidence interval. D^2 was used to calculate the required information size in 18/23 (78%) of dichotomous outcomes, while 7/9 (78%) of continuous outcomes used D^2. The remaining dichotomous outcomes did not present a required information size and two continuous outcomes used I^2. The median percentage acquired information size over the D^2-, I^2, or non-adjusted required information size was 12% (IQR 7% to 22%) for dichotomous outcomes and 61% (IQR 55% to 139%) for continuous outcomes (**Supplemental Table 6 and 7**).

Supplemental Table 6. Study characteristics for Cochrane reviews and non-Cochrane reviews

	Cochrane reviews	Non-Cochrane reviews
**Publications**	27	243
**Publication year**		
2018	14 (51.9%)	38 (15.8%)
2019	7 (25.9%)	48 (19.9%)
2020	2 (7.4%)	65 (27.0%)
2021	4 (14.8%)	92 (37.9%)
**AMSTAR 2 evaluation**		
High	20 (74.1%)	7 (2.9%)
Moderate	2 (7.4%)	16 (6.6%)
Low	1 (3.7%)	34 (14.0%)
Critically low	4 (14.8%)	186 (76.5%)
**Number of TSA extracted**	32	280
Dichotomous outcomes	23 (71.9%)	195 (69.6%)
Continuous outcomes	9 (28.1%)	85 (30.4%)
**Planned details on TSA in protocol**	21 (77.8%)	113 (46.9%)
Planned dichotomous outcomes	19 (70.4%)	83 (34.2%)
Planned continuous outcomes	13 (48.1%)	54 (22.2%)
**Used GRADE**	27 (100%)	167 (68.7%)
**TSA affected GRADE evaluation**	15 (55.6%)	51 (21.0%)

AMSTAR: Assessing the methodological quality of systematic reviews; GRADE: Grading of Recommendations Assessment, Development and Evaluation; Meta-analysis: a non-systematic approach which lacks a pre-published protocol at the time of data extraction; Systematic review: a detailed, organised, and transparent method of gathering, appraising and synthesising data to answer a well-defined question, including a pre-published protocol before starting data extraction; TSA: Trial Sequential Analysis;

Supplemental Table 7. Dichotomous outcomes for Cochrane reviews and non-Cochrane reviews

	Cochrane reviews	Non-Cochrane reviews
**Number of TSA extracted**	23	195
**Extracted TSA was done on**		
Primary outcome	18 (78.3%)	182 (93.3%)
Secondary outcome	5 (21.7%)	13 (6.7%)
Exploratory outcome	0	0
**Model used**		
Random-effects model	13 (56.6%)	128 (65.6%)
Fixed-effect model	2 (8.7%)	45 (23.1%)
Fixed- and random-effects models	8 (34.8%)	21 (10.8%)
Not mentioned	0	1 (0.5%)
**Dichotomous effect estimates used**		
RR	20 (87.0%)	154 (79.0%)
OR	2 (8.7%)	37 (19.0%)
Peto OR	1 (4.3%)	1 (0.5%)
RD	0	1 (0.5%)
Other[a]	0	2 (1.0%)
**Number of trials included in TSA**[b]		
Mean (SD)	9.0 (9.2)	11.3 (9.0)
Median (IQR)	4.0 (2.0 to 13.0)	9.00 (5.0 to 13.0)
Not reported	1 (4.3%)	3 (1.5%)
**Acquired information size**		
Mean (SD)	1,020 (1,130)	14,500 (92,500)
Median (IQR)	643 (213 to 1,320)	1,690 (771 to 5,680)
No extractable information	1 (4.3%)	15 (7.7%)
**TSA results**		
Beneficial	1 (4.3%)	66 (34.2%)
Harmful	1 (4.3%)	11 (5.6%)
Futility	1 (4.3%)	33 (16.9%)
Insignificant	12 (52.2%)	64 (3.8%)
Uninterpretable	8 (34.8%)	21 (10.8%)
**Was the Pc presented?**		
Yes, the value was reported	20 (87.0%)	90 (46.2%)
Yes, but no value was reported	3 (13.0%)	29 (14.9%)
No	0	76 (39.0%)
**How was Pc selected?**		
Previously published data[c]	1 (4.3%)	4 (2.1%)
From current forest plot	22 (95.7%)	90 (46.2%)
Not mentioned	0	14 (7.2%)
Other	0	0
Unclear	0	9 (4.6%)
**Was the RRR presented?**		
Yes	22 (95.7%)	147 (75.4%)
No	1 (4.3%)	48 (24.6%)
**How was RRR selected?**		
Previously published data[c]	3 (13.0%)	11 (5.7%)
From the current forest plot	6 (26.1%)	30 (15.5%)
From author's clinical experience	5 (21.7%)	23 (11.9%)

From other sources	4 (17.4%)	31 (16.1%)
Not mentioned	8 (34.8%)	65 (33.3%)
**Level of RRR used in outcomes**		
0 to 4.9%	0	1 (0.5%)
5 to 9.9%	1 (4.3%)	5 (2.6%)
10 to 14.9%	2 (8.7%)	28 (14.4%)
15 to 19.9%	3 (13.0%)	19 (9.7%)
20 to 24.9%	12 (52.2%)	50 (25.6%)
25 to 29.9%	0	11 (5.6%)
30 to 39.9%	0	12 (6.2%)
40 to 49.9%	0	5 (2.6%)
>50%	2 (8.7%)	10 (5.1%)
**Alpha level chosen**		
< 0.025	1 (4.3%)	3 (1.5%)
0.025 to 0.033	12 (52.2%)	9 (4.6%)
0.033 to 0.05	2 (8.7%)	11 (5.6%)
0.05	8 (34.8%)	156 (80.0%)
0.1	0	0
Not reported	0	16 (8.2%)
**Power chosen (*1-beta*)**		
0.9	9 (39.1%)	125 (64.1%)
Not reported	14 (60.9%)	68 (34.9%)
**Heterogeneity**		
D^2	20 (87.0%)	72 (36.9%)
I^2	0	11 (5.6%)
No heterogeneity corrected in TSA	0	8 (4.1%)
Not described / not clear	3 (13.0%)	68 (34.9%)
Not described, but I^2*=0%*	0	36 (18.5%)
**How was the required information size presented?**		
DARIS	18 (78.3%)	64 (32.8%)
RIS (unadjusted)	0	32 (16.4%)
RIS not presented	4 (17.4%)	10 (5.1%)
Unclear[d]	1 (4.3%)	89 (45.6%)
**TSA-adjusted CI included**		
Yes	11 (47.8%)	35 (17.9%)
Yes, but mislabelled	0	14 (7.2%)
No	7 (30.4%)	144 (73.8%)
Not reported	5 (21.7%)	2 (1.0%)
**Percentage AIS over RIS**		
Mean (SD)	19.6 (22.2)	134 (326)
Median (IQR)	12.3 (6.5 to 21.5)	70.3 (30.7 to 134)
Not reported[e]	4 (17.4%)	26 (13.5%)
**Transparency**		
Excellent	17 (73.9%)	28 (14.4%)
Good	1 (4.3%)	57 (29.2%)
Poor	3 (13.0%)	45 (23.1%)
Very poor	2 (8.7%)	65 (33.3%)

AIS: acquired information size; D2: diversity; DARIS: diversity adjusted required information size; I2: inconsistency; Meta-analysis: a non-systematic approach which lacks a pre-published protocol at the time of

data extraction; OR: odds ratio; Pc: proportion of events in the control group; Peto OR: Peto odds ratio; RD: risk difference; RIS: required information size; RR: risk ratio; RRR: relative risk reduction; Systematic review: a detailed, organised, and transparent method of gathering, appraising and synthesising data to answer a well-defined question, including a pre-published protocol before starting data extraction; TSA: Trial Sequential Analysis; TSA adjusted CI: Trial Sequential Analysis adjusted confidence interval;

a. Preferred alternative method was hazard ratio;

b. Missing value in 4 systematic reviews;

c. Previous systematic reviews, randomised clinical trials or observational studies;

d. Unclear if RIS was adjusted or unadjusted;

e. Missing either AIS, RIS, or both.

Supplemental Table 8. Continuous outcomes for Cochrane reviews and non-Cochrane reviews

	Cochrane reviews	Non-Cochrane reviews
**Number of TSA extracted**	9	85
**Extracted TSA was done on**		
Primary outcome	6 (66.7%)	75 (88.2%)
Secondary outcome	2 (22.2%)	10 (11.8%)
Exploratory outcome	1 (11.1%)	0
**Model used**		
Random-effects model	8 (88.9%)	75 (88.2%)
Fixed-effect model	0	6 (7.1%)
Fixed- and random-effects models	1 (11.1%)	4 (4.7%)
**Continuous effect estimates used**		
Mean difference	7 (77.8%)	77 (90.6%)
Standardised mean difference	2 (22.2%)	7 (8.2%)
Other[a]	0	1 (1.2%)
**Number of trials included in TSA**		
Mean (SD)	14.7 (17.1)	10.4 (8.8)
Median (IQR)	9.0 (4.0 to 16.0)	8.0 (5.8 to 12.3)
**Acquired information size**		
Mean (SD)	1,080 (1,220)	1,090 (1,280)
Median (IQR)	627 (313 to 1,310)	691 (405 to 1,140)
No extractable information	0	6 (7.1%)
**TSA results**		
Beneficial	3 (33.3%)	47 (55.3%)
Harmful	2 (22.2%)	5 (5.9%)
Futile	2 (22.2%)	9 (10.6%)
Insignificant	1 (11.1%)	15 (17.6%)
Uninterpretable	1 (11.1%)	9 (10.6%)
**Was the minimally relevant difference presented**		
Yes	7 (77.8%)	33 (38.8%)
No	2 (22.2%)	52 (61.2%)
**Was variance presented**		
Yes	6 (66.7%)	23 (27.1%)
No	3 (33.3%)	62 (72.9%)
**Alpha level chosen**		
< 0.025	4 (44.4%)	3 (3.5%)
0.025 to 0.033	1 (11.1%)	(4.7%)
0.033 to 0.05	1 (11.1%)	0
0.05	3 (33.3%)	69 (81.2%)
Not reported	0	9 (10.6%)
**Power chosen (*1-beta*)**		
0.9[b]	3 (33.3%)	57 (67.1%)
Not reported	6 (66.7%)	28 (32.9%)
**Heterogeneity**		
D^2	9 (100%)	24 (28.2%)
I^2	0	5 (5.9%)

No heterogeneity corrected in TSA	0	7 (8.2%)
Not described / not clear	0	44 (51.8%)
Not described, but I^2=0%	0	5 (5.9%)
**How was the RIS presented?**		
DARIS	7 (77.8%)	19 (22.4%)
RIS (unadjusted)	2 (22.2%)	46 (54.1%)
RIS not presented	0	4 (4.7%)
Unclear[c]	0	16 (18.8%)
**TSA adjusted CI included**		
Yes	4 (44.4%)	11 (12.9%)
Yes, but mislabelled	1 (11.1%)	3 (3.5%)
No	4 (44.4%)	71 (83.5%)
**Percentage AIS over RIS**		
Mean (SD)	118 (107)	174 (225)
Median (IQR)	61.1 [55.1,139]	105 [68.2,180]
Missing	0	10 (11.8%)
**Transparency**		
Excellent	4 (44.4%)	6 (7.1%)
Good	2 (22.2%)	14 (16.5%)
Poor	2 (22.2%)	22 (25.9%)
Very poor	1 (11.1%)	43 (50.6%)

AIS: acquired information size; D2: diversity; DARIS: diversity adjusted required information size; I2: inconsistency; Meta-analysis: a non-systematic approach which lacks a pre-published protocol at the time of data extraction; RIS: required information size; Systematic review: a detailed, organised, and transparent method of gathering, appraising and synthesising data to answer a well-defined question, including a pre-published protocol before starting data extraction; TSA: Trial Sequential Analysis; TSA adjusted CI: Trial Sequential Analysis adjusted confidence interval;

a. Hedges' g;

b. Studies only reported a power of 0.9;

c. Unclear if RIS was adjusted or unadjusted.

Supplemental Table 9. Agreement between two reviewers on selected extraction variables

Question	Kappa	95% confidence interval
How many RCTs were included in the article?	*0.92*	0.90 to 0.93
Were the downgrade methods for imprecision described?	*0.65*	0.54 to 0.77
What did the authors conclude about the intervention effect on the outcome(s) analysed with trial sequential analysis?	*0.68*	0.63 to 0.73
How much was imprecision downgraded?	*0.65*	0.54 to 0.77
Was the alpha-level defined?	*0.72*	0.66 to 0.78
Was the power defined?	*0.81*	0.75 to 0.86
Was heterogeneity correction used for trial sequential analysis?	*0.50*	0.46 to 0.53
Was proportion of events in the control group (Pc) defined?	*0.77*	0.69 to 0.84
Was the relative risk reduction (RRR) defined?	*0.85*	0.75 to 0.94
Was variance defined?	*0.75*	0.60 to 0.90
Was the minimally relevant difference defined?	*0.70*	0.55 to 0.85
Please rate the overall transparency of the trial sequential analysis	*0.50*	0.46 to 0.55

1. ID

Record ID

____

Standard procedure for data extraction

General workflow Source files via Covidence: Major mistakes and Errors conducted using Trial Sequential Analysis in systematic reviews or meta-analyses (METSA) (covidence.org) Filter by #CovidenceID Add missing files to covidence (e.g. protocol or supplementary material)   Go through REDcap instruments 1-5 in the pre-specified order

Handling missing data or incompatibility When a numerical value is required but none is available, input: "-99" When text is required but none is available, input: "NA" If question is missing appropriate answer, consult JBM

Misc. When citing paper, place citation in " " If in doubt about any of the above, consult CGR or JBM

Reviewer identification

- ◯ BK
- ◯ CGR
- ◯ CL
- ◯ CM
- ◯ EBP
- ◯ JBM
- ◯ JHPR
- ◯ JHS
- ◯ JPS
- ◯ MA
- ◯ MHO
- ◯ SHH
- ◯ VW
- ◯ ZYL
- ◯ Not assigned

REDcap extraction number

____

CovidenceID #

____

DOI-number of the systematic review

____
(Copy/paste the record number)

Provide first author surname and publication year

____
(e.g. Johnson 2021)

In which country did the corresponding author have it's address/primary affiliation?

- ◯ Afghanistan
- ◯ Albania
- ◯ Algeria
- ◯ Andorra
- ◯ Angola
- ◯ Antigua & Deps
- ◯ Argentina
- ◯ Armenia
- ◯ Australia
- ◯ Austria
- ◯ Azerbaijan
- ◯ Bahamas
- ◯ Bahrain
- ◯ Bangladesh
- ◯ Barbados
- ◯ Belarus
- ◯ Belgium
- ◯ Belize
- ◯ Benin
- ◯ Bhutan
- ◯ Bolivia
- ◯ Bosnia Herzegovina
- ◯ Botswana
- ◯ Brazil
- ◯ Brunei
- ◯ Bulgaria
- ◯ Burkina
- ◯ Burundi
- ◯ Cambodia
- ◯ Cameroon
- ◯ Canada
- ◯ Cape Verde
- ◯ Central African Rep
- ◯ Chad
- ◯ Chile
- ◯ China
- ◯ Colombia
- ◯ Comoros
- ◯ Congo
- ◯ Congo (Democratic Rep)
- ◯ Costa Rica
- ◯ Croatia
- ◯ Cuba
- ◯ Cyprus
- ◯ Czech Republic
- ◯ Denmark
- ◯ Djibouti
- ◯ Dominica
- ◯ Dominican Republic
- ◯ East Timor
- ◯ Ecuador
- ◯ Egypt
- ◯ El Salvador
- ◯ Equatorial Guinea
- ◯ Eritrea
- ◯ Estonia
- ◯ Ethiopia
- ◯ Fiji
- ◯ Finland
- ◯ France
- ◯ Gabon
- ◯ Gambia
- ◯ Georgia
- ◯ Germany
- ◯ Ghana
- ◯ Greece
- ◯ Grenada
- ◯ Guatemala
- ◯ Guinea

◯ Guinea-Bissau
◯ Guyana
◯ Haiti
◯ Honduras
◯ Hungary
◯ Iceland
◯ India
◯ Indonesia
◯ Iran
◯ Iraq
◯ Ireland {Republic}
◯ Israel
◯ Italy
◯ Ivory Coast
◯ Jamaica
◯ Japan
◯ Jordan
◯ Kazakhstan
◯ Kenya
◯ Kiribati
◯ Korea North
◯ Korea South
◯ Kosovo
◯ Kuwait
◯ Kyrgyzstan
◯ Laos
◯ Latvia
◯ Lebanon
◯ Lesotho
◯ Liberia
◯ Libya
◯ Liechtenstein
◯ Lithuania
◯ Luxembourg
◯ Macedonia
◯ Madagascar
◯ Malawi
◯ Malaysia
◯ Maldives
◯ Mali
◯ Malta
◯ Marshall Islands
◯ Mauritania
◯ Mauritius
◯ Mexico
◯ Micronesia
◯ Moldova
◯ Monaco
◯ Mongolia
◯ Montenegro
◯ Morocco
◯ Mozambique
◯ {Burma}
◯ Namibia
◯ Nauru
◯ Nepal
◯ Netherlands
◯ New Zealand
◯ Nicaragua
◯ Niger
◯ Nigeria
◯ Norway
◯ Oman
◯ Pakistan
◯ Palau
◯ Panama
◯ Papua New Guinea
◯ Paraguay
◯ Peru
◯ Philippines
◯ Poland

◯ Portugal
◯ Qatar
◯ Romania
◯ Russian Federation
◯ Rwanda
◯ St Kitts & Nevis
◯ St Lucia
◯ Saint Vincent & the Grenadines
◯ Samoa
◯ San Marino
◯ Sao Tome & Principe
◯ Saudi Arabia
◯ Senegal
◯ Serbia
◯ Seychelles
◯ Sierra Leone
◯ Singapore
◯ Slovakia
◯ Slovenia
◯ Solomon Islands
◯ Somalia
◯ South Africa
◯ South Sudan
◯ Spain
◯ Sri Lanka
◯ Sudan
◯ Suriname
◯ Swaziland
◯ Sweden
◯ Switzerland
◯ Syria
◯ Taiwan
◯ Tajikistan
◯ Tanzania
◯ Thailand
◯ Togo
◯ Tonga
◯ Trinidad & Tobago
◯ Tunisia
◯ Turkey
◯ Turkmenistan
◯ Tuvalu
◯ Uganda
◯ Ukraine
◯ United Arab Emirates
◯ United Kingdom
◯ United States
◯ Uruguay
◯ Uzbekistan
◯ Vanuatu
◯ Vatican City
◯ Venezuela
◯ Vietnam
◯ Yemen
◯ Zambia
◯ Zimbabwe

CTU affiliated?	◯ Yes<br>◯ No

Which files were evaluated for this entry?	☐ Main paper<br>☐ Supplementary materials<br>☐ Prepublished protocol<br>☐ Pretrial registration (PROSPERO or elsewhere)<br>☐ Other<br>(Include also material, that didn't contain relevant information, e.g., supplementary material when not relevant for TSA |||| To make a pdf searchable, use OCR like: https://online2pdf.com/convert-scanned-pdf-to-searchable-pdf#)
Please describe which files were evaluated	______________________
Study details	
Choose the appropriate study type	◯ Systematic review (with pre-published/pre-registred protocol)<br>◯ Meta-analysis (without pre-published/pre-registred protocol)<br>(Systematic reviews aim to minimize bias through the use of pre-specified research questions and methods that are documented in protocols)
Was the review an update of a previous systematic review?	◯ Yes<br>◯ No
When the review is an update of an existing review, please identify the protocol for updated review. If no protocol has been made specifically for the updated review, assess whether the updated review explicitly and firmly claims to adhere to methods defined in previous protocols/review versions. Otherwise, mark the updated review as having no predefined protocol.	
Please describe the review history (e.g. list previous versions, protocols, and describe if the update was performed by a different author group)	______________________
Has the protocol been made public before data extraction began?	◯ Yes<br>◯ No<br>◯ Unclear
Was this a Cochrane Review?	◯ Yes<br>◯ No
Medical field and intervention type	
What was the overall research question?	______________________

What is the medical field(s) of interest in the article?

- ☐ Allergy and immunology
- ☐ Alternative medicine
- ☐ Anesthesiology
- ☐ Cardiology
- ☐ Clinical biochemistry
- ☐ Dentistry
- ☐ Dermatology
- ☐ Diagnostic radiology
- ☐ Emergency medicine
- ☐ Endocrinology
- ☐ Family medicine
- ☐ Gastroenterology
- ☐ Gastroentestinal surgery
- ☐ Genetics
- ☐ Geriatric medicine
- ☐ Gynecology
- ☐ Hematology
- ☐ Hepatology
- ☐ Infectious diseases
- ☐ Intensive care
- ☐ Internal medicine
- ☐ Medical genetics
- ☐ Nephrology
- ☐ Neurology
- ☐ Neurosurgery
- ☐ Nuclear medicine
- ☐ Obstetrics
- ☐ Oncology
- ☐ Ophthalmology
- ☐ Orthopedic surgery
- ☐ Oto-rhino-laryngology (ear-nose-throat)
- ☐ Palliative care
- ☐ Pathology
- ☐ Pediatrics
- ☐ Pediatric psychiatry
- ☐ Physical medicine and rehabilitation
- ☐ Plastic surgery
- ☐ Preventive medicine
- ☐ Pulmonary diseases
- ☐ Psychiatry
- ☐ Thoracic surgery
- ☐ Urology
- ☐ Vascular surgery
- ☐ Other

Please describe the medical field of interest in the article

____

What was the overall goal(s) with the intervention?

- ☐ Cure from disease
- ☐ Prevention of disease
- ☐ Prevention of complication to treatment (incl. perioperative care)
- ☐ Disease control
- ☐ Rehabilitation
- ☐ Palliation (pain management)
- ☐ Diagnostic
- ☐ Life support
- ☐ Other

Please describe the goal of the intervention

____

Which intervention type(s) was examined in the article?

- ☐ Pharmacological
- ☐ Surgical
- ☐ Psychotherapy
- ☐ Diagnostic
- ☐ Medical device
- ☐ Nutritional
- ☐ Exercise/rehabilitation
- ☐ Lifestyle
- ☐ Alternative
- ☐ Other

Which intervention was examined in the article?

____

To which specific diagnosis/health issue was the intervention applied?

____

2. Quality assessment (AMSTAR 2)

Record ID	______________________
Quality assessment of systematic reviews	
AMSTAR 2 original guide for quality assessment of systematic reviews<br><br>[Attachment: "AMSTAR 2-Guidance-document.pdf"]	
AMSTAR 2 original questionaire<br><br>[Attachment: "AMSTAR-2.pdf"]	
Original publication AMSTAR 2<br><br>[Attachment: "Shea 2017.pdf"]	
AMSTAR 2	
If study design including statistical analysis plan was not defined prior to data extraction (e.g. in a pre-published protocol), skip ahead and rate as "Critically low"	☐ No protocol or protocol not publicly available prior to data extraction<br>☐ No critical flaws immediately identified
If study design including statistical analysis plan was not defined prior to data extraction (e.g. in a pre-published protocol), skip ahead and rate as "Critically low"	○ Study design including statistical analysis plan not defined prior to data extraction<br>○ No critical flaws immediately identified
1. Did the research questions and inclusion criteria for the review include the components of PICO?	☐ Population<br>☐ Intervention<br>☐ Comparator group<br>☐ Outcome<br>☐ Timeframe for follow-up<br>☐ None of the above
2 = Yes | 1 = Partial yes | 0 = No	______________________
Did the "PICO" question reveal any moderate or critical weaknesses?	○ Yes, critical weakness<br>○ Yes, moderate weakness<br>○ No
Briefly describe the weakness	______________________

2. Did the review explicitly state that the review methods were established prior to the conduct of the review and did the report justify any significant deviations from the protocol?<br><br>The authors state that they had a written protocol or guide that included:	☐ Review question(s)<br>☐ A search strategy<br>☐ Inclusion/exclusion criteria<br>☐ A risk of bias assessment<br>☐ A meta-analysis/synthesis plan, if appropriate, and<br>☐ A plan for investigating causes of heterogeneity<br>☐ Justification for any deviations from the protocol (check also if NA)<br>☐ No
2 = Yes | 1 = Partial yes | 0 = No	______________________
Did the "protocol" question reveal any moderate or critical weaknesses?	◯ Yes, critical weakness<br>◯ Yes, moderate weakness<br>◯ No
Briefly describe the weakness	______________________
3. Did the review authors explain their selection of the study designs for inclusion in the review?	◯ Explanation for including only RCTs<br>◯ Explanation for including only NRSI<br>◯ Explanation for including both RCTs and NRSI<br>◯ None of the above<br>(Regarding missing explanation for including RCT's only: When many RCT's are available or the meta-analysis seek to find a "final conclusion" regarding intervention effects, it can be considered acceptable to not explain why only RCT's were included as this is standard practice. However, for more exploratory meta-analyses, non-RCT's should generally be included, and if they are not, the authors should explain why they weren't included. It is up to you to judge if the lack of explanation is acceptable or not.)
2 = Yes | 1 = Partial yes | 0 = No	______________________
Did the "design" question reveal any moderate or critical weaknesses?	◯ Yes, critical weakness<br>◯ Yes, moderate weakness<br>◯ No
Briefly describe the weakness	______________________

4. Did the review authors use a comprehensive literature search strategy?	☐ Searched at least 2 databases (relevant to research question)<br>☐ Provided keyword and/or search strategy (including timeframe)<br>☐ Justified publication restrictions (e.g. language)<br>☐ Searched the reference lists/bibliographies of included studies<br>☐ Searched trial/study registries<br>☐ Included/consulted content experts in the field<br>☐ Where relevant, searched for grey literature<br>☐ Conducted search within 24 months of completion of the review<br>☐ None of the above
2 = Yes | 1 = Partial yes | 0 = No	________________________
Did the "search" question reveal any moderate or critical weaknesses?	○ Yes, critical weakness<br>○ Yes, moderate weakness<br>○ No
Briefly describe the weakness	______________________________
5. Did the review authors perform study selection in duplicate?	○ At least two reviewers independently agreed on selection of eligible studies and achieved consensus on which studies to include<br>○ Two reviewers selected a sample of eligible studies and achieved good agreement (at least 80 percent), with the remainder selected by one reviewer.<br>○ No
2 = Yes | 1 = Partial yes | 0 = No	________________________
Did the "selection" question reveal any moderate or critical weaknesses?	○ Yes, critical weakness<br>○ Yes, moderate weakness<br>○ No
Briefly describe the weakness	______________________________
6. Did the review authors perform data extraction in duplicate?	○ At least two reviewers achieved consensus on which data to extract from included studies<br>○ Two reviewers extracted data from a sample of eligible studies and achieved good agreement (at least 80 percent), with the remainder extracted by one reviewer.<br>○ No
2 = Yes | 1 = Partial yes | 0 = No	________________________
Did the "extraction" question reveal any moderate or critical weaknesses?	○ Yes, critical weakness<br>○ Yes, moderate weakness<br>○ No

Briefly describe the weakness

____

7. Did the review authors provide a list of excluded studies and justify the exclusions?

- ☐ Provided a list of all potentially relevant studies that were read in full-text form but excluded from the review
- ☐ Justified the exclusion from the review of each potentially relevant study
- ☐ None of the above

(Applies only to studies excluded in full-text screening)

2 = Yes | 1 = Partial yes | 0 = No

____

Did the "exclusion" question reveal any moderate or critical weaknesses?

- ◯ Yes, critical weakness
- ◯ Yes, moderate weakness
- ◯ No

Briefly describe the weakness

____

8. Did the review authors describe the included studies in adequate detail (in relation to the PICO structure)?

- ☐ 1. Described populations
- ☐ 2. Described interventions
- ☐ 3. Described comparators
- ☐ 4. Described outcomes
- ☐ 5. Described research designs
- ☐ 6. Described populations in detail
- ☐ 7. Described interventions in detail (including doses where relevant)
- ☐ 8. Described comparators in detail (including doses where relevant)
- ☐ 9. Described study settings
- ☐ 10. Timeframe for follow-up
- ☐ No

2 = Yes | 1 = Partial yes | 0 = No

____

Did the "details" question reveal any moderate or critical weaknesses?

- ◯ Yes, critical weakness
- ◯ Yes, moderate weakness
- ◯ No

Briefly describe the weakness

____

9. Did the review authors use a satisfactory technique for assessing the risk of bias (RoB) in individual studies that were included in the review?<br><br>RCT's	☐ Unconcealed allocation<br>☐ Lack of blinding of patients and assessors when assessing outcomes (unnecessary for objective outcomes such as all-cause mortality)<br>☐ Allocation sequence that was not truly random<br>☐ Selection of the reported result from among multiple measurements or analyses of a specified outcome<br>☐ Includes only non-randomized studies of interventions (NRSI)<br>☐ None of the above<br>(Please read if in doubt: Chapter 8: Assessing risk of bias in a randomized trial)
2 = Yes | 1 = Partial yes | 0 = No	__________________________
Did the "RoB for RCT" question reveal any moderate or critical weaknesses?	◯ Yes, critical weakness<br>◯ Yes, moderate weakness<br>◯ No
Briefly describe the weakness	________________________________
9. Did the review authors use a satisfactory technique for assessing the risk of bias (RoB) in individual studies that were included in the review?<br><br>NRSI's	☐ Assessed RoB from confounding<br>☐ Assessed RoB from selection bias<br>☐ Methods to ascertain exposures and outcomes<br>☐ Selection of the reported result from among multiple measurements or analyses of a specified outcome<br>☐ Includes only RCT's<br>☐ None of the above
2 = Yes | 1 = Partial yes | 0 = No	__________________________
Did the "RoB for NRSI" question reveal any moderate or critical weaknesses?	◯ Yes, critical weakness<br>◯ Yes, moderate weakness<br>◯ No
Briefly describe the weakness	________________________________
10. Did the review authors report on the sources of funding for the studies included in the review?	◯ Yes ◯ No<br>(Must have reported on the sources of funding for individual studies included in the review. Note: Reporting that the reviewers looked for this information but it was not reported by study authors also qualifies)
2 = Yes | 1 = Partial yes | 0 = No	__________________________
Did the "funding" question reveal any moderate or critical weaknesses?	◯ Yes, critical weakness<br>◯ Yes, moderate weakness<br>◯ No

Briefly describe the weakness	__________________________________
11. Did the review authors use appropriate methods for statistical combination of results from RCT's?	☐ The authors justified combining the data in a meta-analysis<br>☐ They used an appropriate weighted technique to combine study results and adjusted for heterogeneity if present.<br>☐ They investigated the causes of any heterogeneity<br>☐ None of the above
2 = Yes | 1 = Partial yes | 0 = No	______________________________
Did the "meta analysis for RCT" question reveal any moderate or critical weaknesses?	◯ Yes, critical weakness<br>◯ Yes, moderate weakness<br>◯ No
Briefly describe the weakness	__________________________________
11. Did the review authors use appropriate methods for statistical combination of results from NRSI's?	☐ The authors justified combining the data in a meta-analysis<br>☐ They used an appropriate weighted technique to combine study results and adjusted for heterogeneity if present.<br>☐ Investigated the causes of any heterogeneity<br>☐ They statistically combined effect estimates from NRSI that were adjusted for confounding, rather than combining raw data, or justified combining raw data when adjusted effect estimates were not available<br>☐ None of the above<br>☐ The meta analysis did not include any NRSI's
2 = Yes | 1 = Partial yes | 0 = No	______________________________
Did the "meta analysis for NRSI" question reveal any moderate or critical weaknesses?	◯ Yes, critical weakness<br>◯ Yes, moderate weakness<br>◯ No
Briefly describe the weakness	__________________________________
12. Did the review authors assess the potential impact of RoB in individual studies on the results of the meta-analysis or other evidence synthesis?	◯ Included only low risk of bias RCTs<br>◯ If the pooled estimate was based on RCTs and/or NRSI at variable RoB, the authors performed analyses to investigate possible impact of RoB on summary estimates of effect.<br>◯ No
2 = Yes | 1 = Partial yes | 0 = No	______________________________

Did the "RoB impact" question reveal any moderate or critical weaknesses?	◯ Yes, critical weakness<br>◯ Yes, moderate weakness<br>◯ No
Briefly describe the weakness	__________________________________
13. Did the review authors account for RoB in individual studies when interpreting/ discussing the results of the review?	◯ Included only low risk of bias RCTs<br>◯ If RCTs with moderate or high RoB, or NRSI were included the review provided a discussion of the likely impact of RoB on the results<br>◯ No
2 = Yes | 1 = Partial yes | 0 = No	____________________________
Did the "RoB interpretation" question reveal any moderate or critical weaknesses?	◯ Yes, critical weakness<br>◯ Yes, moderate weakness<br>◯ No
Briefly describe the weakness	__________________________________
14. Did the review authors provide a satisfactory explanation for, and discussion of, any heterogeneity observed in the results of the review?	◯ There was no significant heterogeneity in the results<br>◯ If heterogeneity was present the authors performed an investigation of sources of any heterogeneity in the results and discussed the impact of this on the results of the review<br>◯ No
2 = Yes | 1 = Partial yes | 0 = No	____________________________
Did the "heterogeneity" question reveal any moderate or critical weaknesses?	◯ Yes, critical weakness<br>◯ Yes, moderate weakness<br>◯ No
Briefly describe the weakness	__________________________________
15. Did the review authors carry out an adequate investigation of publication bias (small study bias) and discuss its likely impact on the results of the review?	◯ Performed graphical or statistical tests for publication bias and discussed the likelihood and magnitude of impact of publication bias<br>◯ No
2 = Yes | 1 = Partial yes | 0 = No	____________________________
Did the "publication bias" question reveal any moderate or critical weaknesses?	◯ Yes, critical weakness<br>◯ Yes, moderate weakness<br>◯ No

Briefly describe the weakness

____

16. Did the review authors report any potential sources of conflict of interest, including any funding they received for conducting the review?

- ◯ The authors reported no competing interests
- ◯ The authors described their funding sources and how they managed potential conflicts of interest
- ◯ No

2 = Yes | 1 = Partial yes | 0 = No

____

Did the "conflict of interest" question reveal any moderate or critical weaknesses?

- ◯ Yes, critical weakness
- ◯ Yes, moderate weakness
- ◯ No

Briefly describe the weakness

____

Were there any other flaws (critical or non-critical) noted during the AMSTAR 2 assessment?

- ◯ Yes
- ◯ No

Please decribe the flaws noted

____

The table provides a rough overview based on the answers above - but AMSTAR 2 is NOT exhaustive and critical flaws that are not reported via the AMSTAR 2 assessment tool should still play a role in the overall assessment

Question

Yes (2), Partial yes (1), No (0)

Weakness: Critical (2), Moderate (1), No (0)

1. PICO

[amstar_pico_sum]

[amstar_pico_weak]

2. Protocol

[amstar_protocol_sum]

[amstar_protocol_weak]

3. Design

[amstar_design_sum]

[amstar_design_weak]

4. Search strategy

[amstar_search_sum]

[amstar_search_weak]

5. Study selection

[amstar_selection_sum]

[amstar_selection_weak]

6. Extraction

[amstar_extraction_sum]

[amstar_extraction_weak]

7. Exclusion

[amstar_exclusion_sum]

[amstar_exclusion_weak]

8. PICO details

[amstar_details_sum]

[amstar_details_weak]

9. RoB in RCT's

[amstar_rob_rct_sum]

[amstar_rob_rct_weak]

9. RoB in NRSI's

[amstar_rob_nrsi_sum]

[amstar_rob_nrsi_weak]

10. Study funding

[amstar_funding_sum]

[amstar_funding_weak]

11. Statistics RCT

[amstar_meta_rct_sum]

[amstar_meta_rct_weak]

11. Statistics NRSI

[amstar_meta_nrsi_sum]

[amstar_meta_nrsi_weak]

12. RoB impact

[amstar_meta_rob_sum]

[amstar_meta_rob_weak]

13. RoB interpretation

[amstar_res_rob_sum]

[amstar_res_rob_weak]

14. Heterogeneity

[amstar_hetero_sum]

[amstar_hetero_weak]

15. Publication bias

[amstar_pubbias_sum]

[amstar_pubbias_weak]

16. COI

[amstar_coi_sum]

[amstar_coi_weak]

What is the overall confidence in the study?<br><br>See guide below	☐ High<br>☐ Moderate<br>☐ Low<br>☐ Critically low<br>(Please see Shea et al. AMSTAR 2: a critical appraisal tool for systematic reviews that include randomised or non-randomised studies of healthcare interventions, or both. BMJ 2017;358: j4008)

Guide to rating confidence level:

High
(No or one non-critical weakness: the systematic review provides an accurate and comprehensive summary of the results of the available studies that address the question of interest

Moderate
(More than one non-critical weakness*: the systematic review has more than one weakness but no critical flaws. It may provide an accurate summary of the results of the available studies that were included in the review

Low
(One critical flaw with or without non-critical weaknesses: the review has a critical flaw and may not provide an accurate and comprehensive summary of the available studies that address the question of interest

Critically low
(More than one critical flaw with or without non-critical weaknesses: the review has more than one critical flaw and should not be relied on to provide an accurate and comprehensive summary of the available studies

*Multiple non-critical weaknesses may diminish confidence in the review and it may be appropriate to move the overall appraisal down from moderate to low confidence

FILL AT CONSENSUS ONLY

How did you agree to rate the overall confidence level of the study?

- ☐ High
- ☐ Moderate
- ☐ Low
- ☐ Critically low

3. Study description (non-repeatable)

Record ID

____

Instrument 3 contents

Study details
Outcomes   TSA documentation
Study summary and conclusions
GRADE   Misc. comments

To whom were the intervention group(s) compared (what is the type of comparator)?

- ☐ Standard care
- ☐ Other active intervention
- ☐ Placebo
- ☐ No treatment
- ☐ Other

Please describe the comparator

____

Was the literature search date defined?

- ◯ Yes
- ◯ No

Date of literature search (final date)

____
(If authors state month only, e.g, December 2019, choose the first day of the month, i.e., 01-12-2019)

Outcomes

Authors classify outcome measures inconsistently. In this database, outcomes are classified by the following definitions:

Primary or "main" outcome(s): The central research question(s) of the study and the reason that the study is done

Secondary or "additional" outcome(s): Research question(s) applied to all participants of the included studies. Provides additional information on the intervention effect.

Exploratory outcome(s): Research question(s) applied to all participants of the included studies. Primarily used for hypothesis generation.

Subgroup analysis: analyses of any of the outcomes described above, but in a subset of the sample population or the included trials. Can provide information on sources of heterogeneity (variations in demographics, intervention, etc.)
(Cochrane definition: https://handbook-5-1.cochrane.org/chapter_9/9_6_2_what_are_subgroup_analyses.htm)

How many primary outcomes were investigated in this study?

____

List all primary outcomes in study

UDGÅR

____
(Place each outcome on a separate line)

List all primary outcomes in the article ______ ______ ______ ______ ______ ______ ______ ______ ______ ______
______ ______ ______ ______ ______ ______ ______ ______ ______ ______

If more than 20 primary outcomes, use the field below

Please describe outcomes here IF more than 20 primary outcomes or the outcomes were otherwise incompatible with the table above ______________________________

How were the primary outcomes labelled by the authors?
- ◯ "Primary"
- ◯ "Main"
- ◯ Not labelled
- ◯ Other

Please describe how the primary outcomes were labelled by the authors ______________________________

How many secondary outcomes were investigated in this study? ____________________

List all secondary outcomes in the article ______ ______ ______ ______ ______ ______ ______ ______ ______ ______
______ ______ ______ ______ ______ ______ ______ ______ ______ ______

If more than 20 secondary outcomes, use the field below

Please describe outcomes here IF more than 20 secondary outcomes or the outcomes were otherwise incompatible with the table above ______________________________

List all secondary outcomes in study UDGÅR ______________________________
(Place each outcome on a separate line)

How were the secondary outcomes labelled by the authors?
- ◯ "Secondary"
- ◯ "Additional"
- ◯ "Subgroup analysis"
- ◯ Not labelled
- ◯ Other

Please describe how the secondary outcomes were labelled by the authors ______________________________

How many exploratory outcomes were investigated in this study? ____________________

List all exploratory outcomes in the article ______

If more than 20 secondary outcomes, use the field below

Please describe outcomes here IF more than 20 exploratory outcomes or the outcomes were otherwise incompatible with the table above ______________________

List all exploratory outcomes in study UDGÅR ______________________
(Place each outcome on a separate line)

How were the exploratory outcomes labelled by the authors?
- ◯ "Exploratory"
- ◯ "Additional"
- ◯ "Observational"
- ◯ "Subgroup analysis"
- ◯ Not labelled
- ◯ Other

Please describe how the exploratory outcomes were labelled by the authors ______________________

Were any subgroup analysis performed?
- ◯ Yes
- ◯ No

To which outcome(s) were subgroup analyses applied?

Primary ______ ______
____

Secondary ______________________
____

Exploratory ______________________
____

Please describe the subgroup analysis performed in the study

UDGÅR ______________________
(Describe to which outcome(s) the subgroup analysis was performed)

Please describe the subgroup analysis performed in the study ______________________
(Describe how the subgroups were defined)

How were the subgroup analyses labelled by the authors?

- ◯ "Subgroup analysis"
- ◯ "Secondary"
- ◯ "Exploratory"
- ◯ "Additional"
- ◯ "Observational"
- ◯ Not labelled
- ◯ Other

Please describe how the subgroup analyses were labelled by the authors

____

To which outcome(s) were TSA applied?

TSA applied with sufficient data TSA applied but insufficient data (AIS < 5% RIS/DARIS) Intention of TSA but no data available TSA not applied (justified) TSA not applied (not justified)

Primary ______ ______

Secondary ______________________________

Exploratory ____

Subgroup analyses: define type of subgroup and to which outcome ______ ______ ______ ______ ______
Outcomes not listed above ______ ______ ______ ______ ______

UDGÅR Please list all outcomes and subgroup analyses to which the authors explicitly intended to apply TSA as described in the article

____
(Disregarding insufficient information sizes, missing data)

Authors may choose to apply TSA but not share the graph and only report TSA CI's - look closely!

UDGÅR

Which outcomes and subgroup analyses did the authors explicitly intend to apply TSA to (as described in the article)?

Authors may choose to apply TSA but not share the graph and only report TSA CI's - look closely!

[outc_prim_1]

UDGÅR If applicable, please list outcomes where TSA was performed, but could not produce an output due to too small sample size (e.g., AIS < 2% RIS)

____

UDGÅR If applicable, please list outcomes where TSA was intended, but could not be performed due to lack of data, i.e., no data available

____

UDGÅR

On which outcomes were TSA performed, but could not produce an output due to too small sample size (e.g., AIS < 2% RIS)

____

UDGÅR

To which outcomes where TSA was intended, but could not be performed due to lack of data, i.e., no data available

____

[outc_prim_1]

TSA - Documentation

Has the authors stated which version of the Trial Sequential Analysis software they used?

◯ Yes
◯ No

Was the Trial Sequential Analysis software correctly referenced?	◯ Yes<br>◯ No<br>◯ Not referenced<br>(Minimum requirement: exact software version e.g. 0.9.5.10 + source e.g. ctu.dk)
Reference	__________________________________<br>(Please provide information. If "No" state which references were actually provided or "no references provided".)
Was the choice of statistical methods for Trial Sequential Analysis referenced?	◯ Yes<br>◯ No<br>(Either the manual or relevant references should be cited)

Which reference(s) was used?	☐ Brok J et al. Trial sequential analysis reveals insufficient information size and potentially false positive results in many metaanalyses. J. Clin. Epidemiol.2008;61 (8), 763-769.<br>☐ Brok et al. Apparently conclusive meta-analyses may be inconclusive: trial sequential analysis adjustment of random error risk due to repetitive testing of accumulating data in apparently conclusive neonatal meta-analyses. Int J Epidemiol. 2009;38(1):287-298<br>☐ Imberger G, Gluud C, Boylan J, Wetterslev J. Systematic Reviews of Anesthesiologic Interventions Reported as Statistically Significant: Problems with Power, Precision, and Type 1 Error Protection. Anesth Analg. 2015;121:1611-1622<br>☐ Imberger G et al. False-positive findings in Cochrane meta-analyses with and without application of trial sequential analysis: an empirical review. British Medical Journal Open 2016; 6: e011890<br>☐ Jakobsen JC et al. Thresholds for statistical and clinical significance in systematic reviews with meta-analytic methods. BMC Med Res Methodol 2014;14:120.<br>☐ Pogue JM, Yusuf S. Cumulating evidence from randomized trials: utilizing sequential monitoring boundaries for cumulative meta-analysis. Control Clin Trials. 1997;18:580-593.<br>☐ Thorlund K et al. Can trial sequential monitoring boundaries reduce spurious inferences from meta-analyses? Int J Epidemiol 2009;38:276-86.<br>☐ Thorlund K et al. User manual for Trial Sequential Analysis (TSA). Available: www.ctu.dk/tsa/files/tsa_manual.pdf<br>☐ Wetterslev J et al. Trial sequential analysis may establish when firm evidence is reached in cumulative meta-analysis. J. Clin. Epidemiol. 2008;61 (1), 64-75.<br>☐ Wetterslev J et al. Estimating required information size by quantifying diversity in random-effects model meta-analyses. BMC Med Res Methodol. 2009;9:86<br>☐ Wetterslev J et al. Trial sequential analysis in systematic reviews with meta-analysis. BMC Med Res Methodol 2017;17:39.<br>☐ Other
Other references	__________________________________<br>(Please insert complete reference - If the reference is relevant inform Christian Riberholt by e-mail)
Did the authors provide a TSA-report (or other formative report)?	◯ Yes<br>◯ No<br>(Check supplementary materials)
Study summary and conclusions	

How many RCT's were included in the article?

____
(Please list the actual number of RCT included.)

How many RCT's were included in the largest meta analysis?

____
(Largest by sample size. If the RCT has multiple groups and, therefore, is included in the meta-analysis more than once, count each data entry and make a note in the 'general comment field' below.)

How many non-randomized trials were included in the article?

____

How many non-randomized trials were included in the largest meta analysis?

____
(Largest by sample size)

Was the meta analysis presented in a forest plot?

- ◯ Yes
- ◯ No

The following questions ask for the FINAL conclusions of the study and not the "subconclusions" of the individual analyses, i.e., the results of individual TSA analyses or forest plots

---

What did the authors conclude about the intervention effect on the outcome(s) analyzed with TSA?

Outcome(s) analyzed with TSA:
[outc_tsa]

- ◯ Inconclusive
- ◯ Beneficial
- ◯ Harmful
- ◯ Futile
- ◯ Other/multiple

(Reminder to read the entire conclusion - authors may conclude that data indicates effect, only to conclude that the data quality was too low and thus the final conclusion is "inconclusive")

Please describe the conclusion (or copy/paste)

____

Were there any conclusions on the waste of research (relevant when limits for benefit, futility or harm are reached) ?

- ◯ Yes
- ◯ No

(When trials have been made far past RIS/DARIS, the authors may conclude that excessive or unneccessary trials have been made.)

What did the authors conclude about the waste of research?

____

GRADE

Was GRADE used for one or more outcomes to which TSA was applied?

- ◯ Yes, one
- ◯ Yes, more than one
- ◯ No

(GRADE = Grading of Recommendations, Assessment, Development and Evaluations)

(including outcomes where TSA wasn't performed due to lack of data)

How many outcomes were GRADE'd AND analyzed with TSA?

(including outcomes where TSA wasn't performed due to lack of data)

____

UDGÅR

Describe to which outcome(s) GRADE was applied

____
(Describe only outcomes to which TSA was applied. You noted, that the following outcomes were not analyzed: [outc_tsa_miss])

To which outcome(s) were GRADE applied?

Primary ______ ______ ____For outcomes not listed:______
Secondary ____________________________________ ____For outcomes not listed:______
Exploratory ____ ____For outcomes not listed:______

Do you have any further comments on the application of GRADE?

____

Did the Trial Sequential Analysis explicitly influence the GRADE assessment?

◯ Yes ◯ No

Describe how Trial Sequential Analysis influenced GRADE evaluation

____

How was the outcome GRADE'd?

UDGÅR

◯ High
◯ Moderate
◯ Low
◯ Very low

How were the outcomes GRADE'd?

☐ High
☐ Moderate
☐ Low
☐ Very low

Were the downgrade methods for imprecision described?

◯ Yes
◯ No

Which parameters influenced the grading of imprecision in GRADE?

☐ Imprecision downgraded 2 levels if DARIS < 50%; 1 level if DARIS between 50-100%; 0 levels if DARIS is reached 100% or if the cumulative Z-curve crosses boundaries for futility, benefit or harm
☐ Imprecision downgraded but the cut-offs were different than (1) or not specified (describe below)
☐ Imprecision was evaluated using the Trial Sequential adjusted confidence interval
☐ Judgement of the width of conventional 95% CI's
☐ Other

Describe how	__________________________________
How much was imprecision downgraded?	◯ 1 level<br>◯ 2 levels<br>◯ Imprecision was not explicitly downgraded<br>◯ Other
How much was imprecision downgraded?	☐ 1 level<br>☐ 2 levels<br>☐ Imprecision was not explicitly downgraded<br>☐ Other
Please describe how imprecision was downgraded	__________________________________
Misc.	
General comments on study issues or other noteworthy details not described elsewhere:	__________________________________<br>(Please fill in general comments that has not been elucidated in the questionaire)
Did the article mention/apply Bayesian approaches to statistical analysis?<br><br>(search article for "bayes")	◯ Yes<br>◯ No<br>(This question is used to screen for Bayesian content for a potential future project)

4. TSA Description (repeatable)

Record ID ______________________________

Instrument 4 contents

TSA - Outcome details TSA - Methods Dichotomous/continuous TSA - Graphical presentation and results Graphics Results

TSA - Outcome details

Please list all outcomes and subgroups analyzed with TSA (only in first instance of "Instrument 4 - TSA description)

In the previous instrument, you listed the following:
[outc_tsa]

And noted that TSA wasn't performed on the following outcomes due to lack of data:
[outc_tsa_miss]

UDGÅR

____

How many outcomes were analyzed with TSA? UDGÅR

____
(Those that were actually analyzed, i.e., not those with no data available - including subgroup analyses)

REMINDER! When multiple outcomes have been analyzed with TSA, repeat the TSA instrument and extract data from one dichotomous and one continuous outcome measure if applicable.

Dichotomous outcomes
a - Identify TSAs performed on dichotomous outcome measures (if NA, skip and go to 2a)
b - if a>1, identify TSAs from highest rank (primary>secondary>explorative>subgroup)
c - if b>1, identify TSA with highest acquired information size (AIS)
d - if c>1, identify TSA with highest AIS/RIS
e - if d>1, pick the one that appears in the article first Continuous outcomes
a - Identify TSAs performed on continuous outcome measures (if NA, skip)
b - if a>1, identify TSAs from highest rank (primary>secondary>explorative>subgroup)
c - if b>1, identify TSA with highest acquired information size (AIS)
d - if c>1, identify TSA with highest AIS/RIS
e - if d>1, pick the one that appears in the article first

What is the outcome to which TSA was applied?

UDGÅR

____
(Please copy/paste from paper)

What is the outcome to which TSA was applied (pick only one!)

Primary ______ ______

Secondary ________________________________

Exploratory ____

Subgroup analyses ______
If the outcome to which TSA was applied is not listed above, please describe here: ______

What is the data type for the outcome?	◯ Dichotomous<br>◯ Continuous
What is the type of outcome?<br><br>UDGÅR - overflødigt	◯ Primary<br>◯ Secondary<br>◯ Exploratory<br>◯ Subgroup analysis
Was the outcome analyzed with TSA analyzed in a network meta analysis (NMA)?	◯ Yes<br>◯ No
Which analysis model was used for the outcome analyzed with TSA?	◯ Fixed-effect model<br>◯ Random-effects models<br>◯ Fixed- and random-effects models<br>◯ Other<br>◯ None
Please provide additional model details and/or describe other models	__________________________________
Did the authors conclude that there was insufficient data, e.g., AIS < 5% RIS	◯ Yes<br>◯ No

TSA - Methods

This instrument asks for the details of the TSA analysis. Often, only one alpha-level will be chosen for all analyses (TSA, forest plots, etc.) but in the case that alpha-levels (or other details) differ, please note the value used for the TSA analysis and describe the difference in comments at the end of the instrument.

Values for each TSA should be presented in the figure, above the figure (figure head) or below the figure (legend), but will often be found in the description of statistical methods and/or results section.

Was the alpha-level defined?	◯ Yes<br>◯ Yes, conventional at 0.05<br>◯ No<br>(Provide values under comments)
Provide the alpha-level defined	___________________________<br>(Please provide as number, not percentage (e.g., 0.05 and not 5))
Describe the choice of alpha, if possible	__________________________________<br>((if alpha=0.05, note: "conventional"))
Was the alpha level explicitly corrected for multiplicity?	◯ Yes<br>◯ No
Which method was used to adjust alpha for multiplicity?	☐ Bonferroni<br>☐ Other
Please describe the method used for multiplicity correction	__________________________________

Was the power defined?	◯ Yes<br>◯ Yes, conventional at 0.8<br>◯ Yes, conventional at 0.9<br>◯ No
Provide the power defined	____________________________________<br>(Please provide as number, not percentage (e.g., 0.8 and not 80))
Describe the choice of power used in the Trial Sequential Analysis	____<br>((if power=0.8 or 0.9, note: "conventional"))
Was heterogeneity correction used for Trial Sequential Analysis?	◯ Inconsistency (I-square)<br>◯ Diversity (D-square)<br>◯ Not described, but no heterogeneity found in meta analysis (I^2=0%)<br>◯ Not described / not clear<br>◯ Other<br>◯ No<br>(Model variance based = Diversity adjusted)
Describe the choice of heterogeneity used in the Trial Sequential Analysis	____
Was the value for heterogeneity correction provided?	◯ Yes<br>◯ No
Please provide values for heterogeneity correction	____________________________________
TSA methods - dichotomous outcome	
Which association measure was used for analysis in the dichotomous outcome?	☐ Relative risk (RR)<br>☐ Risk difference (RD)<br>☐ Odds Ratio (OR)<br>☐ Peto OR<br>☐ Other
Please provide information on association measures	____
Was it relevant to correct for zero events in one group?<br>(i.e. did one group have zero events?)	◯ Yes<br>◯ No<br>◯ Unclear (not described)

How did they correct for zero events?	☐ Constant<br>☐ Reciprocal<br>☐ Empirical<br>☐ Correction value = 1<br>☐ Correction value = 0.5<br>☐ Correction value = 0.01<br>☐ Correction value = 0.001<br>☐ Trials with no events were excluded<br>☐ No correction described<br>☐ Other methods<br>(Check all relevant boxes)
Please describe the method used for correction of zero events	____
Was proportion of events in the control group (Pc) defined?	◯ Yes, with value<br>◯ Yes, but value not reported<br>◯ No<br>(Provide values under comments)
Where did the choice for the proportion of events in the control group (Pc) come from?	☐ From other published meta-analysis<br>☐ From one or more published randomised trials<br>☐ From one or more published observational studies<br>☐ From the observed data in their meta-analysis<br>☐ Unclear<br>☐ Other<br>☐ Not mentioned
Please describe the choice of Pc	____
Please provide the value defined for Pc	________________________________
Was the relative risk reduction (RRR) defined?	◯ Yes<br>◯ No
Please provide the RRR defined	________________________________<br>(Input as decimal number, not percentage)
Was it described how the relative risk reduction (RRR) was defined?	☐ From a published meta-analysis<br>☐ From one or more published randomised trials<br>☐ From one or more observational studies<br>☐ From the observed data in their meta-analysis<br>☐ Clinical experience (pick this if authors have assessed the RRR as "realistic" or similar)<br>☐ Other<br>☐ No
Describe the choice of RRR used in the Trial Sequential Analysis	____
TSA methods - continuous outcome	

Which association measure was used in the analysis for the continuous outcome?	☐ Mean difference (MD)<br>☐ Standardized mean difference (SMD)<br>☐ Other<br>(Weighted mean difference (WMD) is the same as MD)
Please provide information on association measures	__________________________________
Was variance defined?	◯ Yes<br>◯ No
Provide the variance defined	____________________________
Describe the choice of variance used in the Trial Sequential Analysis	__________________________________
Was MIREDIF defined?	◯ Yes<br>◯ No<br>("MIREDIF" = Minimal relevant difference. Provide values under comments)
Provide the MIREDIF defined	____________________________
Describe the choice of MIREDIF used in the Trial Sequential Analysis	__________________________________
TSA - Graphical presentation and results	
Graphics	
Was the Trial Sequential Analysis presented in a figure?	◯ Yes<br>◯ No
Were was the TSA graph presented?	◯ In article<br>◯ In supplementary materials<br>◯ Other
Please insert reference to the TSA figure, e.g., "Figure 4"	____________________________
Please describe where the TSA graph was presented	__________________________________
Are the conventional 5% limits correctly outlined?	◯ Yes<br>◯ No<br>◯ Not outlined
Why was it not correct?	__________________________________

Was the area of benefit above or below the x-axis?	◯ Above<br>◯ Below
Is the monitoring boundary of benefit correctly outlined?	◯ Yes<br>◯ No<br>◯ No, not outlined due to software limitation if first trial exceeds RIS/DARIS<br>◯ No, not outlined (but not relevant with only negative Z-scores)
Why was it not correct?	__________________________________
Is the monitoring boundary of harm correctly outlined?	◯ Yes<br>◯ No<br>◯ No, not outlined due to software limitation if first trial exceeds RIS/DARIS<br>◯ No, not outlined (but not relevant with only positive Z-scores)
Why was it not correct?	__________________________________
Is the area of futility (the inner wedge) correctly outlined?	◯ Yes<br>◯ No<br>◯ No, not outlined due to software limitation if first trial exceeds RIS/DARIS or cumulative sample size doesn't reach area of futility<br>◯ Inner wedge not applied
Why was it not correct?	__________________________________
Is the line indicating required information size (RIS or DARIS) correctly outlined?	◯ Yes<br>◯ No<br>◯ No, not outlined due to software limitation if first trial exceeds RIS/DARIS
Why was it not correct?	__________________________________
Is the cumulative Z-curve correctly outlined?	◯ Yes<br>◯ No<br>◯ No (trial spacing equal and not based on trial sizes)<br>◯ Not outlined
Why was it not correct?	__________________________________
Was the graphical presentation of the TSA different or flawed in a way not already described?	◯ Yes<br>◯ No

Please describe flaws and/or differences

____

Results

How many trials were analyzed?

____
(If discrepancy between figure and text/table/forest plot - report from figure! If trials are included more than once due to multiple groups, count each data entry and make a note in the 'Misc. comments on TSA' at the end.)

What was the acquired information size?

____
(If NA, input '-99')

Was the AIS directly extracted from figure or figure legend or was it calculated by the extractor, e.g., from forest plots?

- ◯ Directly extracted (e.g., from figure, figure legend or text)
- ◯ Calculated (e.g., summing sample sizes from forest plot)

Did the authors present a required information size for the analysis?

- ☐ Yes, diversity-adjusted required information size (DARIS)
- ☐ Yes, non-adjusted required information size (RIS)
- ☐ Yes, but not clear if RIS or DARIS
- ☐ No

What was the estimated non-adjusted or diversity adjusted required information size (RIS or DARIS)?

____

Was the non-adjusted or diversity adjusted required information size (RIS or DARIS) estimated from number of events or population size?

- ◯ Number of events
- ◯ Population size
- ◯ Not available

If futility wedge is missing, area 6 is limited to the area past RIS/DARIS

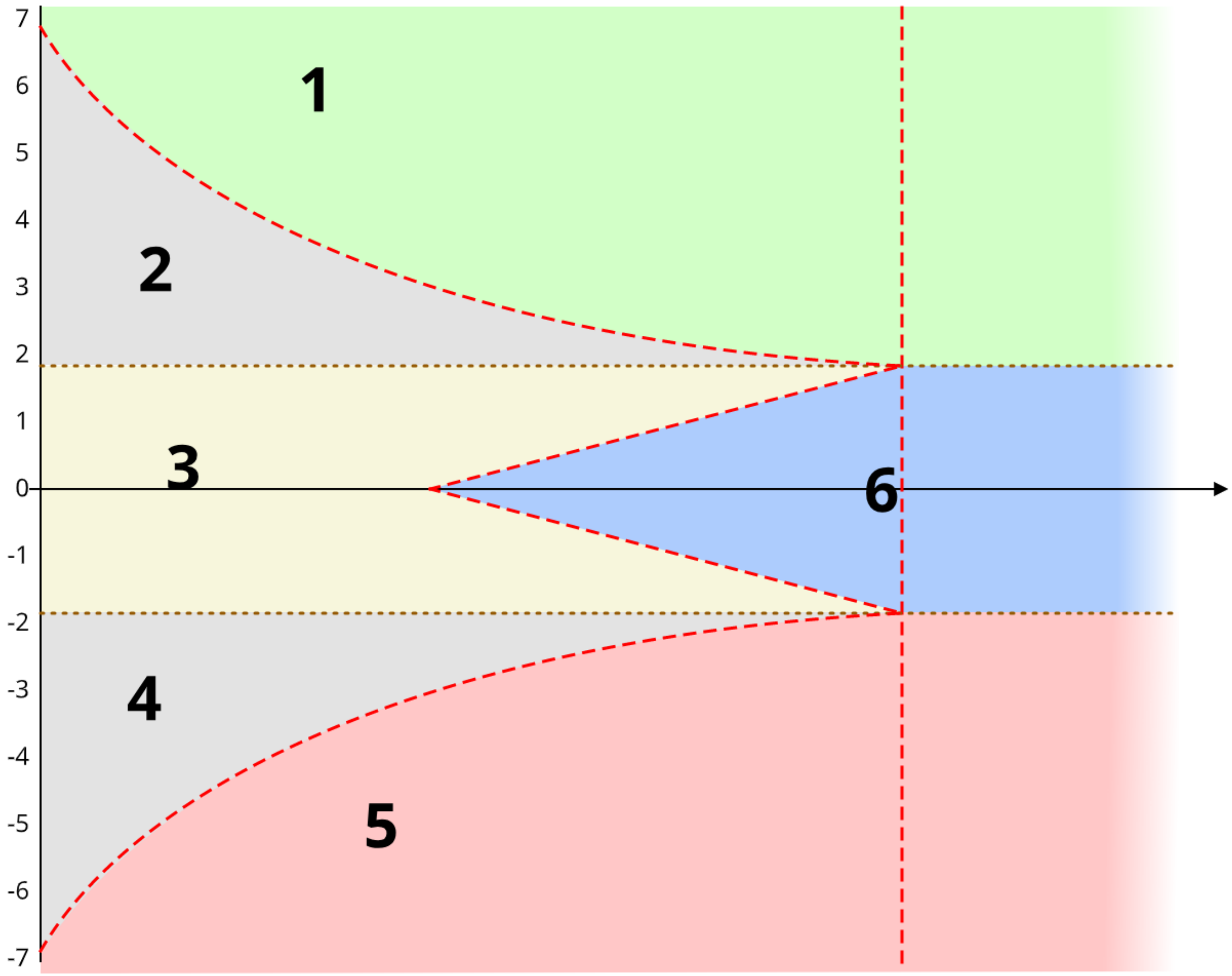

Please note in the table below were the individual trials of the Z-curve was placed in the TSA graphic (see area description in image above) - if TSA contains more than 84 studies, please leave a comment using the comment function and contact JBM

Trial number	TSA graph area (input integer: 1-6)	Trial number	TSA graph area (input integer: 1-6)	Trial number	TSA graph area (input integer: 1-6)
1	______	2	______	3	______
4	______	5	______	6	______
7	______	8	______	9	______
10	______	11	______	12	______
13	______	14	______	15	______
16	______	17	______	18	______
19	______	20	______	21	______
22	______	23	______	24	______
25	______	26	______	27	______
28	______	29	______	30	______
31	______	32	______	33	______
34	______	35	______	36	______
37	______	38	______	39	______
40	______	41	______	42	______
43	______	44	______	45	______
46	______	47	______	48	______
49	______	50	______	51	______
52	______	53	______	54	______
55	______	56	______	57	______
58	______	59	______	60	______
61	______	62	______	63	______
64	______	65	______	66	______
67	______	68	______	69	______
70	______	71	______	72	______
73	______	74	______	75	______

76 ______ 77 ______ 78 ______
79 ______ 80 ______ 81 ______
82 ______ 83 ______ 84 ______

Did the authors provide Trial Sequential adjusted confidence intervals?

◯ Yes
◯ Yes, but mislabelled
◯ No
(Should be labelled as Trial Sequential adjusted confidence interval or TSA CI (not 95%CI))

In what way did they mislabel the Trial Sequential adjusted confidence interval?

____

What was the estimated effect size for the outcome analyzed with TSA?

Point estimate (e.g., mean dif, RR)

______

Conventional CI, lower limit

______

Conventional CI, upper limit

______

TSA adjusted CI, lower limit

______

TSA adjusted CI, upper limit

______

How was the outcome GRADE'd?

◯ High
◯ Moderate
◯ Low
◯ Very low
◯ The outcome was not GRADE'd

How much was imprecision downgraded?

◯ 1 level
◯ 2 levels
◯ Imprecision was not explicitly downgraded
◯ Other

Misc. comments on TSA not considered elsewhere

____

Please rate the overall transparency of the TSA analysis<br><br>You may consider the following variables:<br><br>Discrepancies between graph and text or between text segments Graphic quality of figure - including labels Specification of included trials Acquired information size Required information size (RIS or DARIS) Relative risk reduction or MIREDIF Heterogeneity Proportion of events in the control population (only dichotomous outcomes) Variance (only continuous outcomes) Chosen association measure	◯ Excellent / really good (all parameters transparently presented)<br>◯ Good (few parameters missing)<br>◯ Poor (several important parameters missing)<br>◯ Useless (graph unreadable and/or crucial parameters missing)<br>(Transparency almost equal to reproducibility)
Which parameters impaired the transparency of the TSA ?	☐ Discrepancies between graph and text or between text segments<br>☐ Graphic quality of figure - including labels<br>☐ Specification of included trials<br>☐ Acquired information size<br>☐ Required information size (RIS or DARIS)<br>☐ Relative risk reduction or MIREDIF<br>☐ Heterogeneity<br>☐ Proportion of events in the control population (only dichotomous outcomes)<br>☐ Variance (only continuous outcomes)<br>☐ Chosen association measure<br>☐ Other
Please describe which other parameters influenced your rating	__________________________________

5. Protocol description (non-repeatable)

Record ID ______________________________

Instrument 5 contents

Protocol availability
Coherence check
Outcomes Planning of TSA Planning of dichotomous outcomes Planning of continuous outcomes Planning of GRADE

Protocol availability

Has a protocol been made public before data extraction began?
- ◯ Yes
- ◯ No
- ◯ Unclear

Do you have any comments? ______________________________

Where has the protocol been made publicly available?
- ☐ 1. Scientific journal
- ☐ 2. PROSPERO
- ☐ 3. Web-page
- ☐ 4. Other

(If available from multiple places (e.g. Scientific journal and Prospero) use highest ranked when moving forward)

Please insert link to the protocol in the box ______________________________
(Webpage; CRD-number; DOI; PubmedID etc.)

Date the protocol was first submitted ______________________________

Date the protocol was first published ______________________________

Was the protocol updated, i.e., are more than one version of the protocol available?
- ◯ Yes
- ◯ No

Please provide the dates at which the protocol versions were published (including the first)

Version DATE (Y-M-D)
First version ______
Second version ______
Third version ______
Fourth version ______
Fifth version ______

Was the complete search strategy publicly available before the literature search was completed?

Literature search completed:

[adddet_litdate]

◯ Yes
◯ No
◯ Not clear

Please describe how it was not clear if the search strategy was predefined

____

Was the protocol publicly available before initiation of formal screening?

◯ Yes
◯ No
◯ Not clear

Please describe why it was not clear if protocol was defined prior to formal screening

____

Was the protocol publicly available before completion of formal screening?

◯ Yes
◯ No
◯ Not clear

Please describe why it was not clear if protocol was defined prior to completion of formal screening

____

Coherence check

Please extract information from the latest protocol version that was publicly available BEFORE DATA EXTRACTION

Outcomes

Were the outcomes in the article also defined in the protocol?

Yes No (or different)
Primary ______ ______
____ ______
______ ____

Secondary ______________________________
____
____
____

Exploratory ____
____
____
____

If the outcomes in the article are not listed above, describe here ______ ______

Please describe if differences between article and protocol have been observed.	__________________
Were any additional outcomes defined in the protocol?	◯ Yes<br>◯ No
In summary, which outcomes defined in the protocol are DIFFERENT from those analyzed in the paper?	☐ Primary was different<br>☐ Secondary was different<br>☐ Exploratory was different<br>(CAREFULLY check each outcome. Check box if DIFFERENT)
How many primary outcomes are defined in the protocol?	__________________
List all primary outcomes of the protocol	__________________
Please comment on the differences between primary outcomes of protocol and the paper	__________________
How many secondary outcomes are defined in the protocol?	__________________
List all secondary outcomes of the protocol	__________________
Please comment on the differences between secondary outcomes of protocol and the paper	__________________
How many exploratory outcomes are defined in the protocol?	__________________
List all exploratory outcomes of the protocol	__________________
Please comment on the differences between exploratory outcomes of protocol and the paper	__________________
Were any dichotomous outcomes defined?	◯ Yes<br>◯ No
Was TSA planned for the dichotomous outcomes?	◯ Yes<br>◯ No<br>(Includes generic descriptions such as "TSA will be applied" without specification of outcome)
Were any continuous outcomes defined?	◯ Yes<br>◯ No

Was TSA planned for the continuous outcomes?	◯ Yes<br>◯ No<br>(Includes generic descriptions such as "TSA will be applied" withouth specification of outcome)
Was supgroup analysis planned in the protocol?	◯ Yes<br>◯ No

Was the supgroup analysis that was planned in the protocol the same as was conducted in the study?

◯ Yes
◯ No

You wrote about the subgroup analysis performed in the study:
[outc_subtxt_2]

To the following outcomes:

Primary Secondary Exploratory
[outc_prim_sub_1]
[outc_prim_sub_2]
[outc_prim_sub_3]
[outc_prim_sub_4]
[outc_prim_sub_5]
[outc_prim_sub_6]
[outc_prim_sub_7]
[outc_prim_sub_8]
[outc_prim_sub_9]
[outc_prim_sub_10]
[outc_prim_sub_11]
[outc_prim_sub_12]
[outc_prim_sub_13]
[outc_prim_sub_14]
[outc_prim_sub_15]
[outc_prim_sub_16]
[outc_prim_sub_17]
[outc_prim_sub_18]
[outc_prim_sub_19]
[outc_prim_sub_20]

[outc_prim_sub_comment]

[outc_sec_sub_1]
[outc_sec_sub_2]
[outc_sec_sub_3]
[outc_sec_sub_4]
[outc_sec_sub_5]
[outc_sec_sub_6]
[outc_sec_sub_7]
[outc_sec_sub_8]
[outc_sec_sub_9]
[outc_sec_sub_10]
[outc_sec_sub_11]
[outc_sec_sub_12]
[outc_sec_sub_13]
[outc_sec_sub_14]
[outc_sec_sub_15]
[outc_sec_sub_16]
[outc_sec_sub_17]
[outc_sec_sub_18]
[outc_sec_sub_19]
[outc_sec_sub_20]

[outc_sec_sub_comment]

[outc_exp_sub_1]
[outc_exp_sub_2]
[outc_exp_sub_3]
[outc_exp_sub_4]
[outc_exp_sub_5]
[outc_exp_sub_6]
[outc_exp_sub_7]
[outc_exp_sub_8]
[outc_exp_sub_9]
[outc_exp_sub_10]
[outc_exp_sub_11]
[outc_exp_sub_12]
[outc_exp_sub_13]

[outc_exp_sub_14]
[outc_exp_sub_15]
[outc_exp_sub_16]
[outc_exp_sub_17]
[outc_exp_sub_18]
[outc_exp_sub_19]
[outc_exp_sub_20]

[outc_exp_sub_comment]

How many subgroup analyses were planned?	______________________
Which subgroup analyses were planned?	______________________
Please describe differences between planned and performed subgroup analyses	______________________
Planning of Trial Sequential Analysis	
Did the protocol define that TSA would be applied?	◯ Yes ◯ No
Please copy/paste the description of the TSA plan	______________________ (If TSA is not described in one continuous text, please define segments with " ")
Was the choice of statistical methods for Trial Sequential Analysis referenced?	◯ Yes<br>◯ No<br>(Either the manual or relevant references should be cited)

Which reference(s) was used?	☐ Brok J et al. Trial sequential analysis reveals insufficient information size and potentially false positive results in many metaanalyses. J. Clin. Epidemiol. 2008;61 (8), 763-769.<br>☐ Brok et al. Apparently conclusive meta-analyses may be inconclusive: trial sequential analysis adjustment of random error risk due to repetitive testing of accumulating data in apparently conclusive neonatal meta-analyses. Int J Epidemiol. 2009;38(1):287-298<br>☐ Imberger G, Gluud C, Boylan J, Wetterslev J. Systematic Reviews of Anesthesiologic Interventions Reported as Statistically Significant: Problems with Power, Precision, and Type 1 Error Protection. Anesth Analg. 2015;121:1611-1622<br>☐ Imberger G et al. False-positive findings in Cochrane meta-analyses with and without application of trial sequential analysis: an empirical review. British Medical Journal Open 2016; 6: e011890<br>☐ Jakobsen JC et al. Thresholds for statistical and clinical significance in systematic reviews with meta-analytic methods. BMC Med Res Methodol 2014;14:120.<br>☐ Pogue JM, Yusuf S. Cumulating evidence from randomized trials: utilizing sequential monitoring boundaries for cumulative meta-analysis. Control Clin Trials. 1997;18:580-593.<br>☐ Thorlund K et al. Can trial sequential monitoring boundaries reduce spurious inferences from meta-analyses? Int J Epidemiol 2009;38:276-86.<br>☐ Thorlund K et al. User manual for Trial Sequential Analysis (TSA). Available: www.ctu.dk/tsa/files/tsa_manual.pdf<br>☐ Wetterslev J et al. Trial sequential analysis may establish when firm evidence is reached in cumulative meta-analysis. J. Clin. Epidemiol. 2008;61 (1), 64-75.<br>☐ Wetterslev J et al. Estimating required information size by quantifying diversity in random-effects model meta-analyses. BMC Med Res Methodol. 2009;9:86<br>☐ Wetterslev J et al. Trial sequential analysis in systematic reviews with meta-analysis. BMC Med Res Methodol 2017;17:39.<br>☐ Other
Other references	____________________________<br>(Please insert complete reference - If the reference is relevant inform Christian (Christian who?) by e-mail)
Did the protocol define to which outcomes TSA would be applied?	○ Yes<br>○ No

Were those outcomes the same as were eventually analyzed with TSA in the study?<br><br>(disregard discrepancies caused by lack of data)<br><br>The study applied TSA to the following [tsaout_num][first-instance] outcome(s):<br><br>[tsaout_list][first-instance]	◯ Yes<br>◯ No
Please describe differences between TSA outcomes in protocol and study	__________________________________
Which analysis model was planned for the outcome(s) analyzed with TSA?	◯ Fixed-effect model<br>◯ Random-effects models<br>◯ Fixed- and random-effects models<br>◯ Other<br>◯ The outcome to which TSA was applied was not defined in the protocol<br>◯ None
Please provide additional model details and/or describe other models if relevant	__________________________________
Was the alpha-level defined for the outcome analyzed with TSA?	◯ Yes<br>◯ Yes, conventional alpha at 0.05<br>◯ No<br>◯ The outcome to which TSA was applied was not defined in the protocol
Provide the alpha-level defined	____________________________<br>(Please provide as number, not percentage (e.g., 0.05 and not 5))
Describe the choice of alpha (if possible)	__________________________________
Was the alpha-level explicitly corrected for multiplicity?	◯ Yes<br>◯ No
Which method was chosen to adjust alpha for multiplicity?	☐ Bonferroni<br>☐ Other
Please describe the method chosen for multiplicity correction	__________________________________
Was the power defined for the outcome analyzed with TSA?	◯ Yes<br>◯ Yes, conventional at 0.80<br>◯ Yes, conventional at 0.90<br>◯ No<br>◯ The outcome to which TSA was applied was not defined in the protocol

Provide the power defined	__________________________________ (Please provide as number, not percentage (e.g., 0.8 and not 80))
Describe the choice of power used in the Trial Sequential Analysis	__________________________________
Was heterogeneity correction planned for Trial Sequential Analysis and provided?	◯ Inconsistency (I-square)<br>◯ Diversity (D-square)<br>◯ Other<br>◯ No
Please provide details if relevant	__________________________________
Did the authors plan to calculate TSA adjusted confidence intervals?	◯ Yes<br>◯ No
Planning of dichotomous outcomes	
Which association measure(s) was chosen for analysis of the dichotomous outcome(s)?	☐ Relative risk (RR)<br>☐ Risk difference (RD)<br>☐ Odds Ratio (OR)<br>☐ Peto OR<br>☐ Other<br>☐ Not described
Please provide information on association measures	__________________________________
Was handling of the analysis in case of zero events proposed?	◯ Yes<br>◯ No
What was the planned strategy for handling zero events?	☐ Constant<br>☐ Reciprocal<br>☐ Emperical<br>☐ Correction value = 1<br>☐ Correction value = 0.5<br>☐ Correction value = 0.01<br>☐ Correction value = 0.001<br>☐ Trials with no events were excluded<br>☐ Other methods
Please provide details	__________________________________
Did the protocol define the numerical value of Pc (Proportion of events in the control group)?	◯ Yes<br>◯ No
Please provide the value defined for Pc	__________________________________

Did the protocol define where the proportion of events in the control group (Pc) would come from?	☐ From other published meta-analysis<br>☐ From one or more published randomised trials<br>☐ From one or more published observational studies<br>☐ From the observed data in their own meta-analysis<br>☐ Unclear<br>☐ Other<br>☐ No
Was the relative risk reduction (RRR) defined?	○ Yes<br>○ No
Please provide the RRR defined	______________________________
Was it described how the relative risk reduction (RRR) was/would be defined?	☐ From a published meta-analysis<br>☐ From one or more published randomised trials<br>☐ From one or more observational studies<br>☐ From the observed data in their meta-analysis<br>☐ Clinical experience<br>☐ Other<br>☐ No
Describe the choice of RRR	_____________________________________
Planning of continuous outcomes	
Which association measure was chosen for the analysis of the continuous outcome(s)?	☐ Mean difference (MD)<br>☐ Standardized mean difference (SMD)<br>☐ Other<br>☐ Not defined
Please provide information on association measures	_____________________________________
Was variance defined in the protocol for continuous outcomes?	○ Yes, from published meta-analysis<br>○ Yes, from one or more published randomised trials<br>○ Yes, from one or more observational studies<br>○ Yes, the squared standard deviation of the observed mean difference<br>○ Other<br>○ No<br>(Provide values under comments)
Provide the variance(s) defined	______________________________
Describe the choice of variance	_____________________________________

Was the minimally relevant different (MIREDIF) defined for each relevant outcome?	◯ Yes, from published meta-analysis<br>◯ Yes, from one or more published randomised trials<br>◯ Yes, from one or more observational studies<br>◯ Yes, MIREDIF defined as observed SD/2<br>◯ Other<br>◯ No<br>(Provide values under comments)
Provide the MIREDIF(s) defined	__________________________________
Describe the choice of MIREDIF	__________________________________
Planning of GRADE	
Was GRADE assessment planned in the protocol	◯ Yes<br>◯ No
Did the protocol define which outcomes would undergo a GRADE assessment?	◯ Yes<br>◯ No
Was the outcome(s) planned for GRADE assessment the same as was assessed in the study?<br><br>GRADE assessment(s) in study:<br><br>[studcon_gradetxt]	◯ Yes<br>◯ No<br>(Only for outcomes to which TSA were applied)
Please describe differences between GRADE plan in protocol and GRADE in study	__________________________________
Was the downgrade methods for imprecision in GRADE described in the protocol?	◯ Yes ◯ No
Did the protocol describe how imprecision would be downgraded with the use of Trial Sequential Analysis?	☐ Yes, imprecision downgraded 2 levels if DARIS < 50%; 1 level if DARIS between 50-100%; 0 levels if DARIS is reached 100% or if the cumulative Z-curve crosses boundaries for futility, benefit or harm<br>☐ Yes, imprecision downgraded but the cut-offs were different than (1) or not specified (describe below)<br>☐ Yes, imprecision was evaluated using the Trial Sequential adjusted confidence interval<br>☐ Other<br>☐ No
Please describe the plan for downgrading imprecision with the use of TSA	__________________________________
Please describe the plan for downgrading imprecision	__________________________________

Do you have any additional comments?

____
